# Supplementary material for: Using DHIS2 routine data for health system preparedness in resource-limited settings: A Bayesian predictive approach in Bangladesh
Source: PLOS Glob Public Health. 2026 Mar 3;6(3):e0005231. doi: 10.1371/journal.pgph.0005231 (PMC12956080; doi:10.1371/journal.pgph.0005231)
Supplement: S1 File — (PDF) [file pgph.0005231.s001.pdf]

S1 File: Trend and prediction of maternal and neonatal health services

Bangladesh

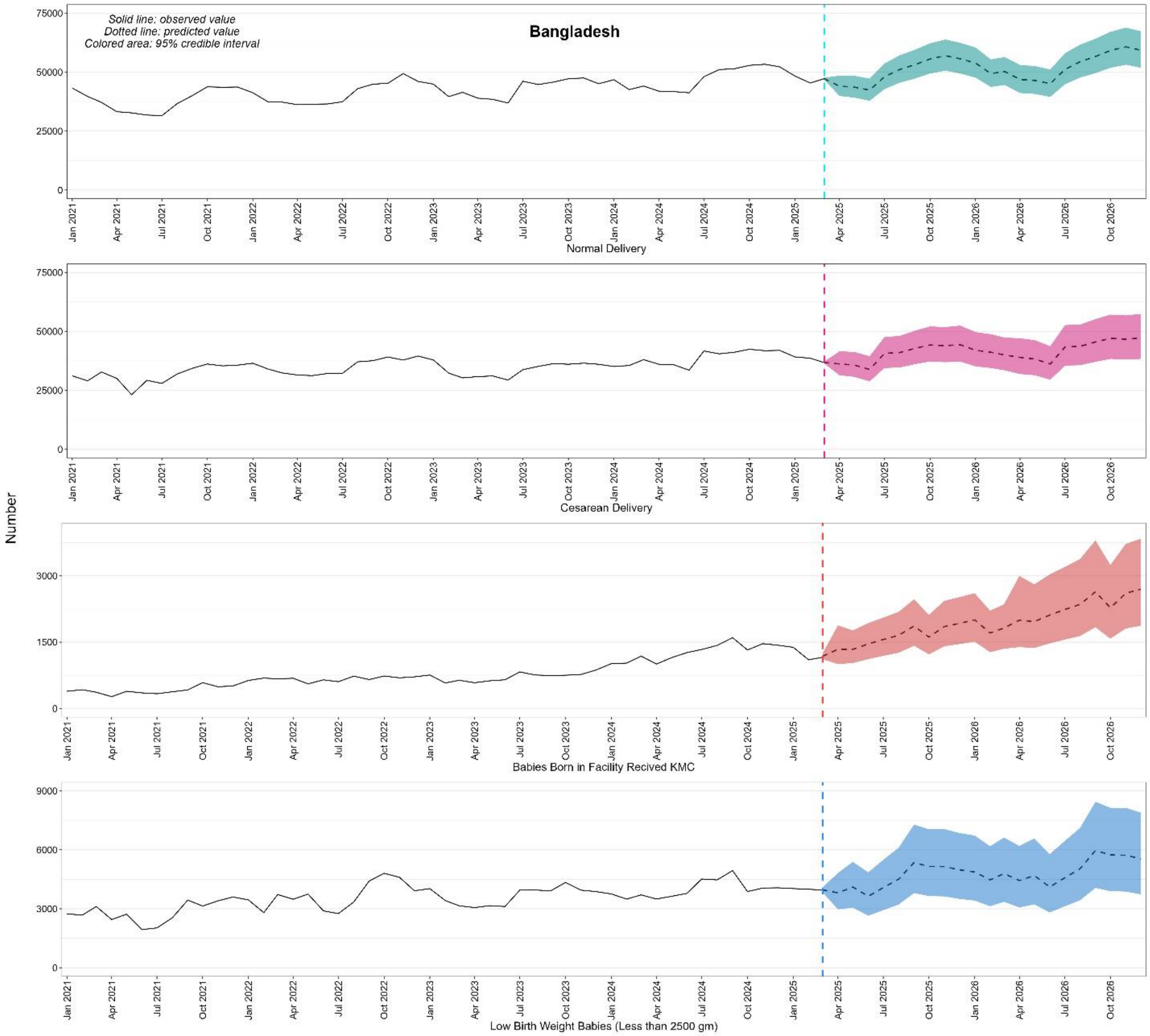

Barisal

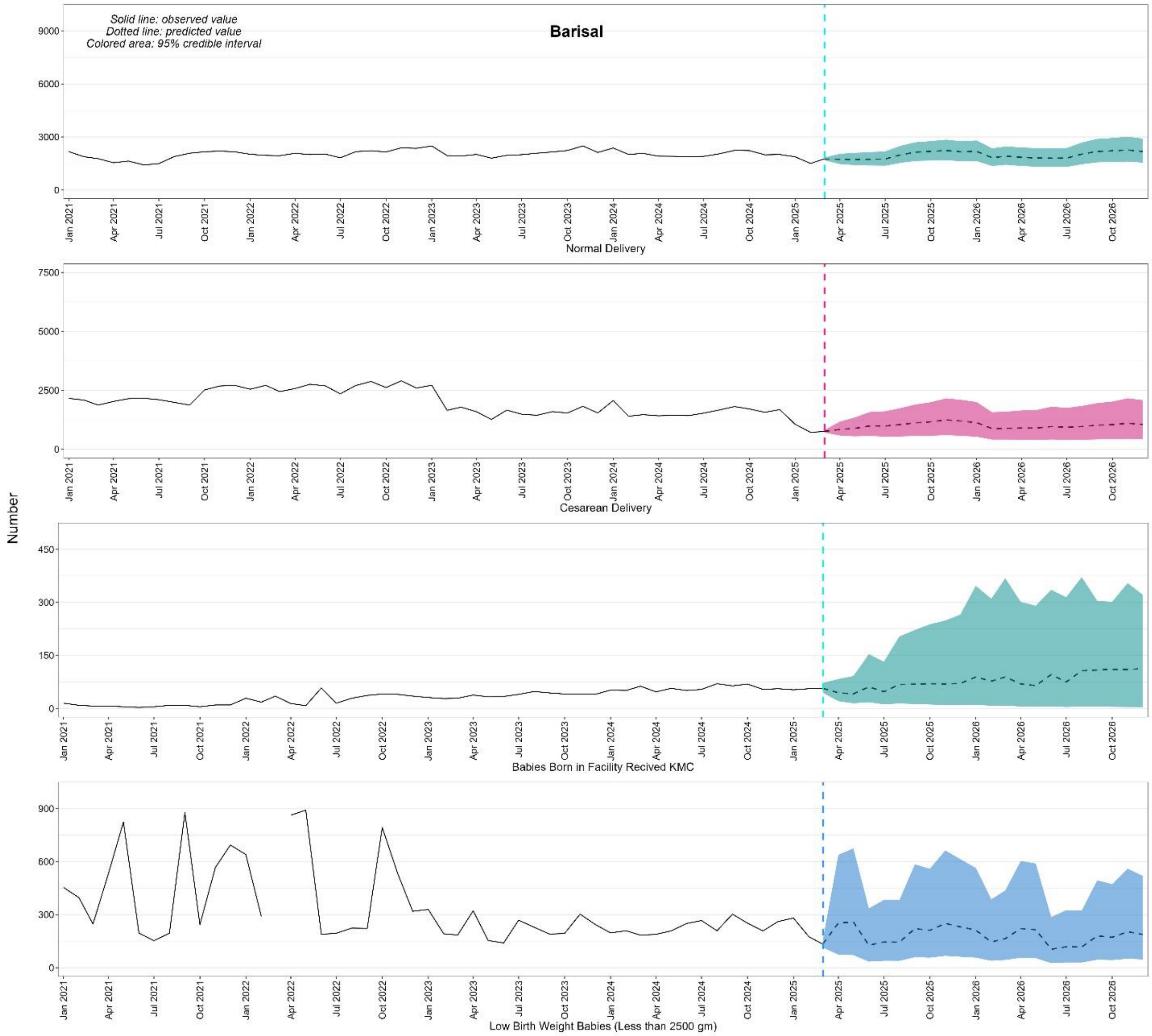
Solid line: observed value
Dotted line: predicted value
Colored area: 95% credible interval
Barisal
Number
Normal Delivery
Cesarean Delivery
Babies Born in Facility Recived KMC
Low Birth Weight Babies (Less than 2500 gm)

Chittagong

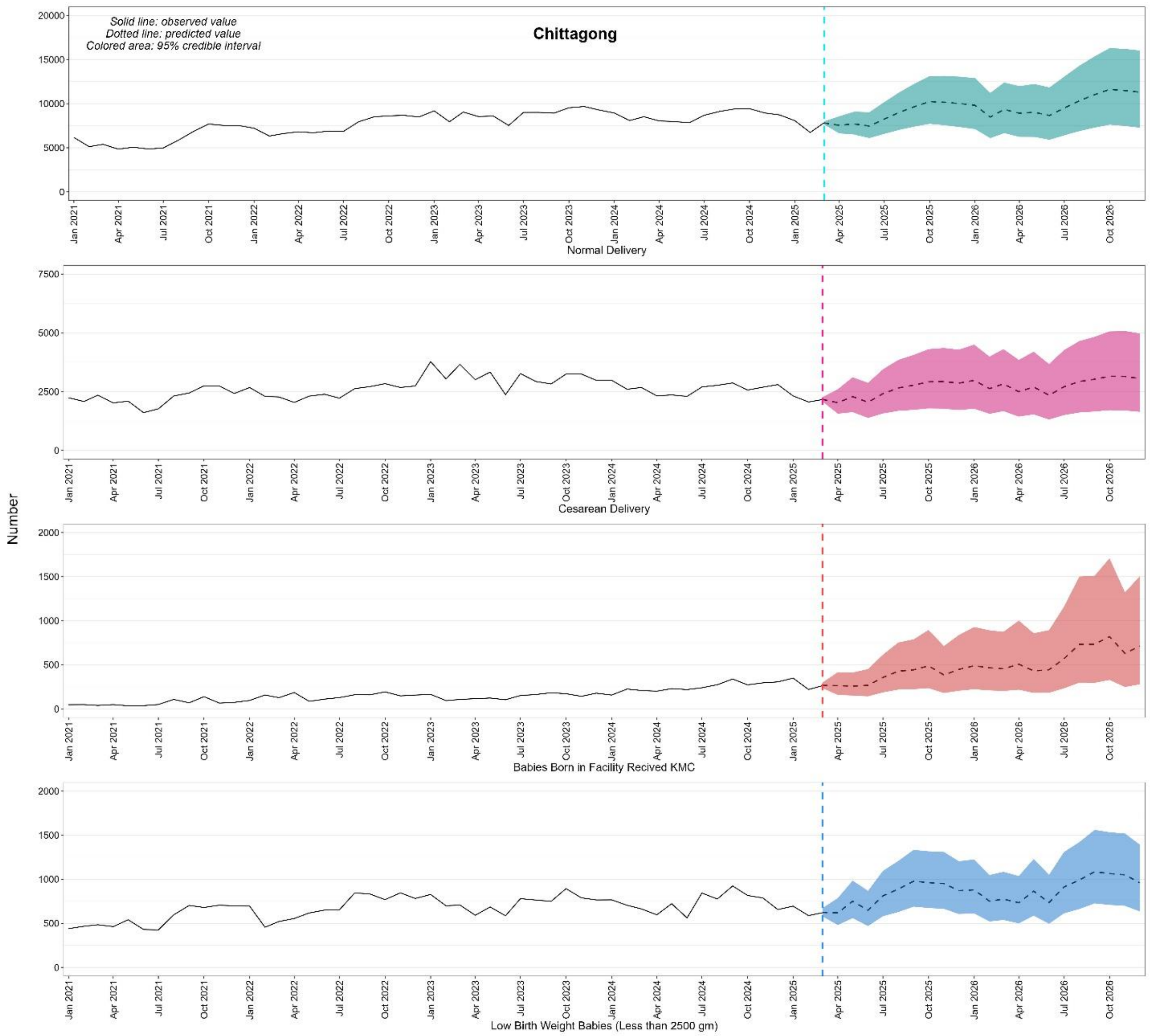
Solid line: observed value
Dotted line: predicted value
Colored area: 95% credible interval
Chittagong
Normal Delivery
Cesarean Delivery
Babies Born in Facility Recived KMC
Low Birth Weight Babies (Less than 2500 gm)
Number

Dhaka

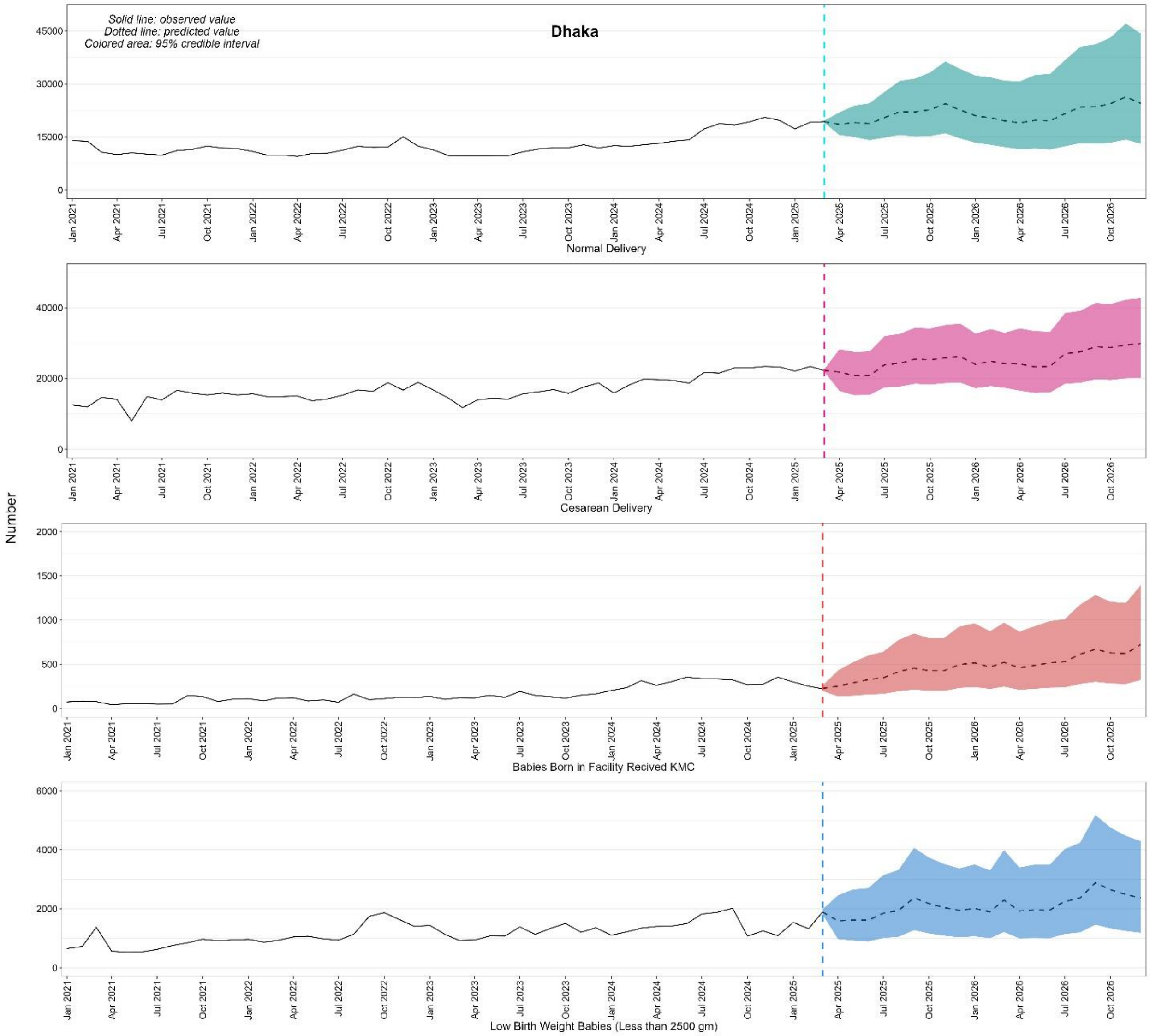
Solid line: observed value
Dotted line: predicted value
Colored area: 95% credible interval
Dhaka
Number
Normal Delivery
Cesarean Delivery
Babies Born in Facility Recived KMC
Low Birth Weight Babies (Less than 2500 gm)

Khulna

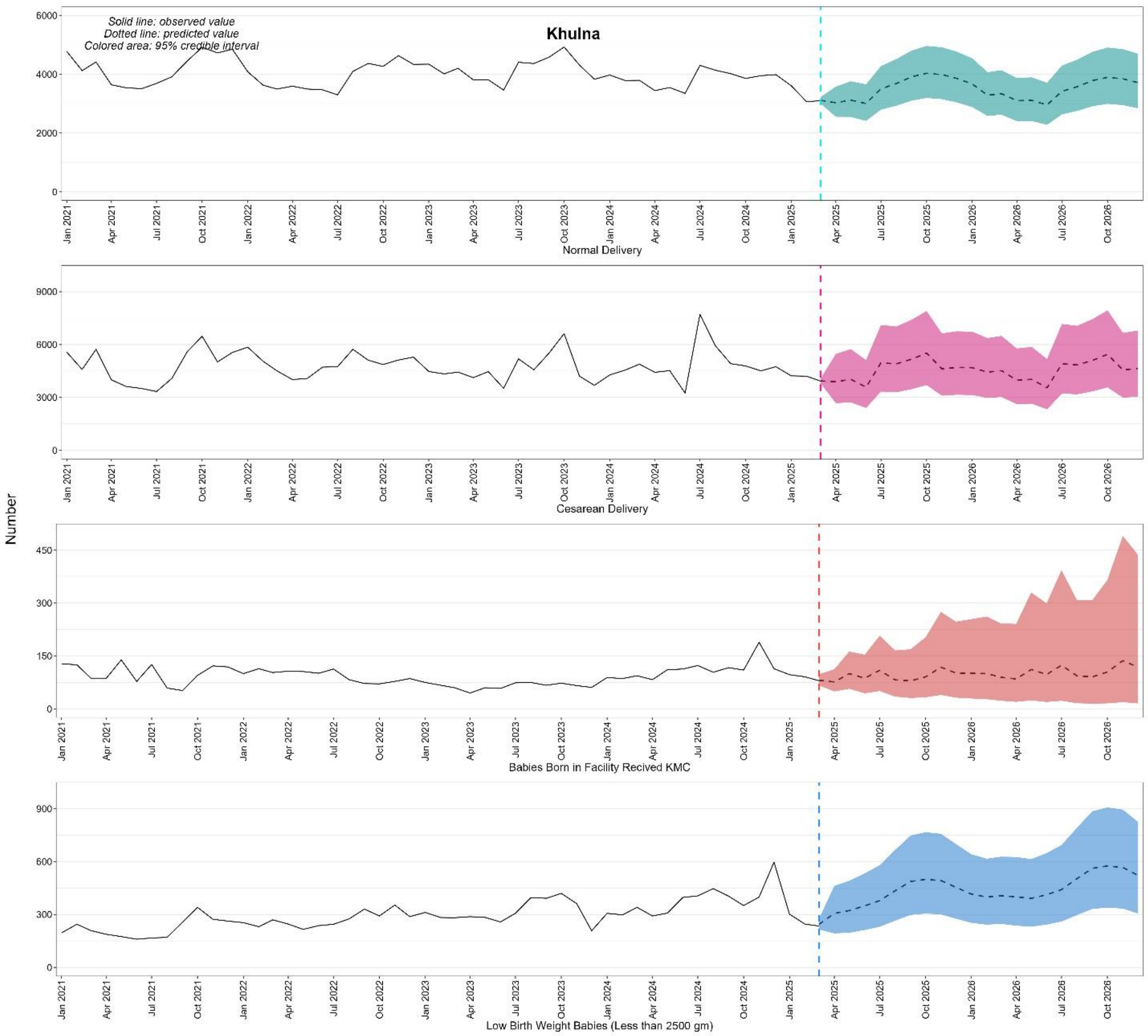
Khulna
Solid line: observed value
Dotted line: predicted value
Colored area: 95% credible interval
Number
Normal Delivery
Cesarean Delivery
Babies Born in Facility Recived KMC
Low Birth Weight Babies (Less than 2500 gm)

Mymensingh

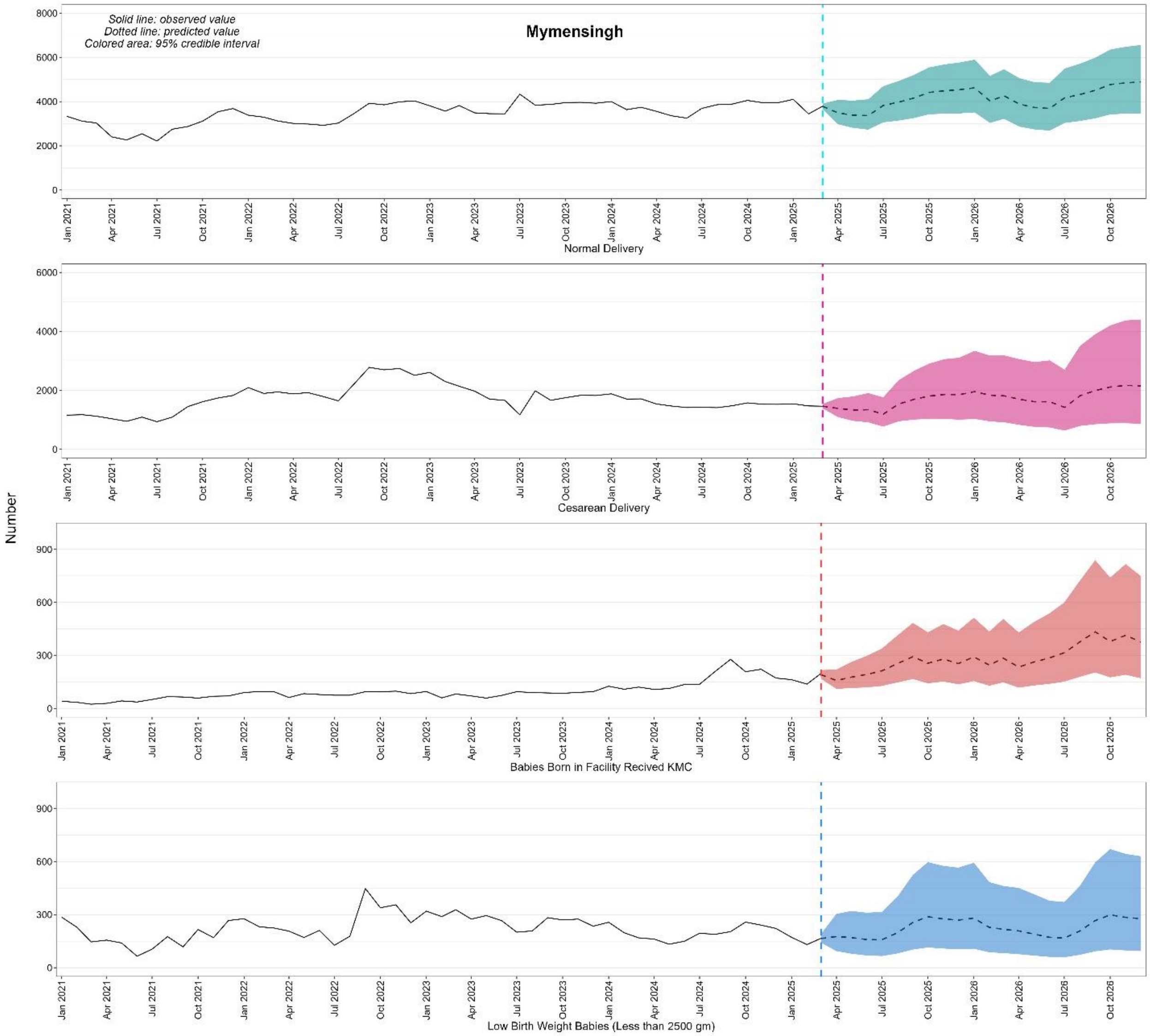
Solid line: observed value
Dotted line: predicted value
Colored area: 95% credible interval
Mymensingh
Number
Normal Delivery
Cesarean Delivery
Babies Born in Facility Recived KMC
Low Birth Weight Babies (Less than 2500 gm)

Rajshahi

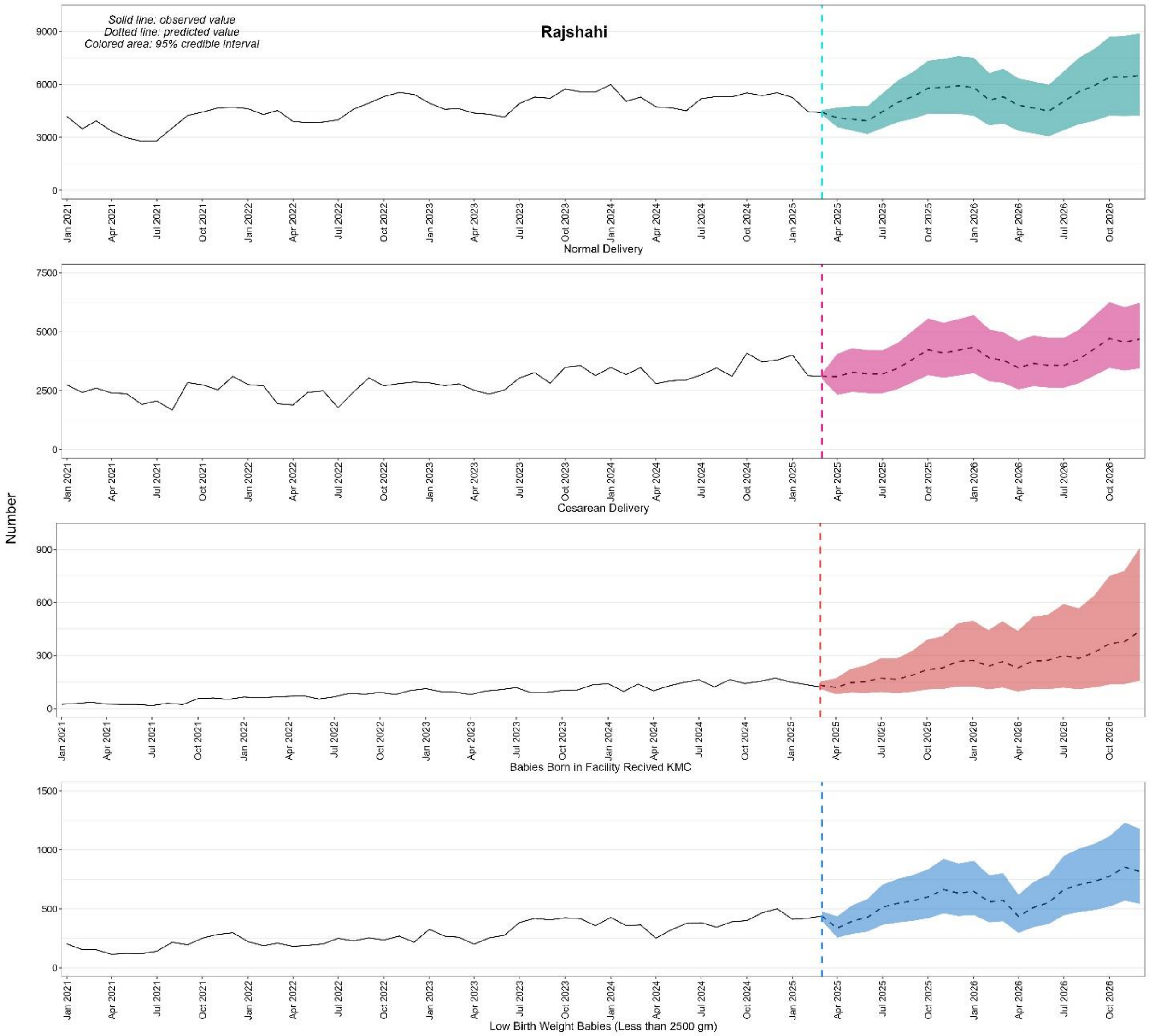
Solid line: observed value
Dotted line: predicted value
Colored area: 95% credible interval
Rajshahi
Number
Normal Delivery
Cesarean Delivery
Babies Born in Facility Recived KMC
Low Birth Weight Babies (Less than 2500 gm)

Rangpur

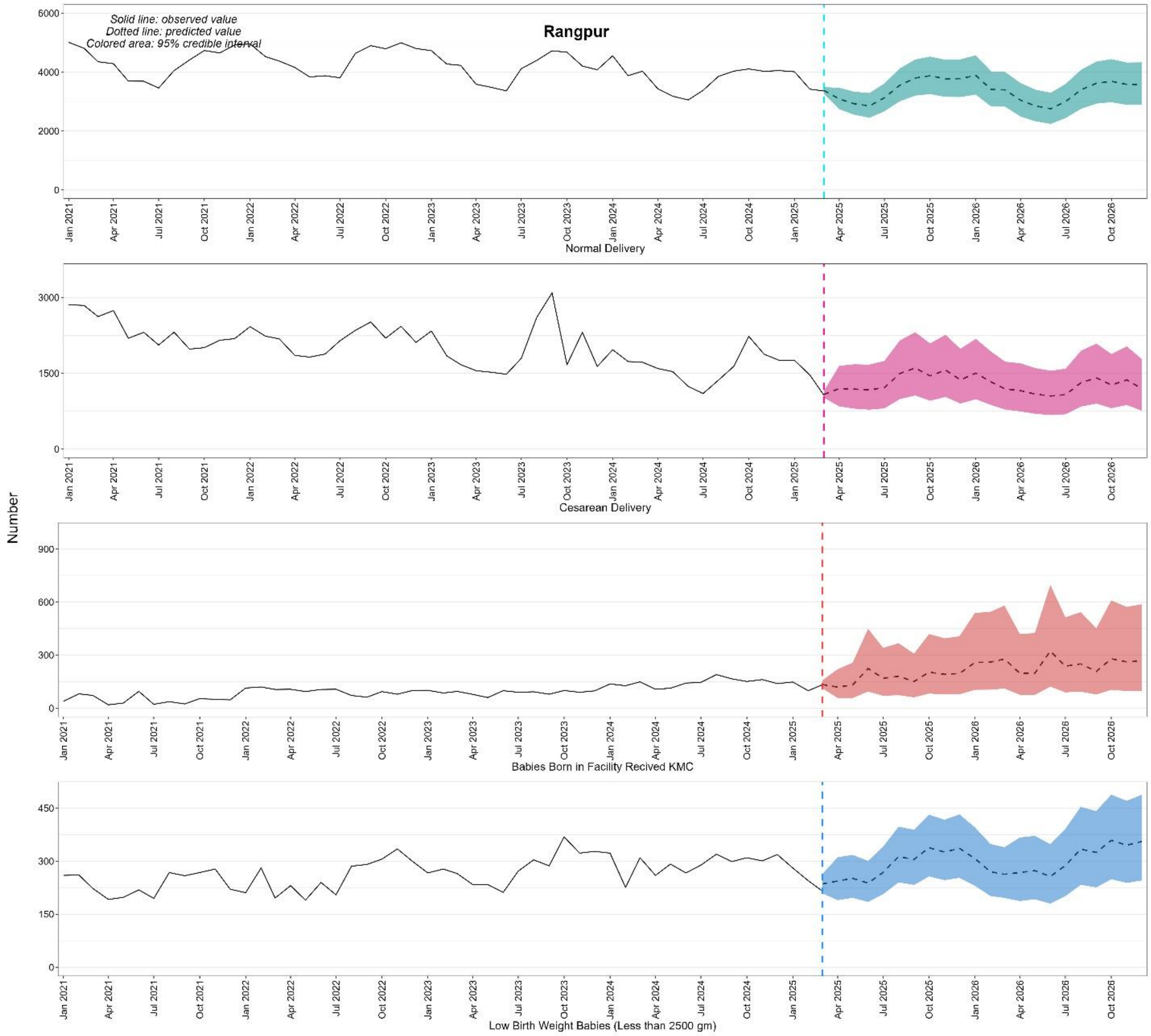
Solid line: observed value
Dotted line: predicted value
Colored area: 95% credible interval
Rangpur
Number
Normal Delivery
Cesarean Delivery
Babies Born in Facility Recived KMC
Low Birth Weight Babies (Less than 2500 gm)

Sylhet

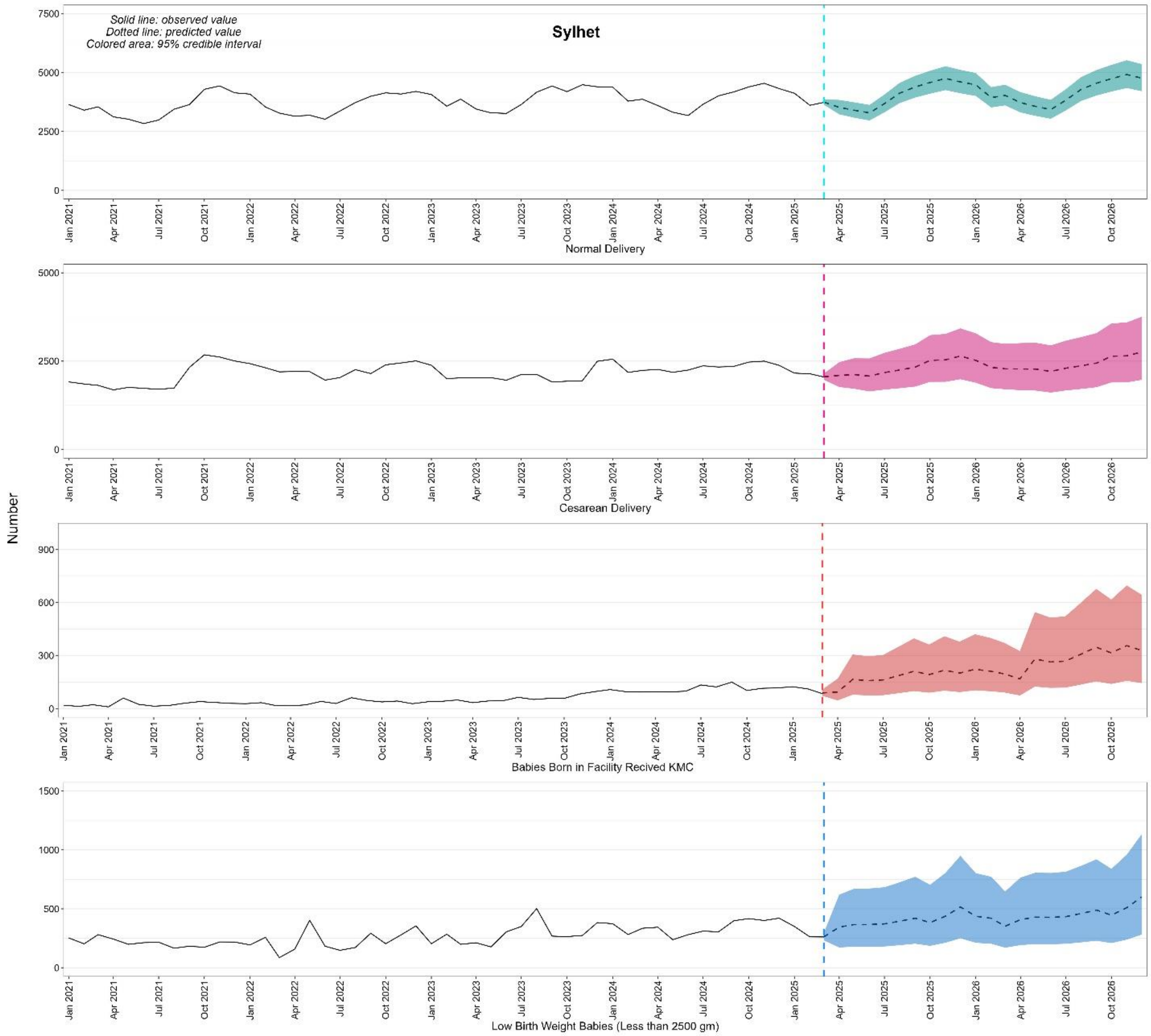
Solid line: observed value
Dotted line: predicted value
Colored area: 95% credible interval
Sylhet
Normal Delivery
Cesarean Delivery
Babies Born in Facility Recived KMC
Low Birth Weight Babies (Less than 2500 gm)
Number
