# Supplementary material for: Using DHIS2 routine data for health system preparedness in resource-limited settings: A Bayesian predictive approach in Bangladesh
Source: PLOS Glob Public Health. 2026 Mar 3;6(3):e0005231. doi: 10.1371/journal.pgph.0005231 (PMC12956080; doi:10.1371/journal.pgph.0005231)
Supplement: S2 File — (PDF) [file pgph.0005231.s002.pdf]

S2 File: Trend and prediction of child health services

Bangladesh

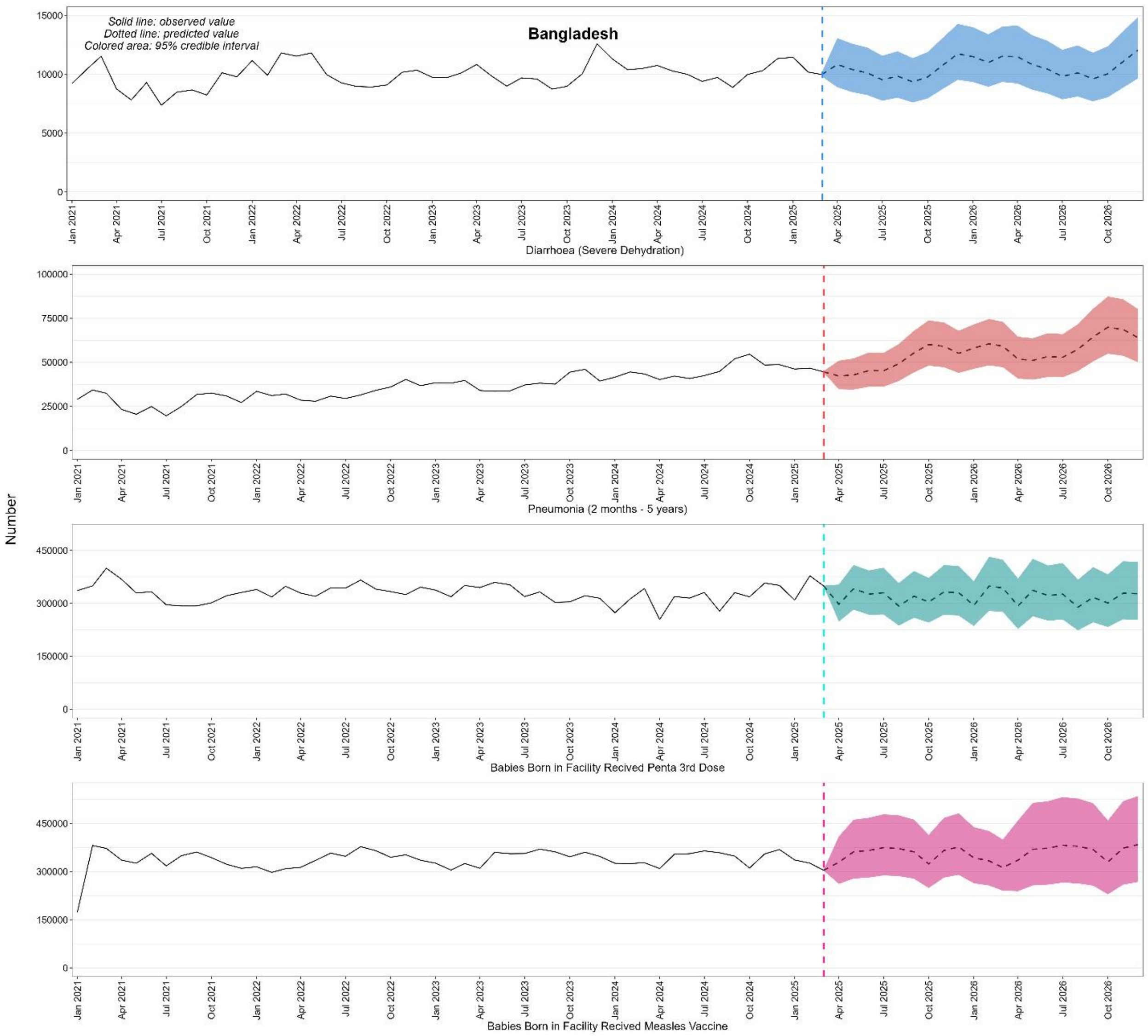

Barisal

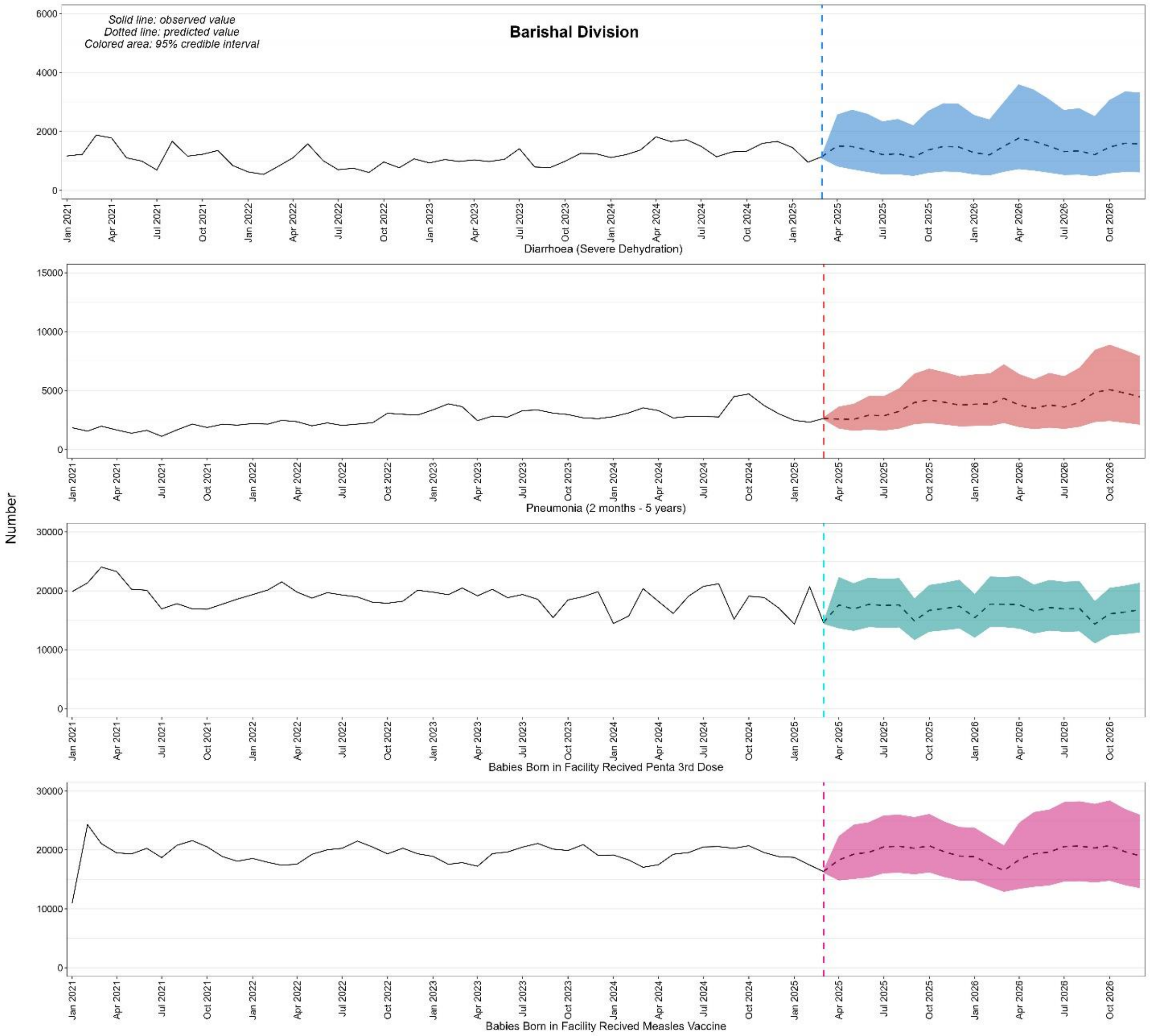
Solid line: observed value
Dotted line: predicted value
Colored area: 95% credible interval
Barishal Division
Diarrhoea (Severe Dehydration)
Pneumonia (2 months - 5 years)
Babies Born in Facility Recived Penta 3rd Dose
Babies Born in Facility Recived Measles Vaccine
Number

Chittagong

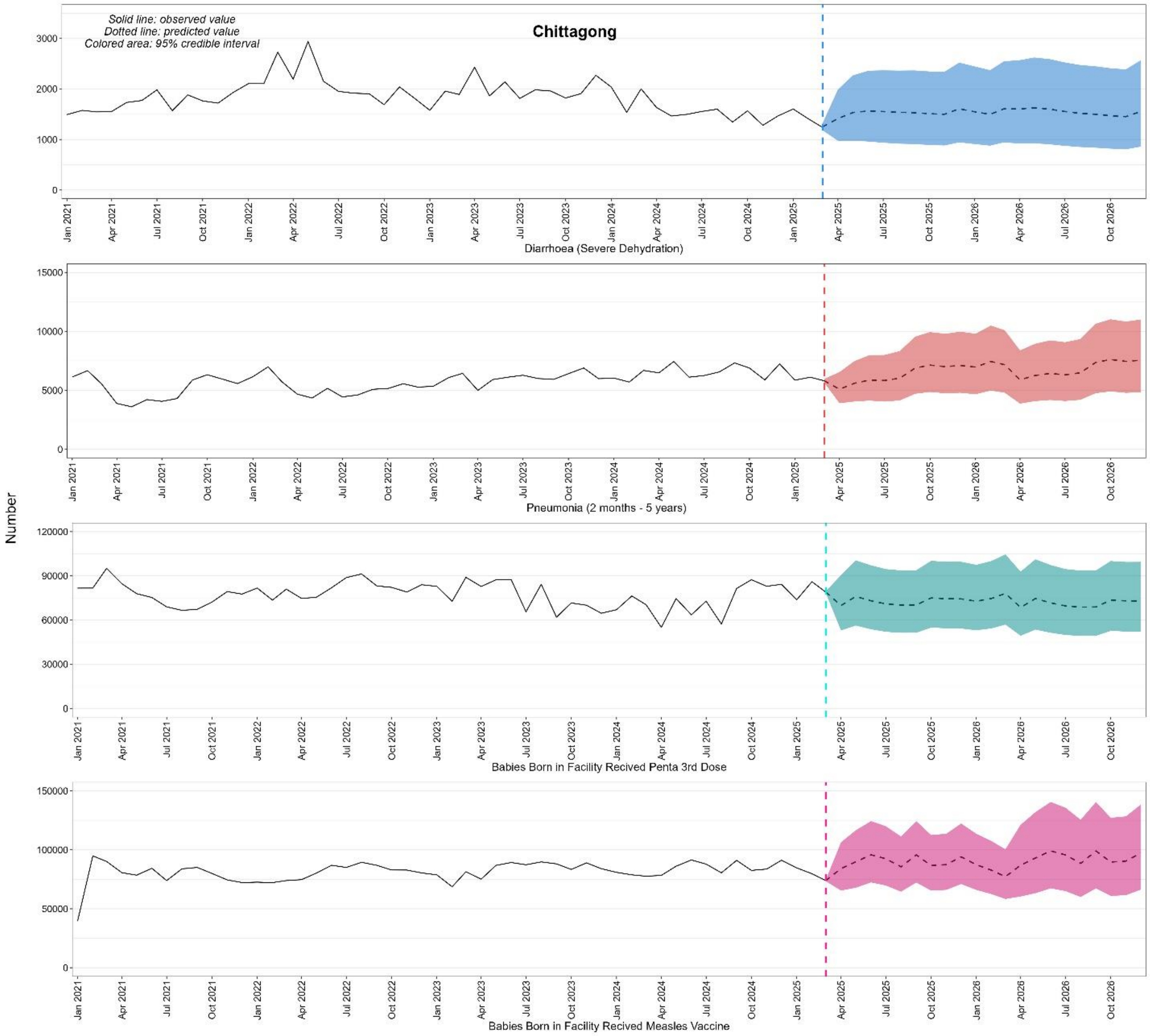

Solid line: observed value
Dotted line: predicted value
Colored area: 95% credible interval
Chittagong
Number
Diarrhoea (Severe Dehydration)
Pneumonia (2 months - 5 years)
Babies Born in Facility Recived Penta 3rd Dose
Babies Born in Facility Recived Measles Vaccine

Dhaka

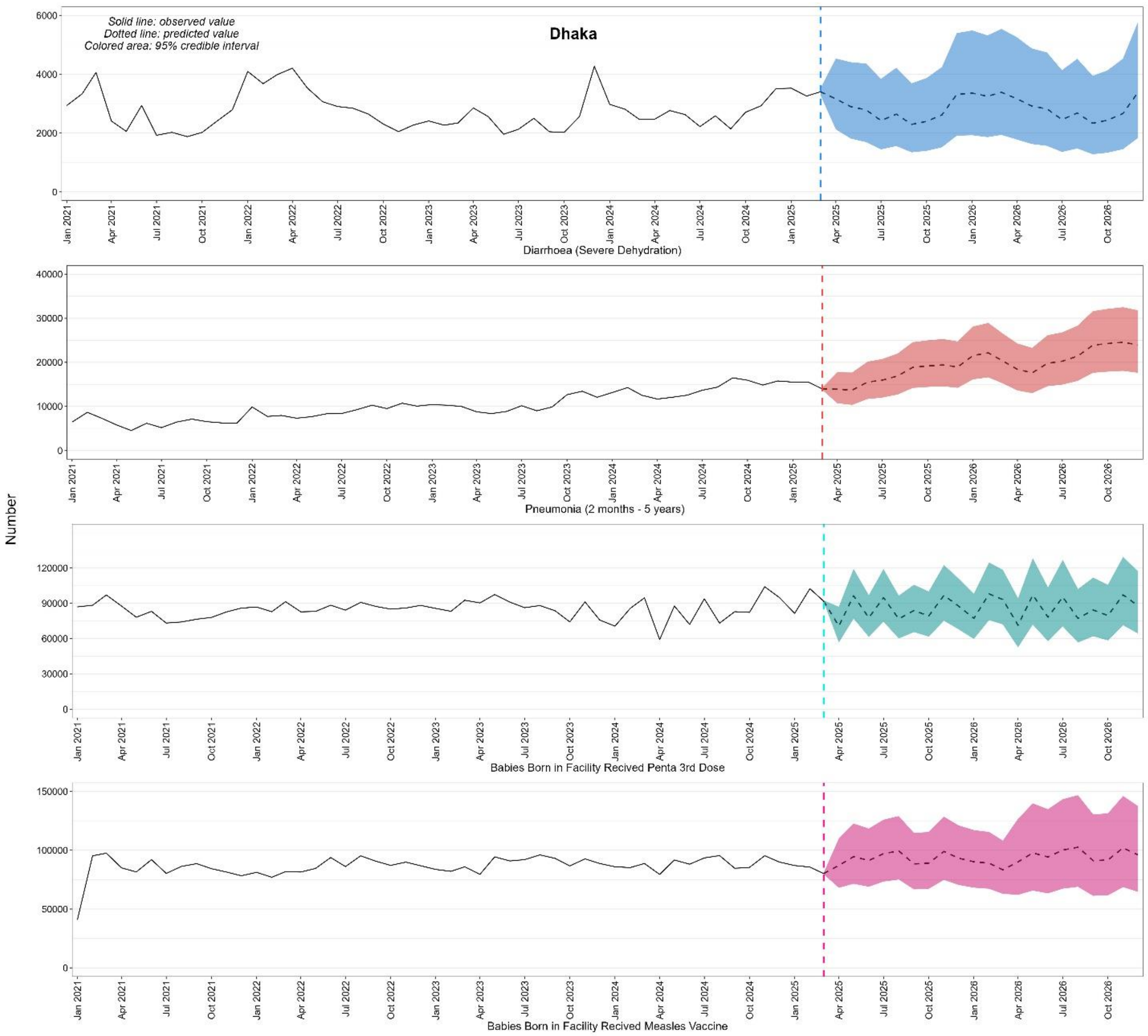
Solid line: observed value
Dotted line: predicted value
Colored area: 95% credible interval
Dhaka
Number
Diarrhoea (Severe Dehydration)
Pneumonia (2 months - 5 years)
Babies Born in Facility Recived Penta 3rd Dose
Babies Born in Facility Recived Measles Vaccine

Khulna

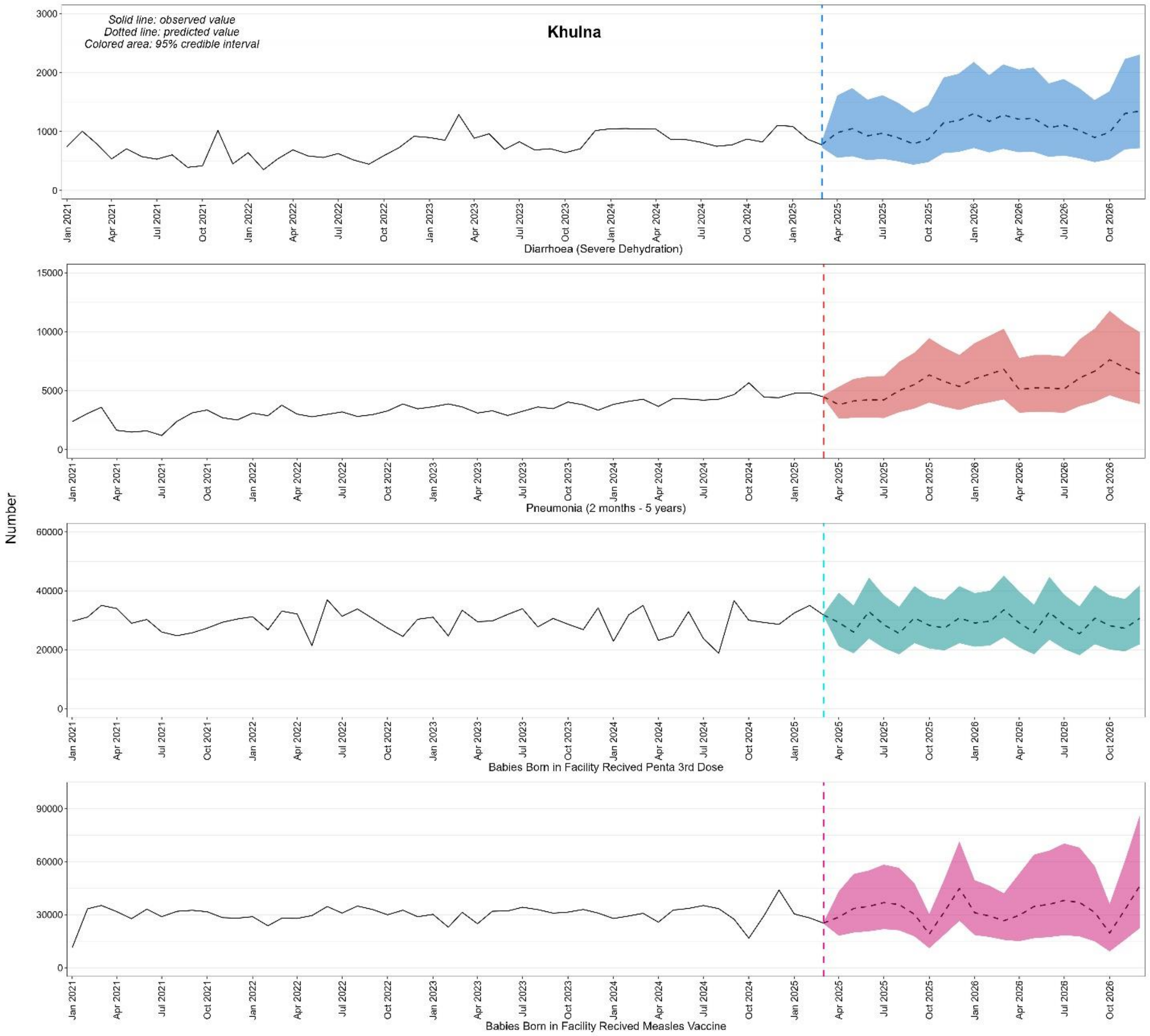

Solid line: observed value
Dotted line: predicted value
Colored area: 95% credible interval
Khulna
Diarrhoea (Severe Dehydration)
Pneumonia (2 months - 5 years)
Babies Born in Facility Recived Penta 3rd Dose
Babies Born in Facility Recived Measles Vaccine
Number

Mymensingh

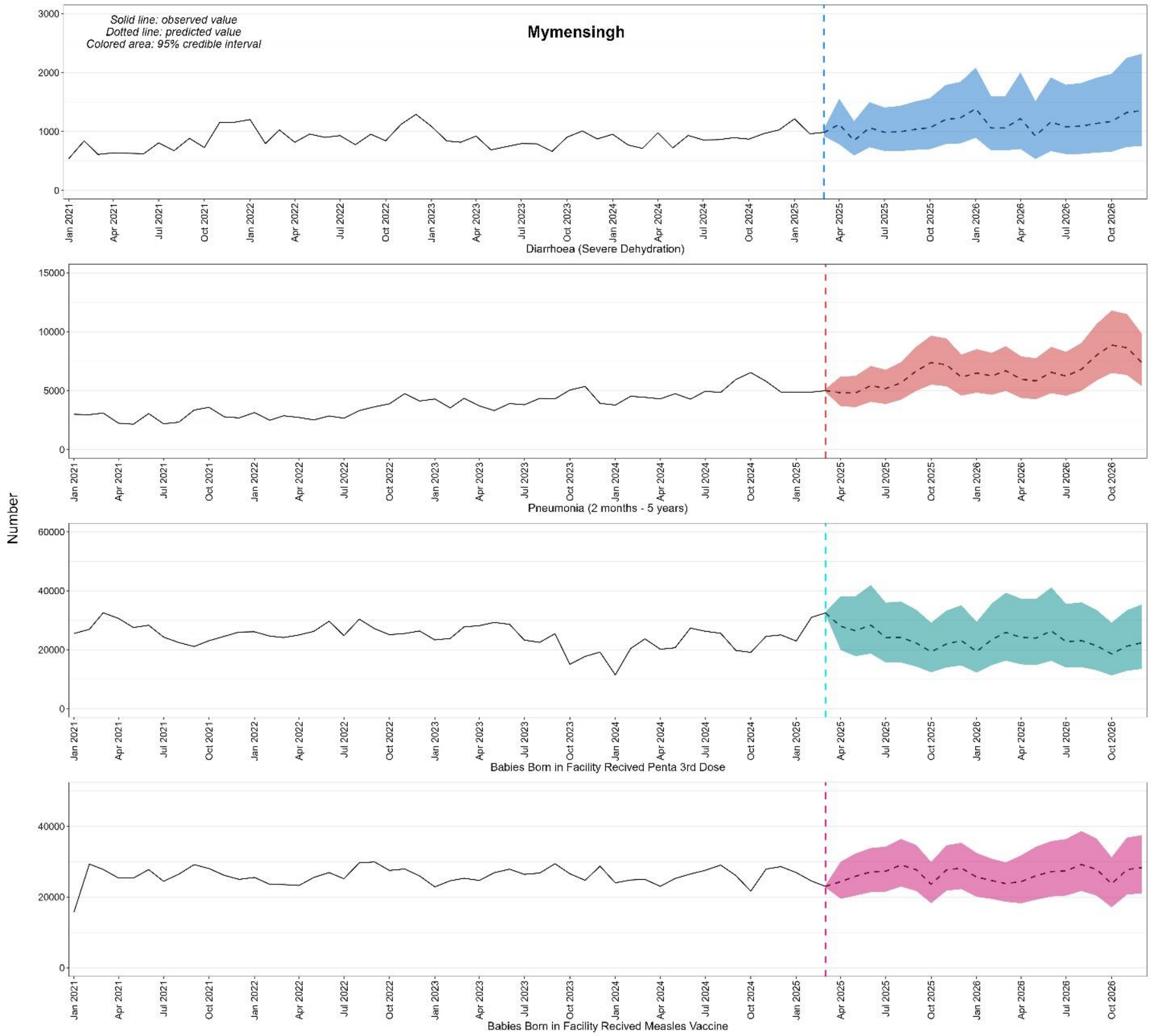

Mymensingh
Solid line: observed value
Dotted line: predicted value
Colored area: 95% credible interval
Number
Diarrhoea (Severe Dehydration)
Pneumonia (2 months - 5 years)
Babies Born in Facility Recived Penta 3rd Dose
Babies Born in Facility Recived Measles Vaccine

Rajshahi

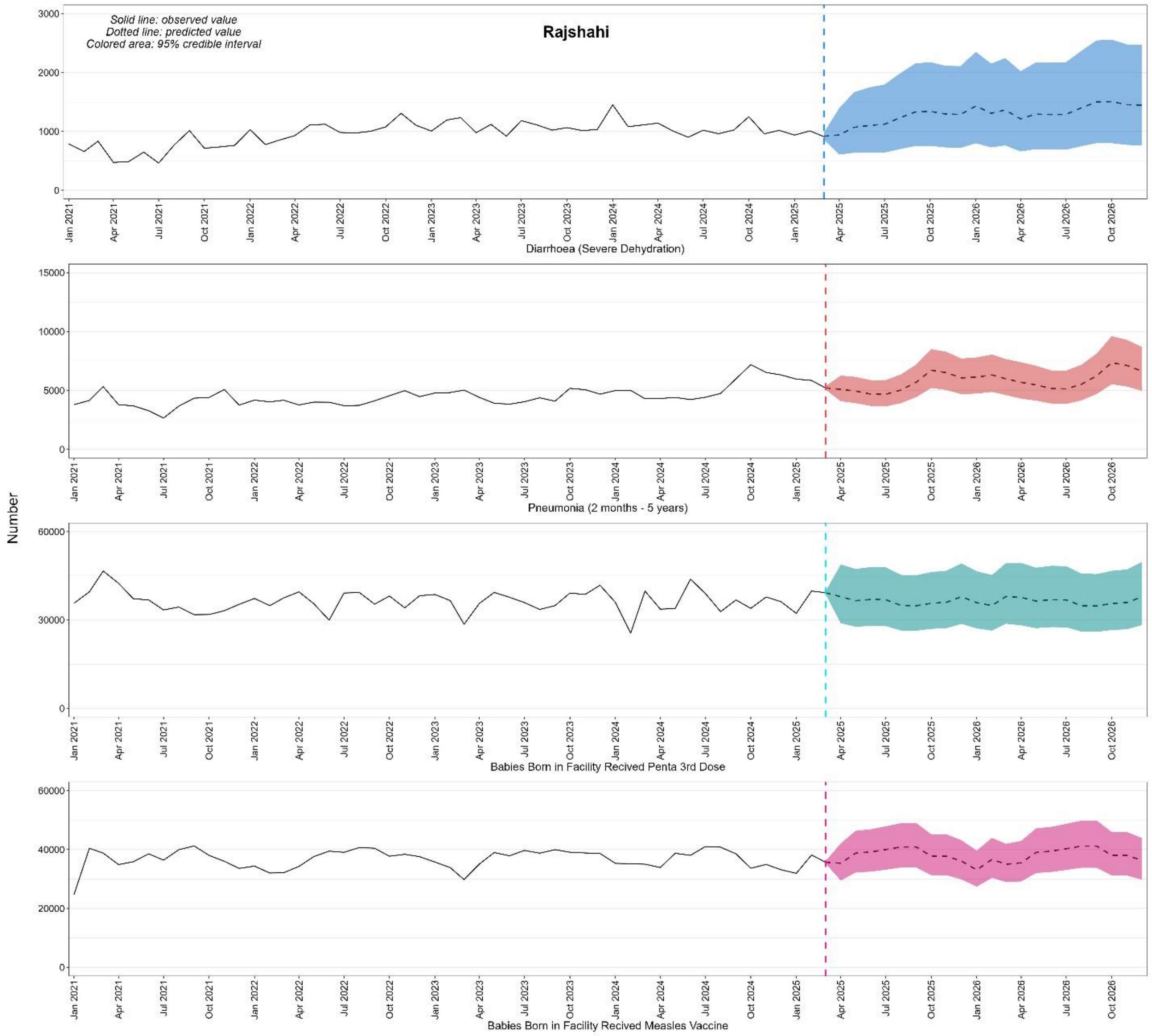
Solid line: observed value
Dotted line: predicted value
Colored area: 95% credible interval
Rajshahi
Number
Diarrhoea (Severe Dehydration)
Pneumonia (2 months - 5 years)
Babies Born in Facility Recived Penta 3rd Dose
Babies Born in Facility Recived Measles Vaccine

Rangpur

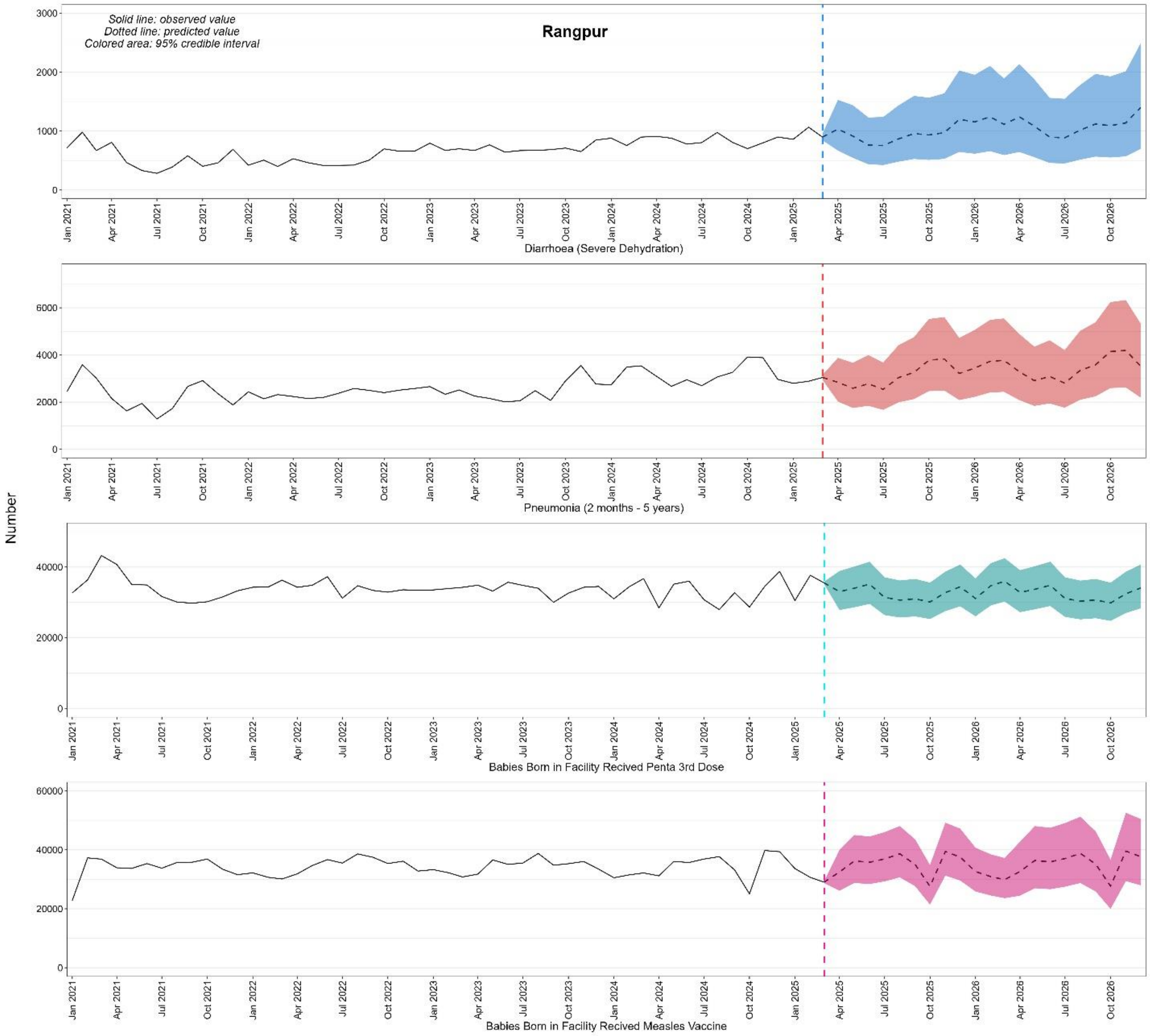
Solid line: observed value
Dotted line: predicted value
Colored area: 95% credible interval
Rangpur
Number
Diarrhoea (Severe Dehydration)
Pneumonia (2 months - 5 years)
Babies Born in Facility Recived Penta 3rd Dose
Babies Born in Facility Recived Measles Vaccine

Sylhet

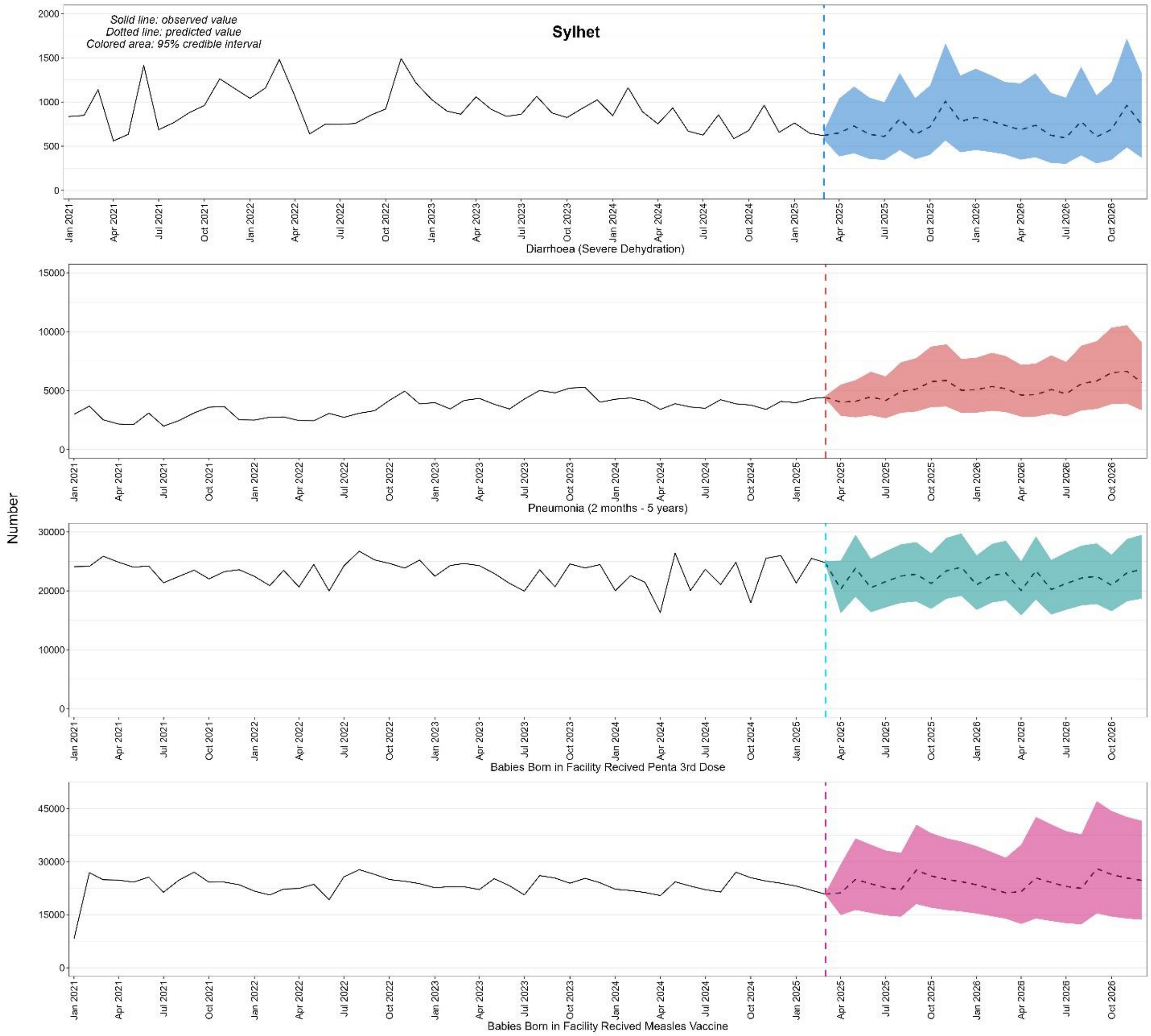

Solid line: observed value
Dotted line: predicted value
Colored area: 95% credible interval
Sylhet
Diarrhoea (Severe Dehydration)
Pneumonia (2 months - 5 years)
Babies Born in Facility Recived Penta 3rd Dose
Babies Born in Facility Recived Measles Vaccine
Number
