# Supplementary material for: Using DHIS2 routine data for health system preparedness in resource-limited settings: A Bayesian predictive approach in Bangladesh
Source: PLOS Glob Public Health. 2026 Mar 3;6(3):e0005231. doi: 10.1371/journal.pgph.0005231 (PMC12956080; doi:10.1371/journal.pgph.0005231)
Supplement: S3 File — (PDF) [file pgph.0005231.s003.pdf]

S3 File: Trend and prediction of hospital-based services

Bangladesh

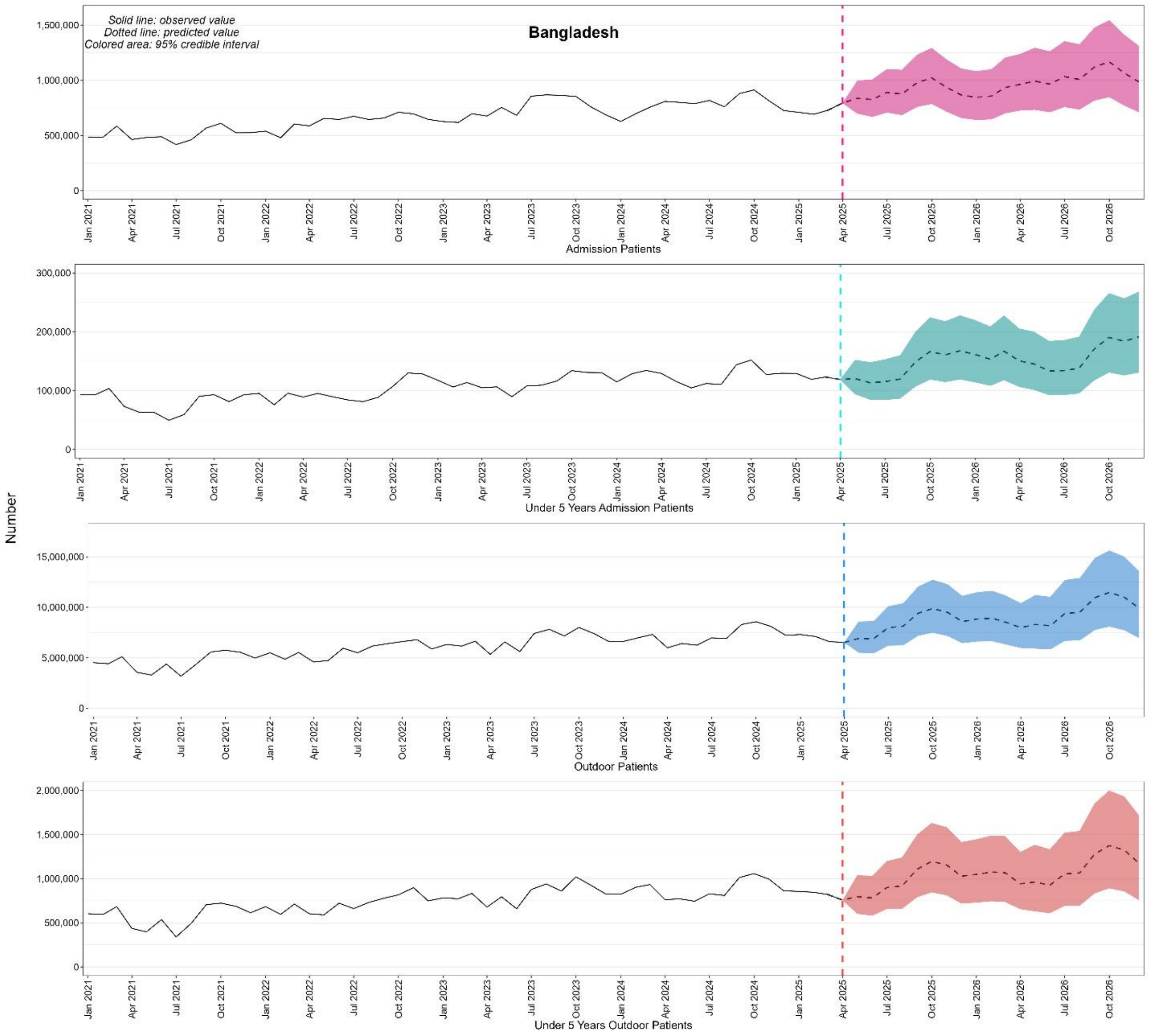

Barisal

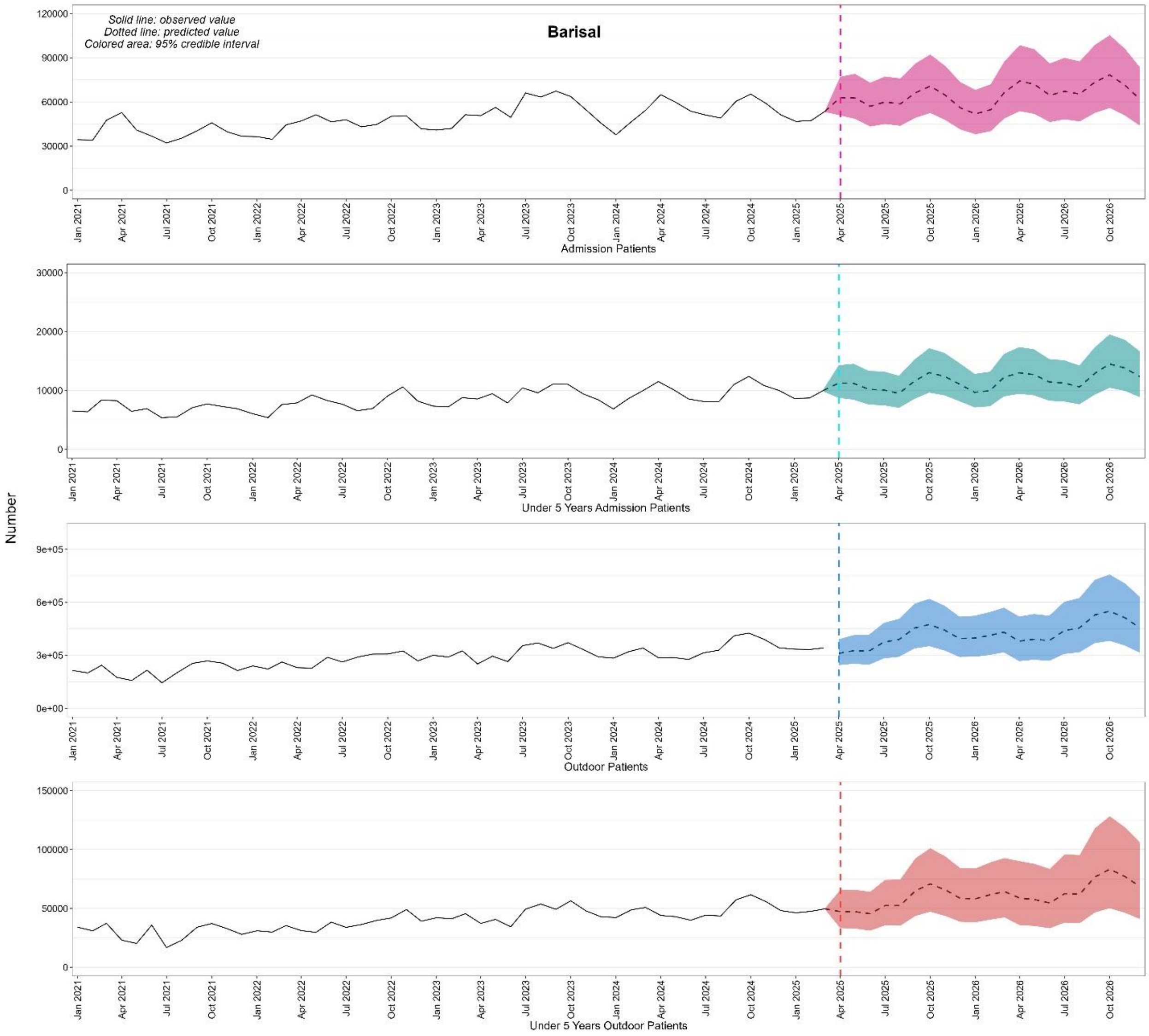
Barisal
Solid line: observed value
Dotted line: predicted value
Colored area: 95% credible interval
Number
Admission Patients
Under 5 Years Admission Patients
Outdoor Patients
Under 5 Years Outdoor Patients

Chittagong

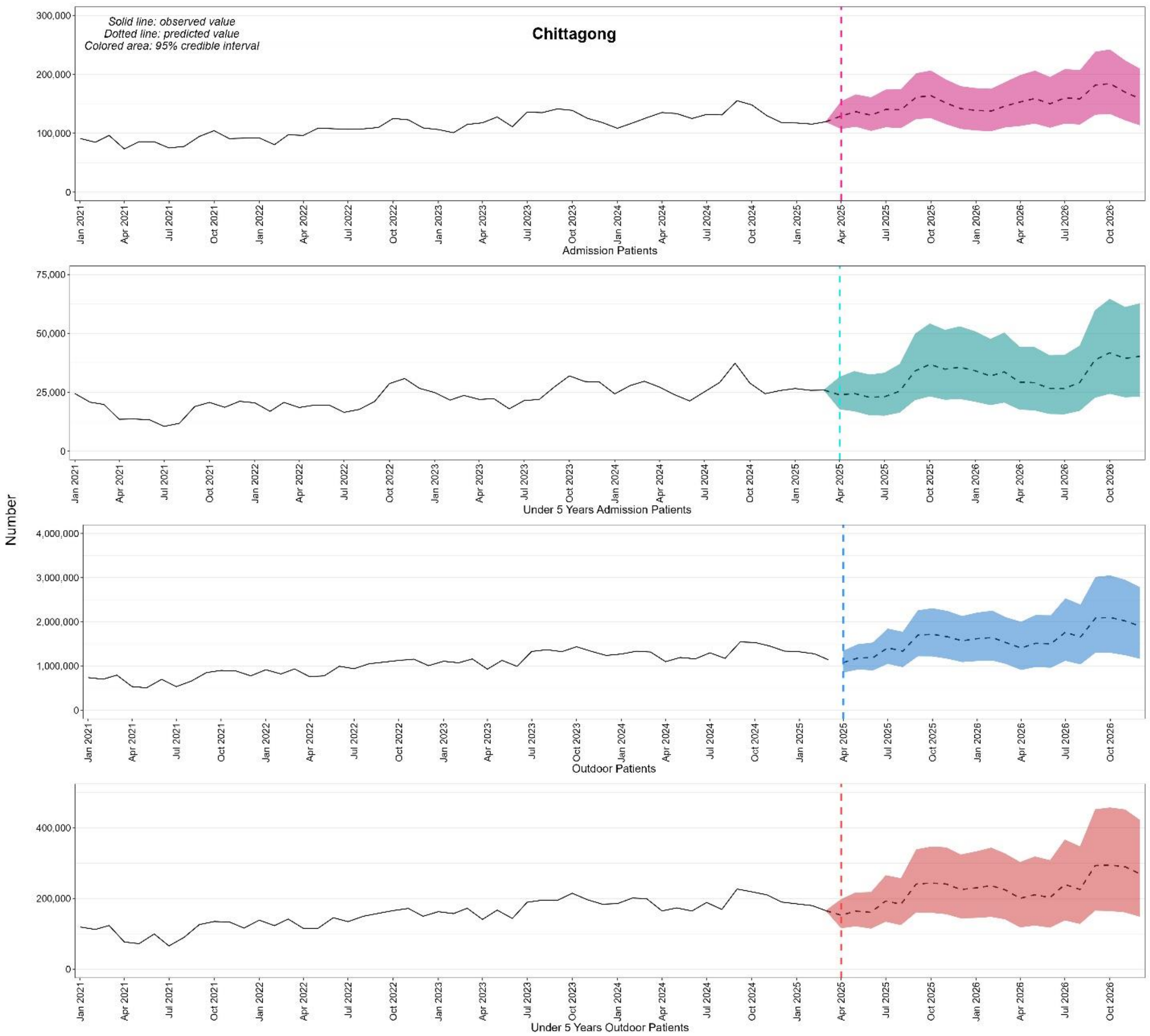
Solid line: observed value
Dotted line: predicted value
Colored area: 95% credible interval
Chittagong
Admission Patients
Under 5 Years Admission Patients
Outdoor Patients
Under 5 Years Outdoor Patients
Number

Dhaka

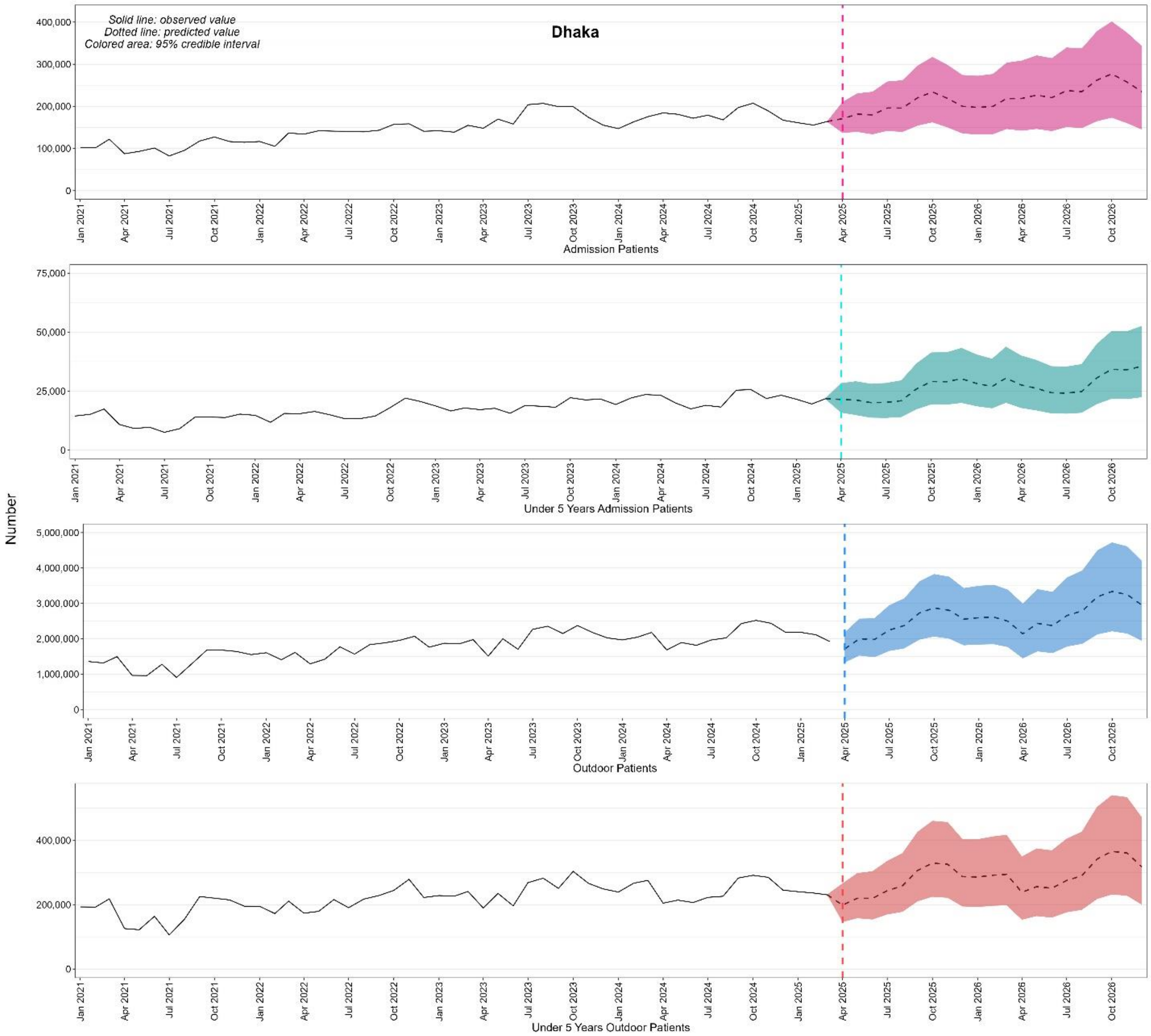
Solid line: observed value
Dotted line: predicted value
Colored area: 95% credible interval
Dhaka
Admission Patients
Under 5 Years Admission Patients
Number
Outdoor Patients
Under 5 Years Outdoor Patients

Khulna

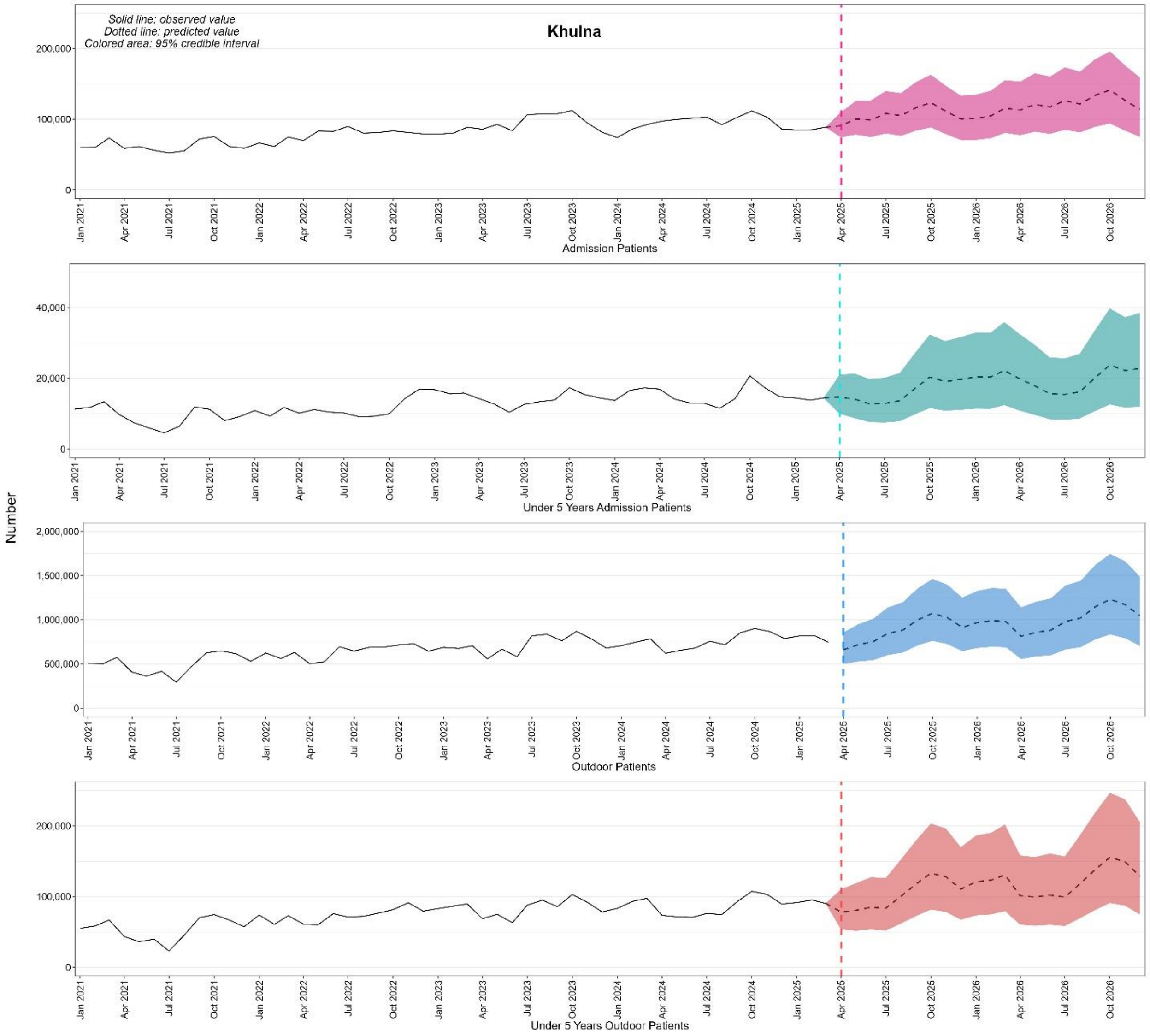

Solid line: observed value
Dotted line: predicted value
Colored area: 95% credible interval
Khulna
Admission Patients
Under 5 Years Admission Patients
Number
Outdoor Patients
Under 5 Years Outdoor Patients

Mymensingh

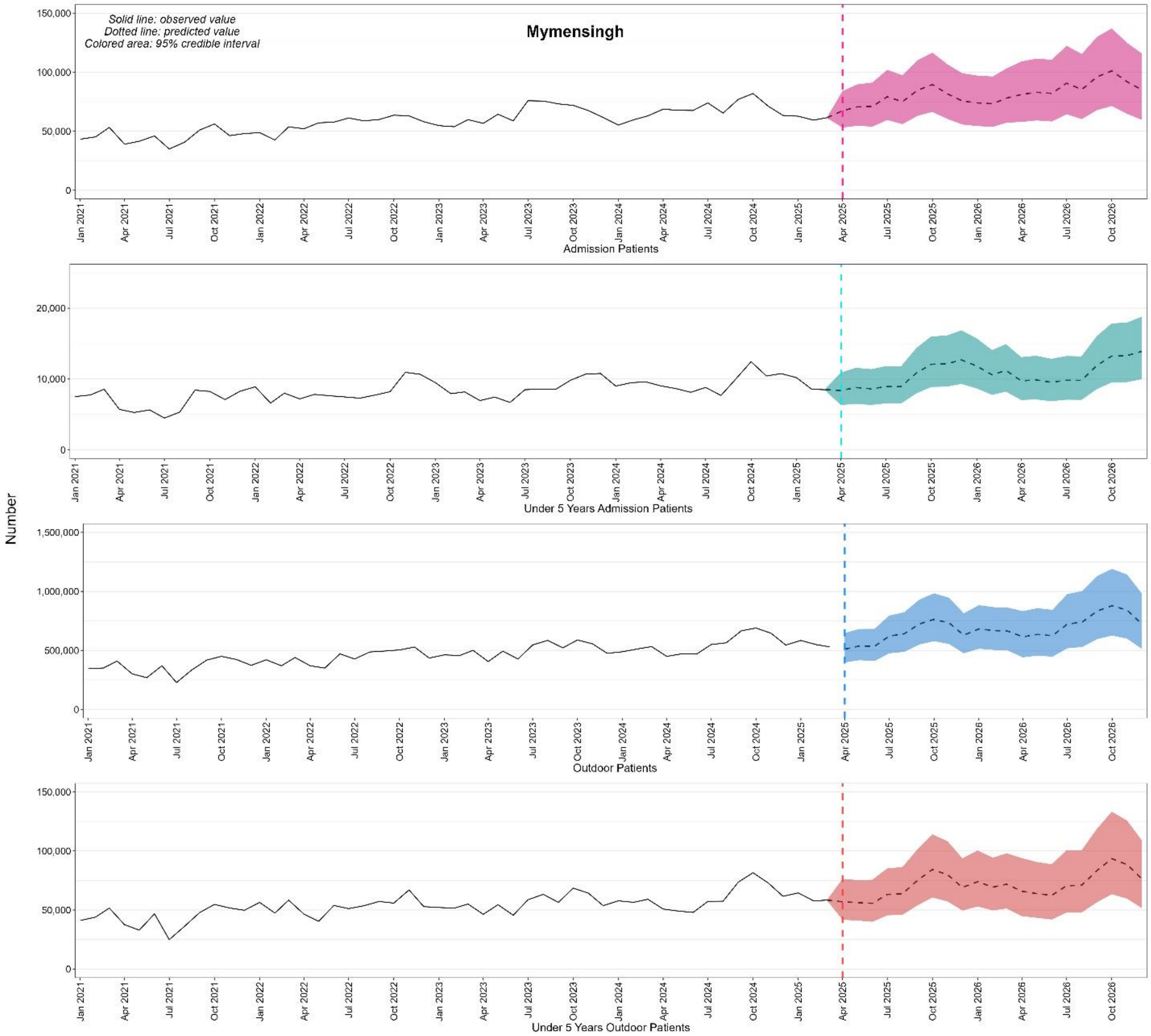
Solid line: observed value
Dotted line: predicted value
Colored area: 95% credible interval
Mymensingh
Admission Patients
Under 5 Years Admission Patients
Number
Outdoor Patients
Under 5 Years Outdoor Patients

Rajshahi

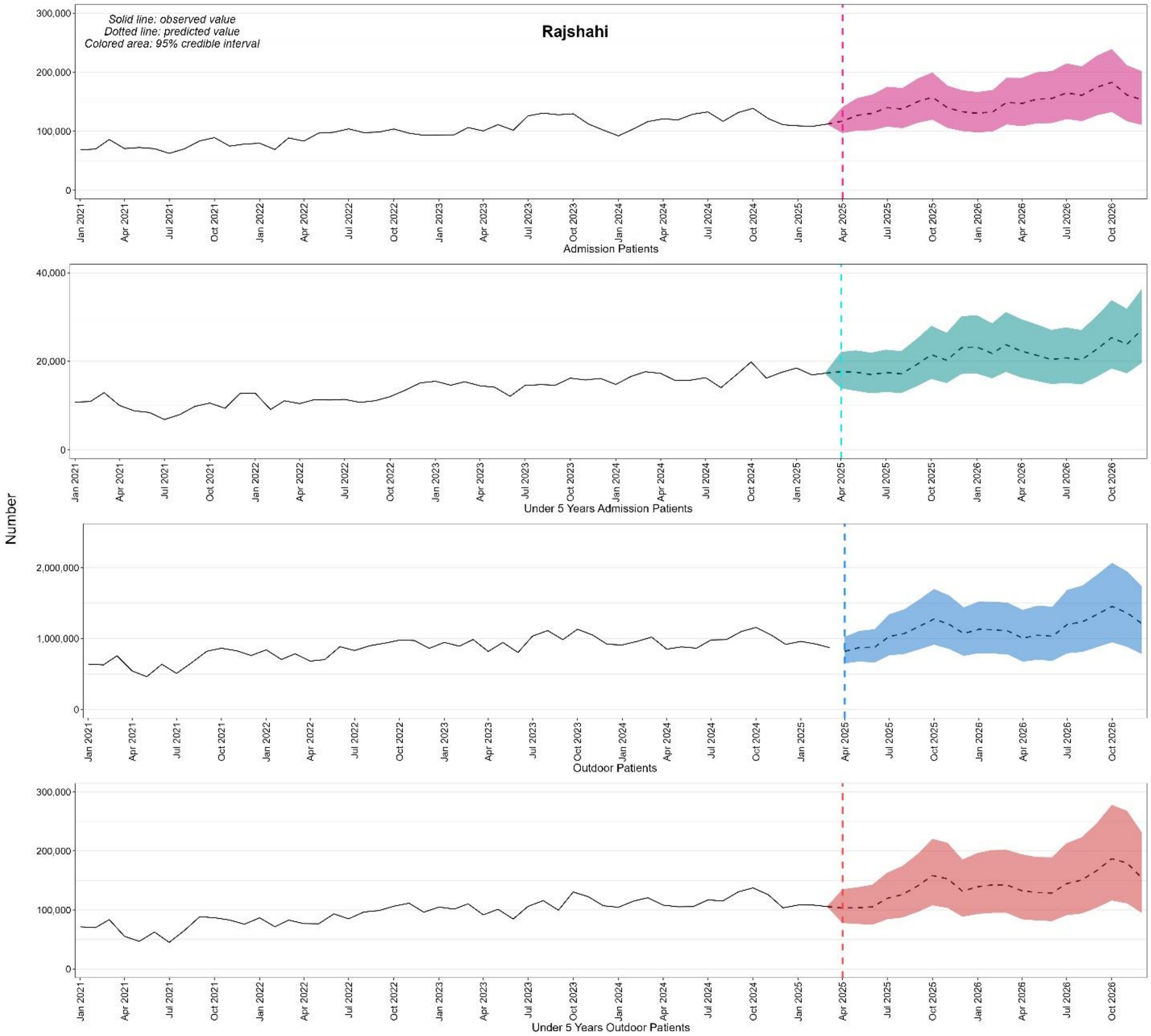
Solid line: observed value
Dotted line: predicted value
Colored area: 95% credible interval
Rajshahi
Number
Admission Patients
Under 5 Years Admission Patients
Outdoor Patients
Under 5 Years Outdoor Patients

Rangpur

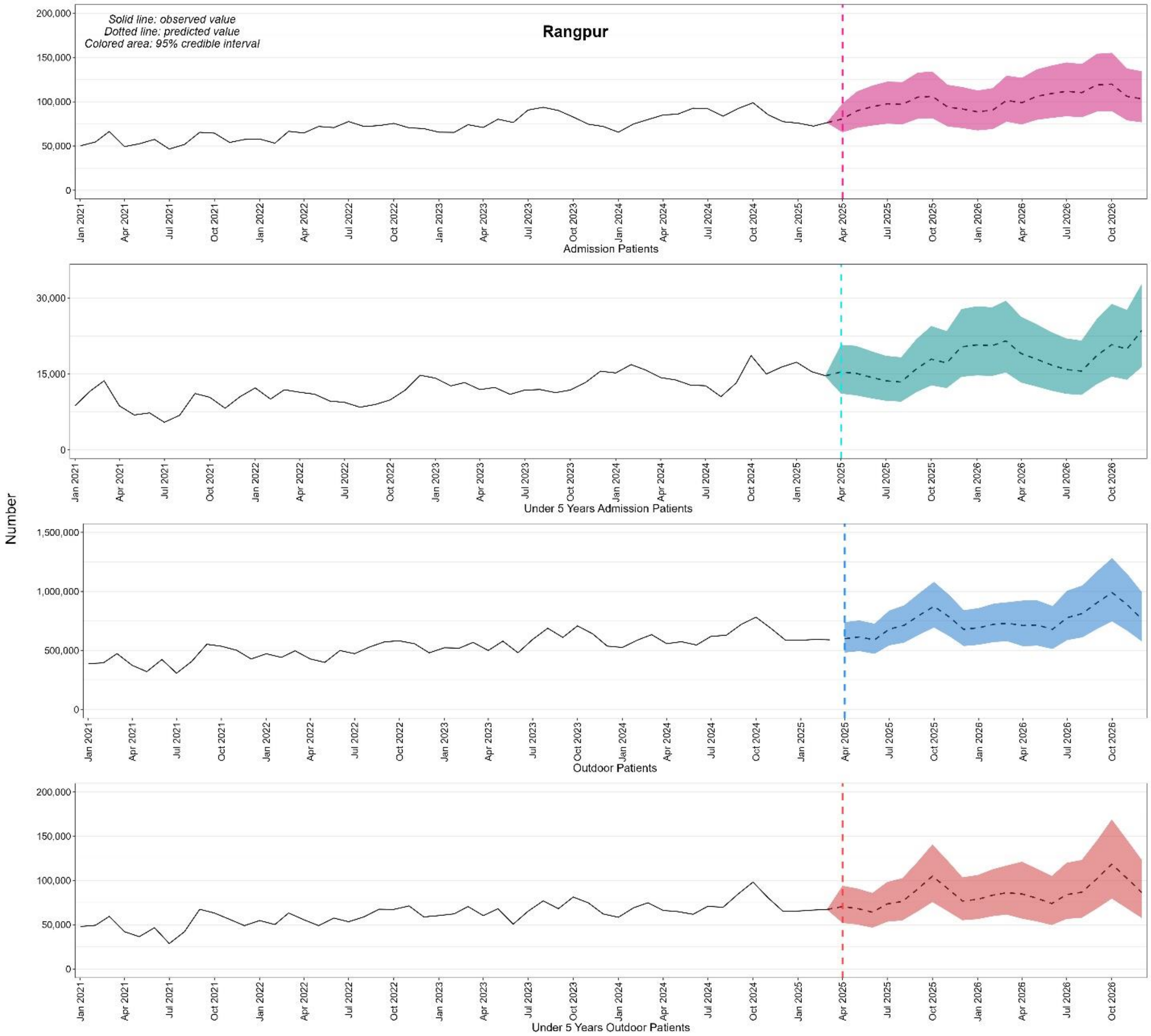
Solid line: observed value
Dotted line: predicted value
Colored area: 95% credible interval
Rangpur
Number
Admission Patients
Under 5 Years Admission Patients
Outdoor Patients
Under 5 Years Outdoor Patients

Sylhet

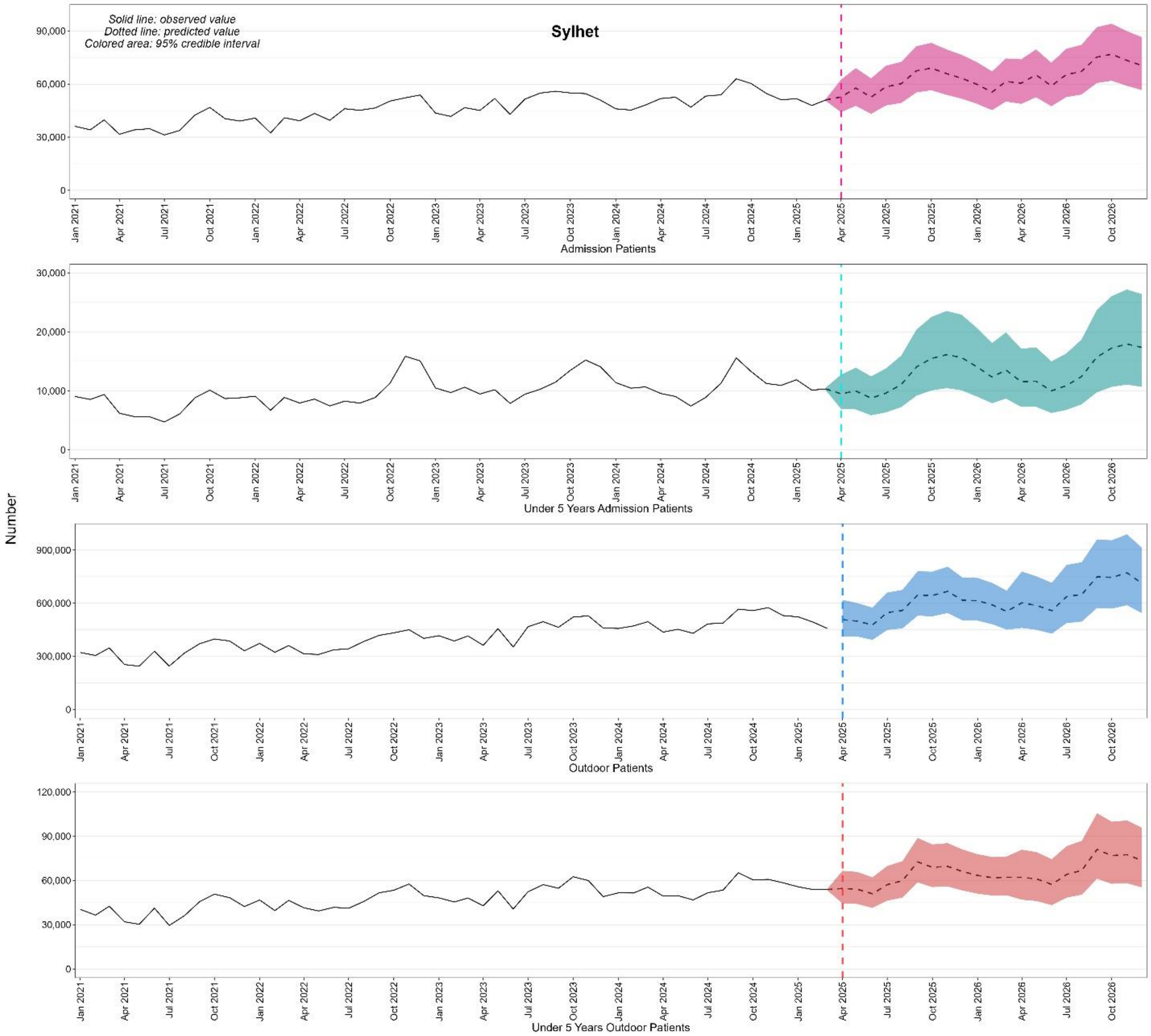
Solid line: observed value
Dotted line: predicted value
Colored area: 95% credible interval
Sylhet
Admission Patients
Under 5 Years Admission Patients
Outdoor Patients
Under 5 Years Outdoor Patients
Number
