# Supplementary material for: Using DHIS2 routine data for health system preparedness in resource-limited settings: A Bayesian predictive approach in Bangladesh
Source: PLOS Glob Public Health. 2026 Mar 3;6(3):e0005231. doi: 10.1371/journal.pgph.0005231 (PMC12956080; doi:10.1371/journal.pgph.0005231)
Supplement: S4 File — (PDF) [file pgph.0005231.s004.pdf]

S4_file: Posterior predictive check comparing observed data with 95% predictive credible intervals

Bangladesh

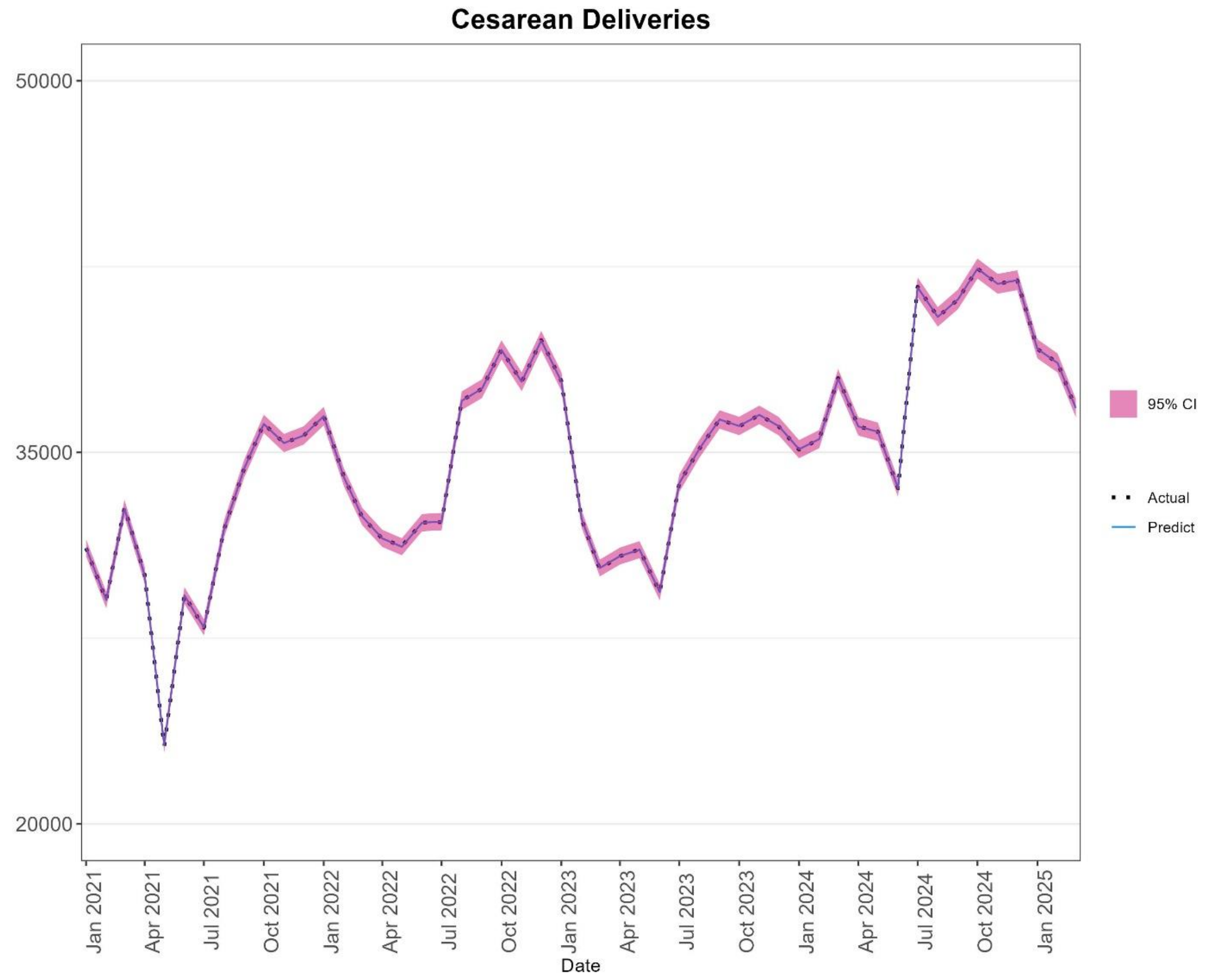

Normal Vaginal Deliveries

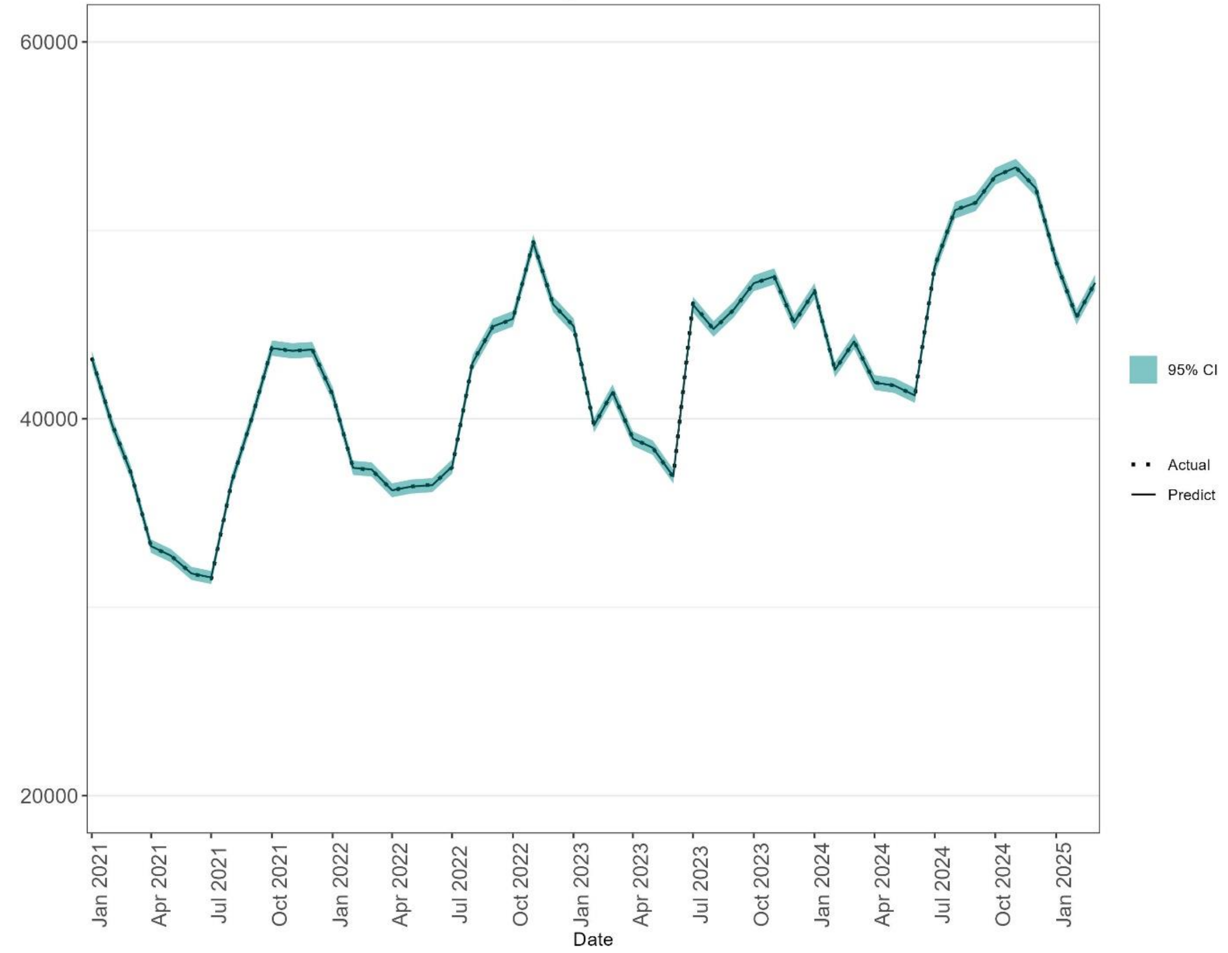

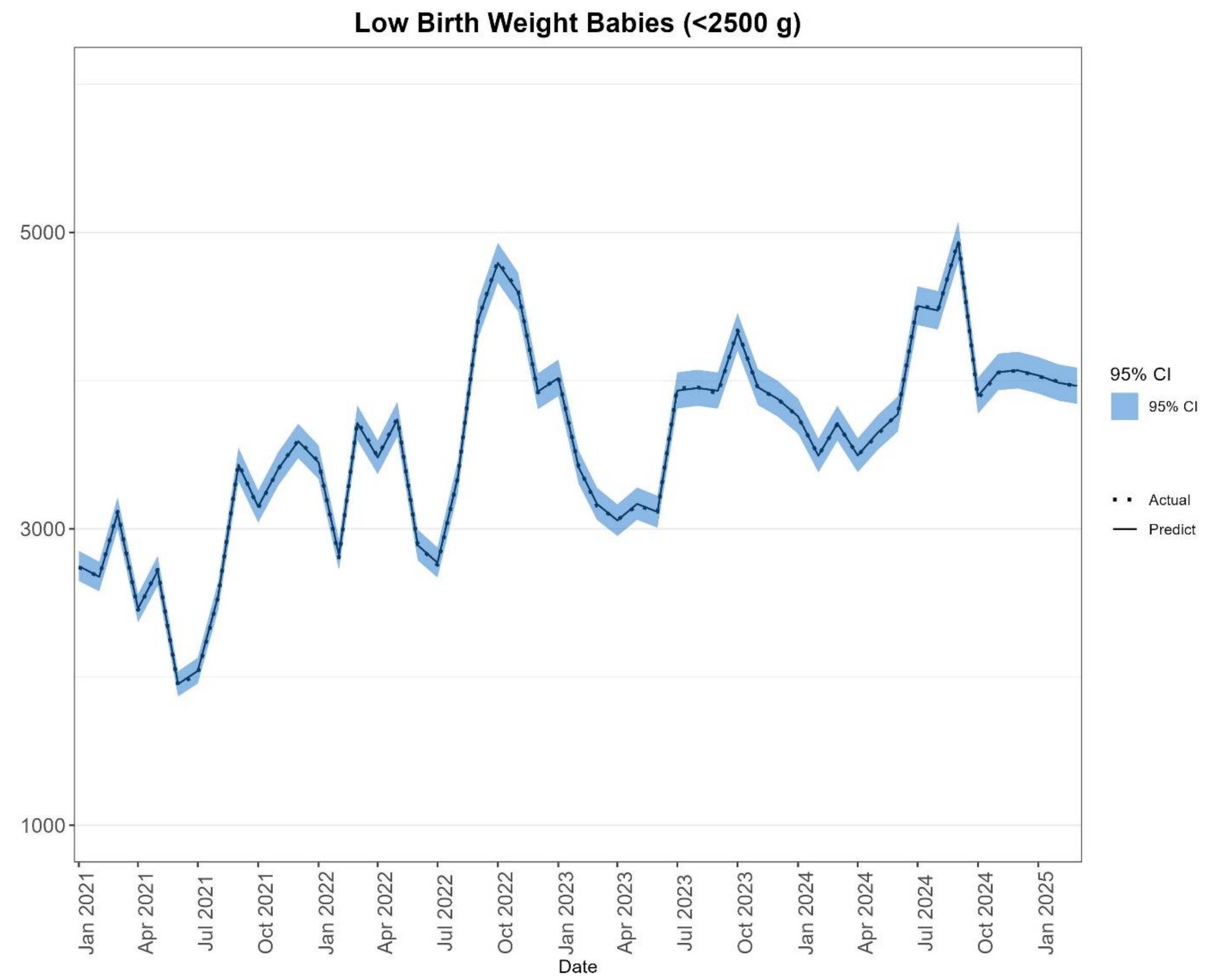

Low Birth Weight Babies (<2500 g)
5000
3000
1000
Jan 2021
Apr 2021
Jul 2021
Oct 2021
Jan 2022
Apr 2022
Jul 2022
Oct 2022
Jan 2023
Apr 2023
Jul 2023
Oct 2023
Jan 2024
Apr 2024
Jul 2024
Oct 2024
Jan 2025
Date
95% CI
95% CI
Actual
Predict

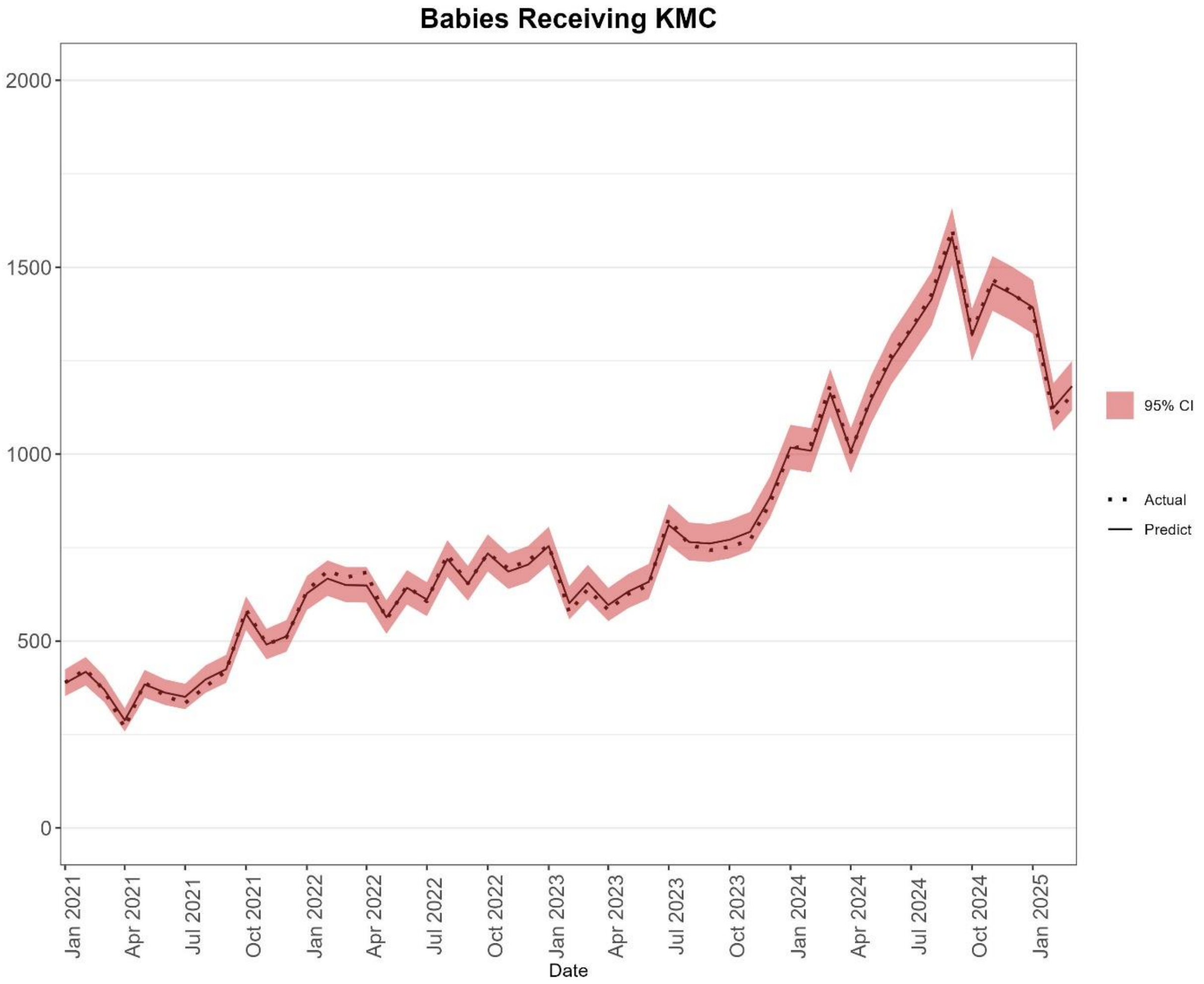
Babies Receiving KMC
2000
1500
1000
500
0
Jan 2021
Apr 2021
Jul 2021
Oct 2021
Jan 2022
Apr 2022
Jul 2022
Oct 2022
Jan 2023
Apr 2023
Jul 2023
Oct 2023
Jan 2024
Apr 2024
Jul 2024
Oct 2024
Jan 2025
Date
95% CI
Actual
Predict

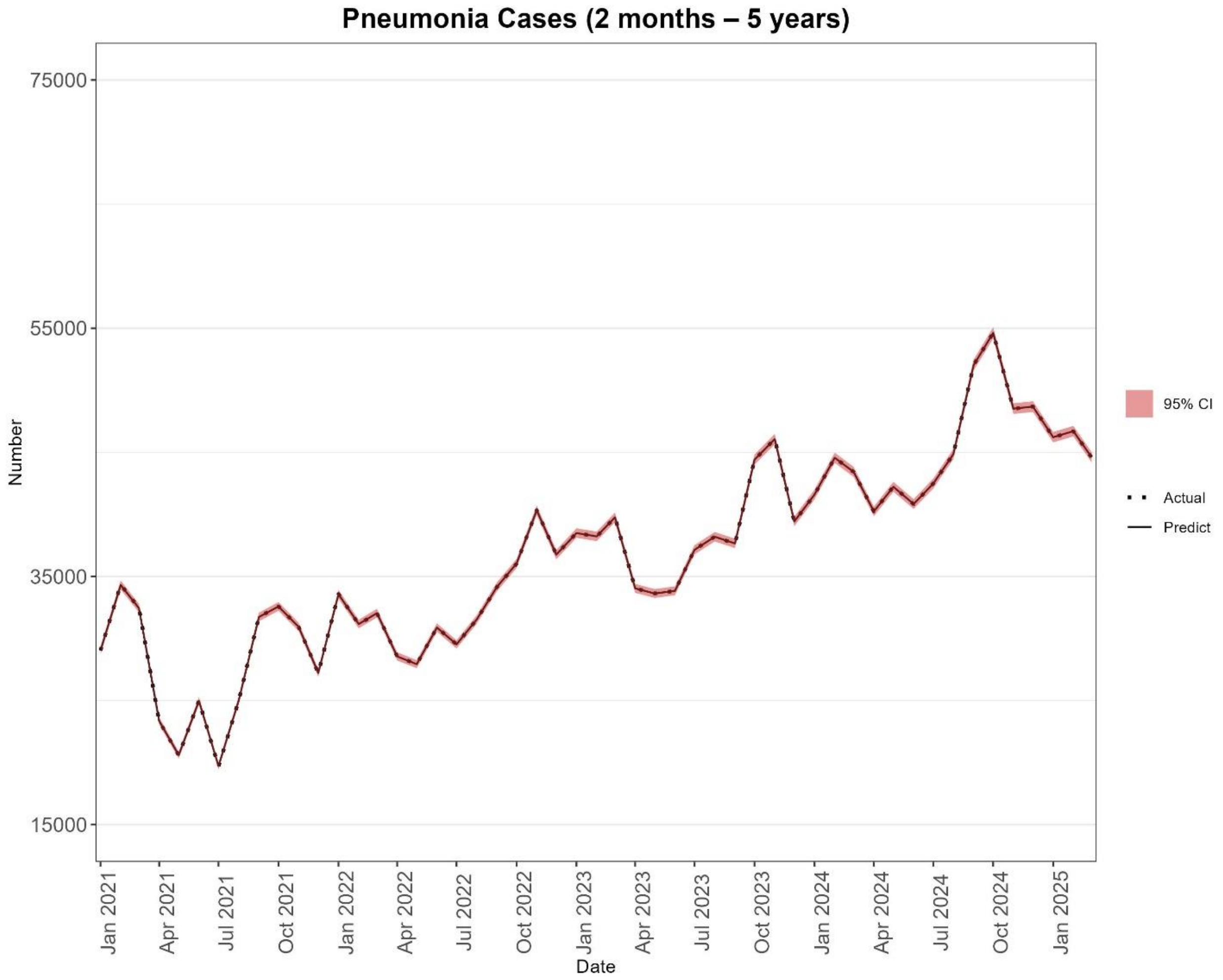
Pneumonia Cases (2 months – 5 years)
75000
55000
35000
15000
Number
Jan 2021
Apr 2021
Jul 2021
Oct 2021
Jan 2022
Apr 2022
Jul 2022
Oct 2022
Jan 2023
Apr 2023
Jul 2023
Oct 2023
Jan 2024
Apr 2024
Jul 2024
Oct 2024
Jan 2025
Date
95% CI
Actual
Predict

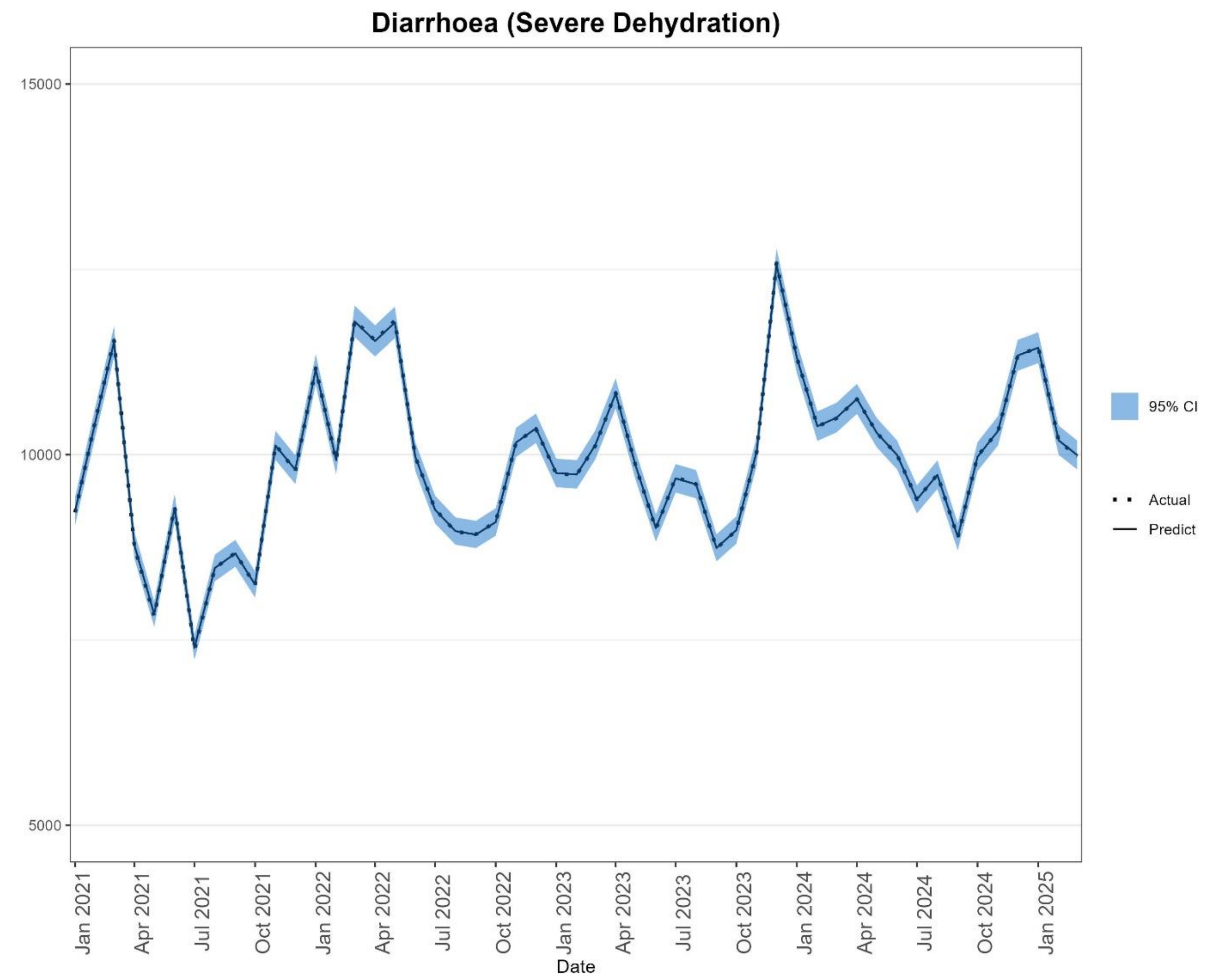

Diarrhoea (Severe Dehydration)
15000
10000
5000
Jan 2021
Apr 2021
Jul 2021
Oct 2021
Jan 2022
Apr 2022
Jul 2022
Oct 2022
Jan 2023
Apr 2023
Jul 2023
Oct 2023
Jan 2024
Apr 2024
Jul 2024
Oct 2024
Jan 2025
Date
95% CI
Actual
Predict

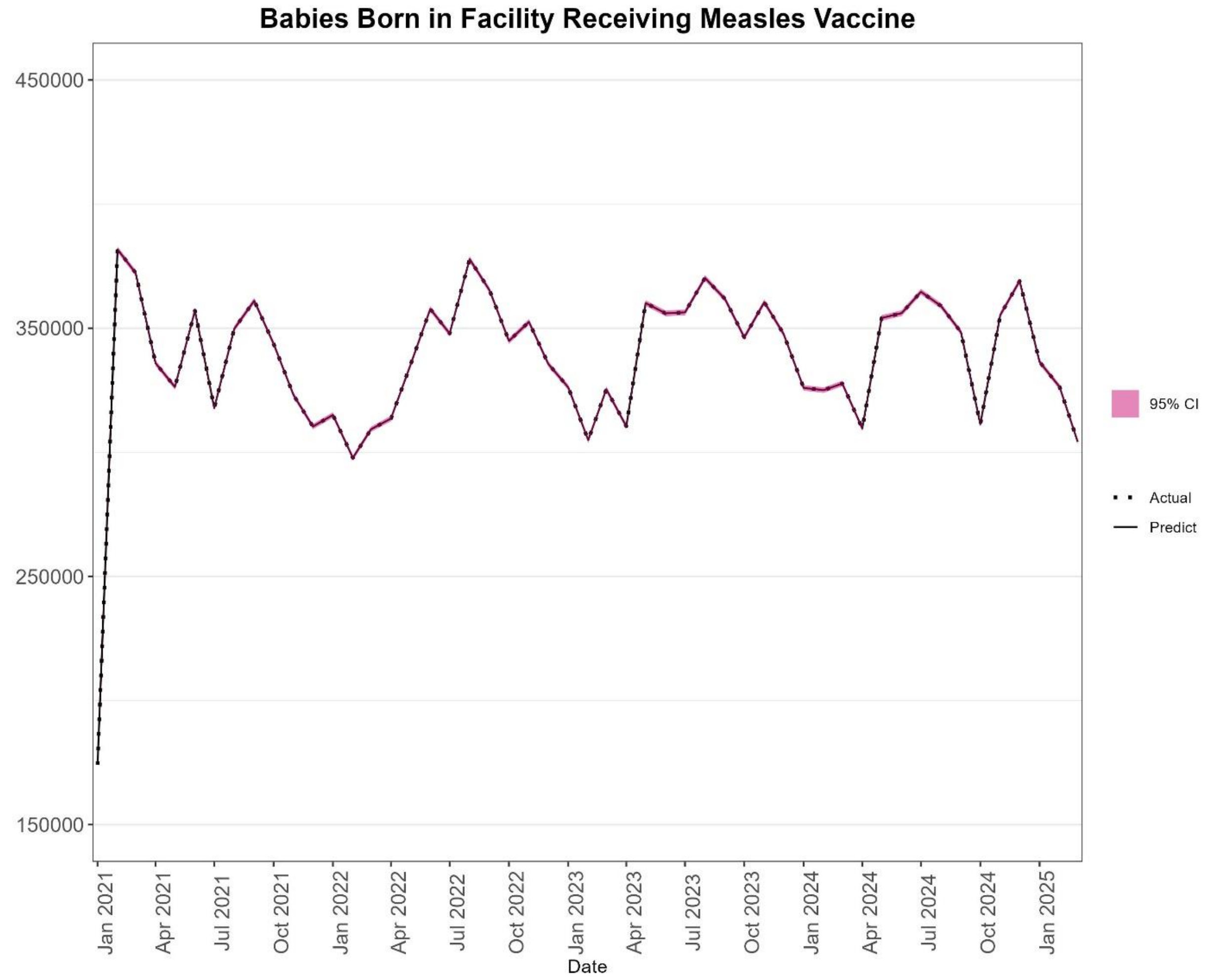
Babies Born in Facility Receiving Measles Vaccine
450000
350000
250000
150000
Jan 2021
Apr 2021
Jul 2021
Oct 2021
Jan 2022
Apr 2022
Jul 2022
Oct 2022
Jan 2023
Apr 2023
Jul 2023
Oct 2023
Jan 2024
Apr 2024
Jul 2024
Oct 2024
Jan 2025
Date
95% CI
Actual
Predict

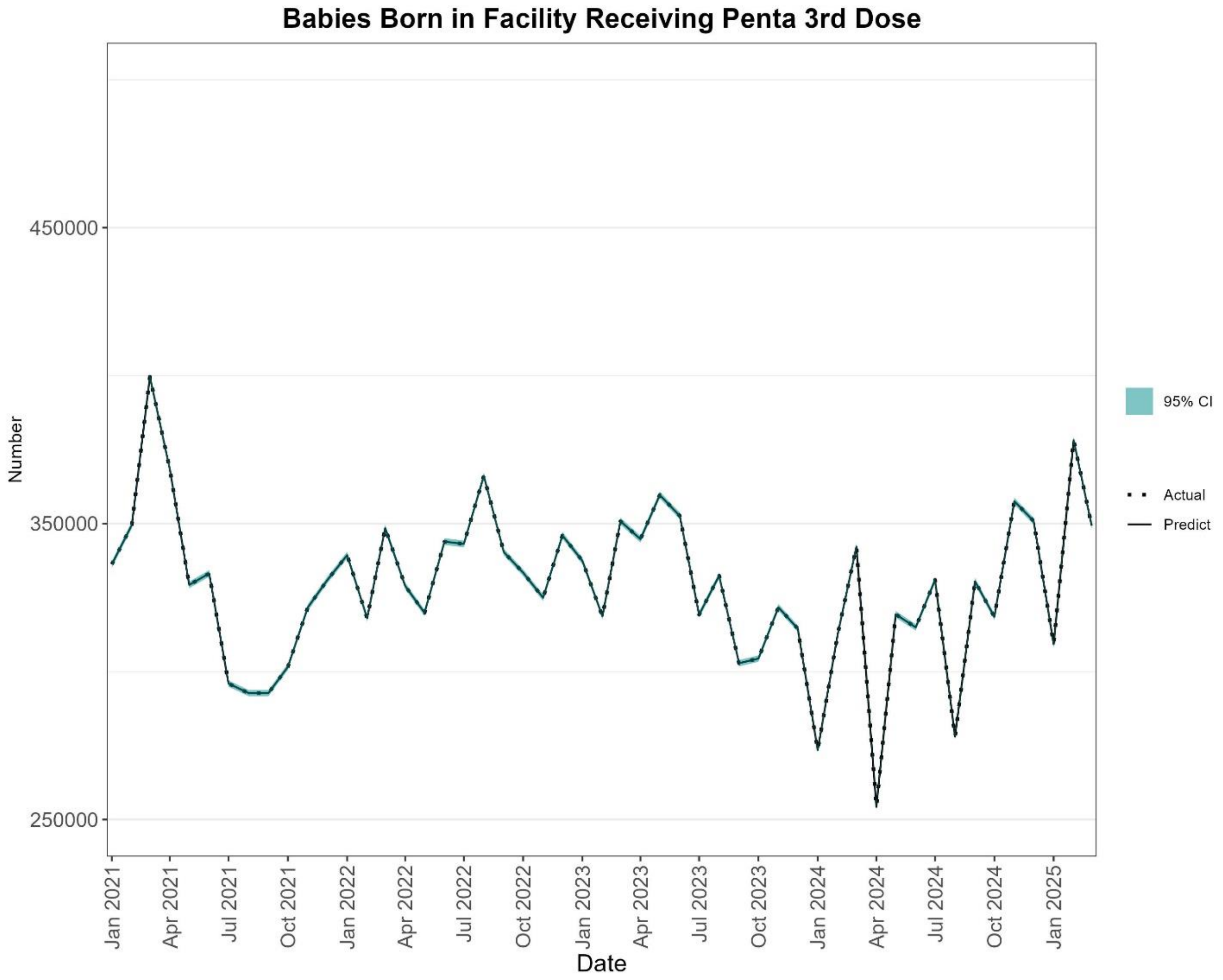
Babies Born in Facility Receiving Penta 3rd Dose
Number
450000
350000
250000
Jan 2021
Apr 2021
Jul 2021
Oct 2021
Jan 2022
Apr 2022
Jul 2022
Oct 2022
Jan 2023
Apr 2023
Jul 2023
Oct 2023
Jan 2024
Apr 2024
Jul 2024
Oct 2024
Jan 2025
Date
95% CI
Actual
Predict

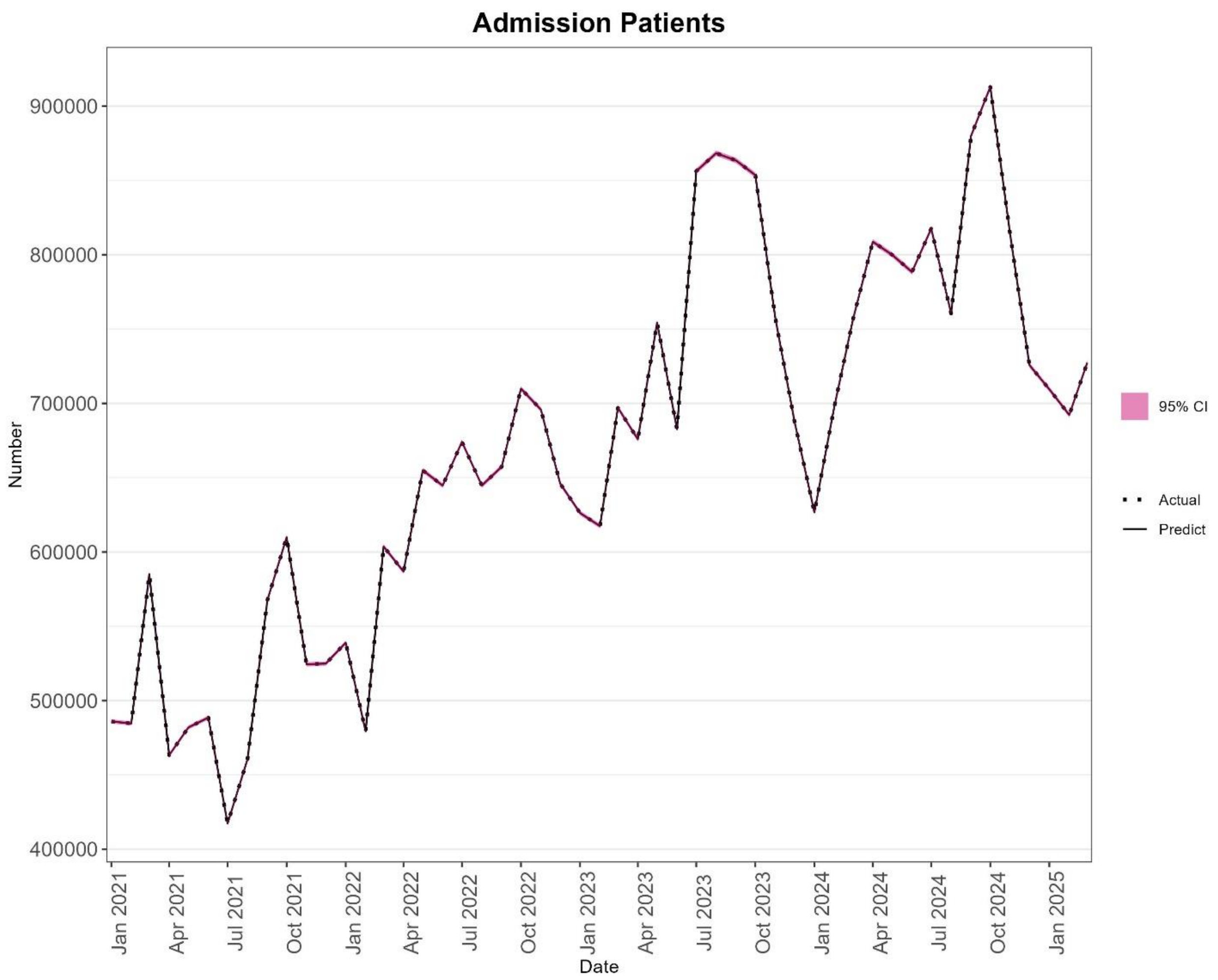
Admission Patients
900000
800000
700000
600000
500000
400000
Number
Jan 2021
Apr 2021
Jul 2021
Oct 2021
Jan 2022
Apr 2022
Jul 2022
Oct 2022
Jan 2023
Apr 2023
Jul 2023
Oct 2023
Jan 2024
Apr 2024
Jul 2024
Oct 2024
Jan 2025
Date
95% CI
Actual
Predict

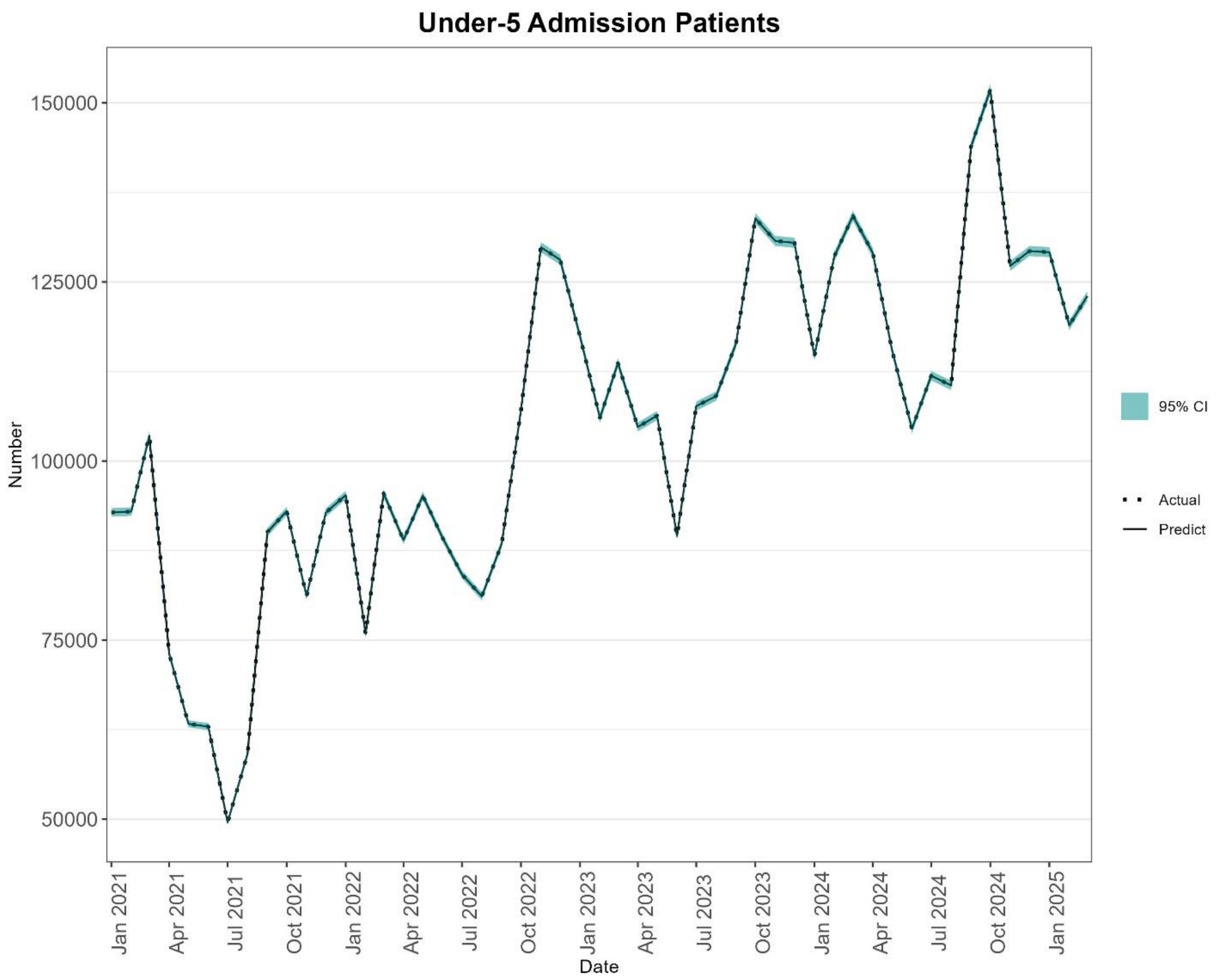
Under-5 Admission Patients
150000
125000
100000
75000
50000
Number
Jan 2021
Apr 2021
Jul 2021
Oct 2021
Jan 2022
Apr 2022
Jul 2022
Oct 2022
Jan 2023
Apr 2023
Jul 2023
Oct 2023
Jan 2024
Apr 2024
Jul 2024
Oct 2024
Jan 2025
Date
95% CI
Actual
Predict

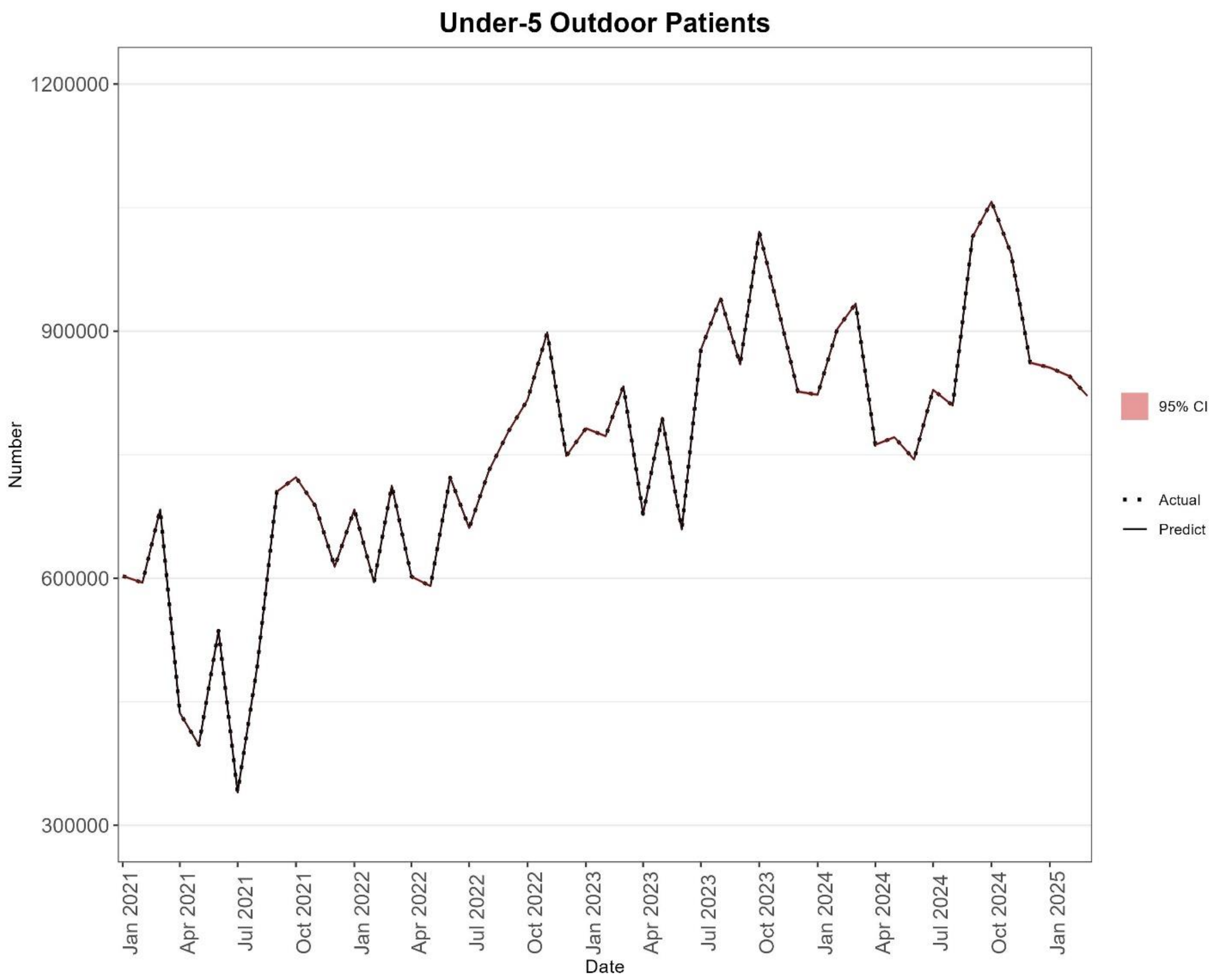

Under-5 Outdoor Patients
1200000
900000
600000
300000
Number
Jan 2021
Apr 2021
Jul 2021
Oct 2021
Jan 2022
Apr 2022
Jul 2022
Oct 2022
Jan 2023
Apr 2023
Jul 2023
Oct 2023
Jan 2024
Apr 2024
Jul 2024
Oct 2024
Jan 2025
Date
95% CI
Actual
Predict

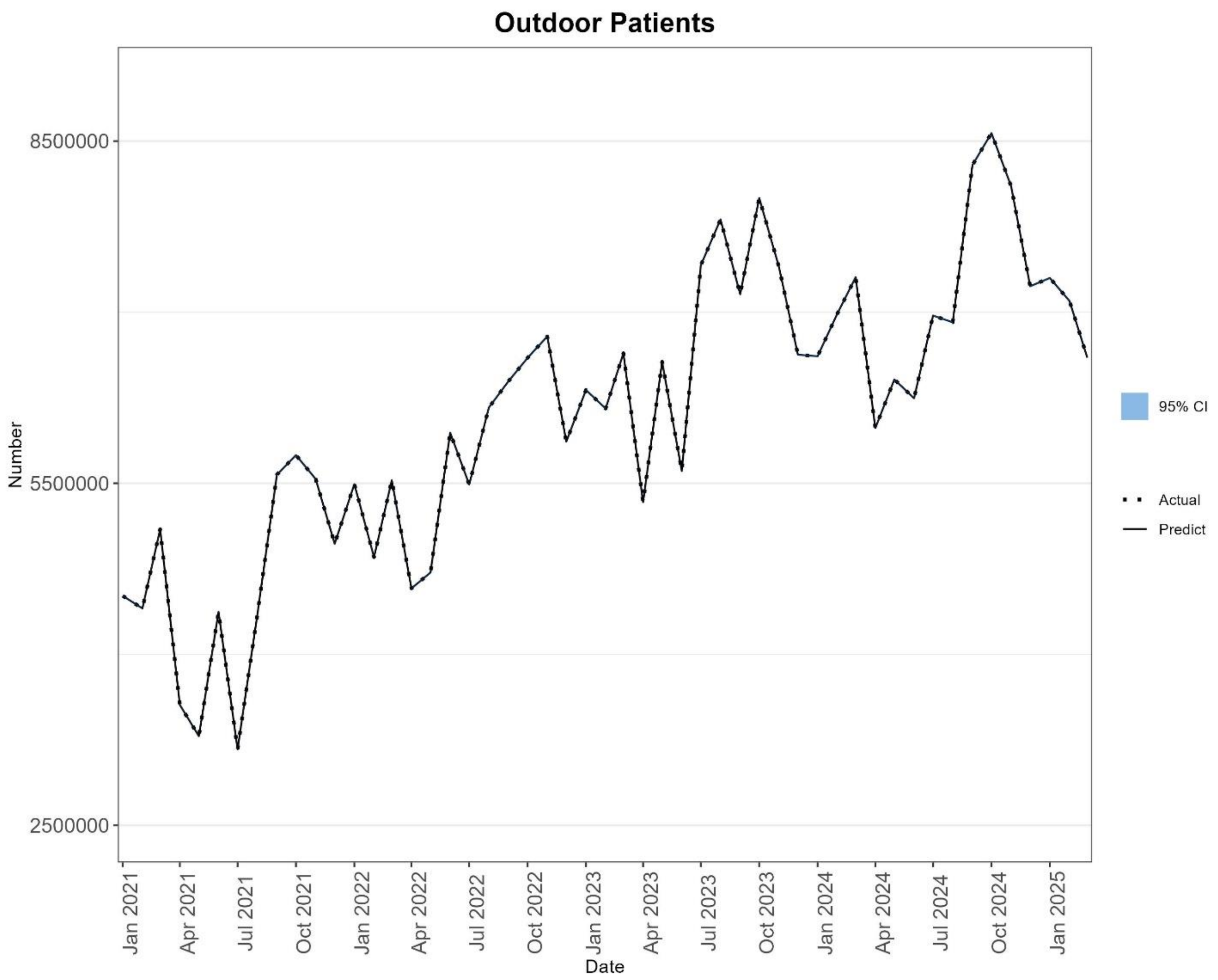

Outdoor Patients
8500000
5500000
2500000
Number
Jan 2021
Apr 2021
Jul 2021
Oct 2021
Jan 2022
Apr 2022
Jul 2022
Oct 2022
Jan 2023
Apr 2023
Jul 2023
Oct 2023
Jan 2024
Apr 2024
Jul 2024
Oct 2024
Jan 2025
Date
95% CI
Actual
Predict

Barishal

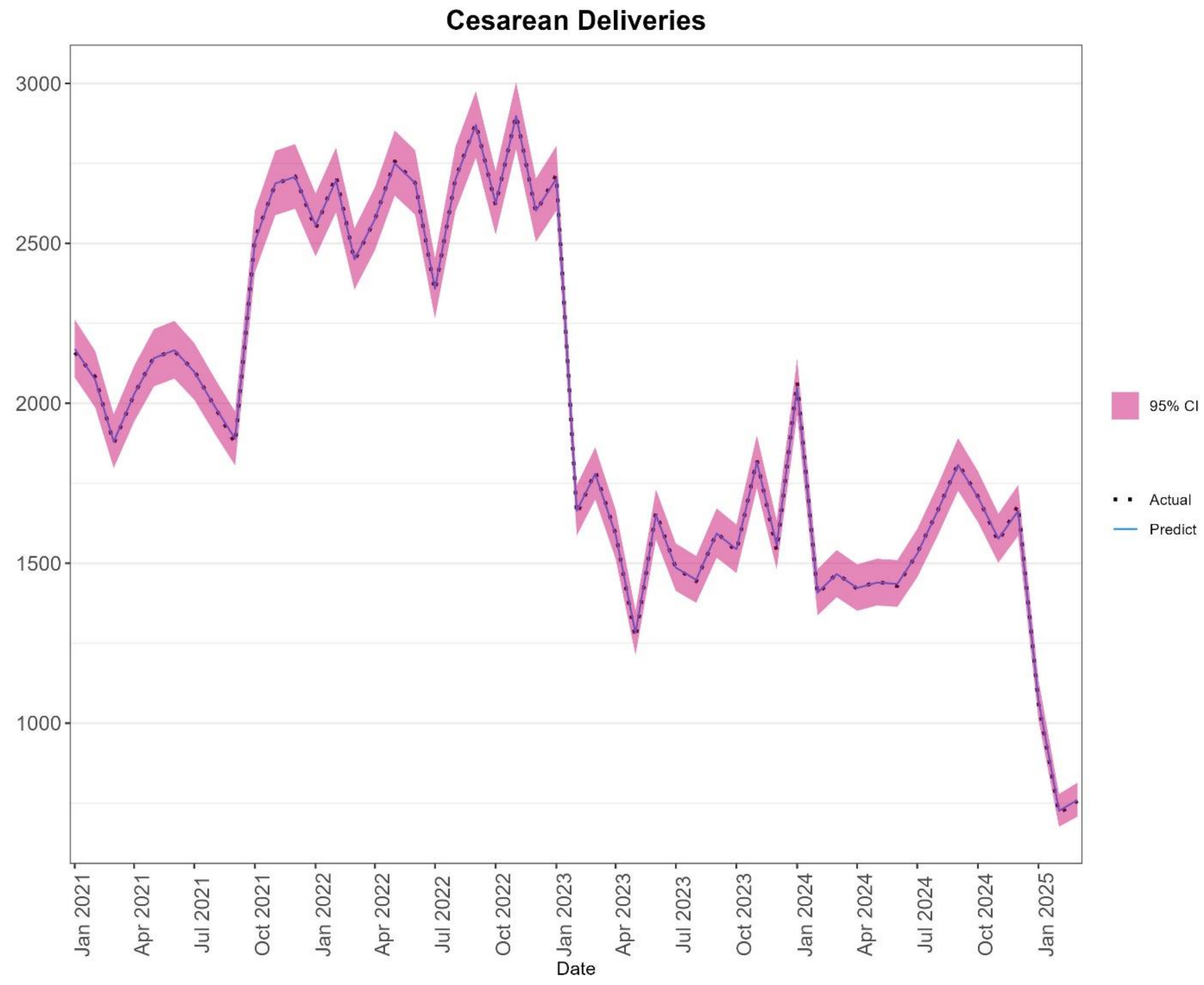

Cesarean Deliveries
3000
2500
2000
1500
1000
Jan 2021
Apr 2021
Jul 2021
Oct 2021
Jan 2022
Apr 2022
Jul 2022
Oct 2022
Jan 2023
Apr 2023
Jul 2023
Oct 2023
Jan 2024
Apr 2024
Jul 2024
Oct 2024
Jan 2025
Date
95% CI
Actual
Predict

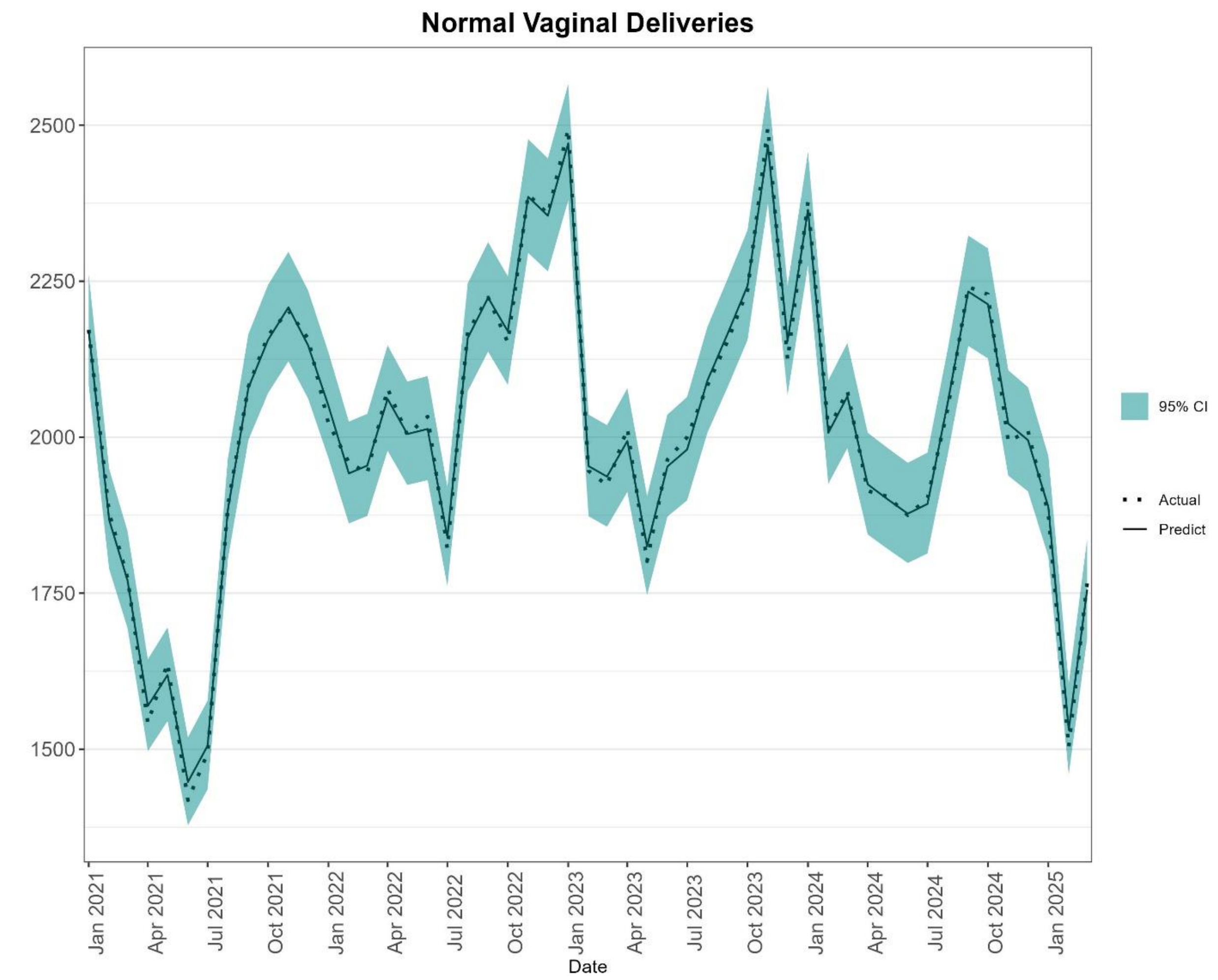
Normal Vaginal Deliveries
2500
2250
2000
1750
1500
Jan 2021
Apr 2021
Jul 2021
Oct 2021
Jan 2022
Apr 2022
Jul 2022
Oct 2022
Jan 2023
Apr 2023
Jul 2023
Oct 2023
Jan 2024
Apr 2024
Jul 2024
Oct 2024
Jan 2025
Date
95% CI
Actual
Predict

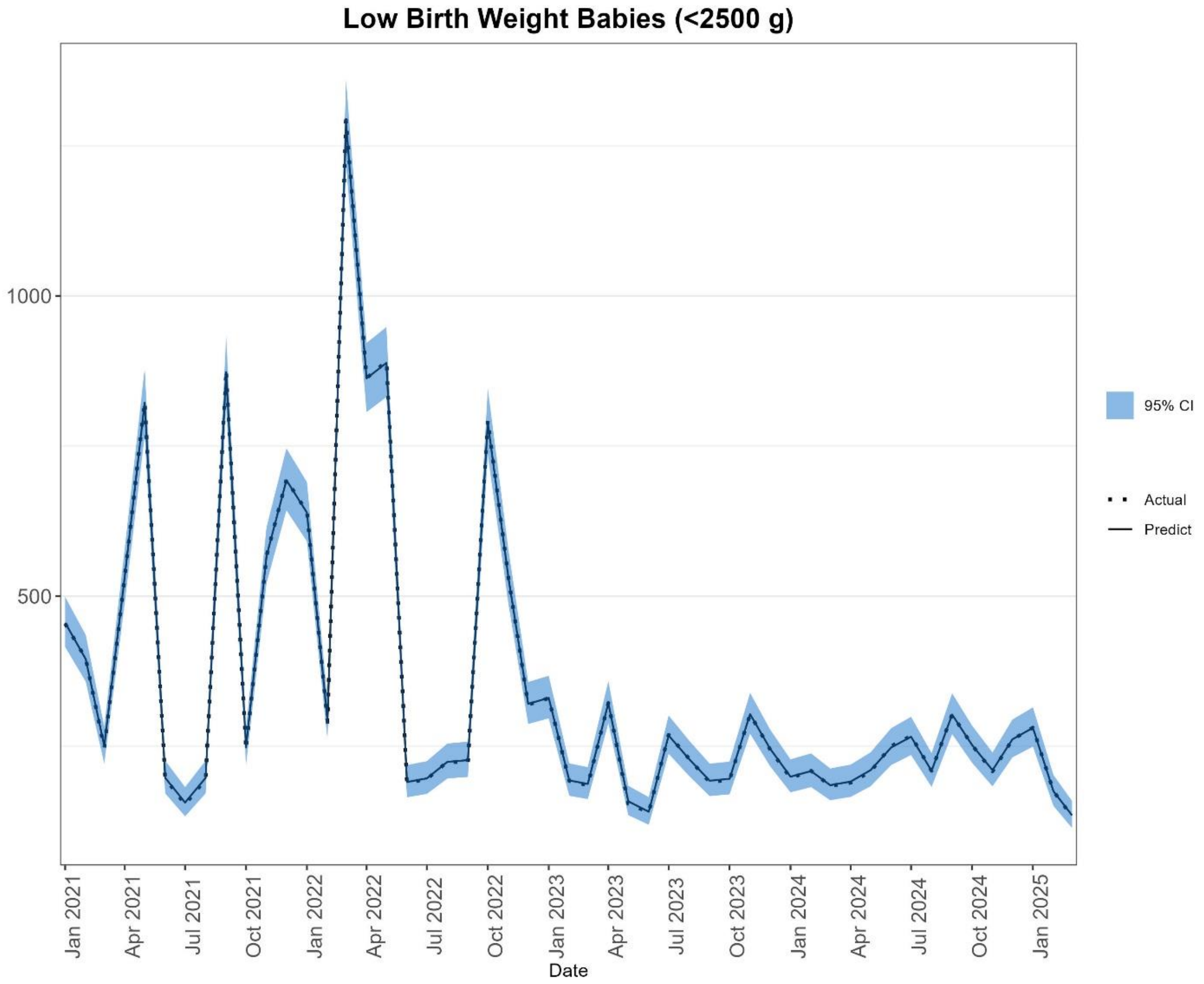
Low Birth Weight Babies (<2500 g)
1000
500
95% CI
Actual
Predict
Jan 2021
Apr 2021
Jul 2021
Oct 2021
Jan 2022
Apr 2022
Jul 2022
Oct 2022
Jan 2023
Apr 2023
Jul 2023
Oct 2023
Jan 2024
Apr 2024
Jul 2024
Oct 2024
Jan 2025
Date

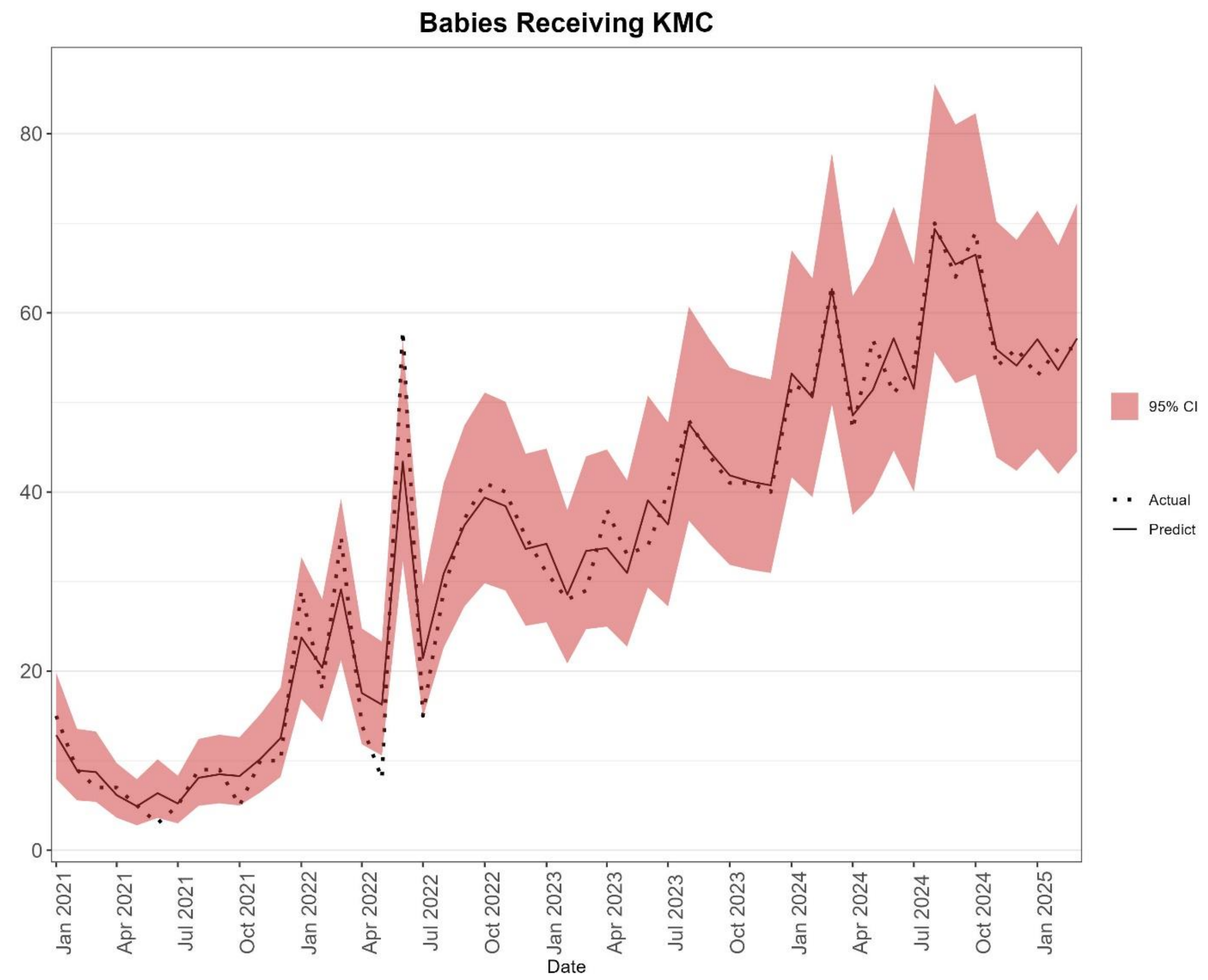

Babies Receiving KMC
0
20
40
60
80
Jan 2021
Apr 2021
Jul 2021
Oct 2021
Jan 2022
Apr 2022
Jul 2022
Oct 2022
Jan 2023
Apr 2023
Jul 2023
Oct 2023
Jan 2024
Apr 2024
Jul 2024
Oct 2024
Jan 2025
Date
95% CI
Actual
Predict

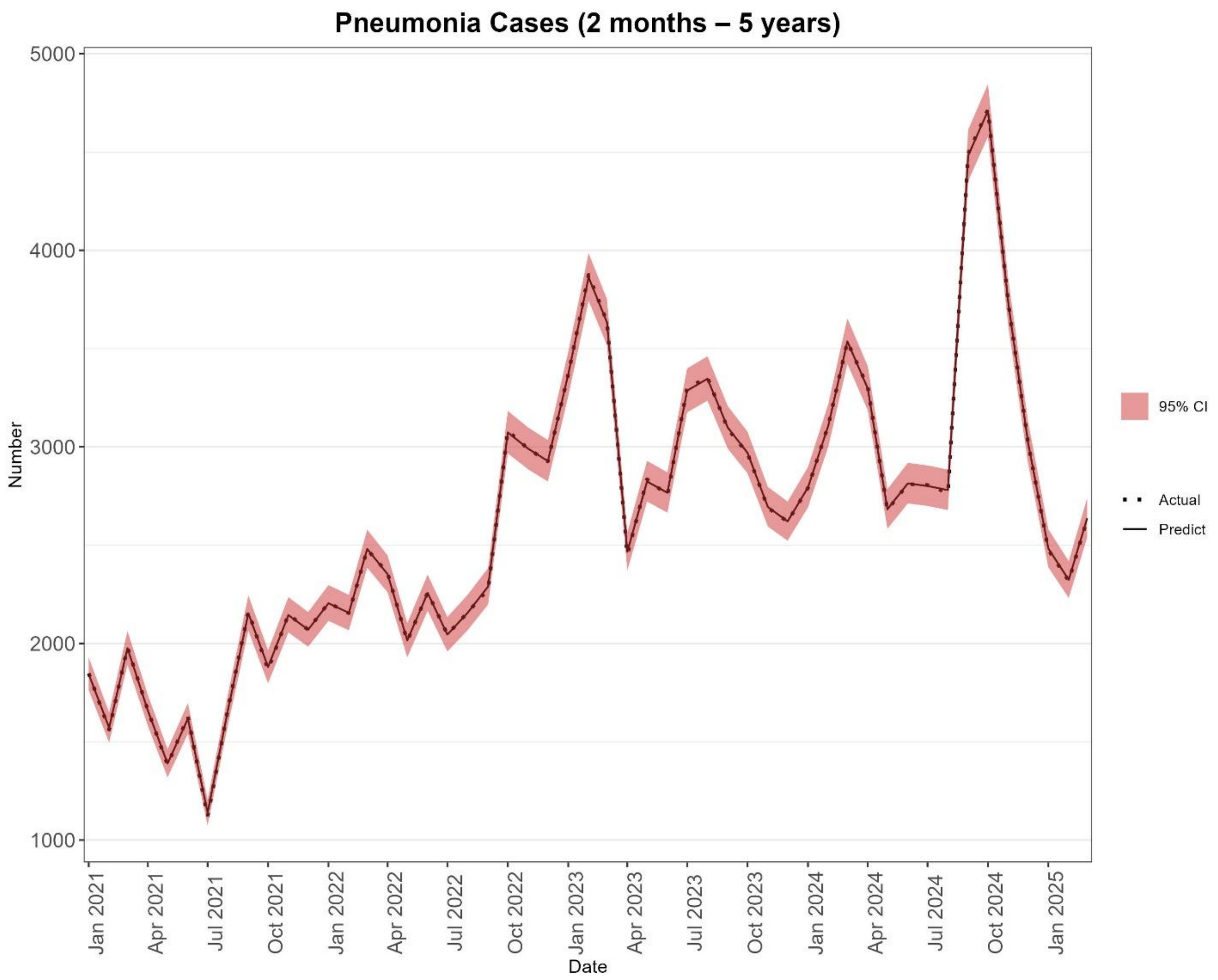
Pneumonia Cases (2 months – 5 years)
5000
4000
3000
2000
1000
Number
Jan 2021
Apr 2021
Jul 2021
Oct 2021
Jan 2022
Apr 2022
Jul 2022
Oct 2022
Jan 2023
Apr 2023
Jul 2023
Oct 2023
Jan 2024
Apr 2024
Jul 2024
Oct 2024
Jan 2025
Date
95% CI
Actual
Predict

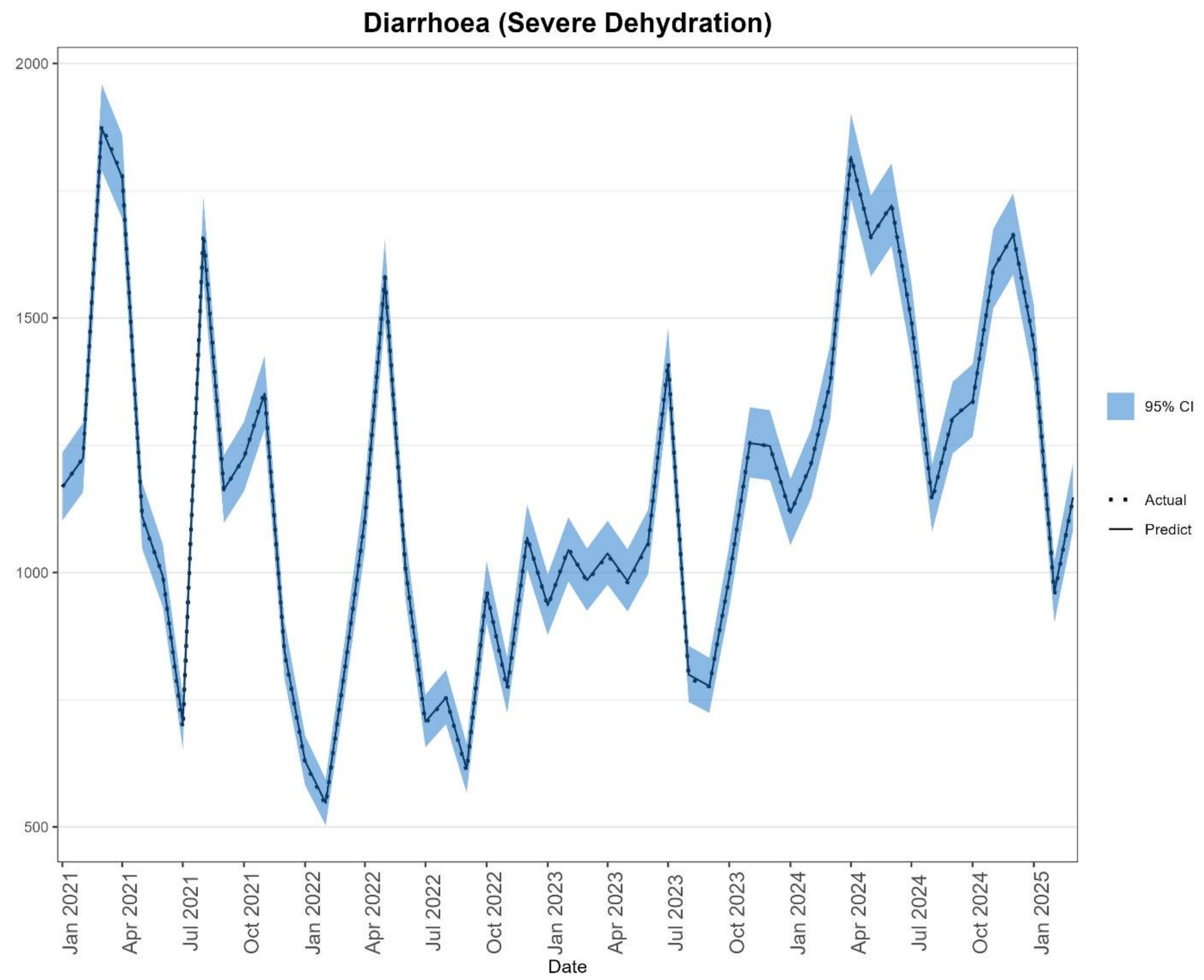

Diarrhoea (Severe Dehydration)
2000
1500
1000
500
95% CI
Actual
Predict
Jan 2021
Apr 2021
Jul 2021
Oct 2021
Jan 2022
Apr 2022
Jul 2022
Oct 2022
Jan 2023
Apr 2023
Jul 2023
Oct 2023
Jan 2024
Apr 2024
Jul 2024
Oct 2024
Jan 2025
Date

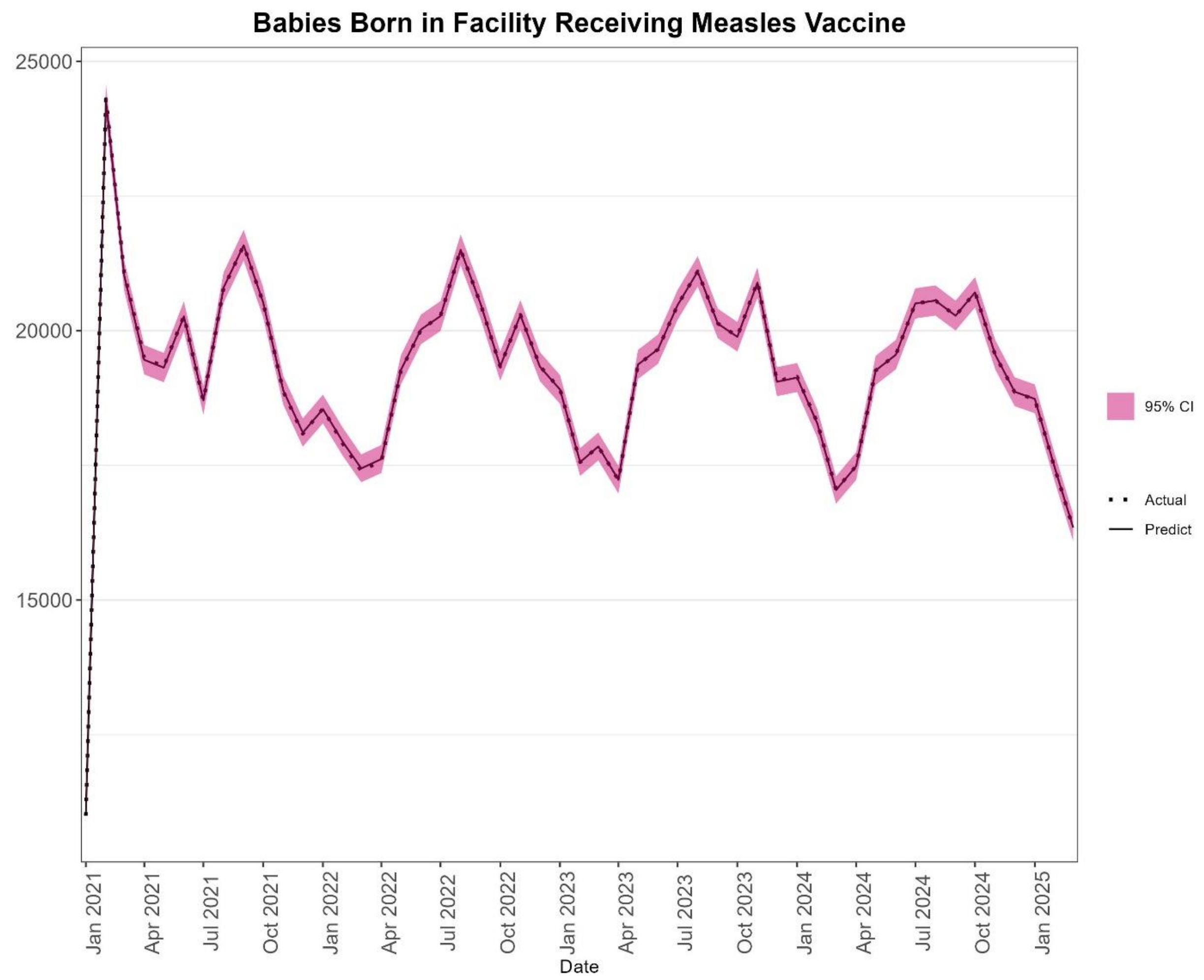

Babies Born in Facility Receiving Measles Vaccine
25000
20000
15000
Jan 2021
Apr 2021
Jul 2021
Oct 2021
Jan 2022
Apr 2022
Jul 2022
Oct 2022
Jan 2023
Apr 2023
Jul 2023
Oct 2023
Jan 2024
Apr 2024
Jul 2024
Oct 2024
Jan 2025
Date
95% CI
Actual
Predict

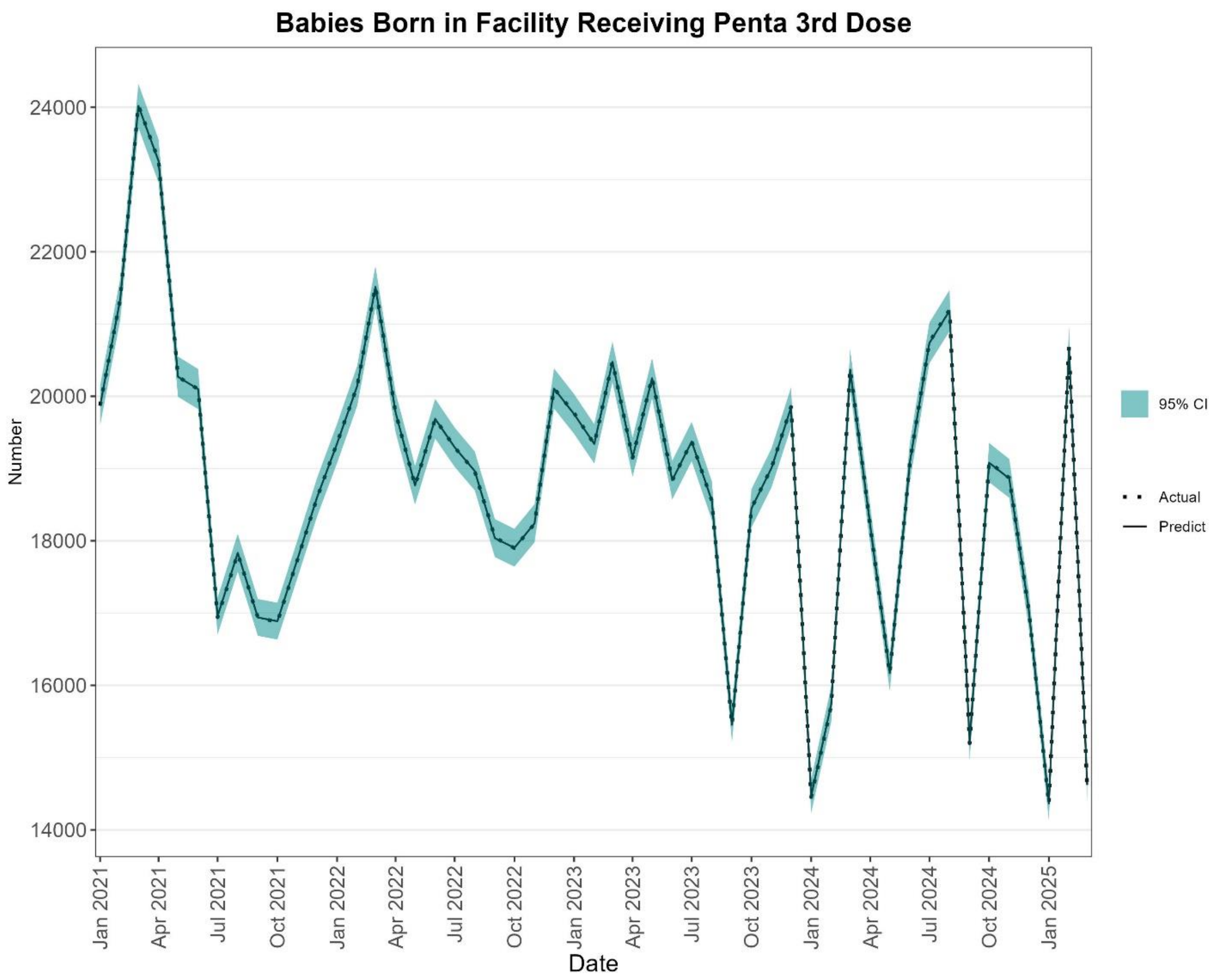

Babies Born in Facility Receiving Penta 3rd Dose
24000
22000
20000
18000
16000
14000
Number
Jan 2021
Apr 2021
Jul 2021
Oct 2021
Jan 2022
Apr 2022
Jul 2022
Oct 2022
Jan 2023
Apr 2023
Jul 2023
Oct 2023
Jan 2024
Apr 2024
Jul 2024
Oct 2024
Jan 2025
Date
95% CI
Actual
Predict

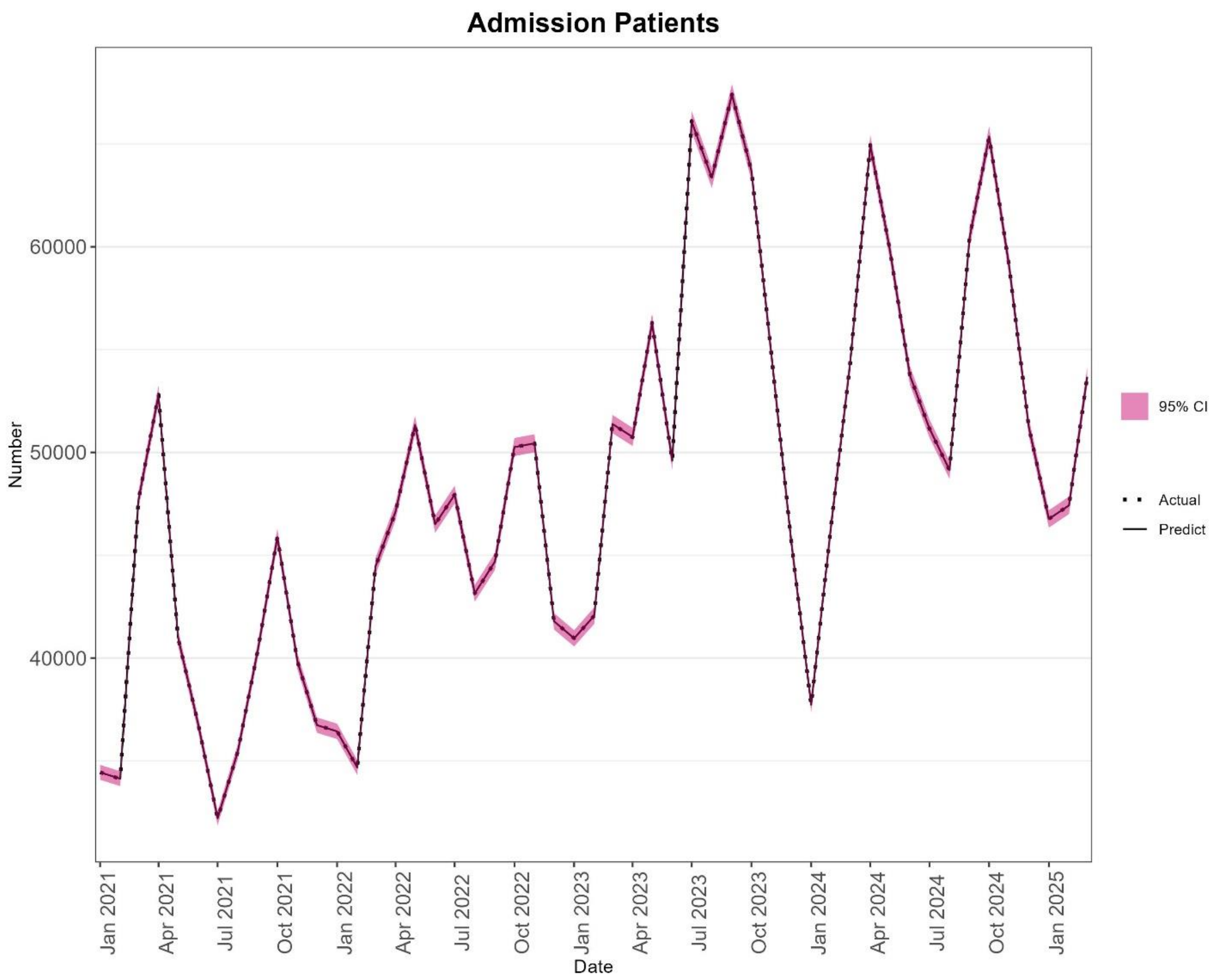

Admission Patients
60000
50000
40000
Number
Jan 2021
Apr 2021
Jul 2021
Oct 2021
Jan 2022
Apr 2022
Jul 2022
Oct 2022
Jan 2023
Apr 2023
Jul 2023
Oct 2023
Jan 2024
Apr 2024
Jul 2024
Oct 2024
Jan 2025
Date
95% CI
Actual
Predict

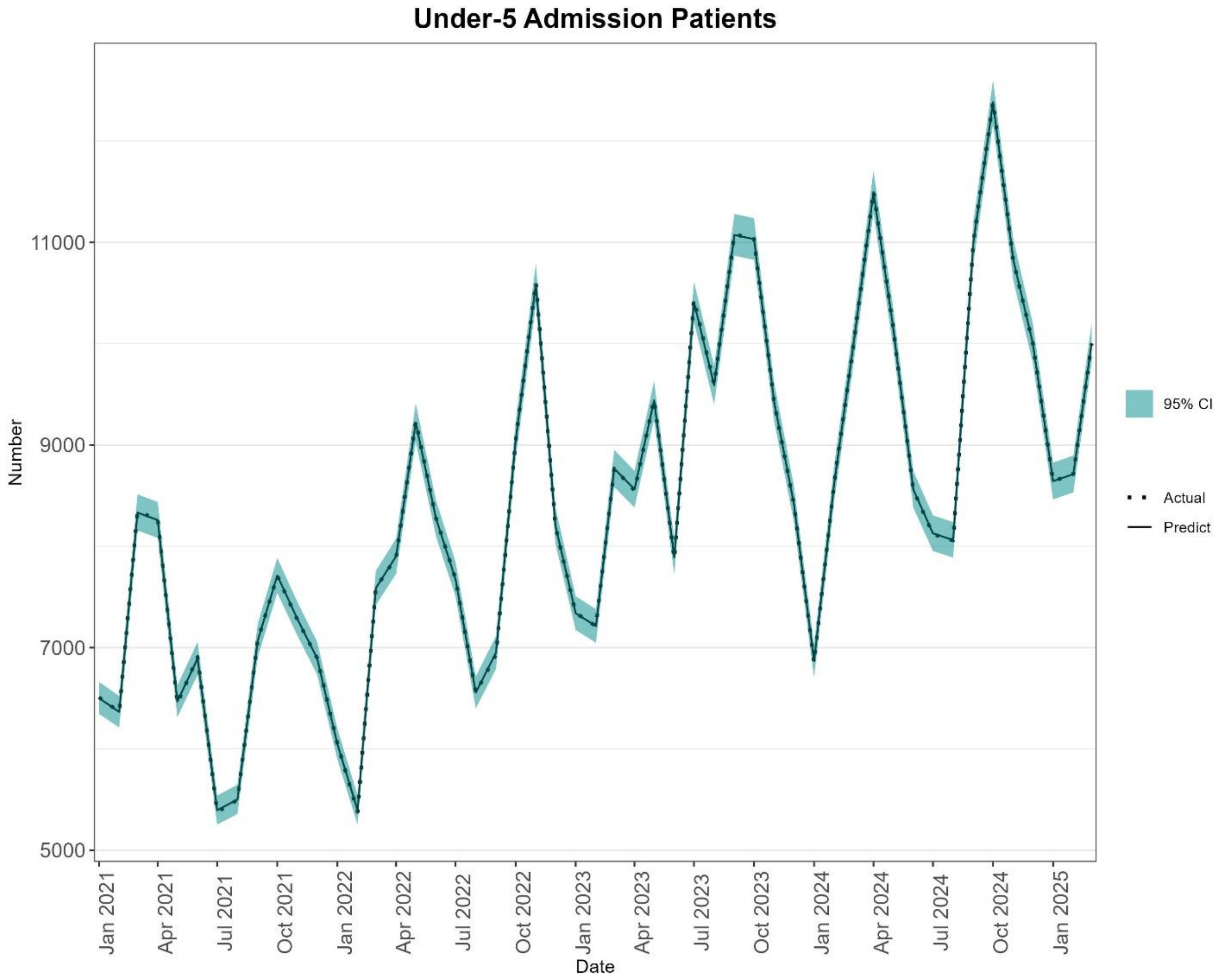
Under-5 Admission Patients
Number
11000
9000
7000
5000
Date
Jan 2021
Apr 2021
Jul 2021
Oct 2021
Jan 2022
Apr 2022
Jul 2022
Oct 2022
Jan 2023
Apr 2023
Jul 2023
Oct 2023
Jan 2024
Apr 2024
Jul 2024
Oct 2024
Jan 2025
95% CI
Actual
Predict

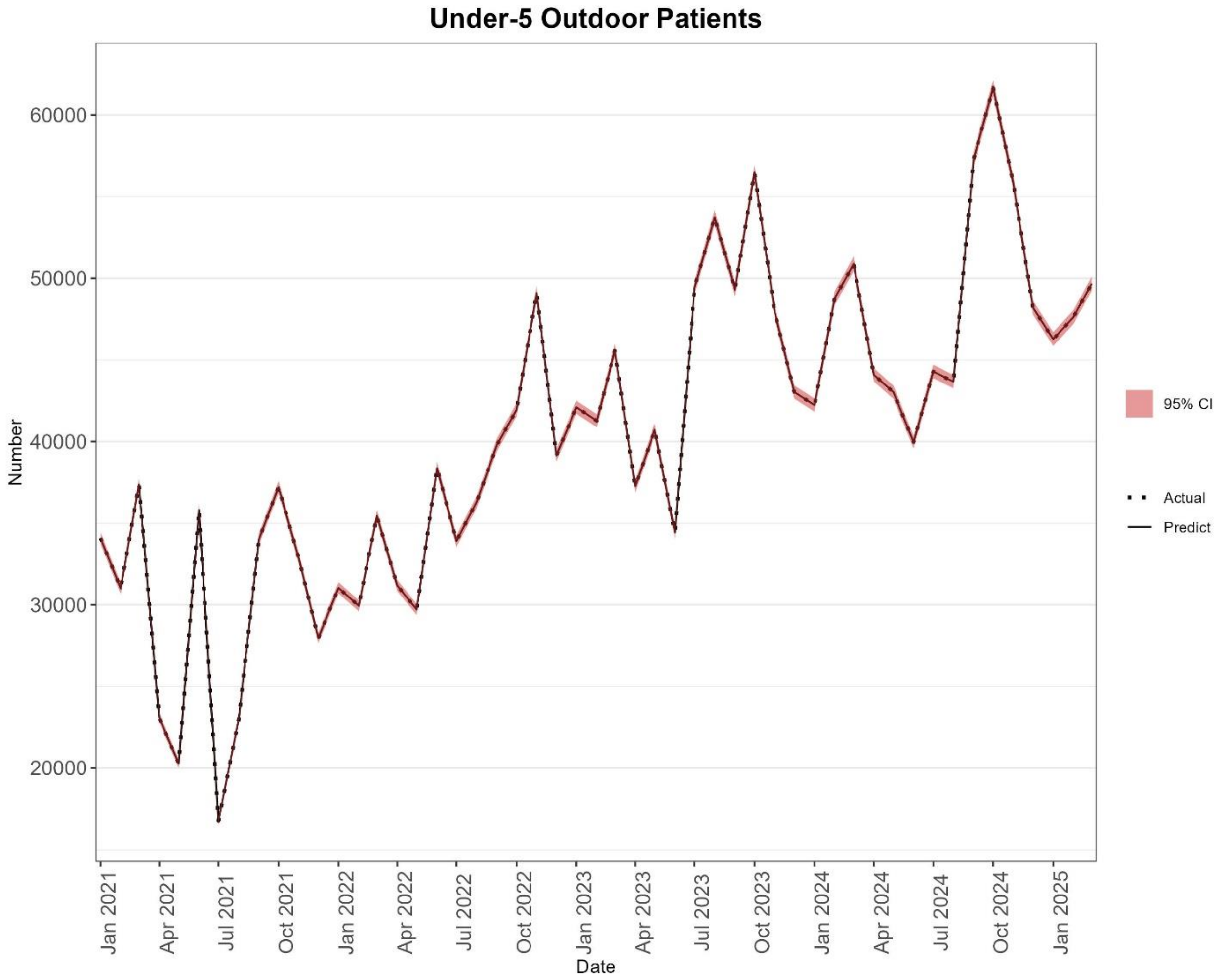

Under-5 Outdoor Patients
60000
50000
40000
30000
20000
Number
Jan 2021
Apr 2021
Jul 2021
Oct 2021
Jan 2022
Apr 2022
Jul 2022
Oct 2022
Jan 2023
Apr 2023
Jul 2023
Oct 2023
Jan 2024
Apr 2024
Jul 2024
Oct 2024
Jan 2025
Date
95% CI
Actual
Predict

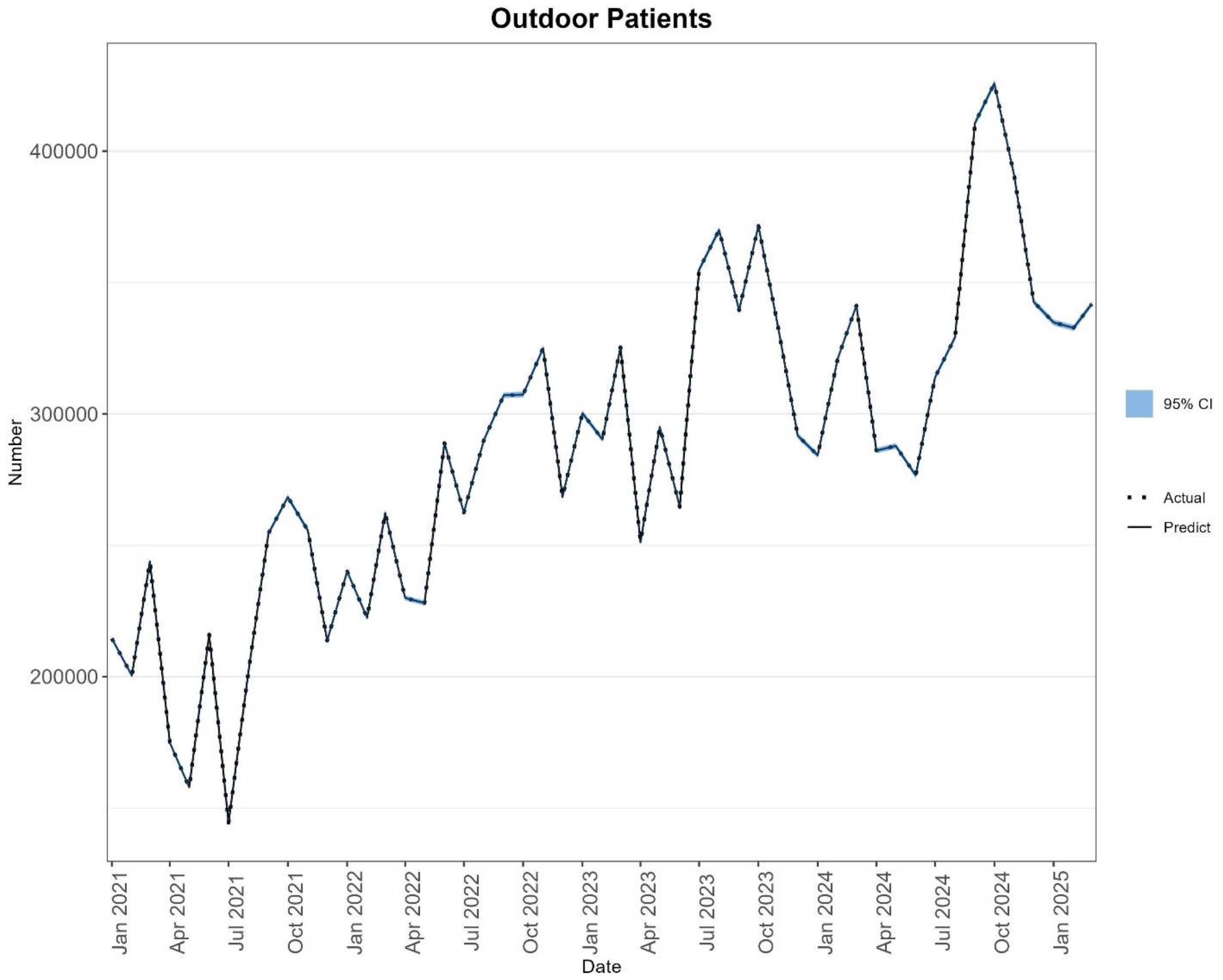

Outdoor Patients
400000
300000
200000
Number
Jan 2021
Apr 2021
Jul 2021
Oct 2021
Jan 2022
Apr 2022
Jul 2022
Oct 2022
Jan 2023
Apr 2023
Jul 2023
Oct 2023
Jan 2024
Apr 2024
Jul 2024
Oct 2024
Jan 2025
Date
95% CI
Actual
Predict

Chittagong

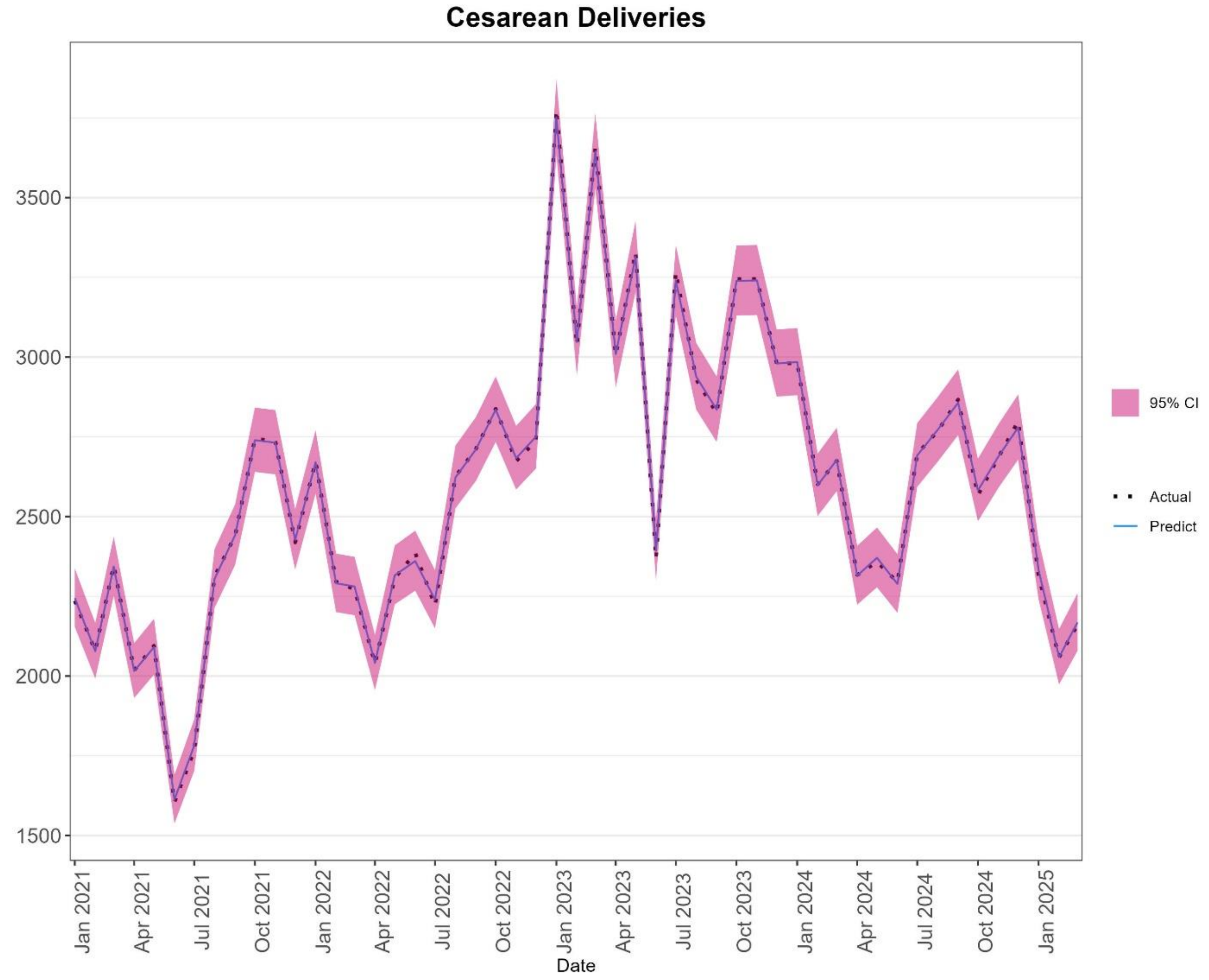

Cesarean Deliveries
3500
3000
2500
2000
1500
Jan 2021
Apr 2021
Jul 2021
Oct 2021
Jan 2022
Apr 2022
Jul 2022
Oct 2022
Jan 2023
Apr 2023
Jul 2023
Oct 2023
Jan 2024
Apr 2024
Jul 2024
Oct 2024
Jan 2025
Date
95% CI
Actual
Predict

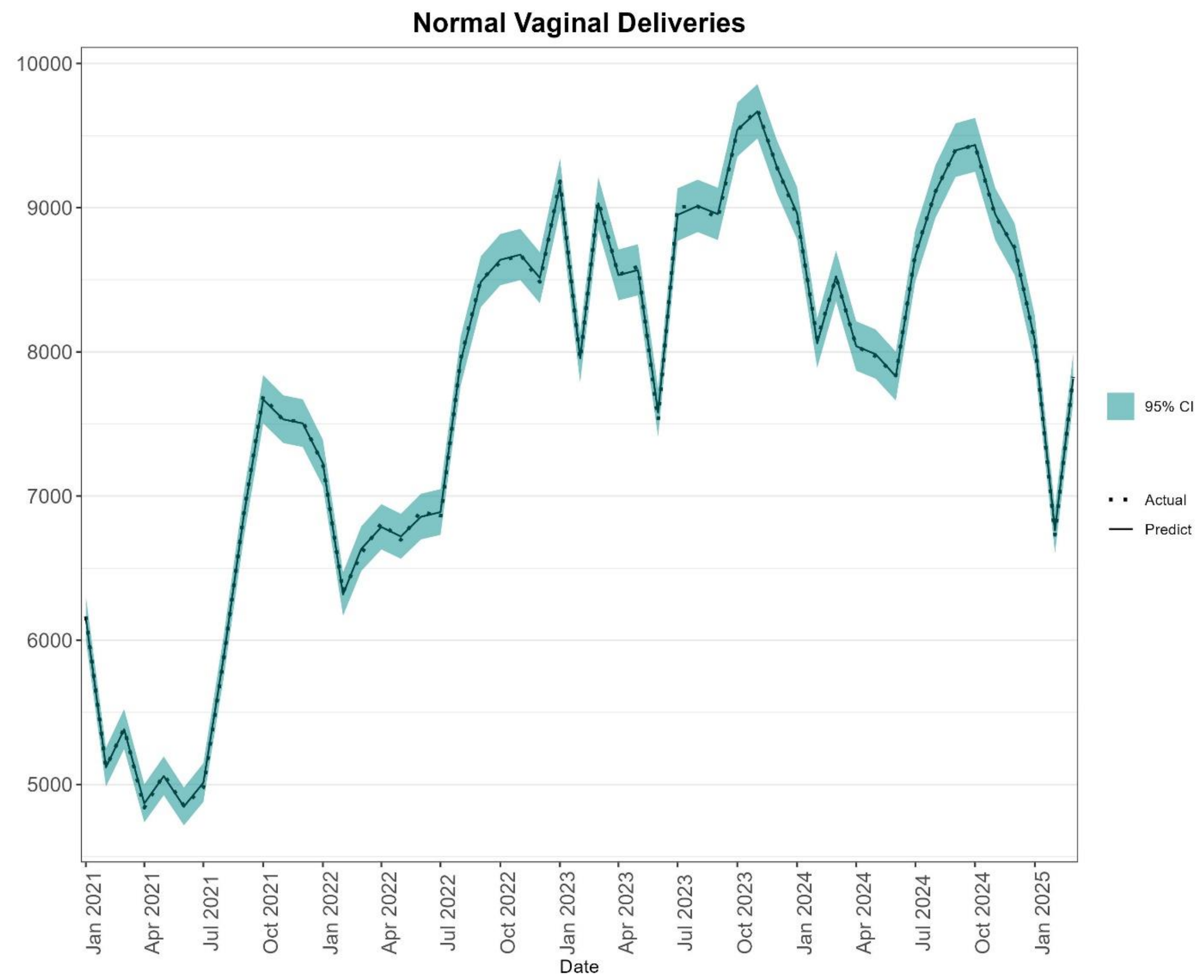
Normal Vaginal Deliveries
10000
9000
8000
7000
6000
5000
Jan 2021
Apr 2021
Jul 2021
Oct 2021
Jan 2022
Apr 2022
Jul 2022
Oct 2022
Jan 2023
Apr 2023
Jul 2023
Oct 2023
Jan 2024
Apr 2024
Jul 2024
Oct 2024
Jan 2025
Date
95% CI
Actual
Predict

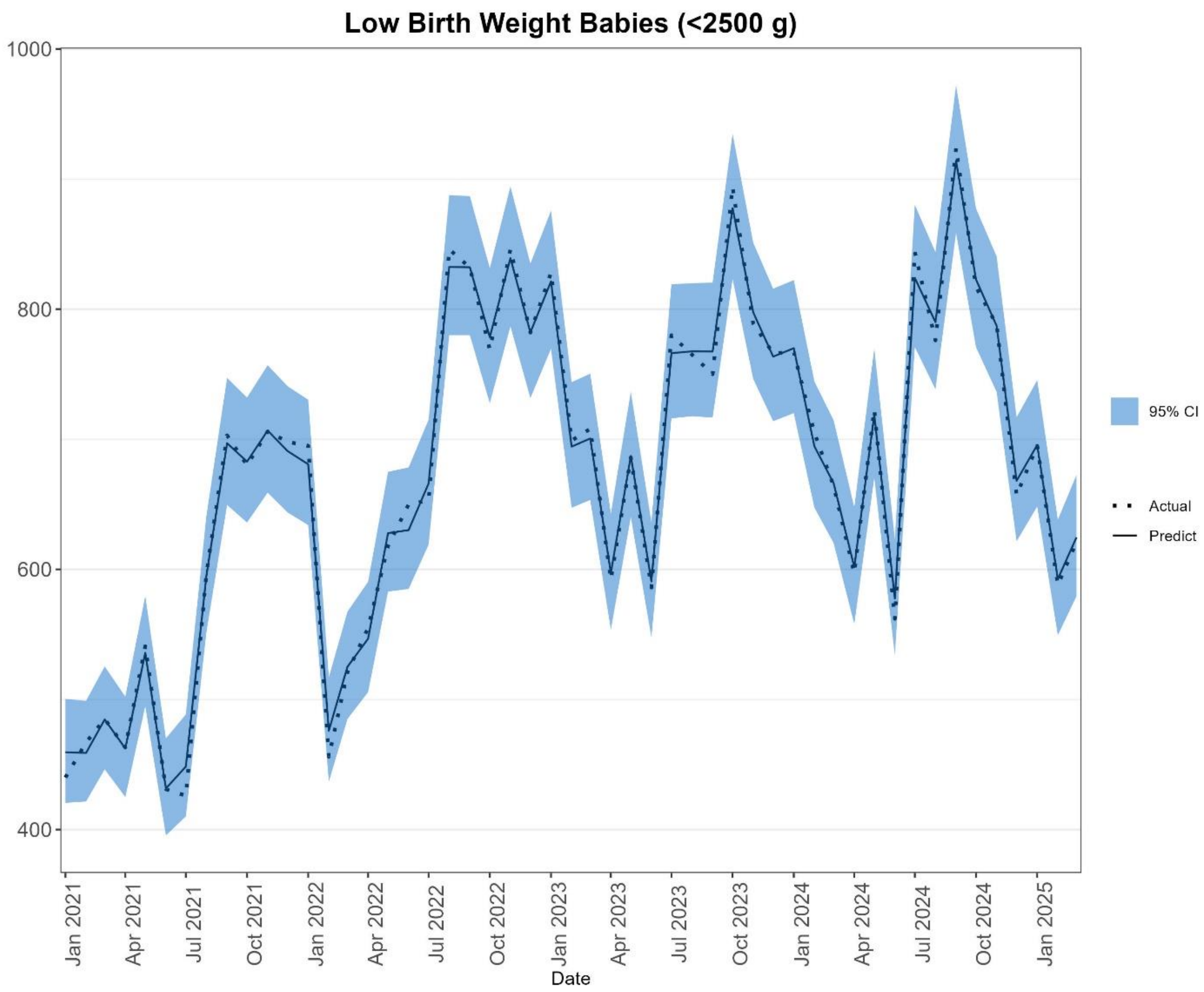

Low Birth Weight Babies (<2500 g)
1000
800
600
400
Jan 2021
Apr 2021
Jul 2021
Oct 2021
Jan 2022
Apr 2022
Jul 2022
Oct 2022
Jan 2023
Apr 2023
Jul 2023
Oct 2023
Jan 2024
Apr 2024
Jul 2024
Oct 2024
Jan 2025
Date
95% CI
Actual
Predict

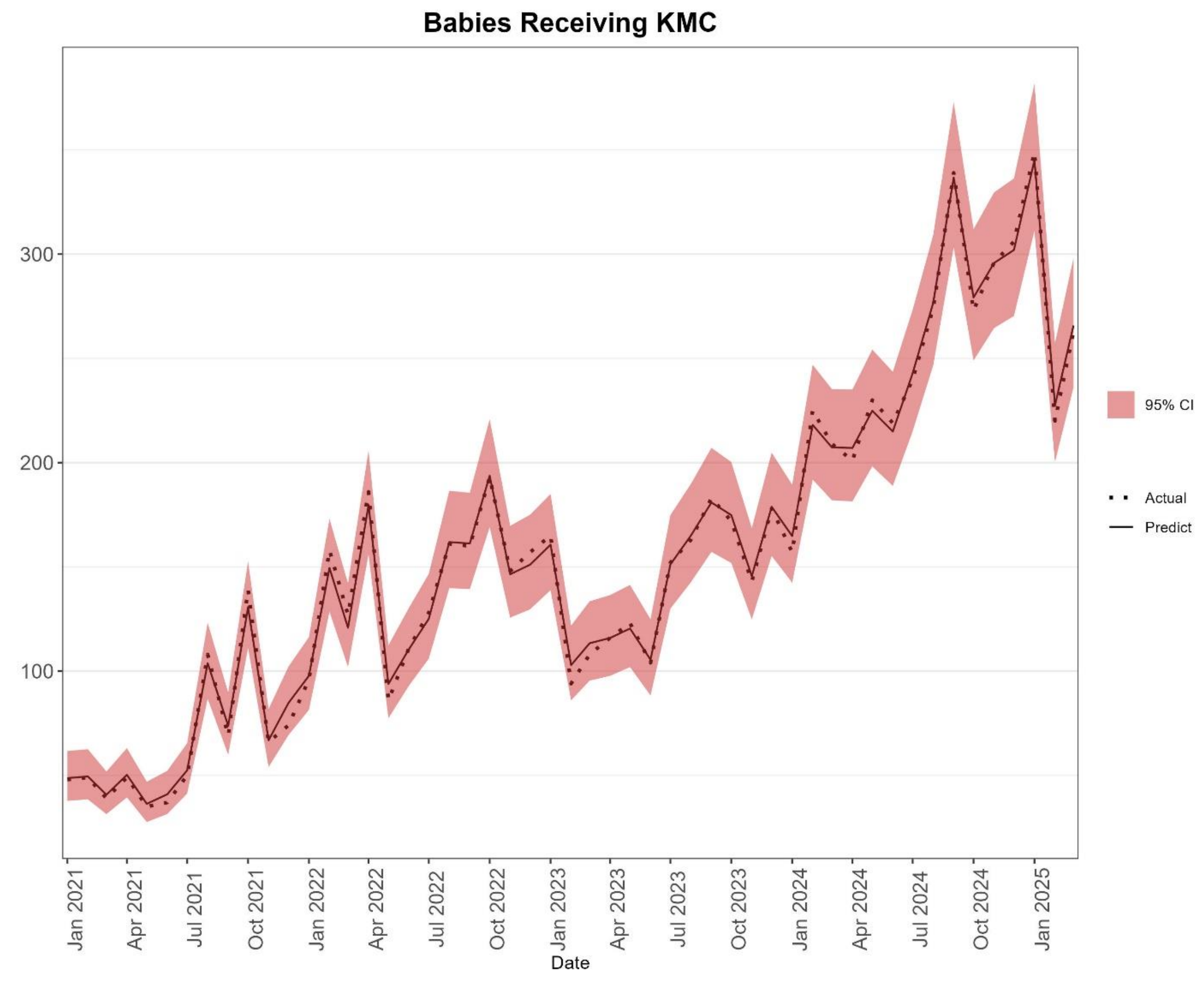
Babies Receiving KMC
300
200
100
Jan 2021
Apr 2021
Jul 2021
Oct 2021
Jan 2022
Apr 2022
Jul 2022
Oct 2022
Jan 2023
Apr 2023
Jul 2023
Oct 2023
Jan 2024
Apr 2024
Jul 2024
Oct 2024
Jan 2025
Date
95% CI
Actual
Predict

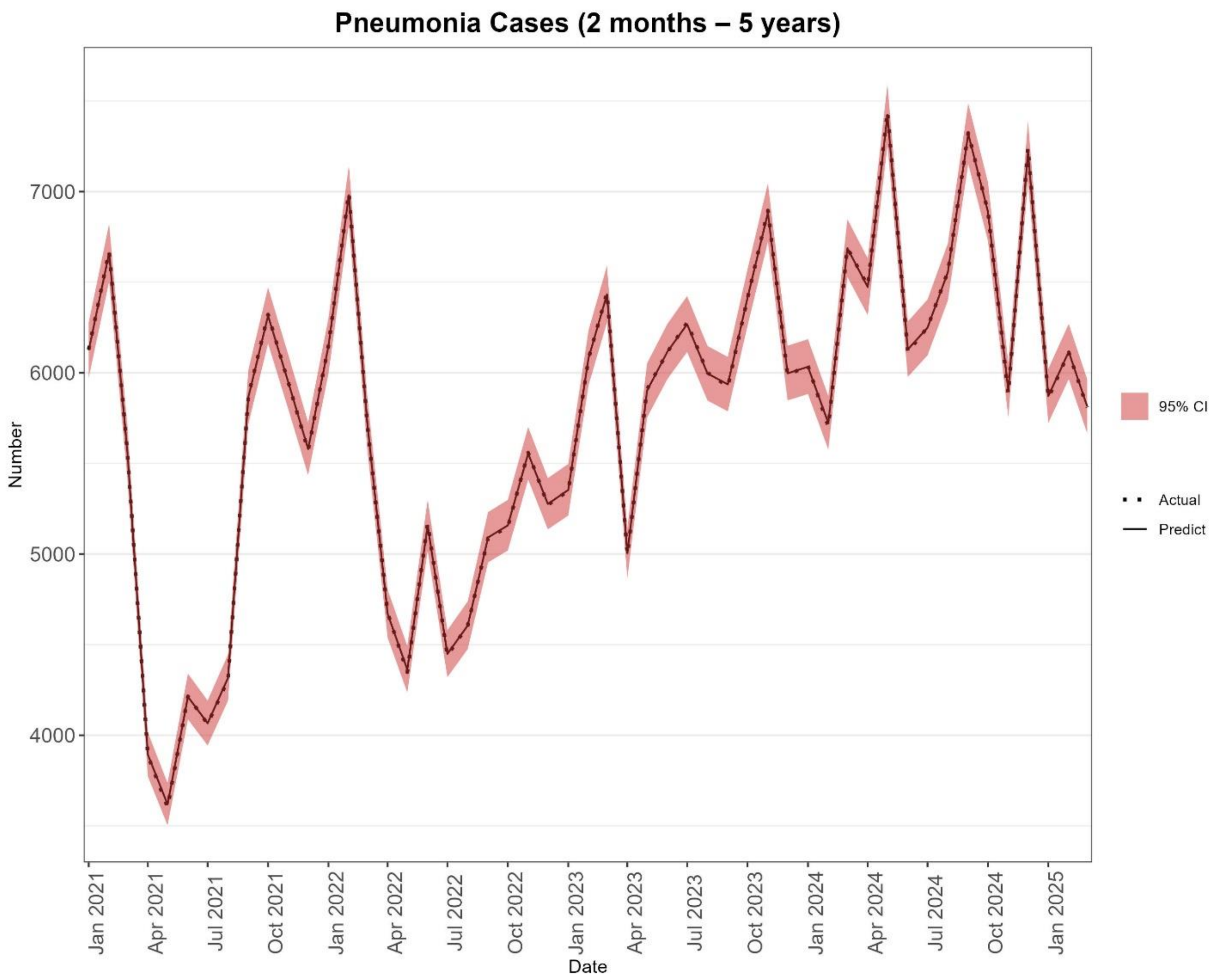
Pneumonia Cases (2 months – 5 years)
Number
7000
6000
5000
4000
Date
Jan 2021
Apr 2021
Jul 2021
Oct 2021
Jan 2022
Apr 2022
Jul 2022
Oct 2022
Jan 2023
Apr 2023
Jul 2023
Oct 2023
Jan 2024
Apr 2024
Jul 2024
Oct 2024
Jan 2025
95% CI
Actual
Predict

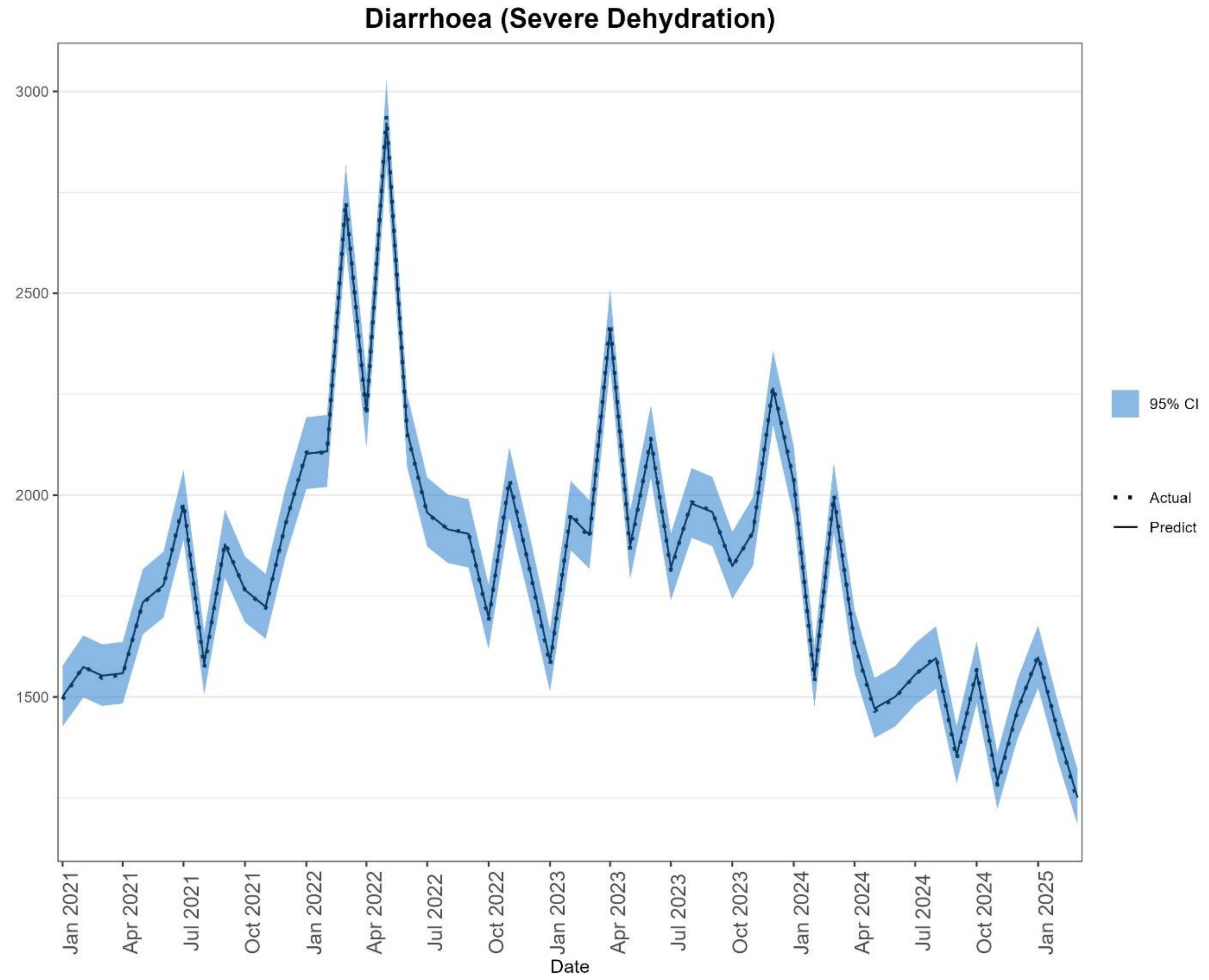

Diarrhoea (Severe Dehydration)
3000
2500
2000
1500
Jan 2021
Apr 2021
Jul 2021
Oct 2021
Jan 2022
Apr 2022
Jul 2022
Oct 2022
Jan 2023
Apr 2023
Jul 2023
Oct 2023
Jan 2024
Apr 2024
Jul 2024
Oct 2024
Jan 2025
Date
95% CI
Actual
Predict

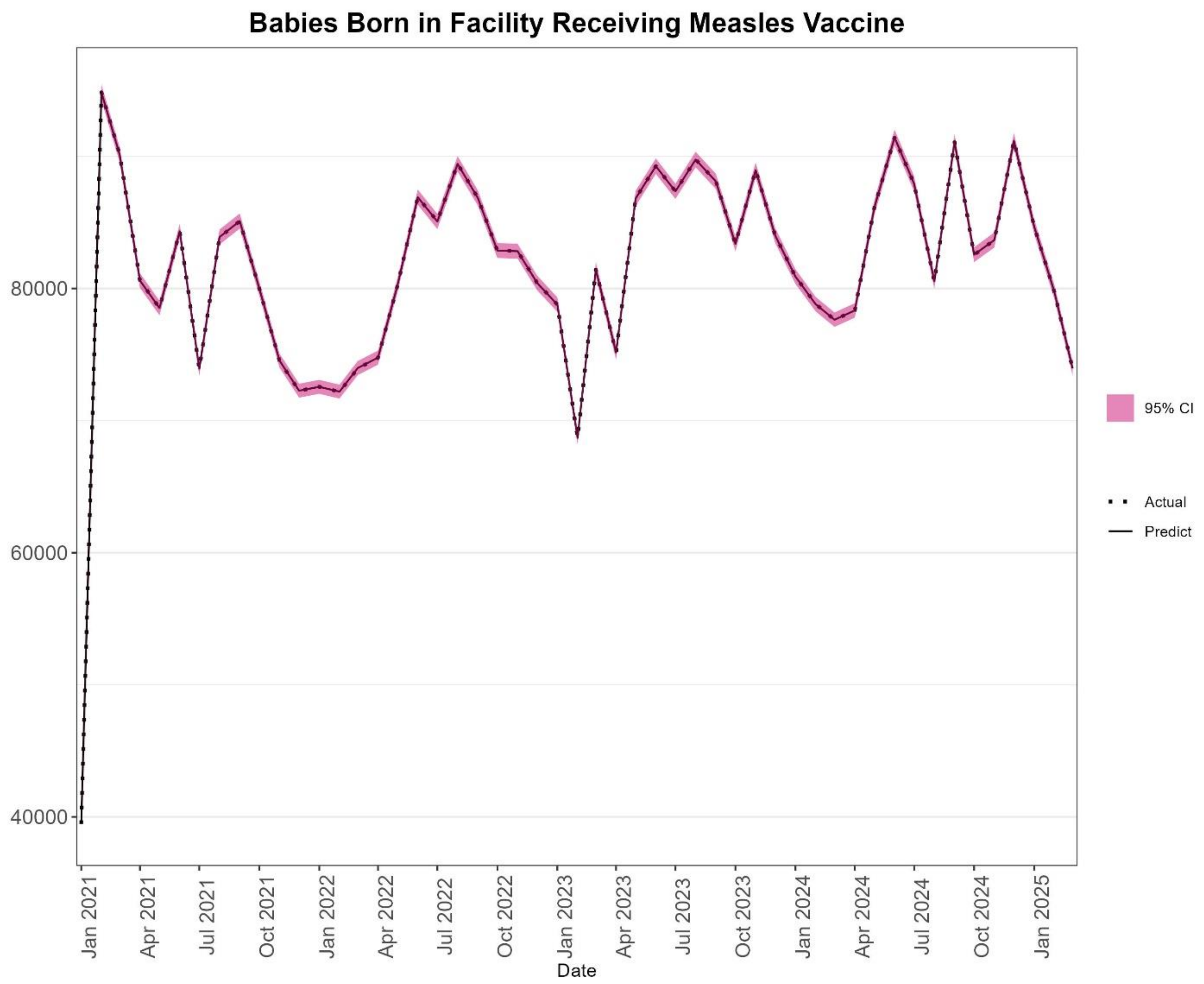

Babies Born in Facility Receiving Measles Vaccine
80000
60000
40000
Jan 2021
Apr 2021
Jul 2021
Oct 2021
Jan 2022
Apr 2022
Jul 2022
Oct 2022
Jan 2023
Apr 2023
Jul 2023
Oct 2023
Jan 2024
Apr 2024
Jul 2024
Oct 2024
Jan 2025
Date
95% CI
Actual
Predict

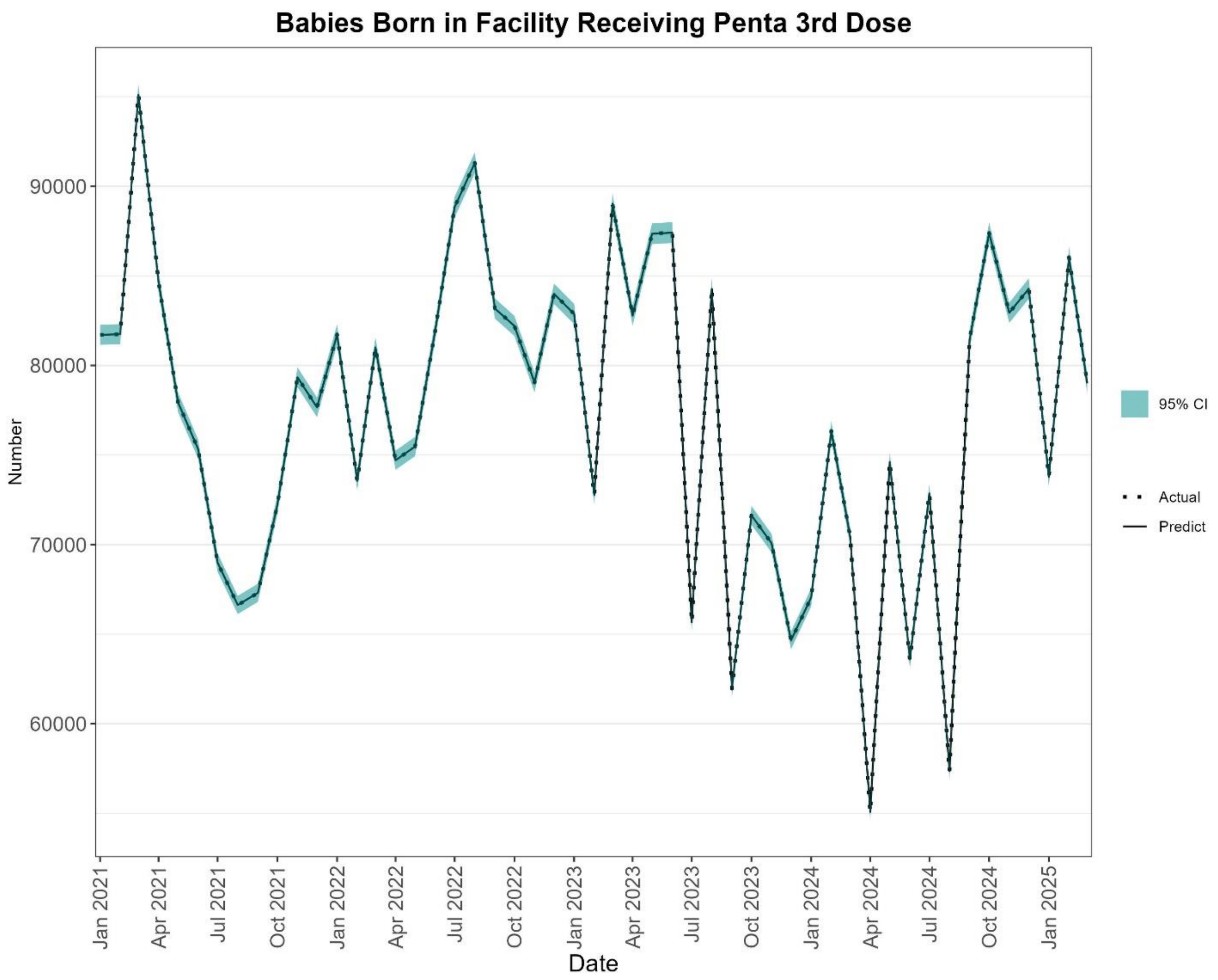
Babies Born in Facility Receiving Penta 3rd Dose
Number
90000
80000
70000
60000
Jan 2021
Apr 2021
Jul 2021
Oct 2021
Jan 2022
Apr 2022
Jul 2022
Oct 2022
Jan 2023
Apr 2023
Jul 2023
Oct 2023
Jan 2024
Apr 2024
Jul 2024
Oct 2024
Jan 2025
Date
95% CI
Actual
Predict

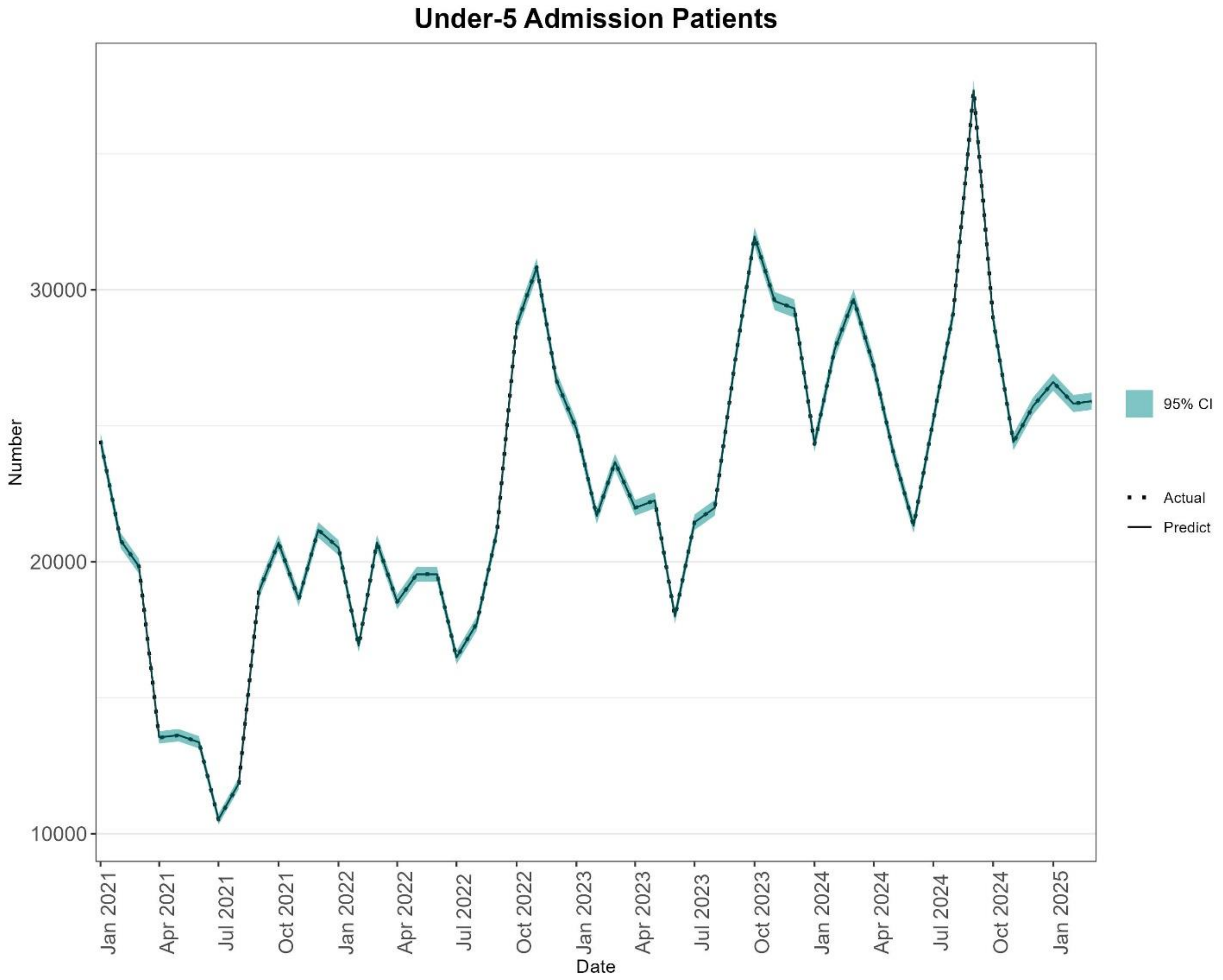
Under-5 Admission Patients
Number
30000
20000
10000
Jan 2021
Apr 2021
Jul 2021
Oct 2021
Jan 2022
Apr 2022
Jul 2022
Oct 2022
Jan 2023
Apr 2023
Jul 2023
Oct 2023
Jan 2024
Apr 2024
Jul 2024
Oct 2024
Jan 2025
Date
95% CI
Actual
Predict

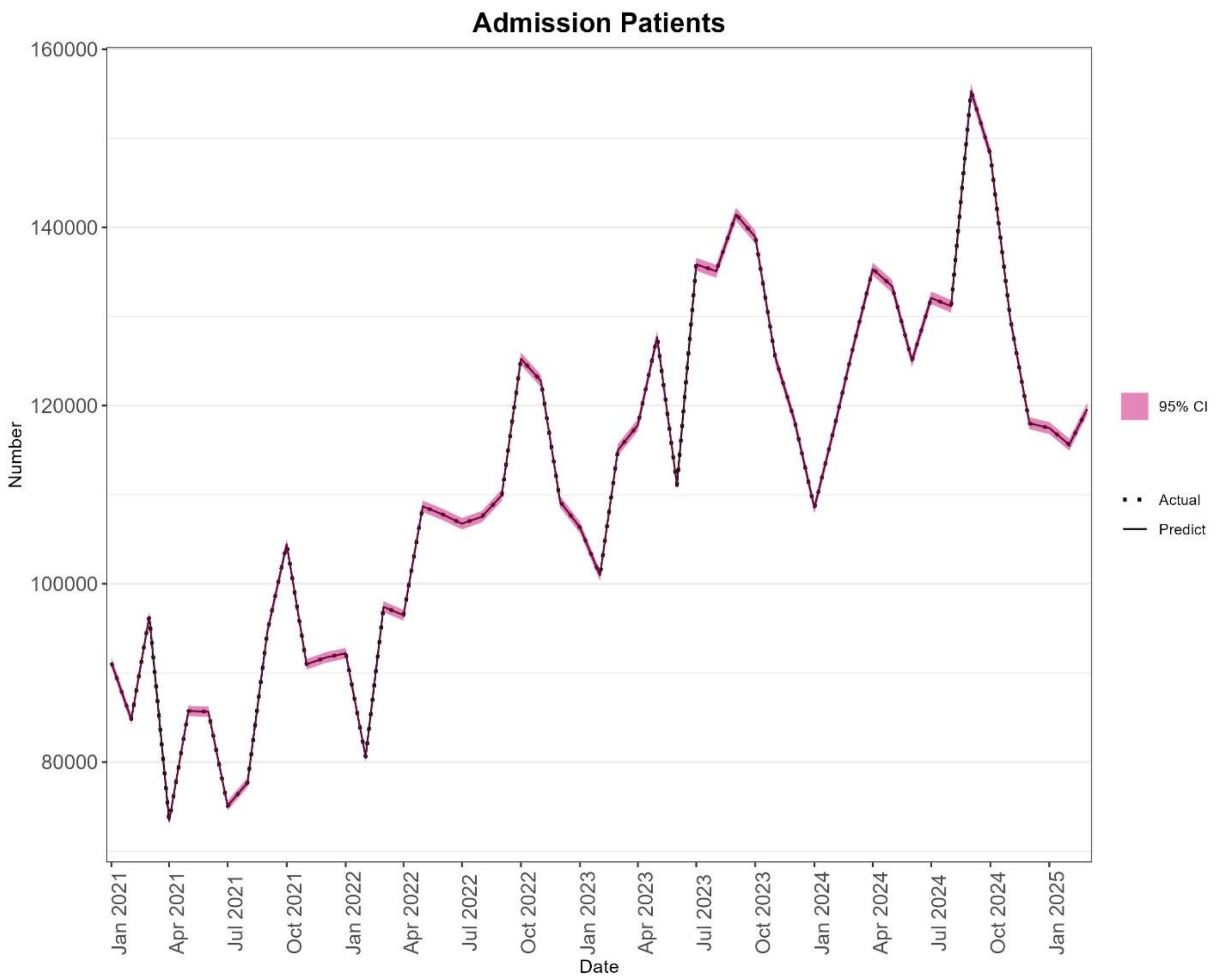

Admission Patients
160000
140000
120000
100000
80000
Number
Jan 2021
Apr 2021
Jul 2021
Oct 2021
Jan 2022
Apr 2022
Jul 2022
Oct 2022
Jan 2023
Apr 2023
Jul 2023
Oct 2023
Jan 2024
Apr 2024
Jul 2024
Oct 2024
Jan 2025
Date
95% CI
Actual
Predict

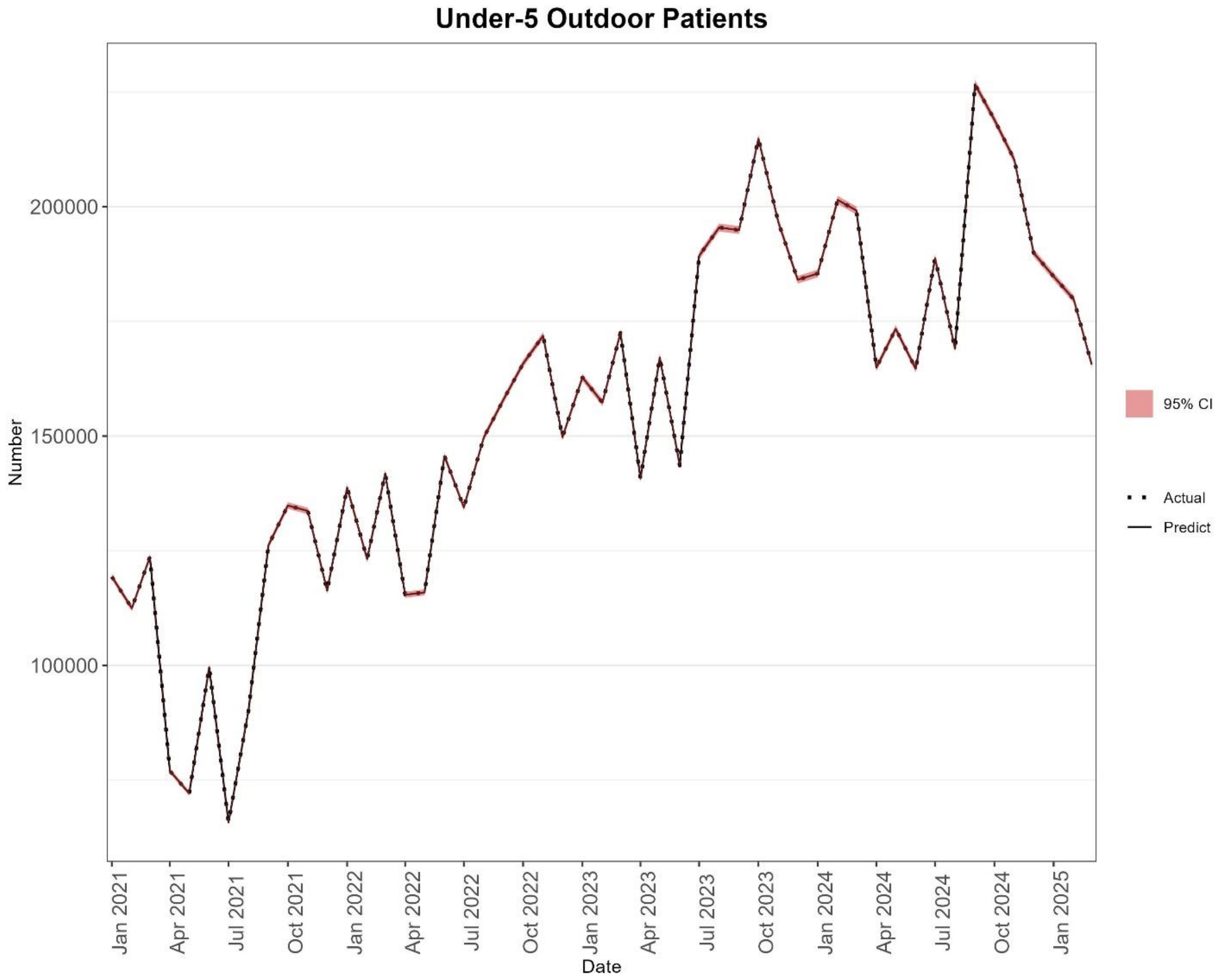
Under-5 Outdoor Patients
200000
150000
100000
Number
Jan 2021
Apr 2021
Jul 2021
Oct 2021
Jan 2022
Apr 2022
Jul 2022
Oct 2022
Jan 2023
Apr 2023
Jul 2023
Oct 2023
Jan 2024
Apr 2024
Jul 2024
Oct 2024
Jan 2025
Date
95% CI
Actual
Predict

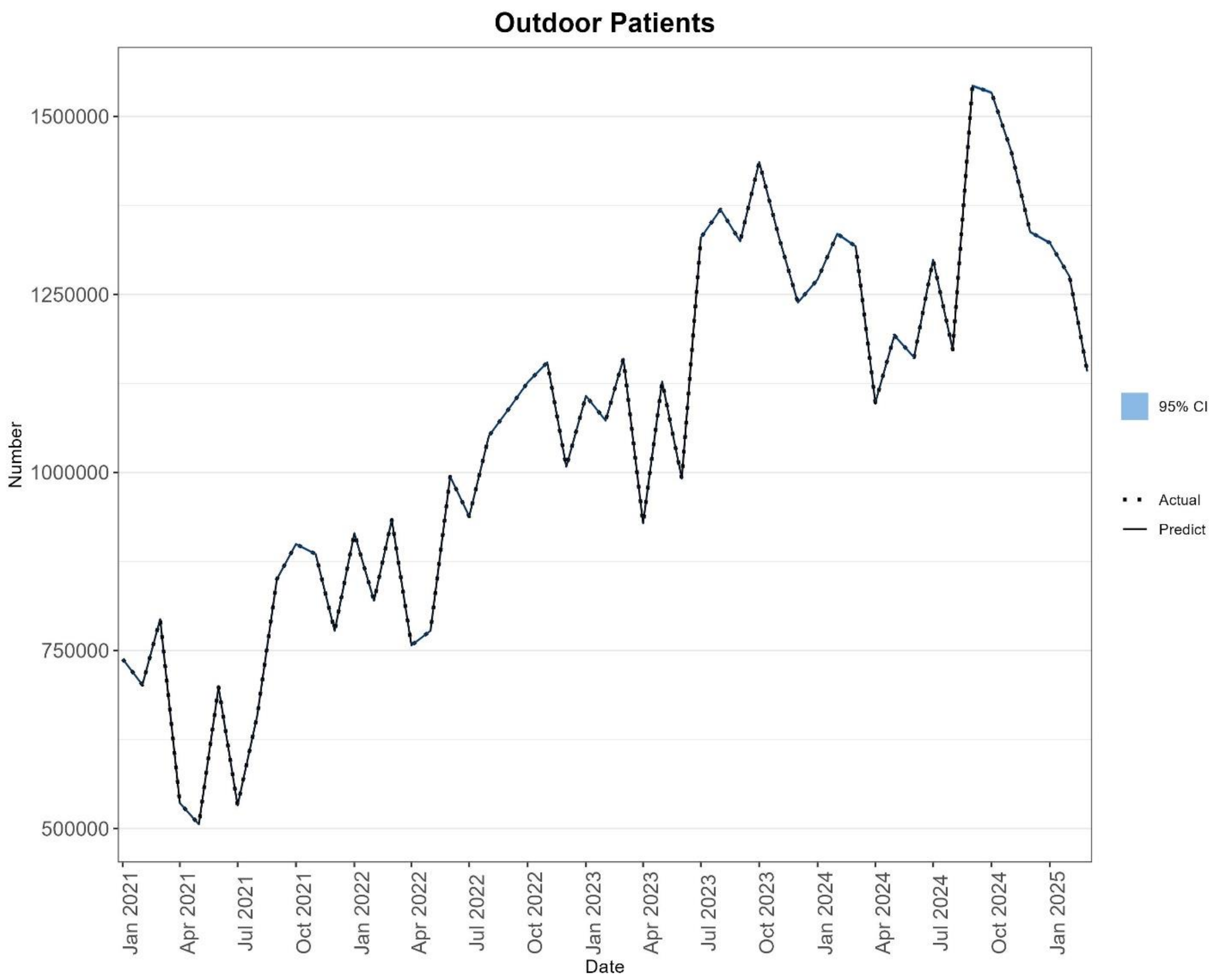

Outdoor Patients
1500000
1250000
1000000
750000
500000
Number
Jan 2021
Apr 2021
Jul 2021
Oct 2021
Jan 2022
Apr 2022
Jul 2022
Oct 2022
Jan 2023
Apr 2023
Jul 2023
Oct 2023
Jan 2024
Apr 2024
Jul 2024
Oct 2024
Jan 2025
Date
95% CI
Actual
Predict

Dhaka

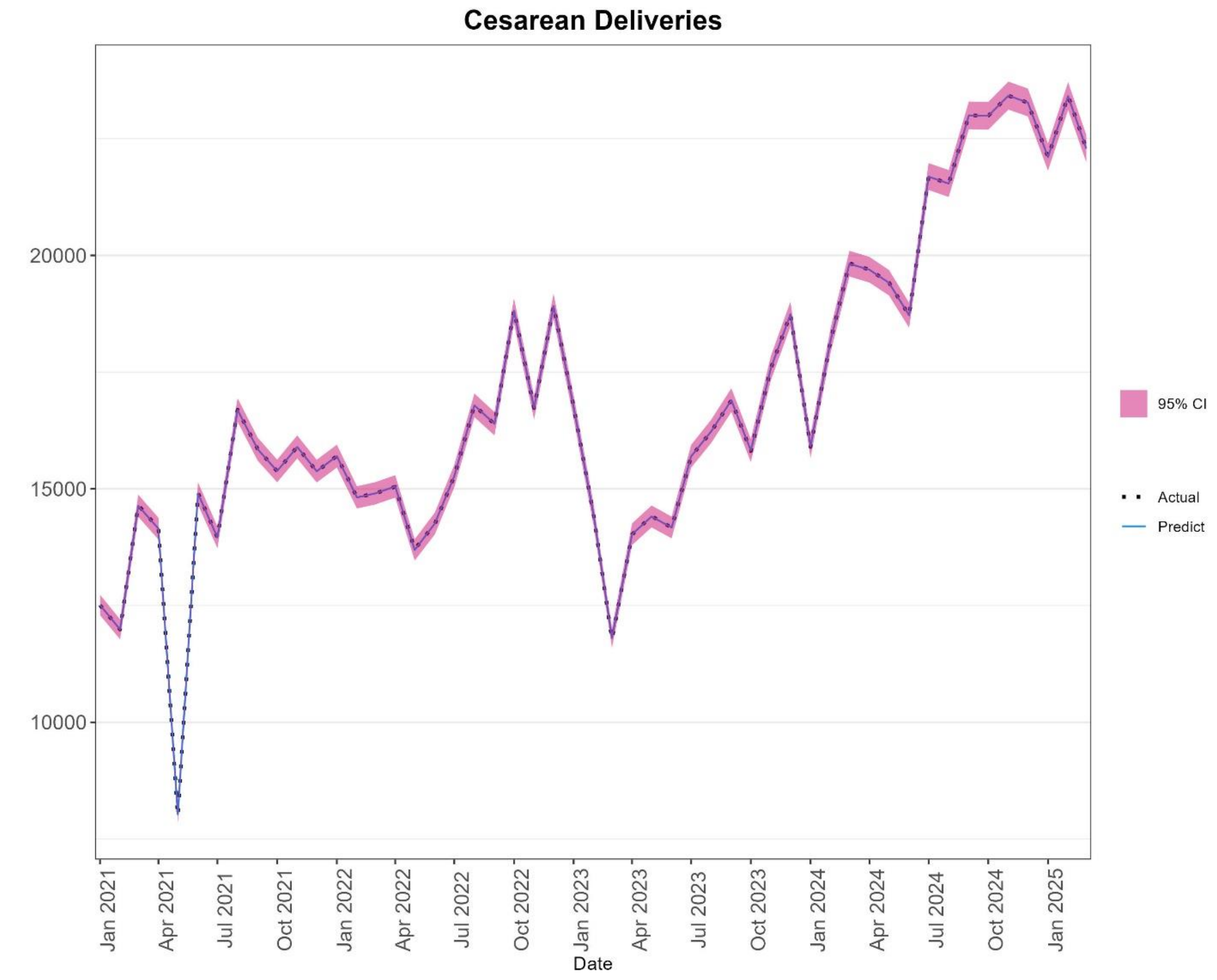

Cesarean Deliveries
20000
15000
10000
Jan 2021
Apr 2021
Jul 2021
Oct 2021
Jan 2022
Apr 2022
Jul 2022
Oct 2022
Jan 2023
Apr 2023
Jul 2023
Oct 2023
Jan 2024
Apr 2024
Jul 2024
Oct 2024
Jan 2025
Date
95% CI
Actual
Predict

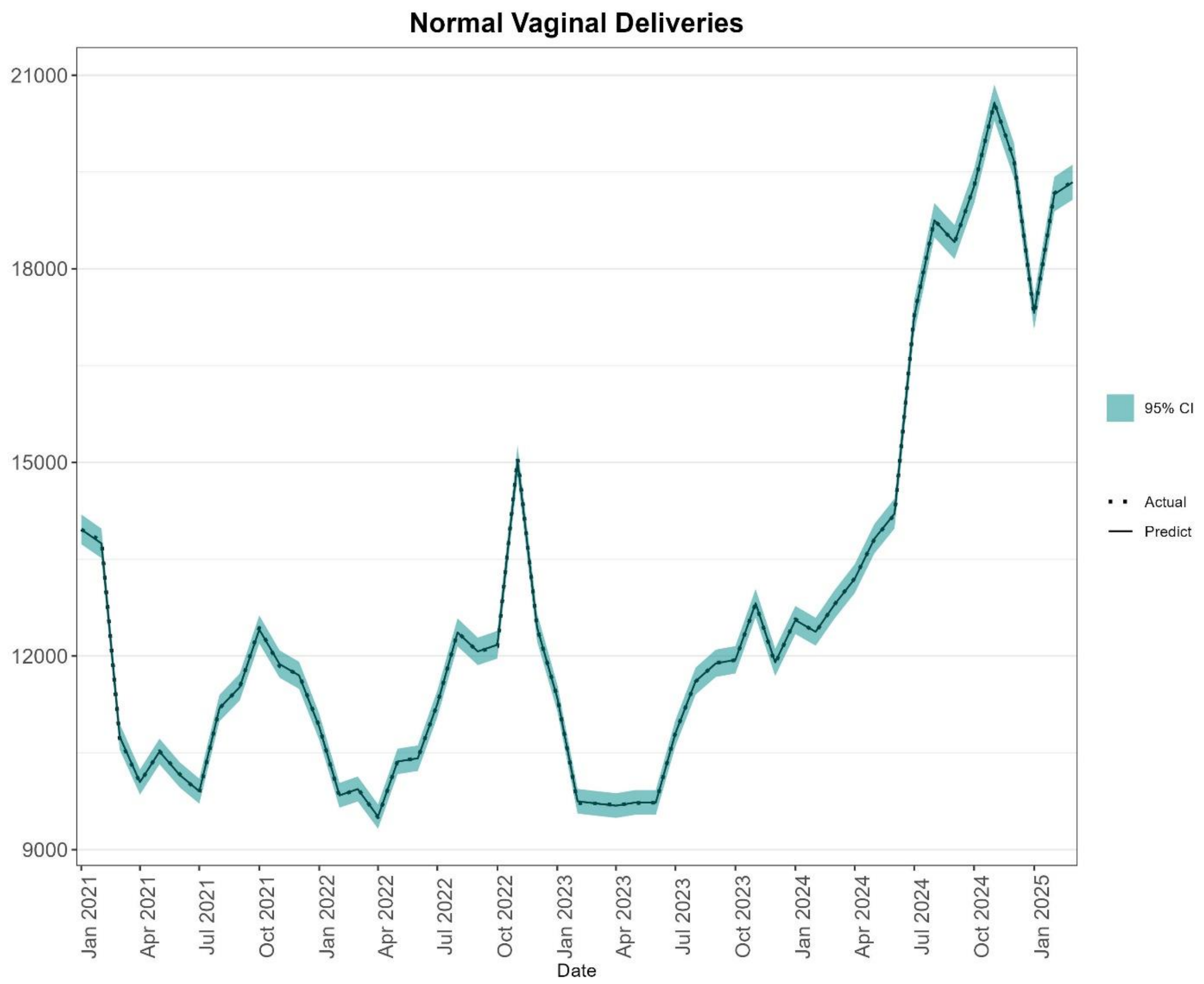
Normal Vaginal Deliveries
21000
18000
15000
12000
9000
Jan 2021
Apr 2021
Jul 2021
Oct 2021
Jan 2022
Apr 2022
Jul 2022
Oct 2022
Jan 2023
Apr 2023
Jul 2023
Oct 2023
Jan 2024
Apr 2024
Jul 2024
Oct 2024
Jan 2025
Date
95% CI
Actual
Predict

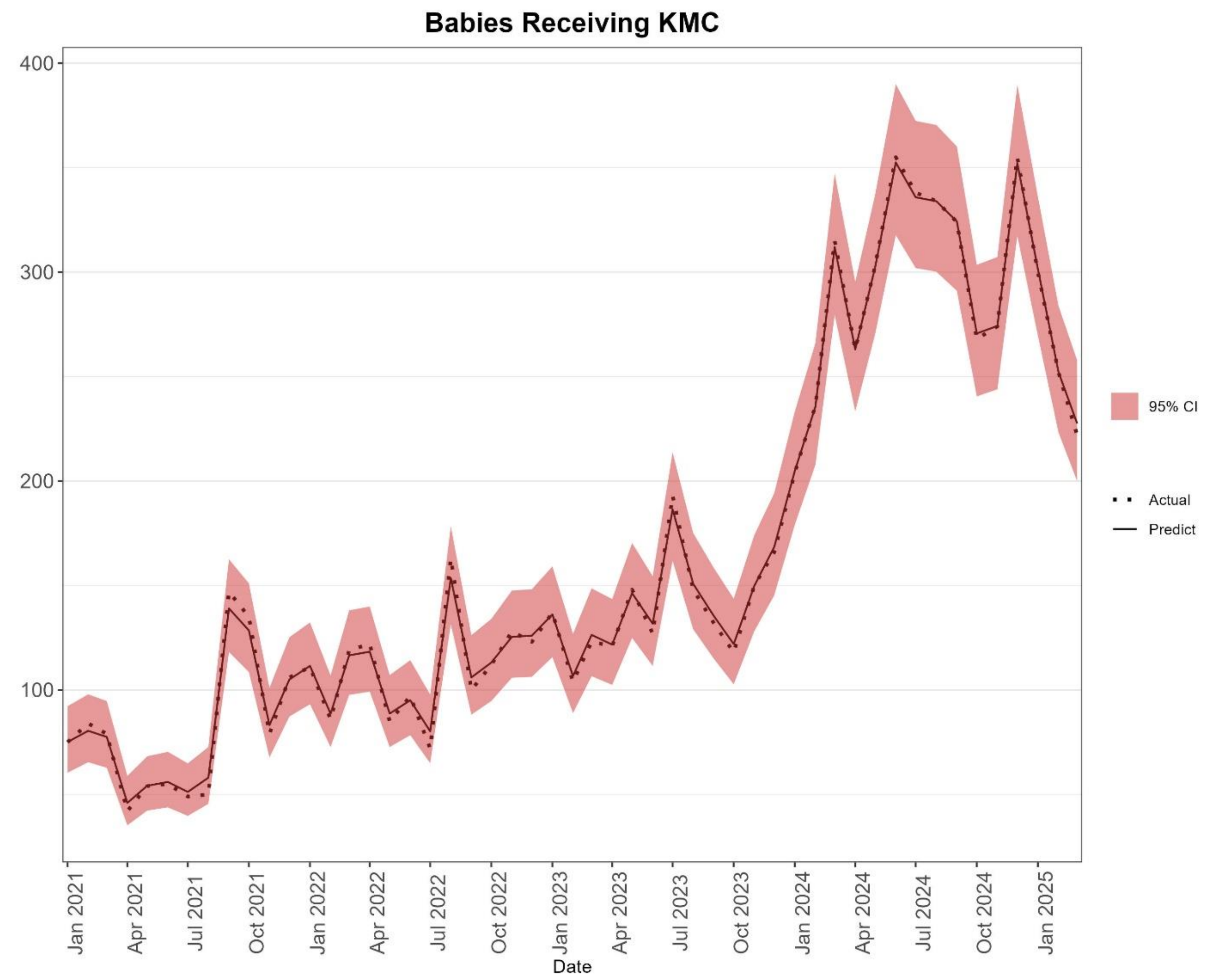

Babies Receiving KMC
400
300
200
100
Jan 2021
Apr 2021
Jul 2021
Oct 2021
Jan 2022
Apr 2022
Jul 2022
Oct 2022
Jan 2023
Apr 2023
Jul 2023
Oct 2023
Jan 2024
Apr 2024
Jul 2024
Oct 2024
Jan 2025
Date
95% CI
Actual
Predict

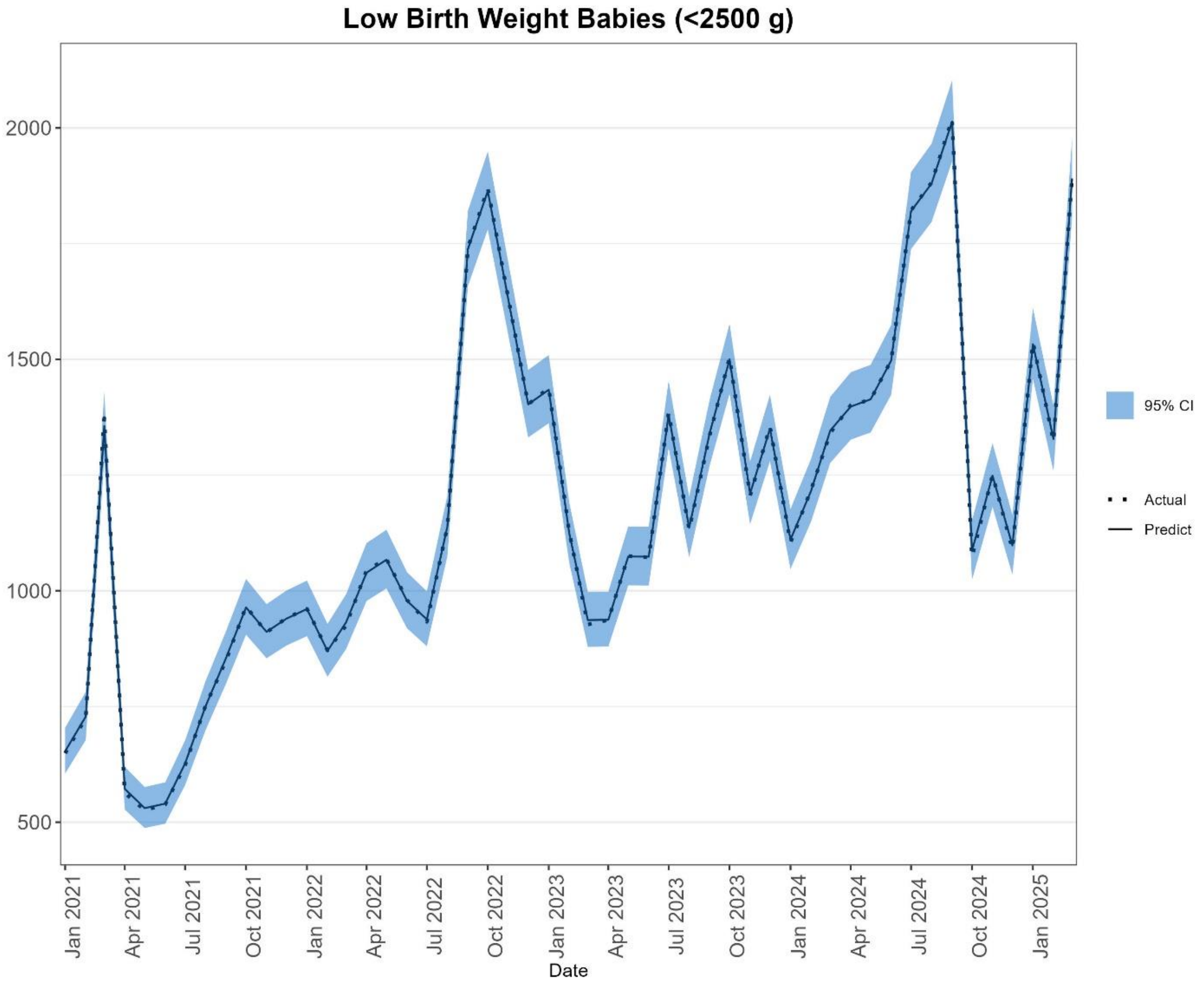

Low Birth Weight Babies (<2500 g)
2000
1500
1000
500
95% CI
Actual
Predict
Jan 2021
Apr 2021
Jul 2021
Oct 2021
Jan 2022
Apr 2022
Jul 2022
Oct 2022
Jan 2023
Apr 2023
Jul 2023
Oct 2023
Jan 2024
Apr 2024
Jul 2024
Oct 2024
Jan 2025
Date

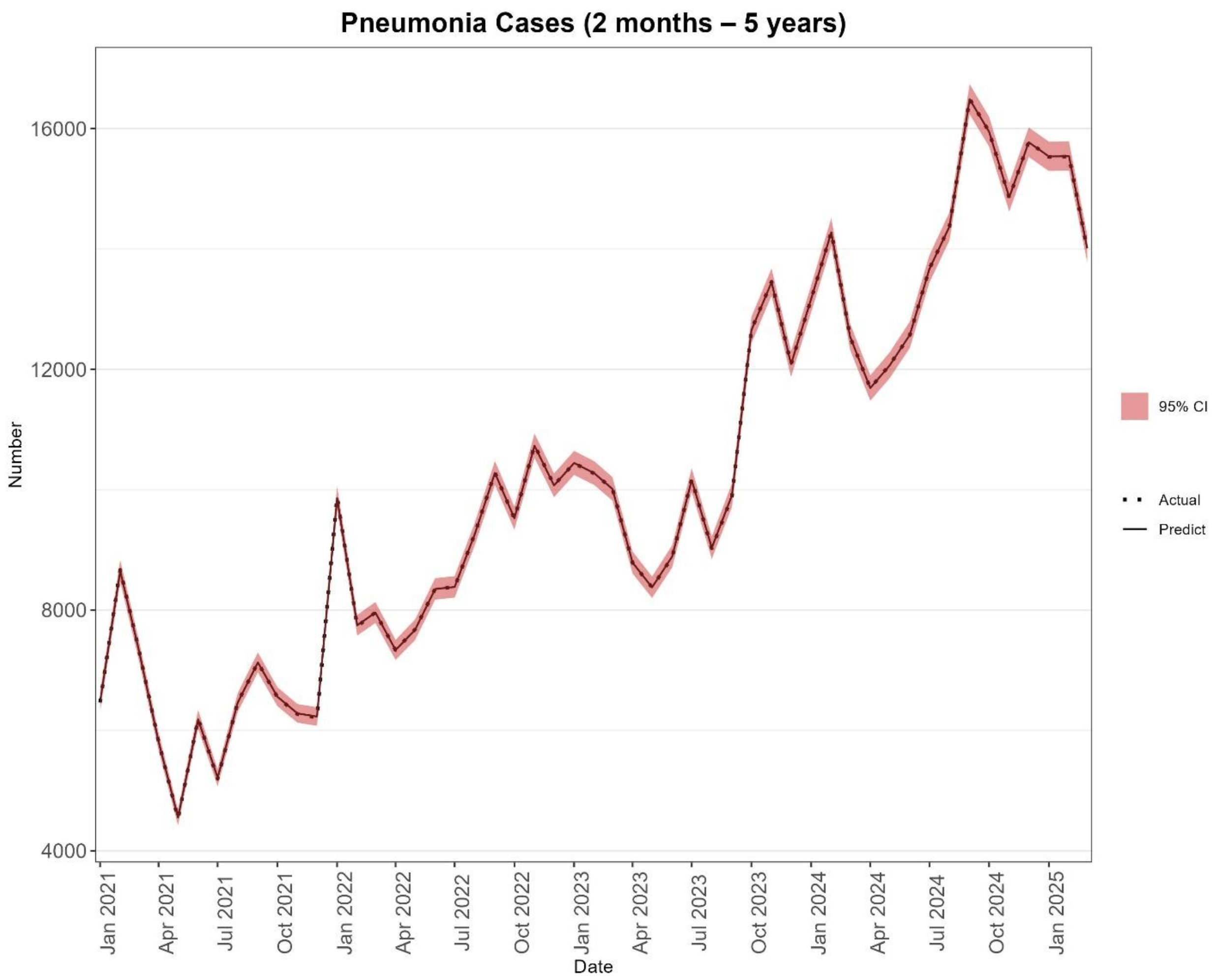

Pneumonia Cases (2 months – 5 years)
Number
16000
12000
8000
4000
Jan 2021
Apr 2021
Jul 2021
Oct 2021
Jan 2022
Apr 2022
Jul 2022
Oct 2022
Jan 2023
Apr 2023
Jul 2023
Oct 2023
Jan 2024
Apr 2024
Jul 2024
Oct 2024
Jan 2025
Date
95% CI
Actual
Predict

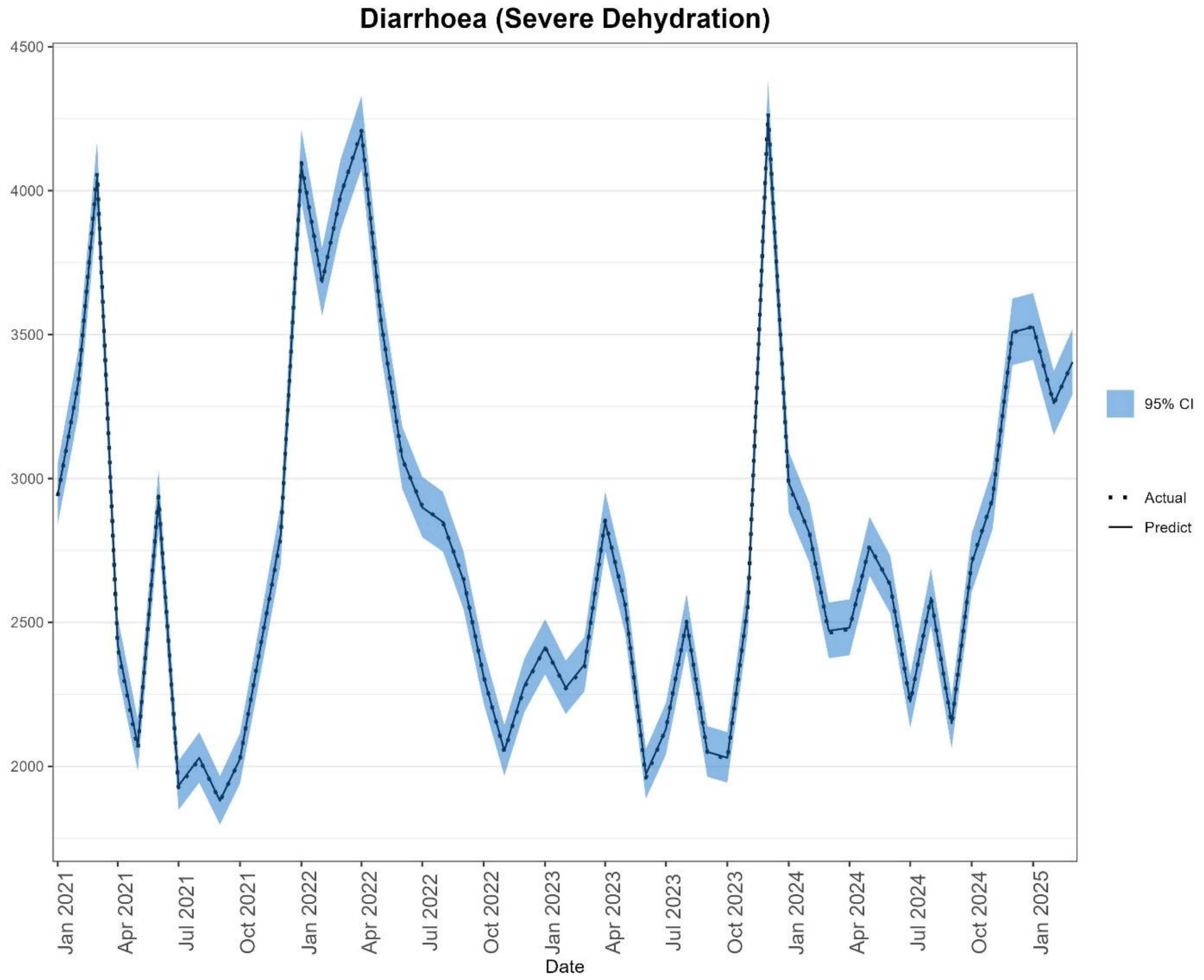
Diarrhoea (Severe Dehydration)
4500
4000
3500
3000
2500
2000
Jan 2021
Apr 2021
Jul 2021
Oct 2021
Jan 2022
Apr 2022
Jul 2022
Oct 2022
Jan 2023
Apr 2023
Jul 2023
Oct 2023
Jan 2024
Apr 2024
Jul 2024
Oct 2024
Jan 2025
Date
95% CI
Actual
Predict

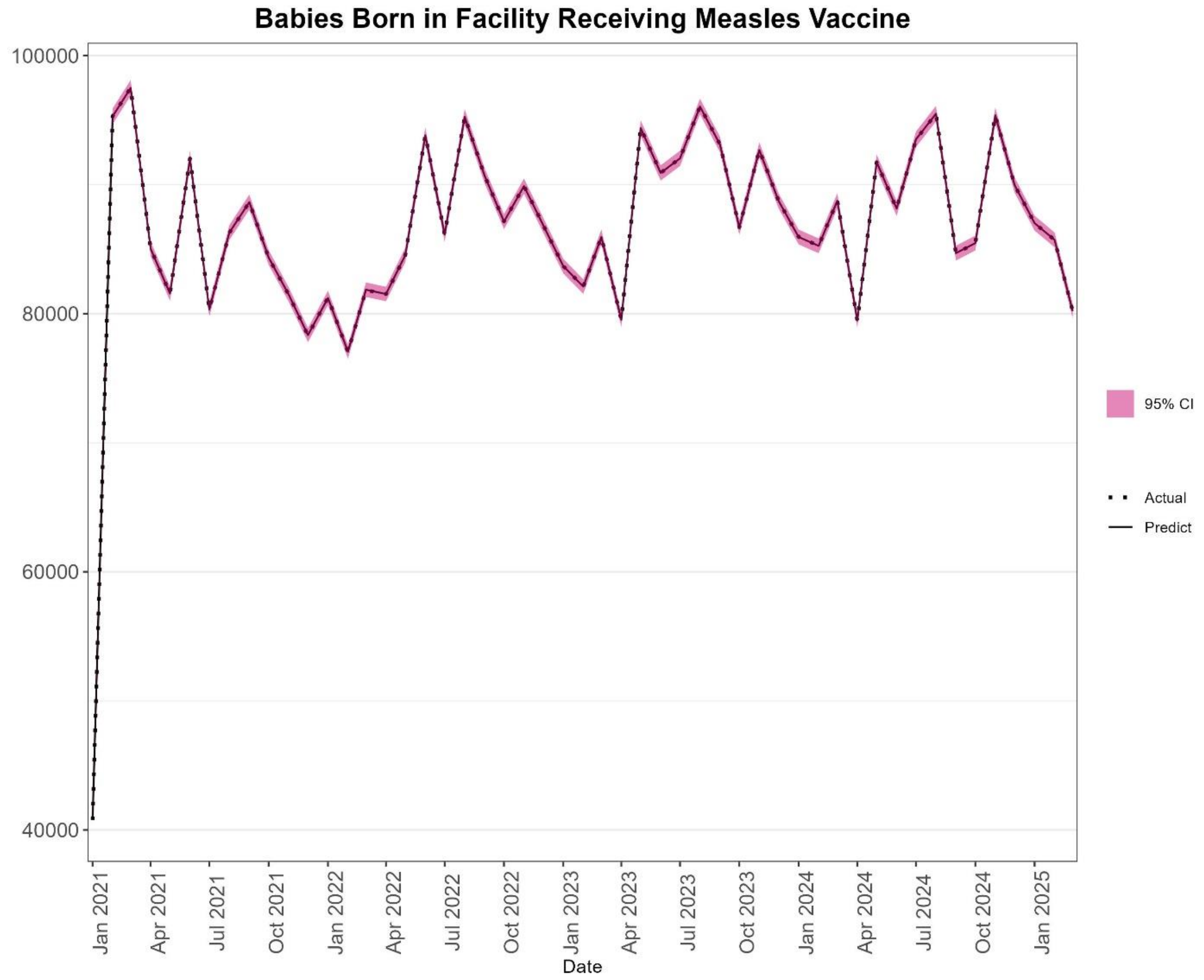
Babies Born in Facility Receiving Measles Vaccine
100000
80000
60000
40000
Jan 2021
Apr 2021
Jul 2021
Oct 2021
Jan 2022
Apr 2022
Jul 2022
Oct 2022
Jan 2023
Apr 2023
Jul 2023
Oct 2023
Jan 2024
Apr 2024
Jul 2024
Oct 2024
Jan 2025
Date
95% CI
Actual
Predict

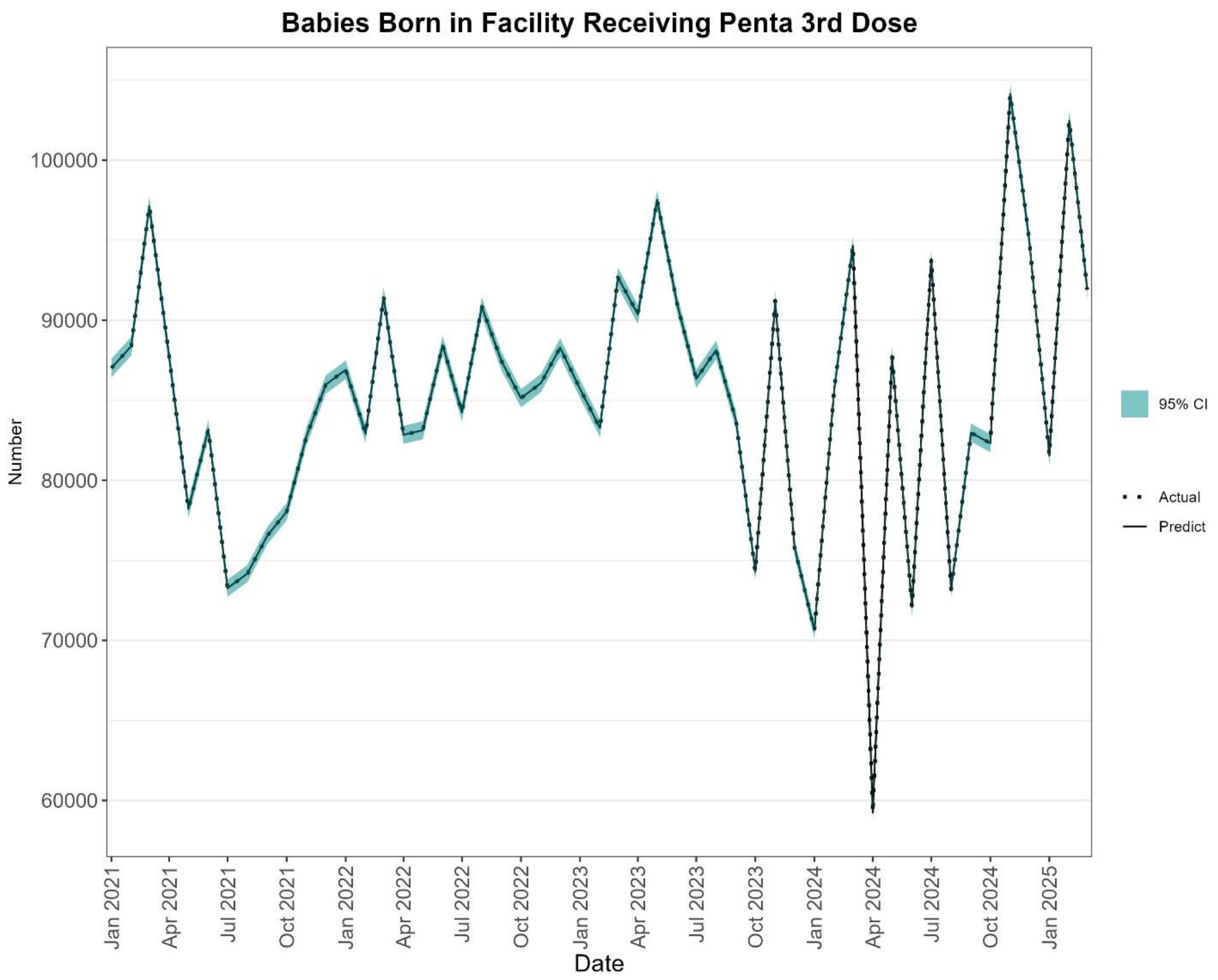
Babies Born in Facility Receiving Penta 3rd Dose
Number
100000
90000
80000
70000
60000
Date
Jan 2021
Apr 2021
Jul 2021
Oct 2021
Jan 2022
Apr 2022
Jul 2022
Oct 2022
Jan 2023
Apr 2023
Jul 2023
Oct 2023
Jan 2024
Apr 2024
Jul 2024
Oct 2024
Jan 2025
95% CI
Actual
Predict

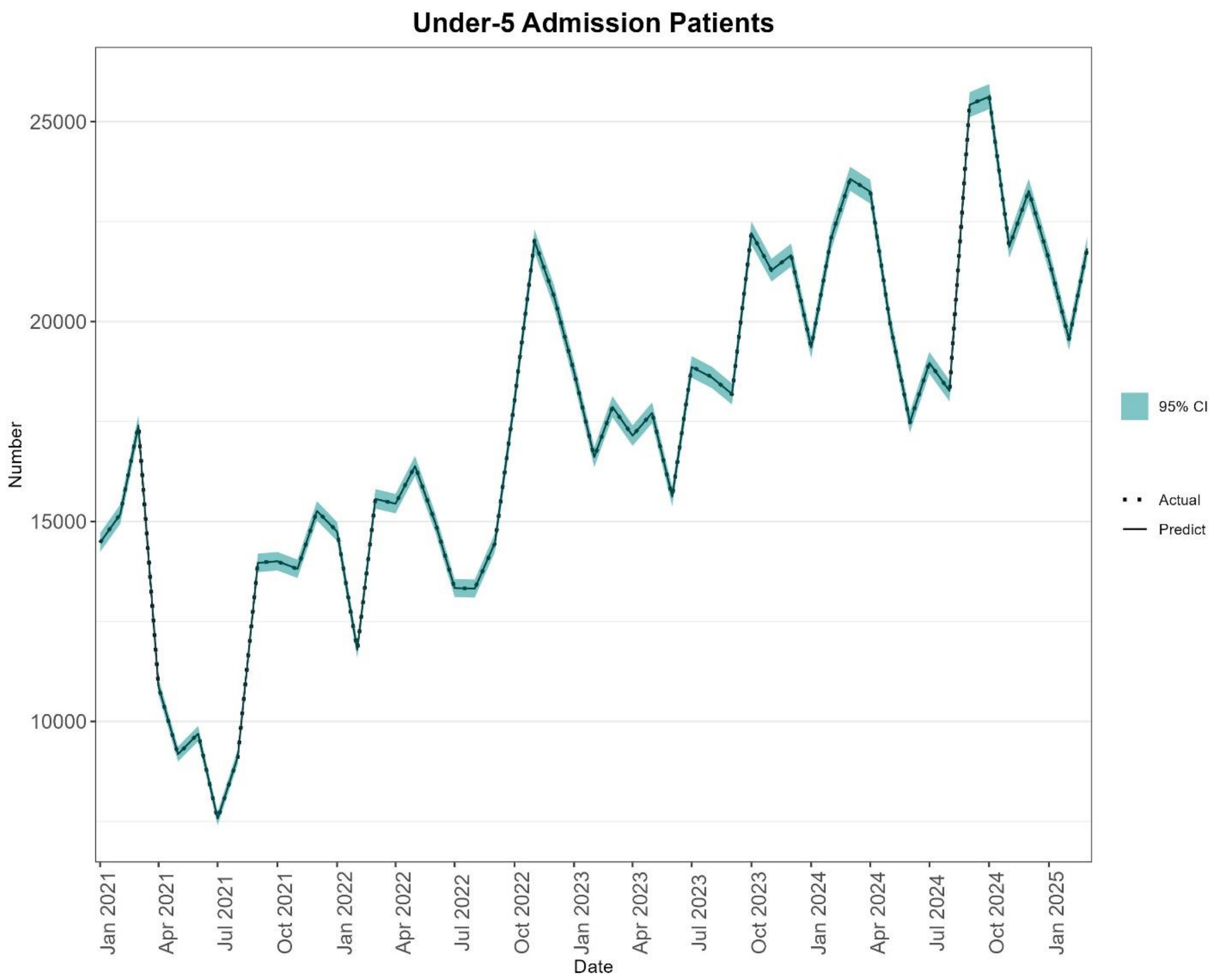

Under-5 Admission Patients
Number
25000
20000
15000
10000
Date
Jan 2021
Apr 2021
Jul 2021
Oct 2021
Jan 2022
Apr 2022
Jul 2022
Oct 2022
Jan 2023
Apr 2023
Jul 2023
Oct 2023
Jan 2024
Apr 2024
Jul 2024
Oct 2024
Jan 2025
95% CI
Actual
Predict

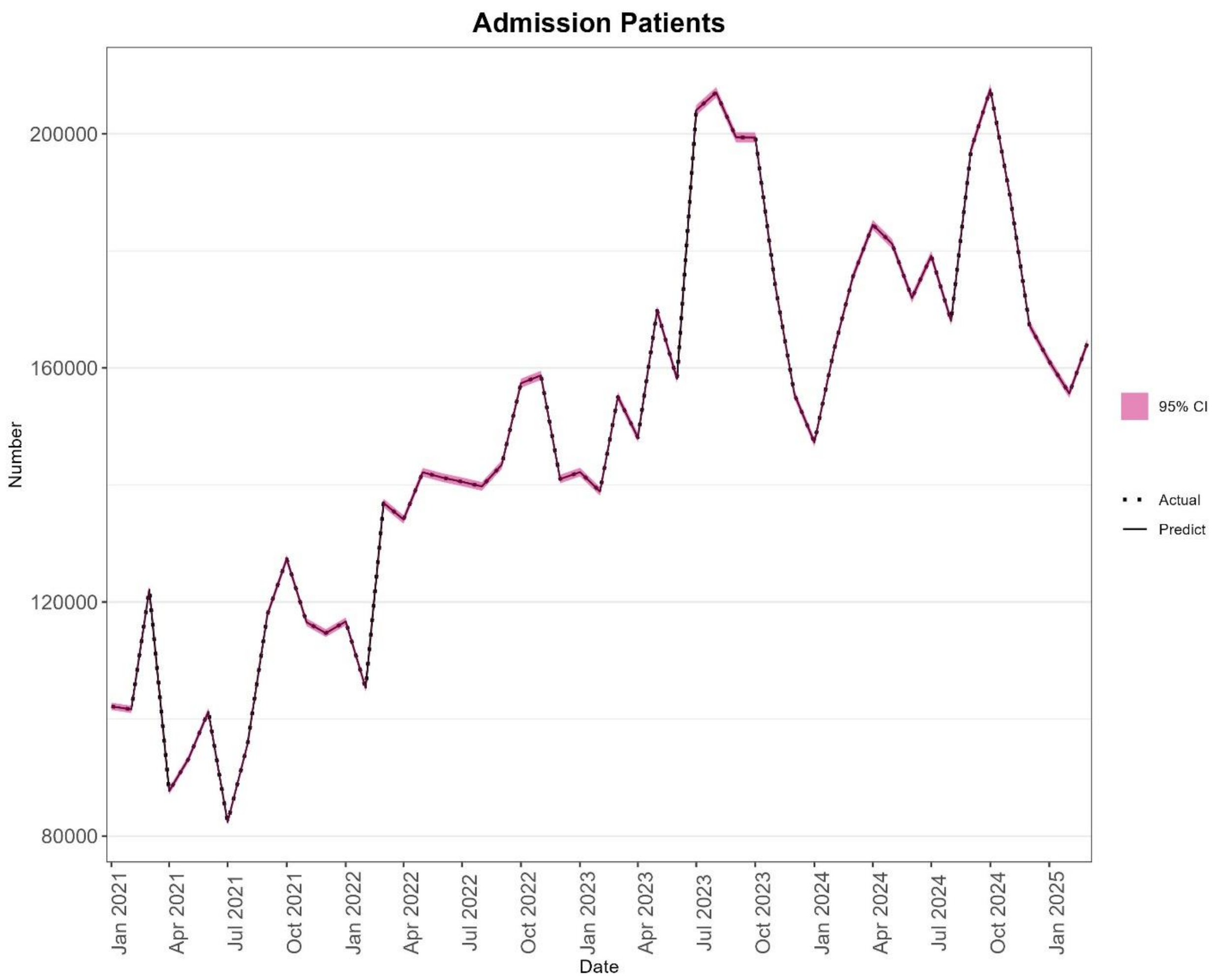

Admission Patients
Number
200000
160000
120000
80000
Date
Jan 2021
Apr 2021
Jul 2021
Oct 2021
Jan 2022
Apr 2022
Jul 2022
Oct 2022
Jan 2023
Apr 2023
Jul 2023
Oct 2023
Jan 2024
Apr 2024
Jul 2024
Oct 2024
Jan 2025
95% CI
Actual
Predict

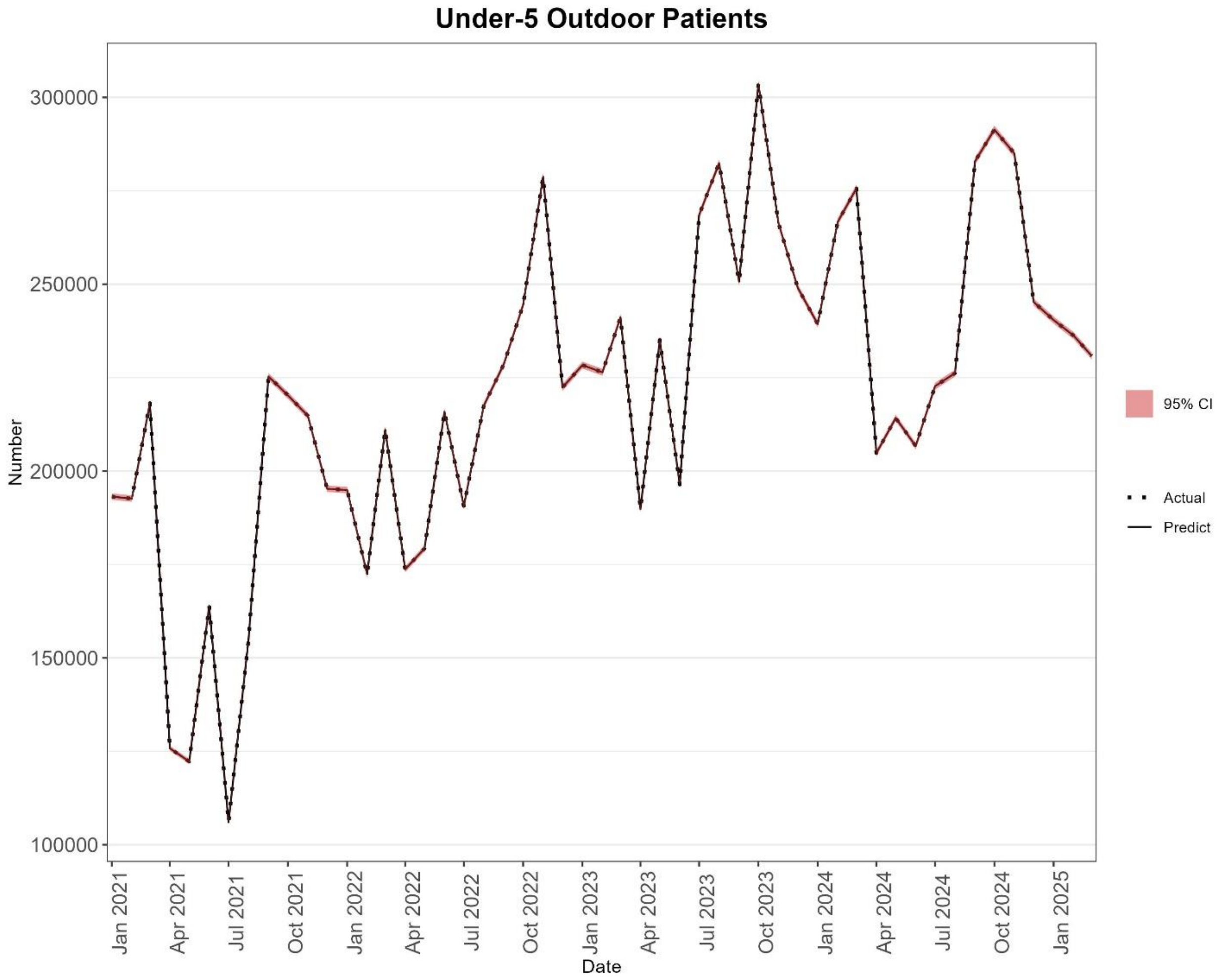

Under-5 Outdoor Patients
300000
250000
200000
150000
100000
Number
Jan 2021
Apr 2021
Jul 2021
Oct 2021
Jan 2022
Apr 2022
Jul 2022
Oct 2022
Jan 2023
Apr 2023
Jul 2023
Oct 2023
Jan 2024
Apr 2024
Jul 2024
Oct 2024
Jan 2025
Date
95% CI
Actual
Predict

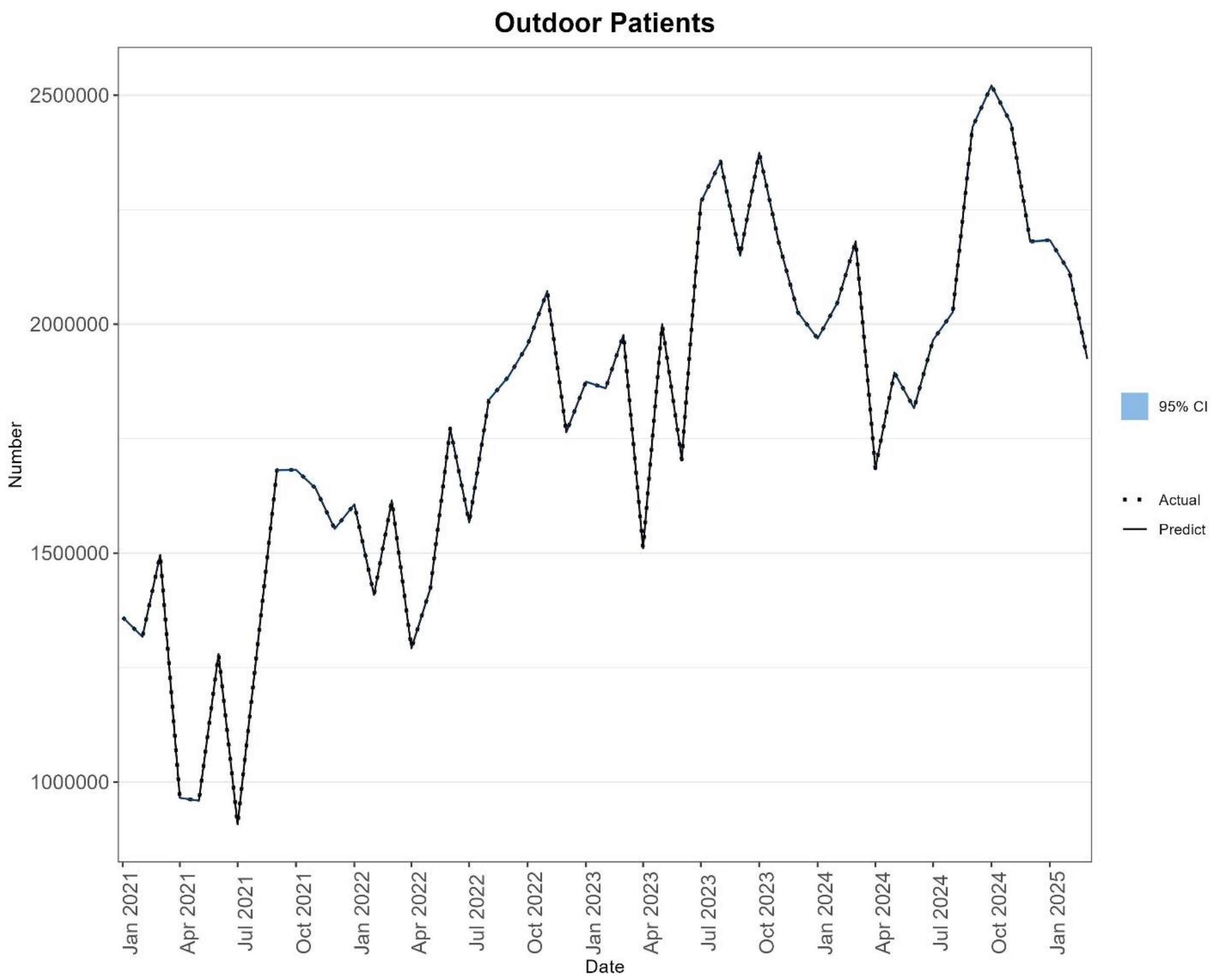

Outdoor Patients
2500000
2000000
1500000
1000000
Number
Jan 2021
Apr 2021
Jul 2021
Oct 2021
Jan 2022
Apr 2022
Jul 2022
Oct 2022
Jan 2023
Apr 2023
Jul 2023
Oct 2023
Jan 2024
Apr 2024
Jul 2024
Oct 2024
Jan 2025
Date
95% CI
Actual
Predict

Khulna

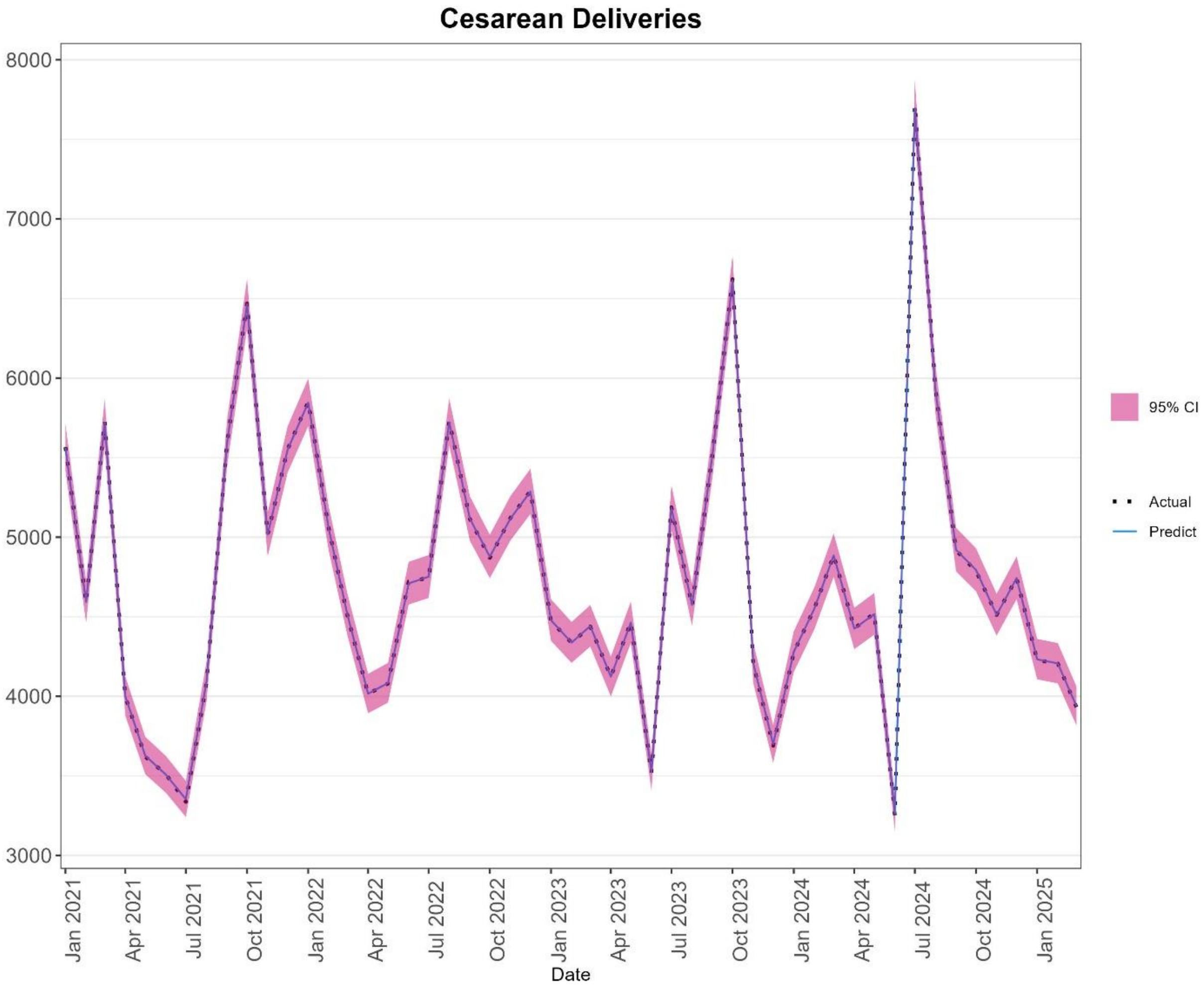

Cesarean Deliveries
8000
7000
6000
5000
4000
3000
Jan 2021
Apr 2021
Jul 2021
Oct 2021
Jan 2022
Apr 2022
Jul 2022
Oct 2022
Jan 2023
Apr 2023
Jul 2023
Oct 2023
Jan 2024
Apr 2024
Jul 2024
Oct 2024
Jan 2025
Date
95% CI
Actual
Predict

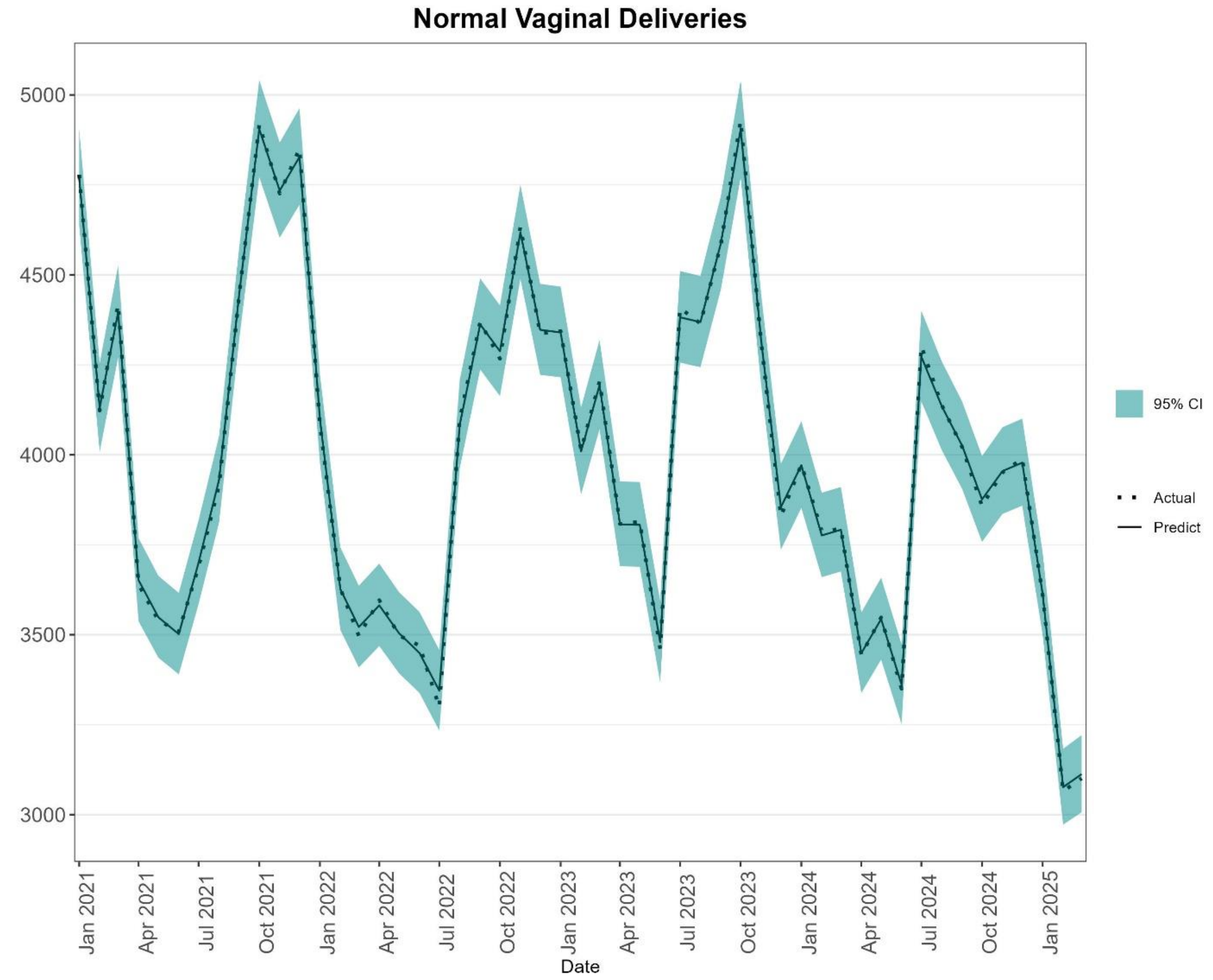
Normal Vaginal Deliveries
5000
4500
4000
3500
3000
Jan 2021
Apr 2021
Jul 2021
Oct 2021
Jan 2022
Apr 2022
Jul 2022
Oct 2022
Jan 2023
Apr 2023
Jul 2023
Oct 2023
Jan 2024
Apr 2024
Jul 2024
Oct 2024
Jan 2025
Date
95% CI
Actual
Predict

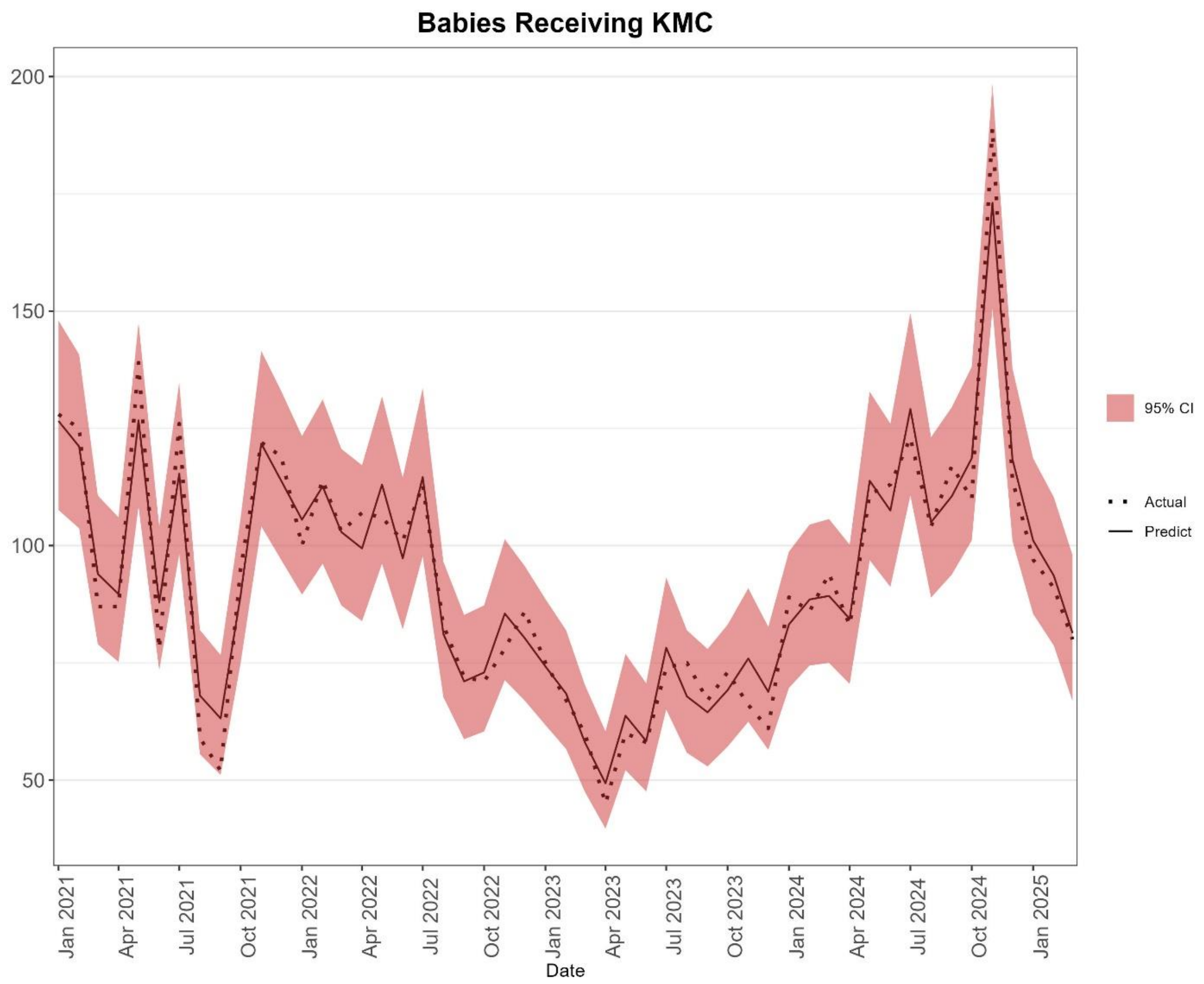

Babies Receiving KMC
200
150
100
50
95% CI
Actual
Predict
Jan 2021
Apr 2021
Jul 2021
Oct 2021
Jan 2022
Apr 2022
Jul 2022
Oct 2022
Jan 2023
Apr 2023
Jul 2023
Oct 2023
Jan 2024
Apr 2024
Jul 2024
Oct 2024
Jan 2025
Date

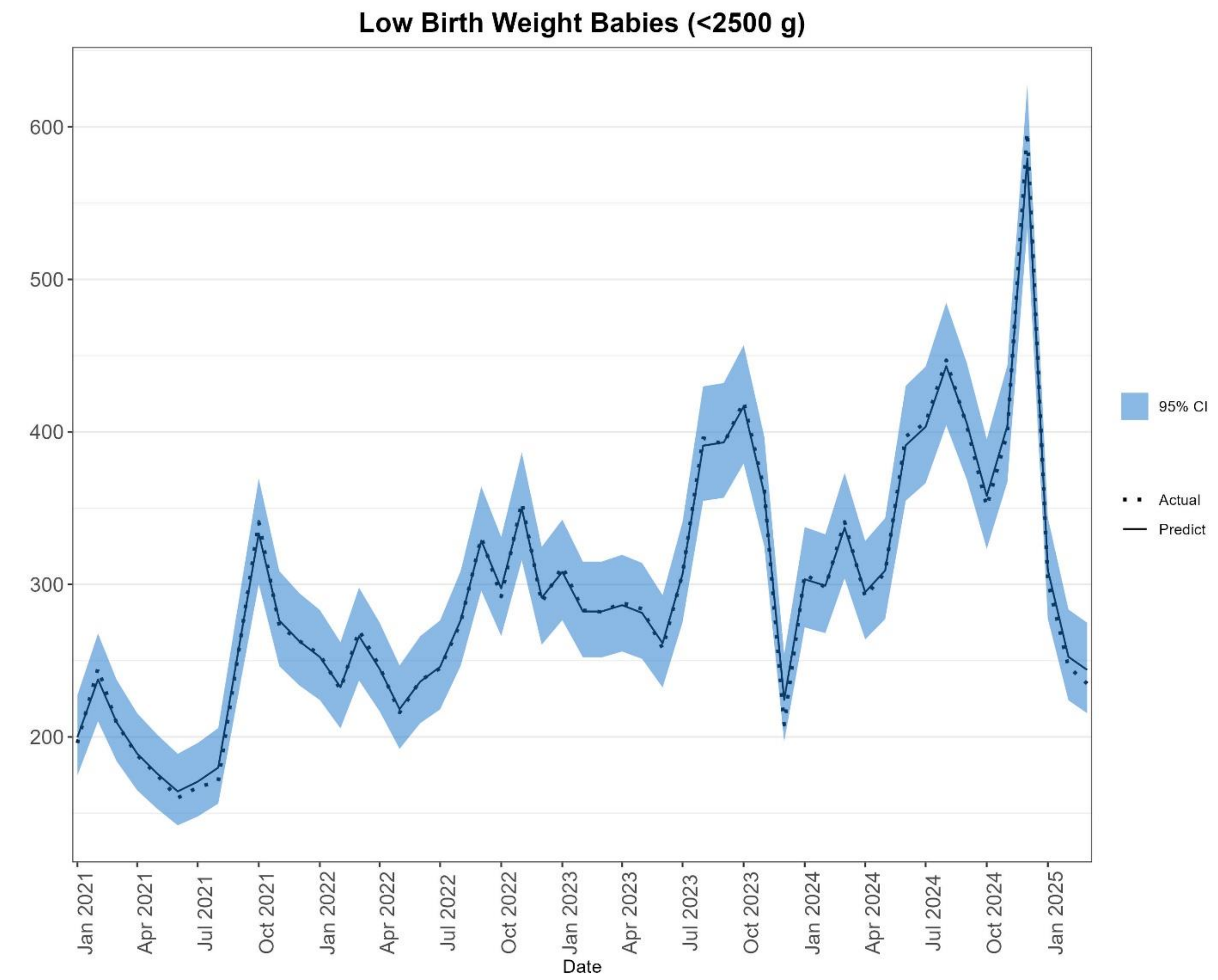

Low Birth Weight Babies (<2500 g)
600
500
400
300
200
Jan 2021
Apr 2021
Jul 2021
Oct 2021
Jan 2022
Apr 2022
Jul 2022
Oct 2022
Jan 2023
Apr 2023
Jul 2023
Oct 2023
Jan 2024
Apr 2024
Jul 2024
Oct 2024
Jan 2025
Date
95% CI
Actual
Predict

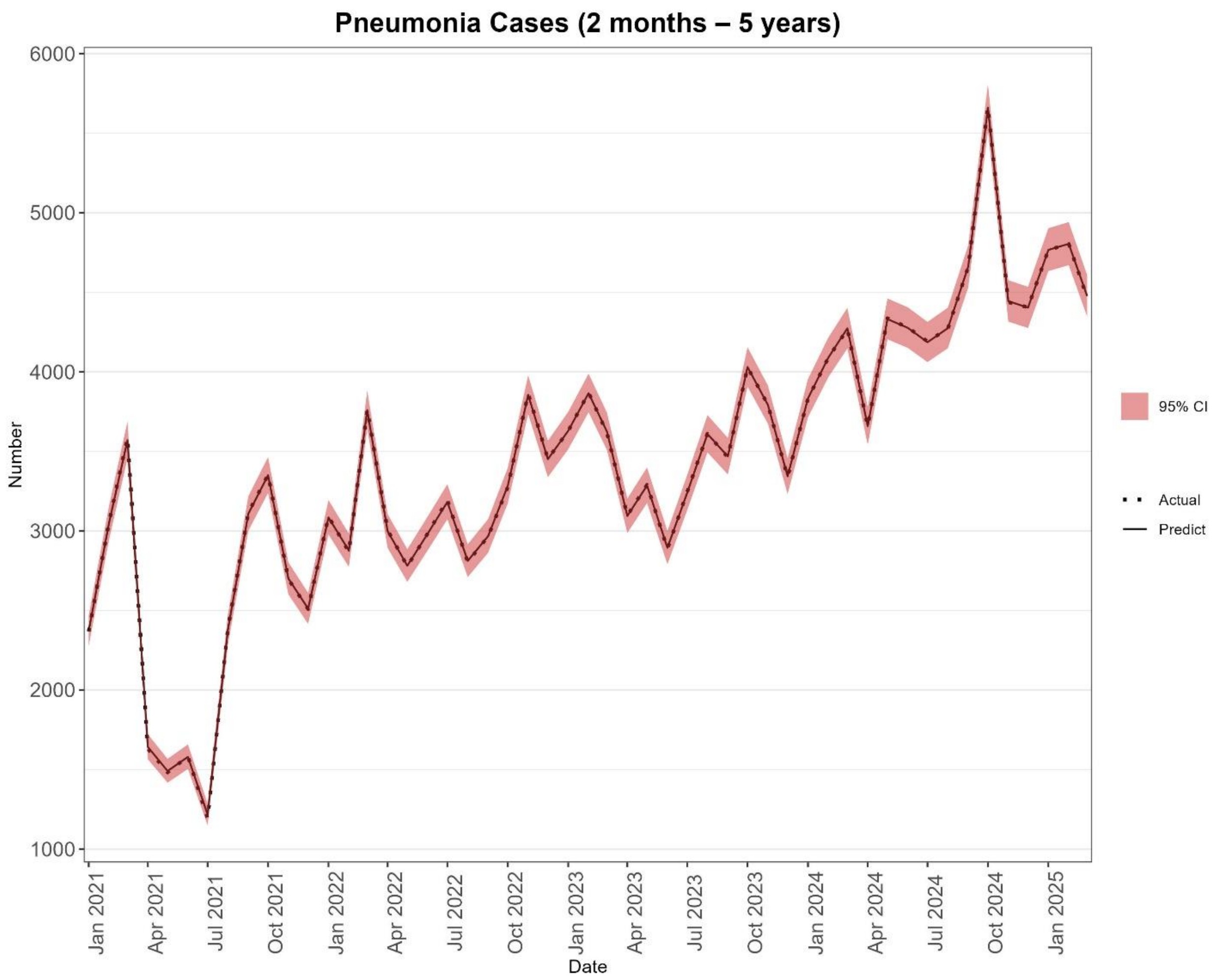
Pneumonia Cases (2 months – 5 years)
6000
5000
4000
3000
2000
1000
Number
Jan 2021
Apr 2021
Jul 2021
Oct 2021
Jan 2022
Apr 2022
Jul 2022
Oct 2022
Jan 2023
Apr 2023
Jul 2023
Oct 2023
Jan 2024
Apr 2024
Jul 2024
Oct 2024
Jan 2025
Date
95% CI
Actual
Predict

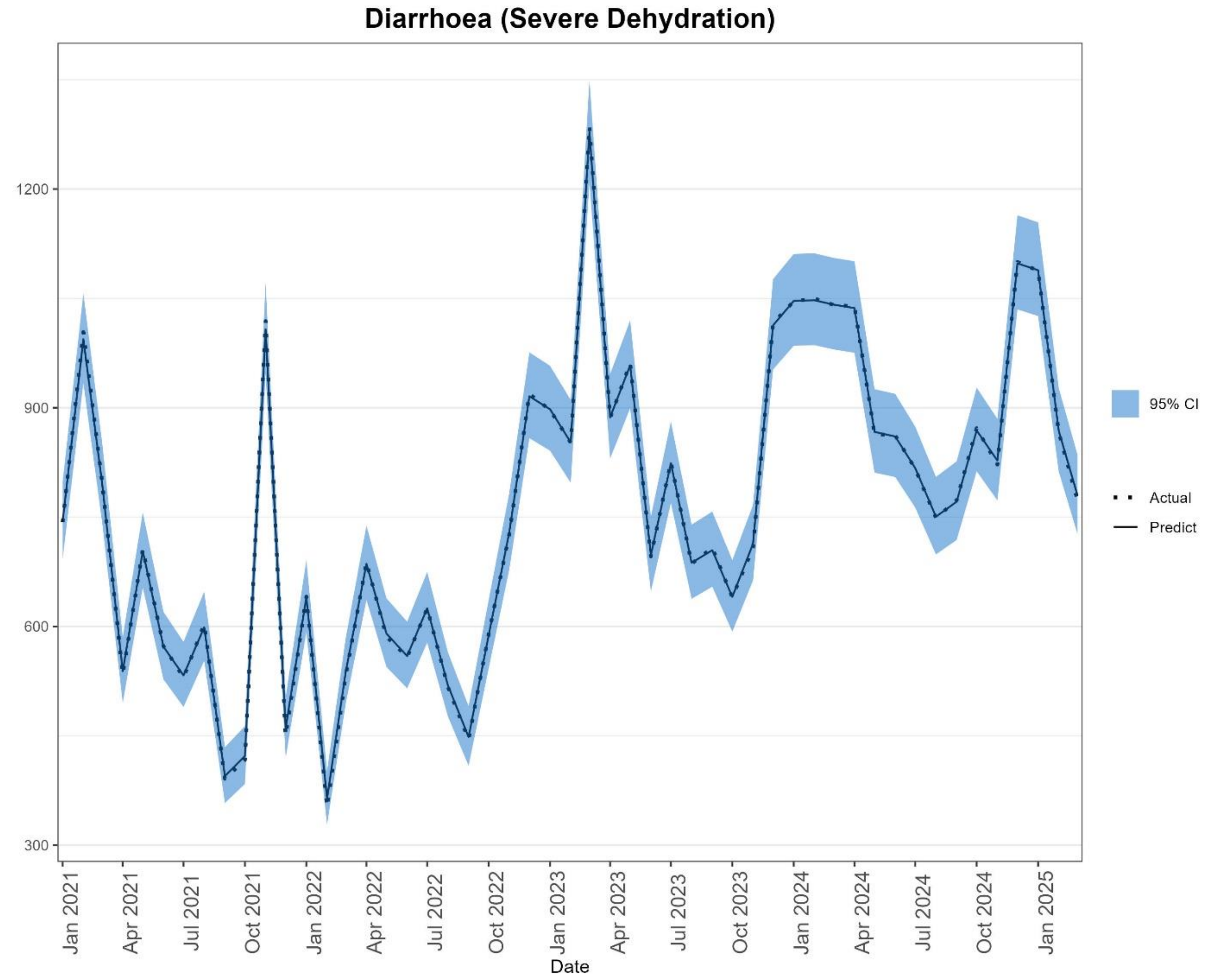

Diarrhoea (Severe Dehydration)
1200
900
600
300
Jan 2021
Apr 2021
Jul 2021
Oct 2021
Jan 2022
Apr 2022
Jul 2022
Oct 2022
Jan 2023
Apr 2023
Jul 2023
Oct 2023
Jan 2024
Apr 2024
Jul 2024
Oct 2024
Jan 2025
Date
95% CI
Actual
Predict

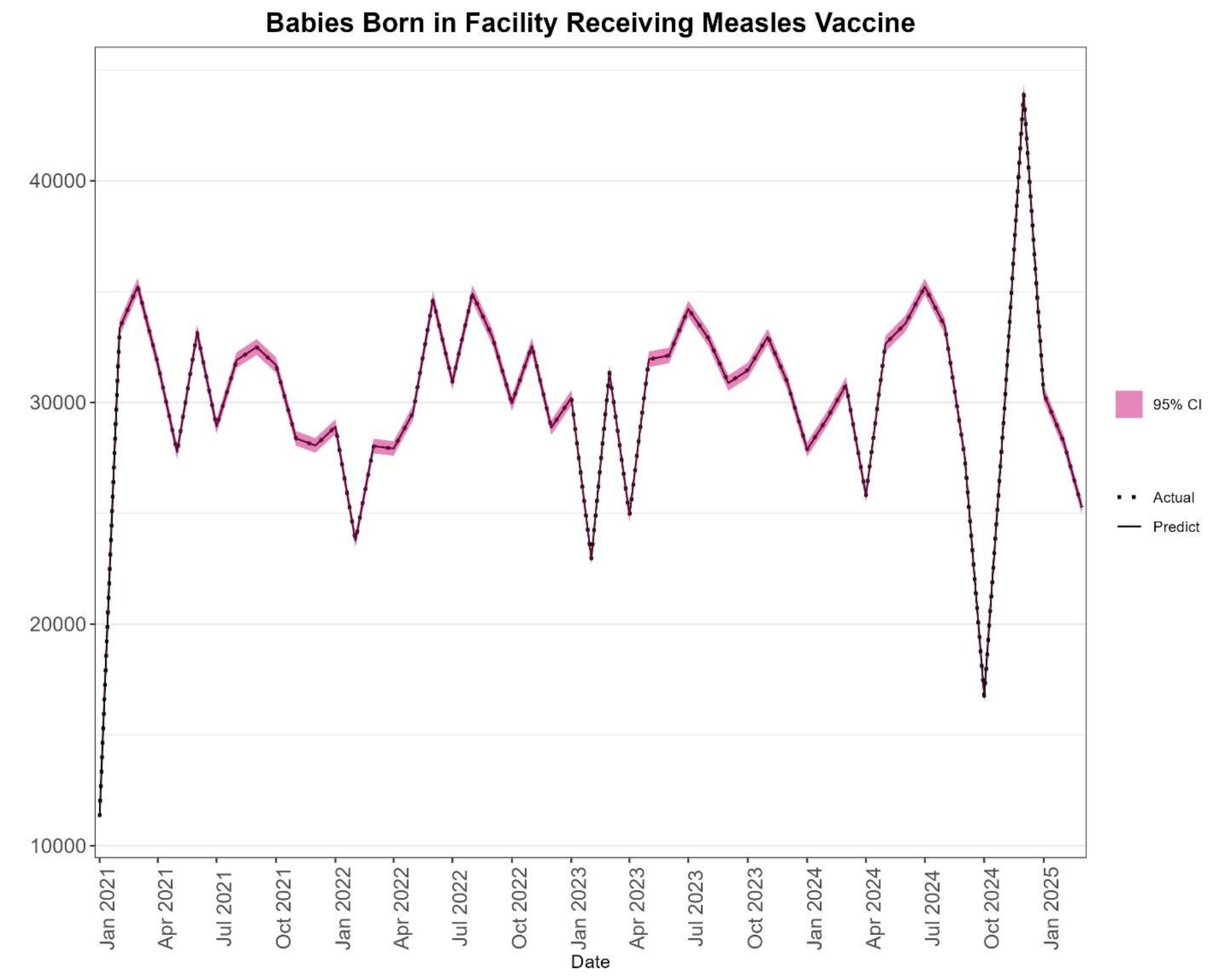

Babies Born in Facility Receiving Measles Vaccine
40000
30000
20000
10000
Jan 2021
Apr 2021
Jul 2021
Oct 2021
Jan 2022
Apr 2022
Jul 2022
Oct 2022
Jan 2023
Apr 2023
Jul 2023
Oct 2023
Jan 2024
Apr 2024
Jul 2024
Oct 2024
Jan 2025
Date
95% CI
Actual
Predict

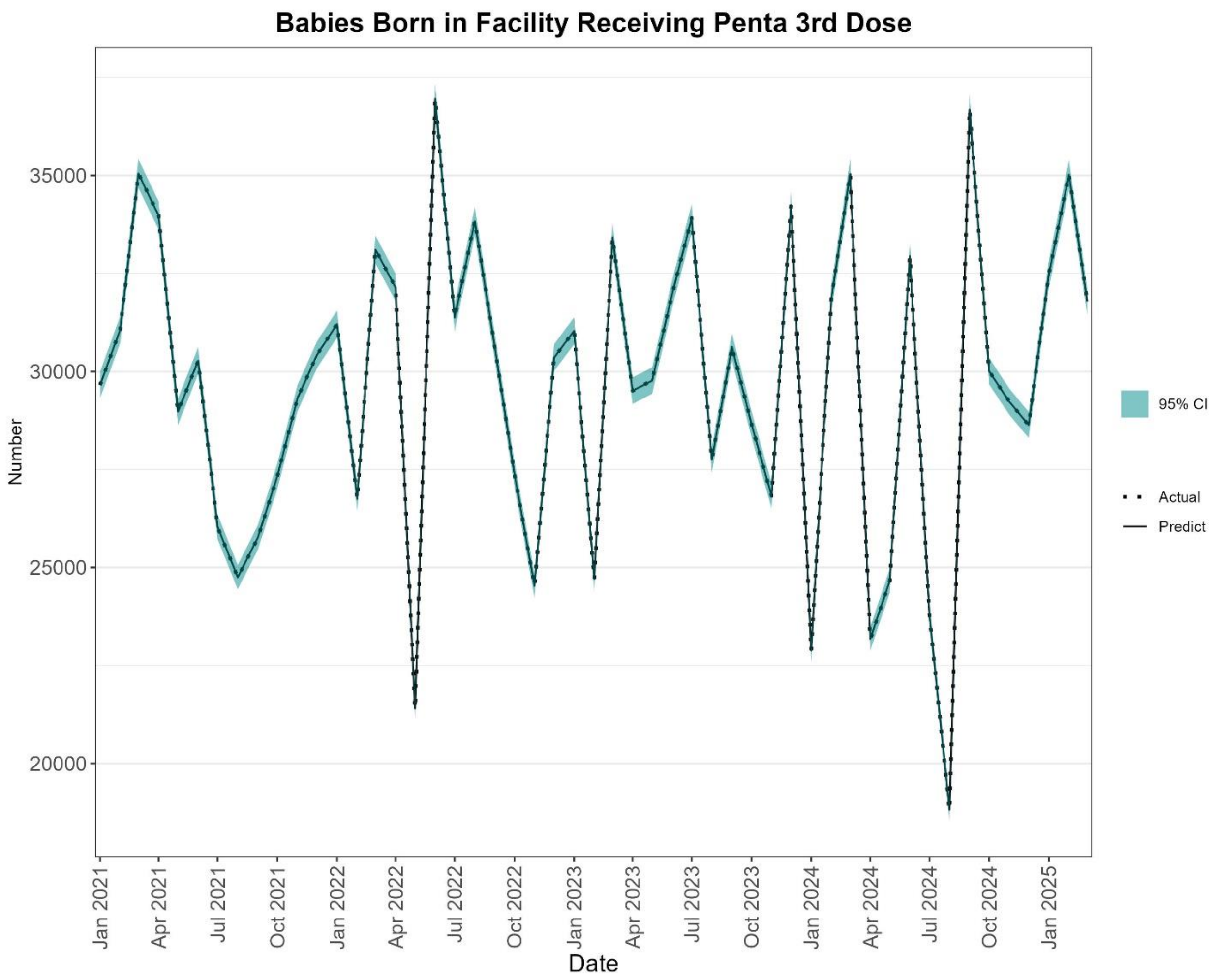
Babies Born in Facility Receiving Penta 3rd Dose
Number
35000
30000
25000
20000
Jan 2021
Apr 2021
Jul 2021
Oct 2021
Jan 2022
Apr 2022
Jul 2022
Oct 2022
Jan 2023
Apr 2023
Jul 2023
Oct 2023
Jan 2024
Apr 2024
Jul 2024
Oct 2024
Jan 2025
Date
95% CI
Actual
Predict

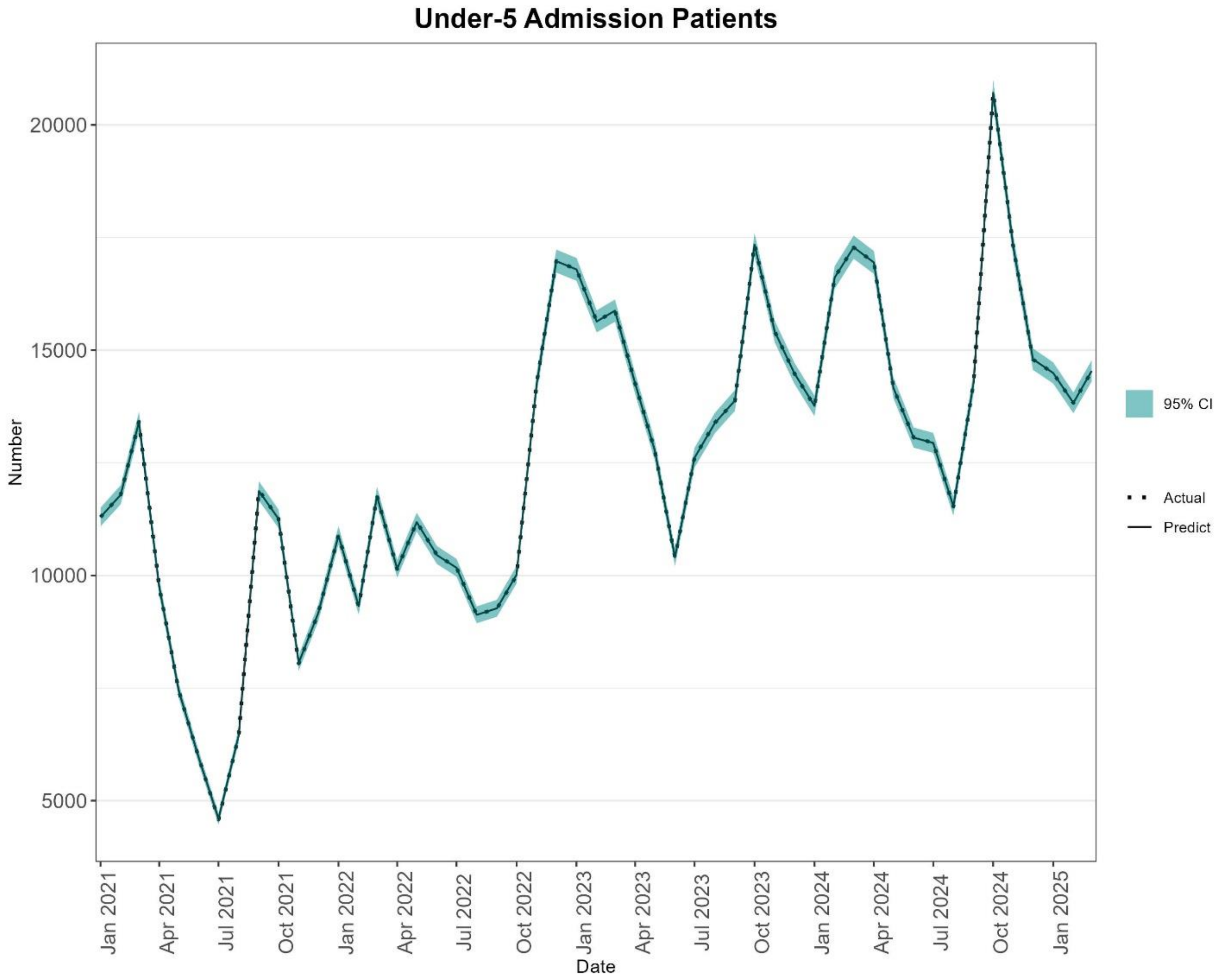

Under-5 Admission Patients
20000
15000
10000
5000
Number
Jan 2021
Apr 2021
Jul 2021
Oct 2021
Jan 2022
Apr 2022
Jul 2022
Oct 2022
Jan 2023
Apr 2023
Jul 2023
Oct 2023
Jan 2024
Apr 2024
Jul 2024
Oct 2024
Jan 2025
Date
95% CI
Actual
Predict

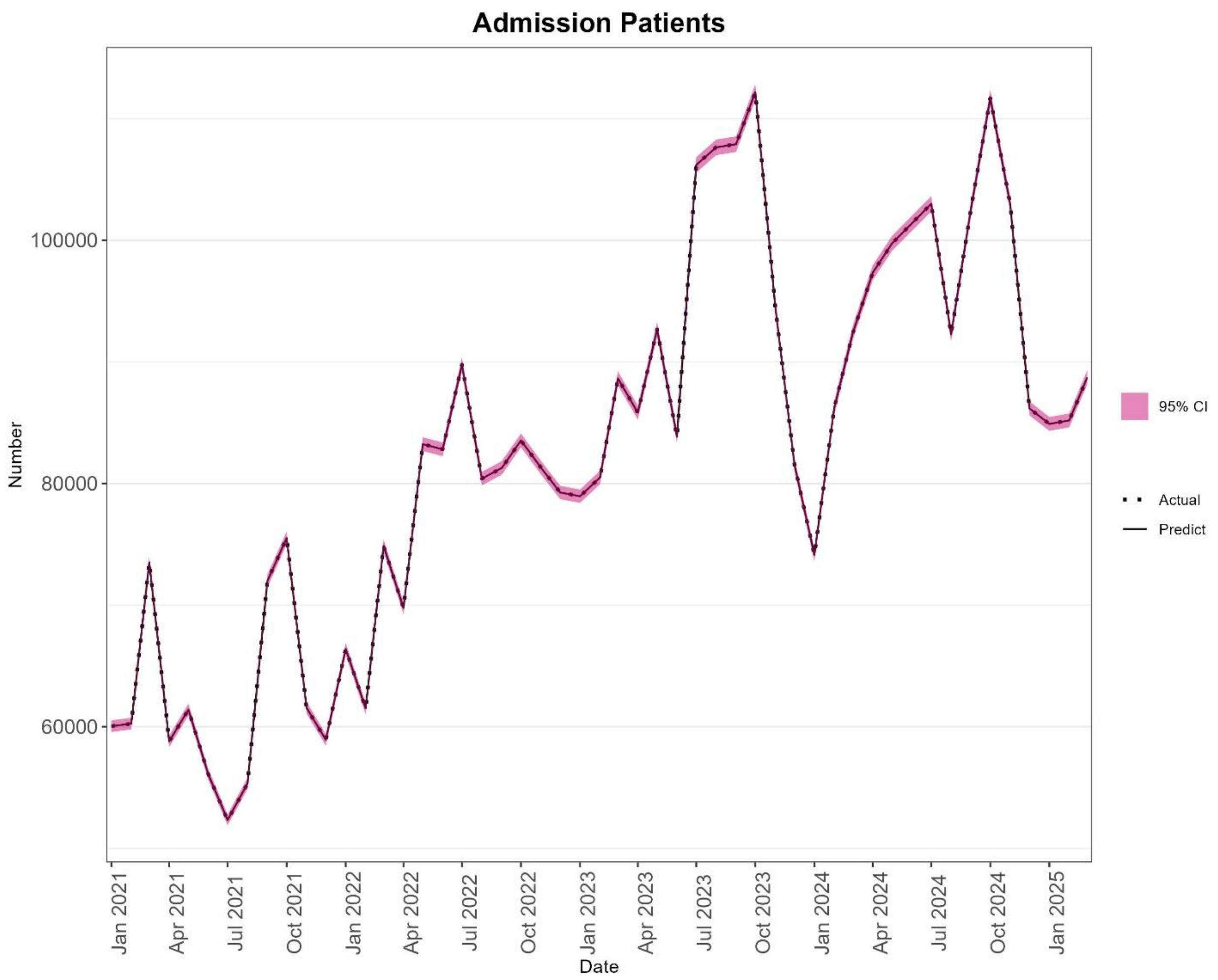
Admission Patients
Number
100000
80000
60000
Date
Jan 2021
Apr 2021
Jul 2021
Oct 2021
Jan 2022
Apr 2022
Jul 2022
Oct 2022
Jan 2023
Apr 2023
Jul 2023
Oct 2023
Jan 2024
Apr 2024
Jul 2024
Oct 2024
Jan 2025
95% CI
Actual
Predict

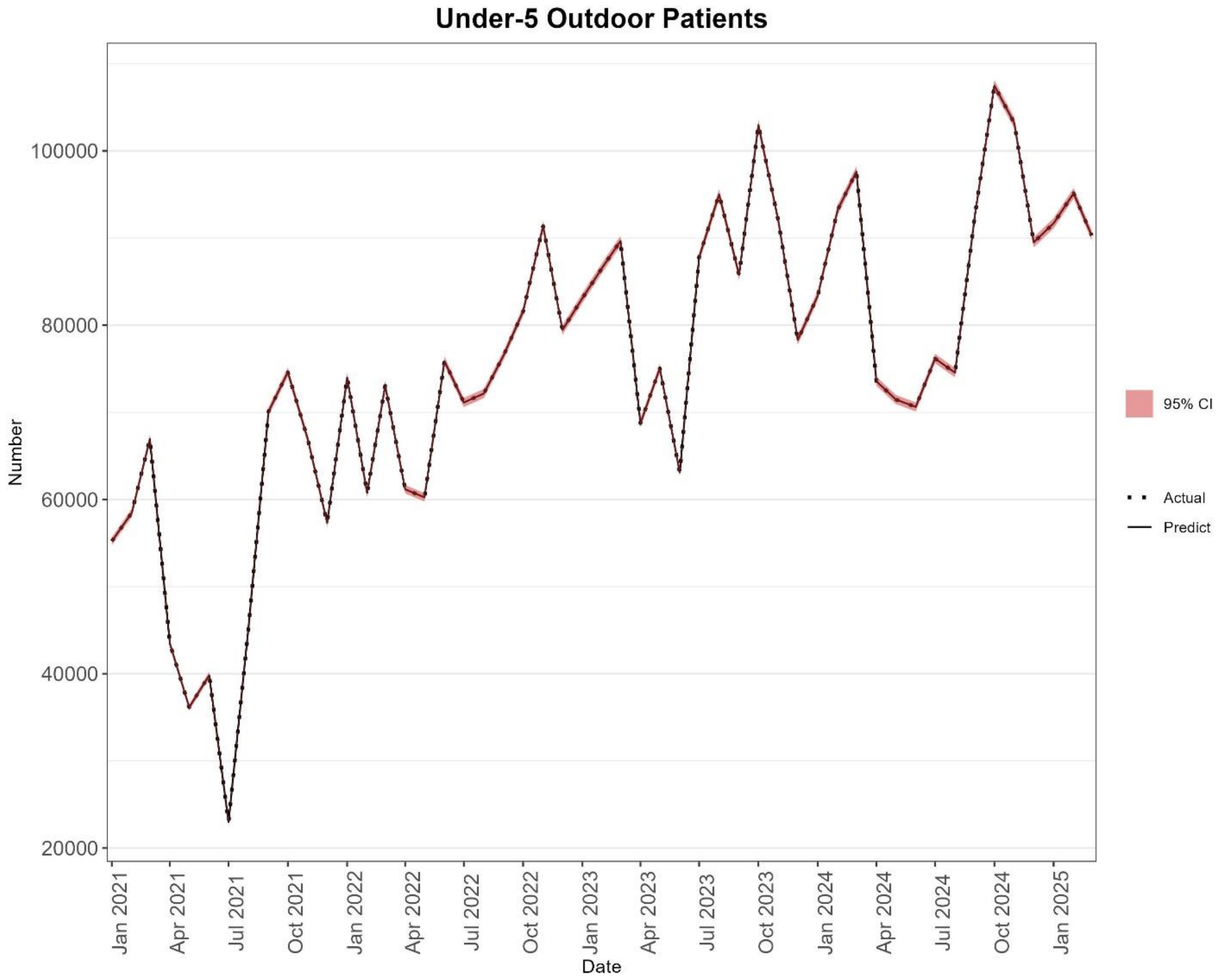
Under-5 Outdoor Patients
100000
80000
60000
40000
20000
Number
Jan 2021
Apr 2021
Jul 2021
Oct 2021
Jan 2022
Apr 2022
Jul 2022
Oct 2022
Jan 2023
Apr 2023
Jul 2023
Oct 2023
Jan 2024
Apr 2024
Jul 2024
Oct 2024
Jan 2025
Date
95% CI
Actual
Predict

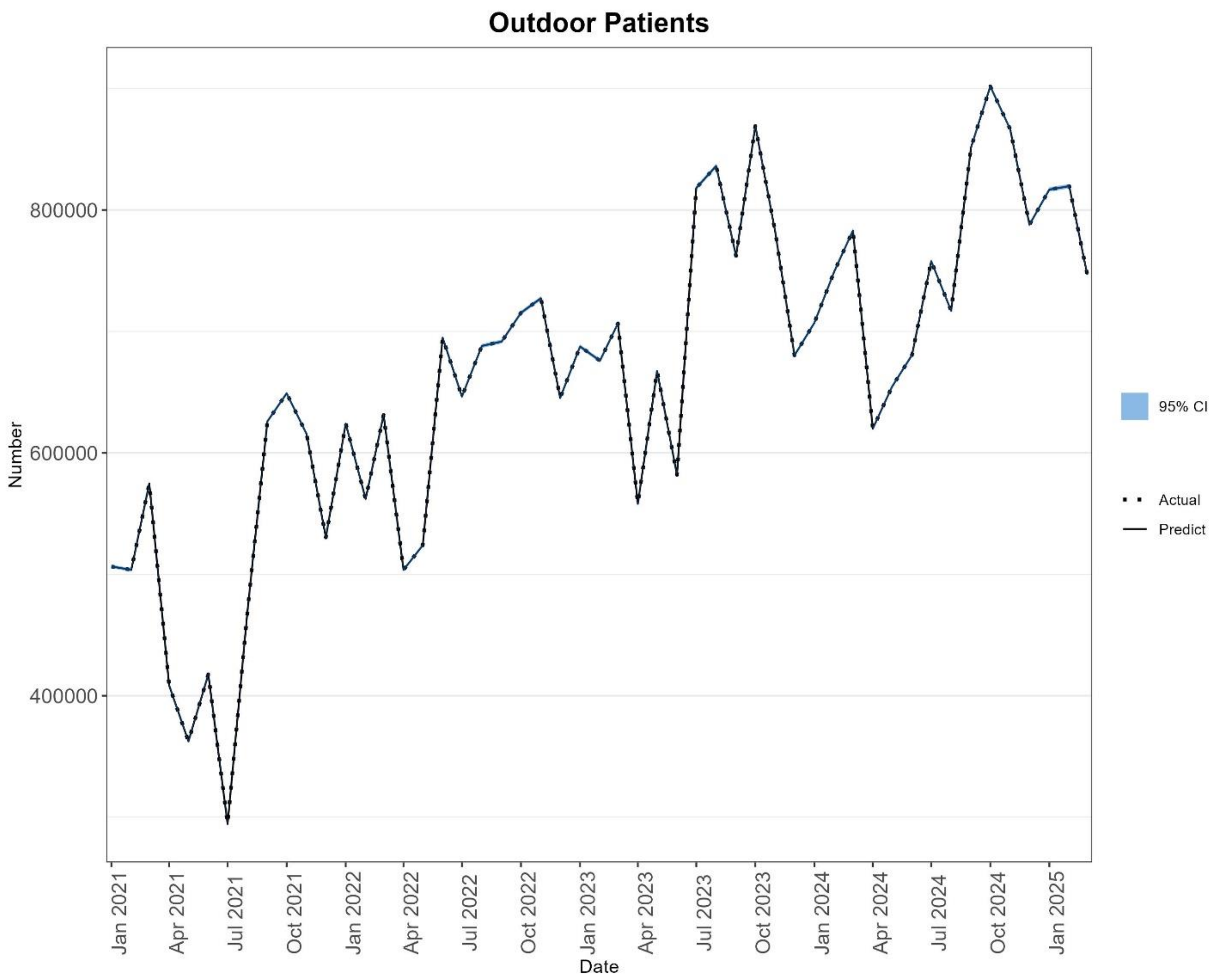

Outdoor Patients
800000
600000
400000
Number
Jan 2021
Apr 2021
Jul 2021
Oct 2021
Jan 2022
Apr 2022
Jul 2022
Oct 2022
Jan 2023
Apr 2023
Jul 2023
Oct 2023
Jan 2024
Apr 2024
Jul 2024
Oct 2024
Jan 2025
Date
95% CI
Actual
Predict

Mymensingh

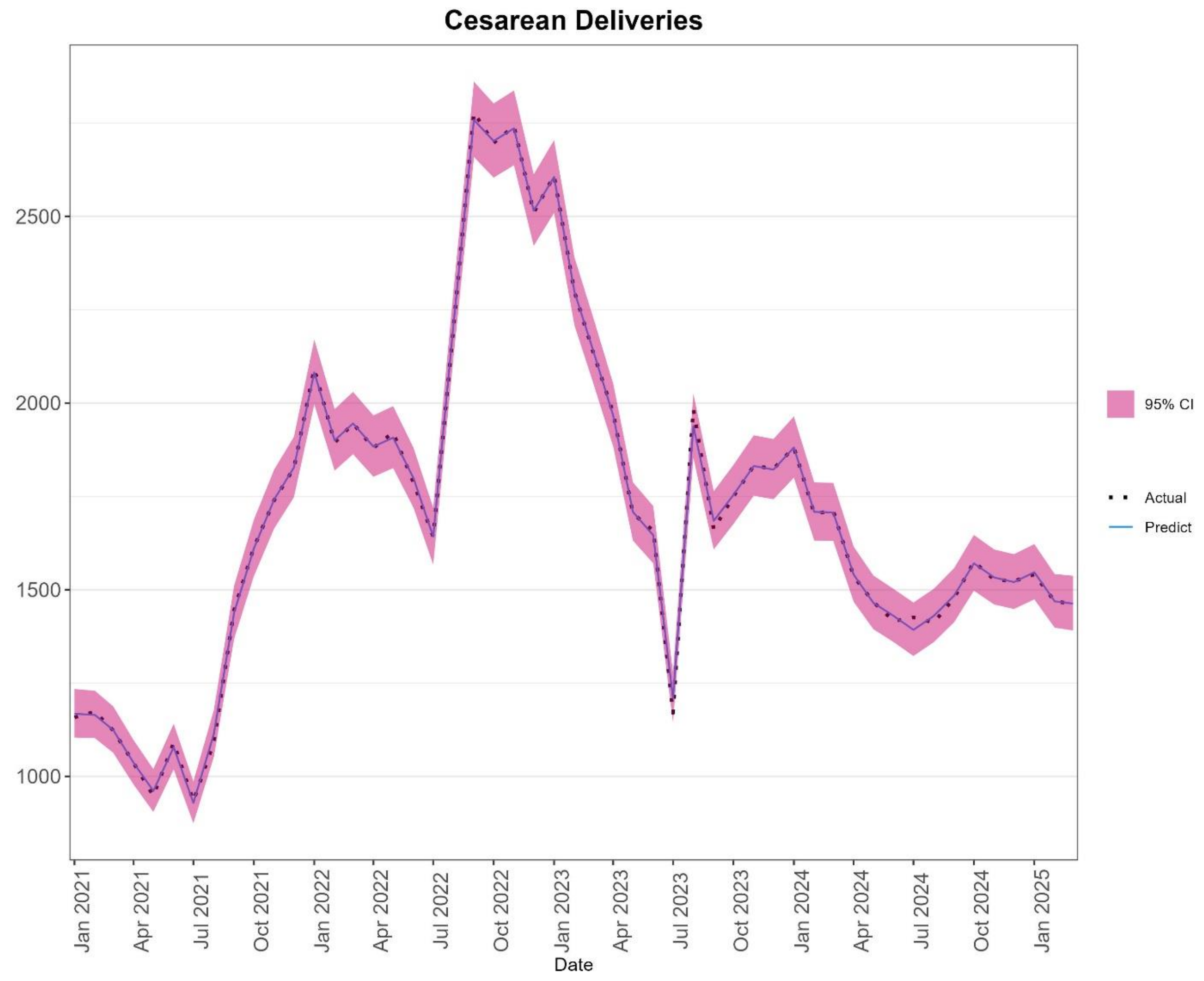
Cesarean Deliveries
2500
2000
1500
1000
Jan 2021
Apr 2021
Jul 2021
Oct 2021
Jan 2022
Apr 2022
Jul 2022
Oct 2022
Jan 2023
Apr 2023
Jul 2023
Oct 2023
Jan 2024
Apr 2024
Jul 2024
Oct 2024
Jan 2025
Date
95% CI
Actual
Predict

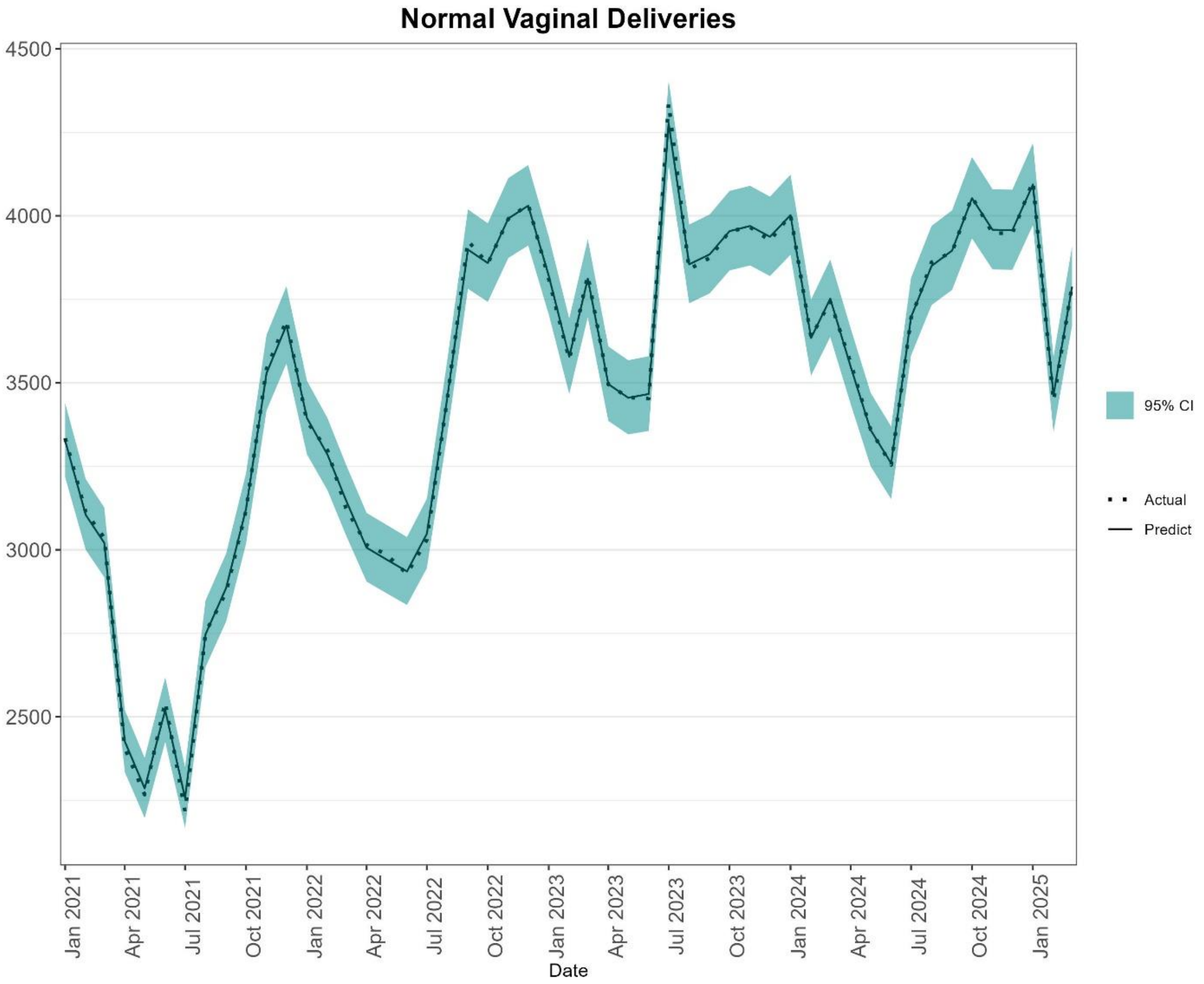

Normal Vaginal Deliveries
4500
4000
3500
3000
2500
Jan 2021
Apr 2021
Jul 2021
Oct 2021
Jan 2022
Apr 2022
Jul 2022
Oct 2022
Jan 2023
Apr 2023
Jul 2023
Oct 2023
Jan 2024
Apr 2024
Jul 2024
Oct 2024
Jan 2025
Date
95% CI
Actual
Predict

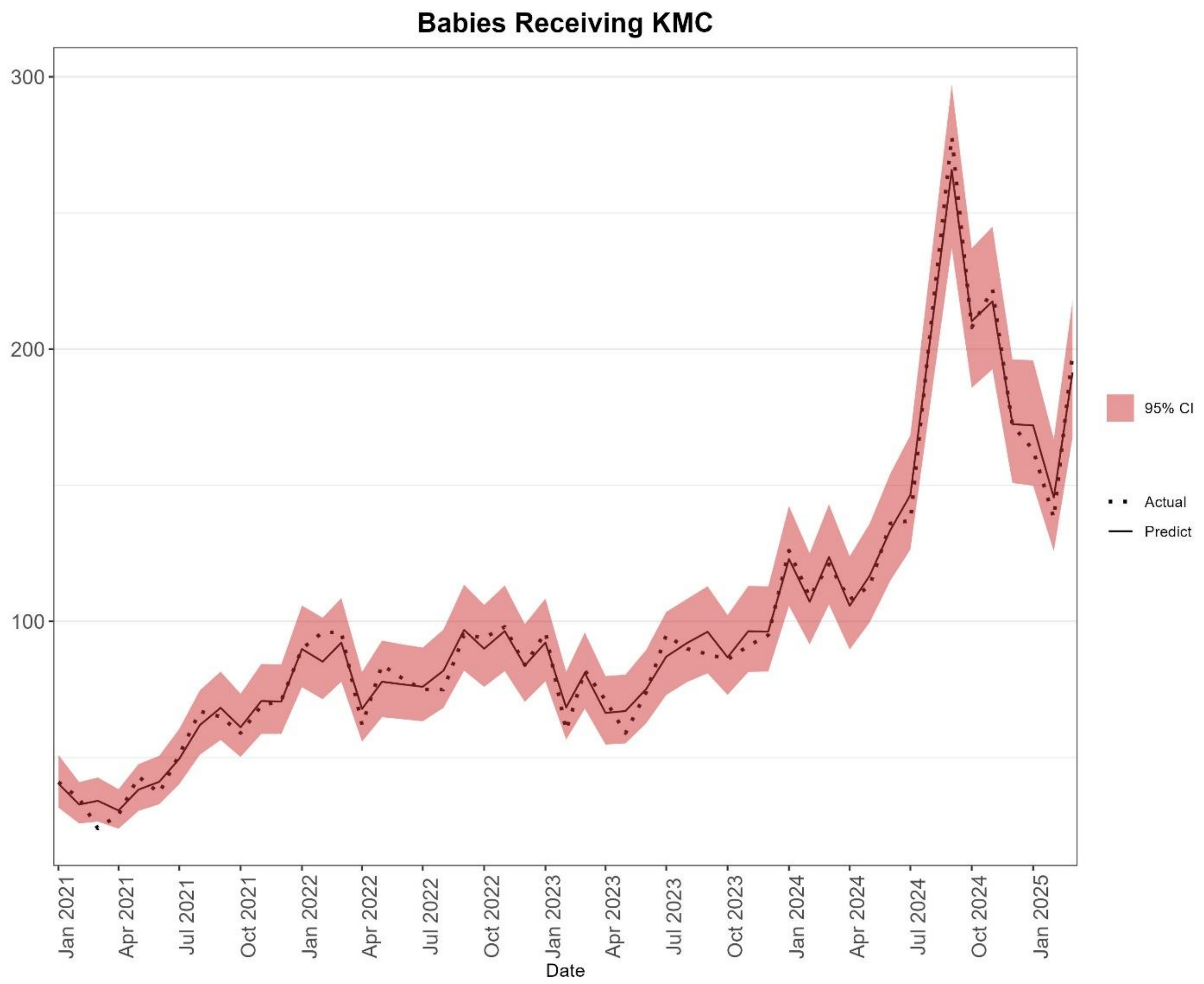

Babies Receiving KMC
300
200
100
Jan 2021
Apr 2021
Jul 2021
Oct 2021
Jan 2022
Apr 2022
Jul 2022
Oct 2022
Jan 2023
Apr 2023
Jul 2023
Oct 2023
Jan 2024
Apr 2024
Jul 2024
Oct 2024
Jan 2025
Date
95% CI
Actual
Predict

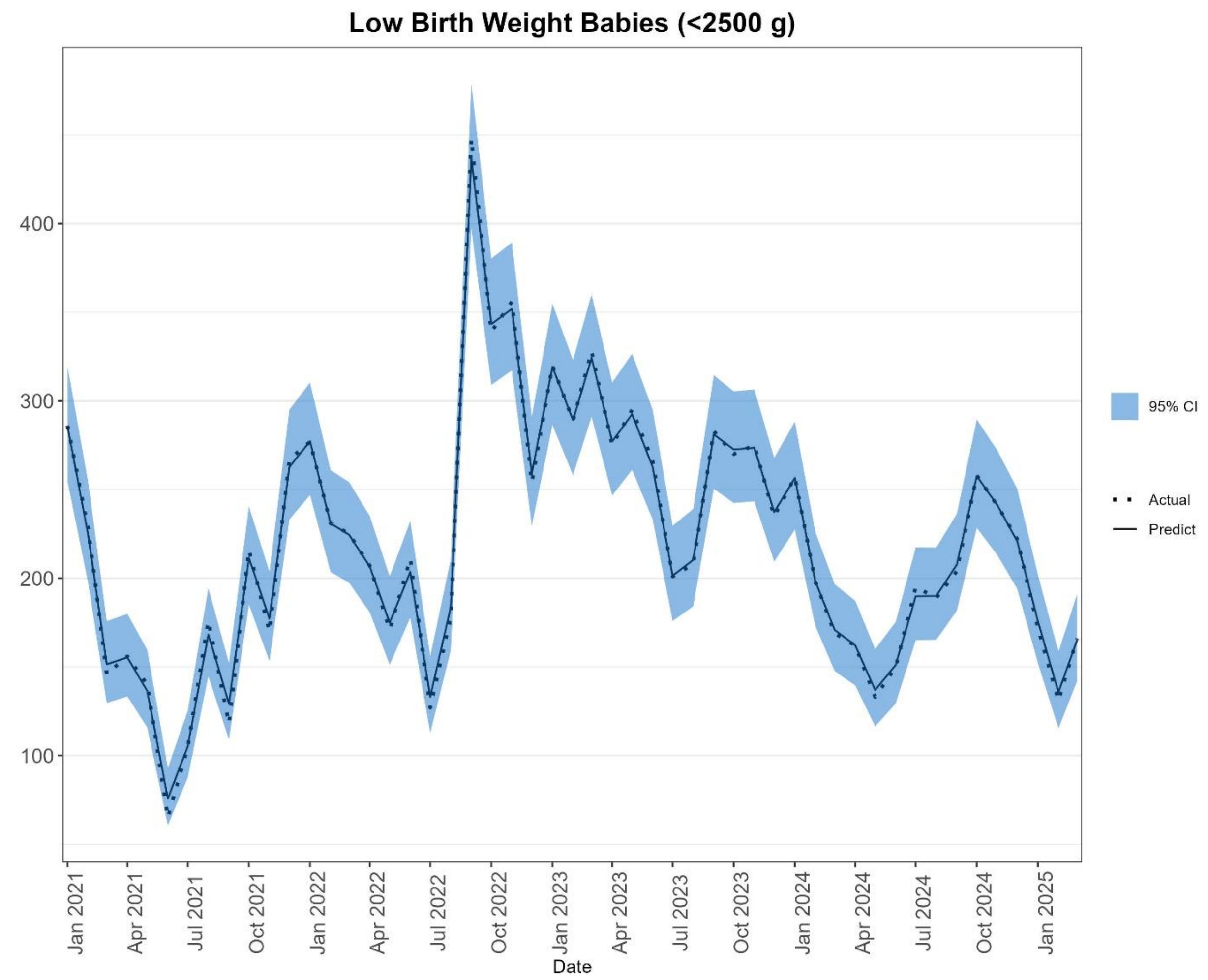

Low Birth Weight Babies (<2500 g)
400
300
200
100
Jan 2021
Apr 2021
Jul 2021
Oct 2021
Jan 2022
Apr 2022
Jul 2022
Oct 2022
Jan 2023
Apr 2023
Jul 2023
Oct 2023
Jan 2024
Apr 2024
Jul 2024
Oct 2024
Jan 2025
Date
95% CI
Actual
Predict

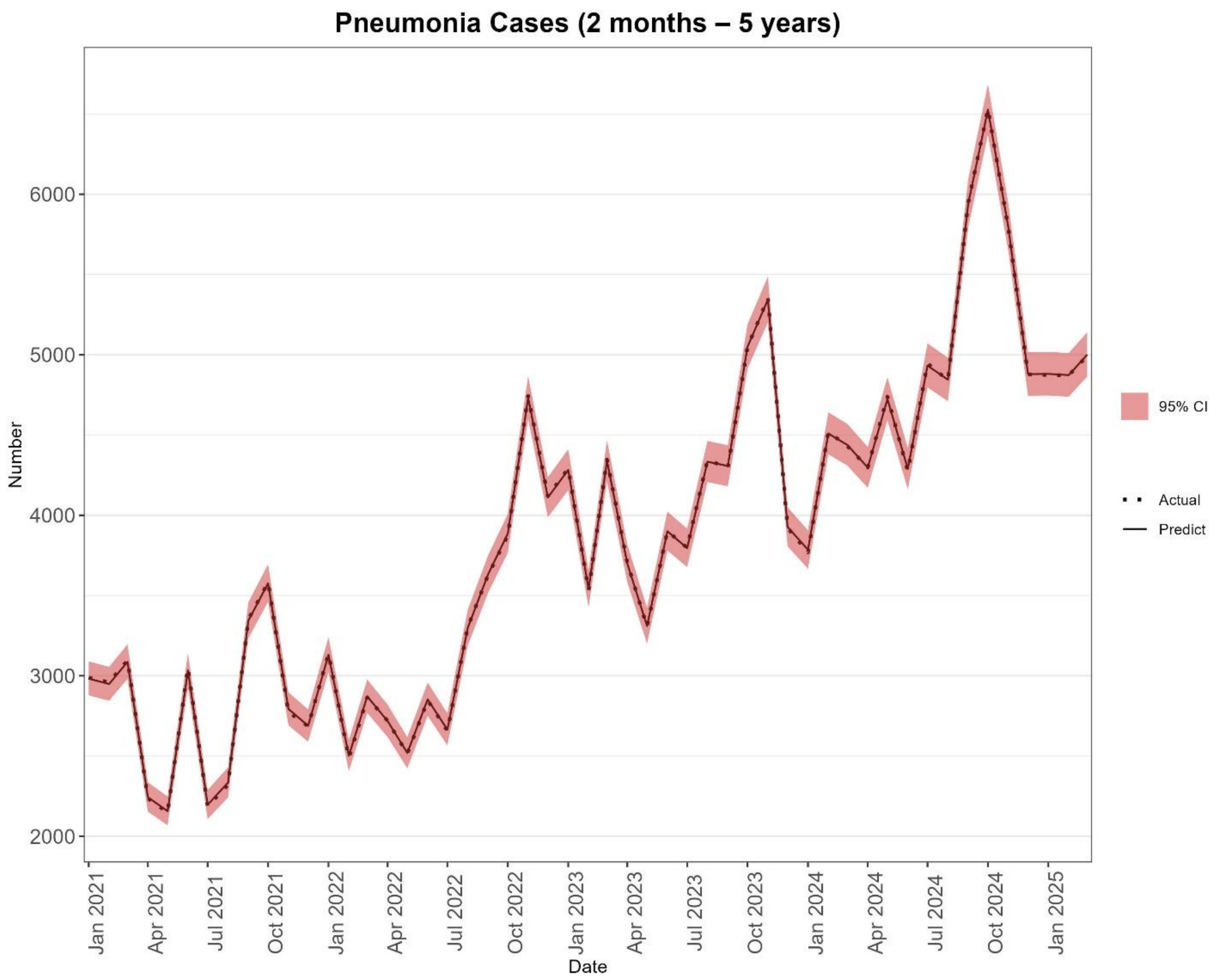

Pneumonia Cases (2 months – 5 years)
Number
Date
2000
3000
4000
5000
6000
Jan 2021
Apr 2021
Jul 2021
Oct 2021
Jan 2022
Apr 2022
Jul 2022
Oct 2022
Jan 2023
Apr 2023
Jul 2023
Oct 2023
Jan 2024
Apr 2024
Jul 2024
Oct 2024
Jan 2025
95% CI
Actual
Predict

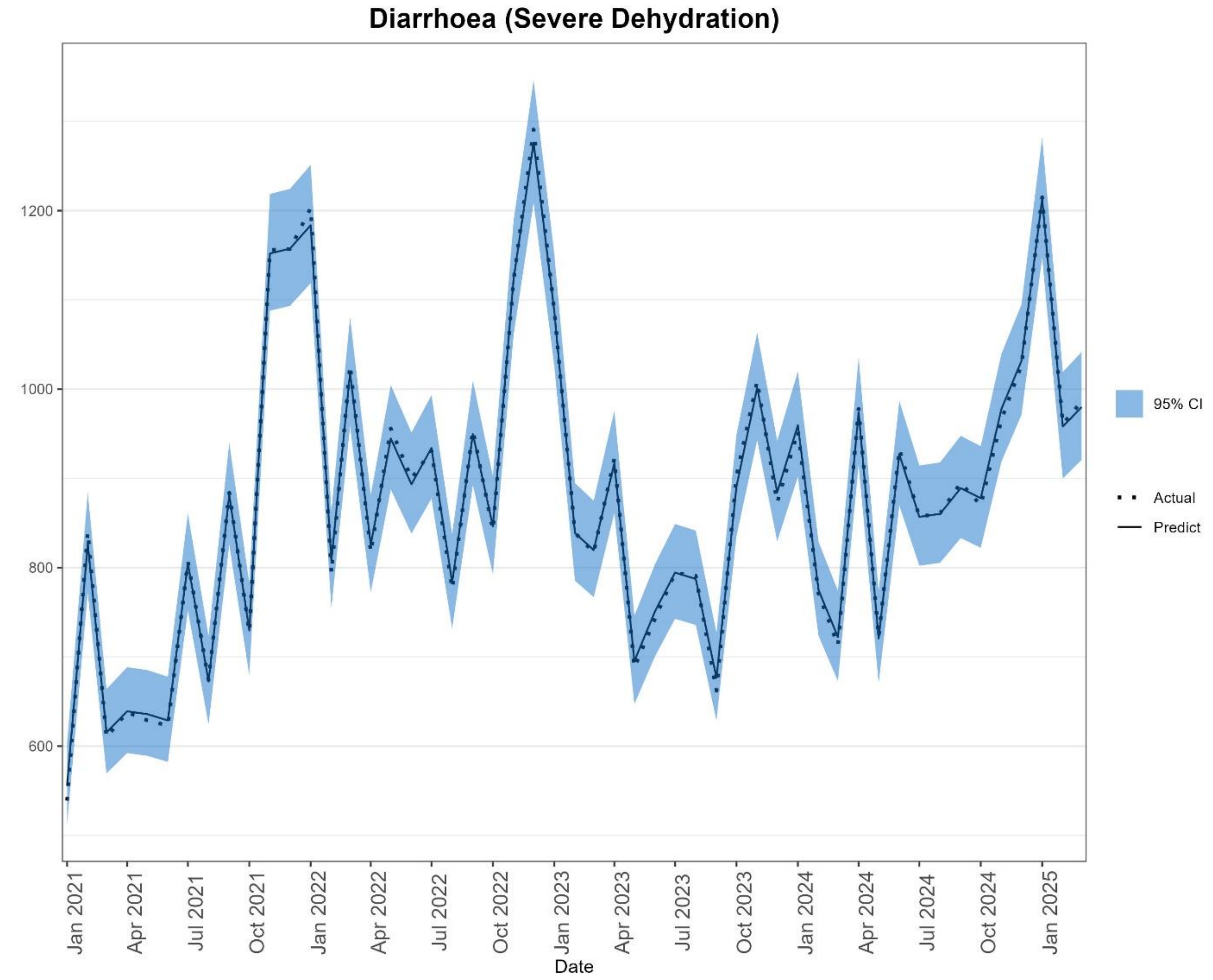

Diarrhoea (Severe Dehydration)
1200
1000
800
600
Jan 2021
Apr 2021
Jul 2021
Oct 2021
Jan 2022
Apr 2022
Jul 2022
Oct 2022
Jan 2023
Apr 2023
Jul 2023
Oct 2023
Jan 2024
Apr 2024
Jul 2024
Oct 2024
Jan 2025
Date
95% CI
Actual
Predict

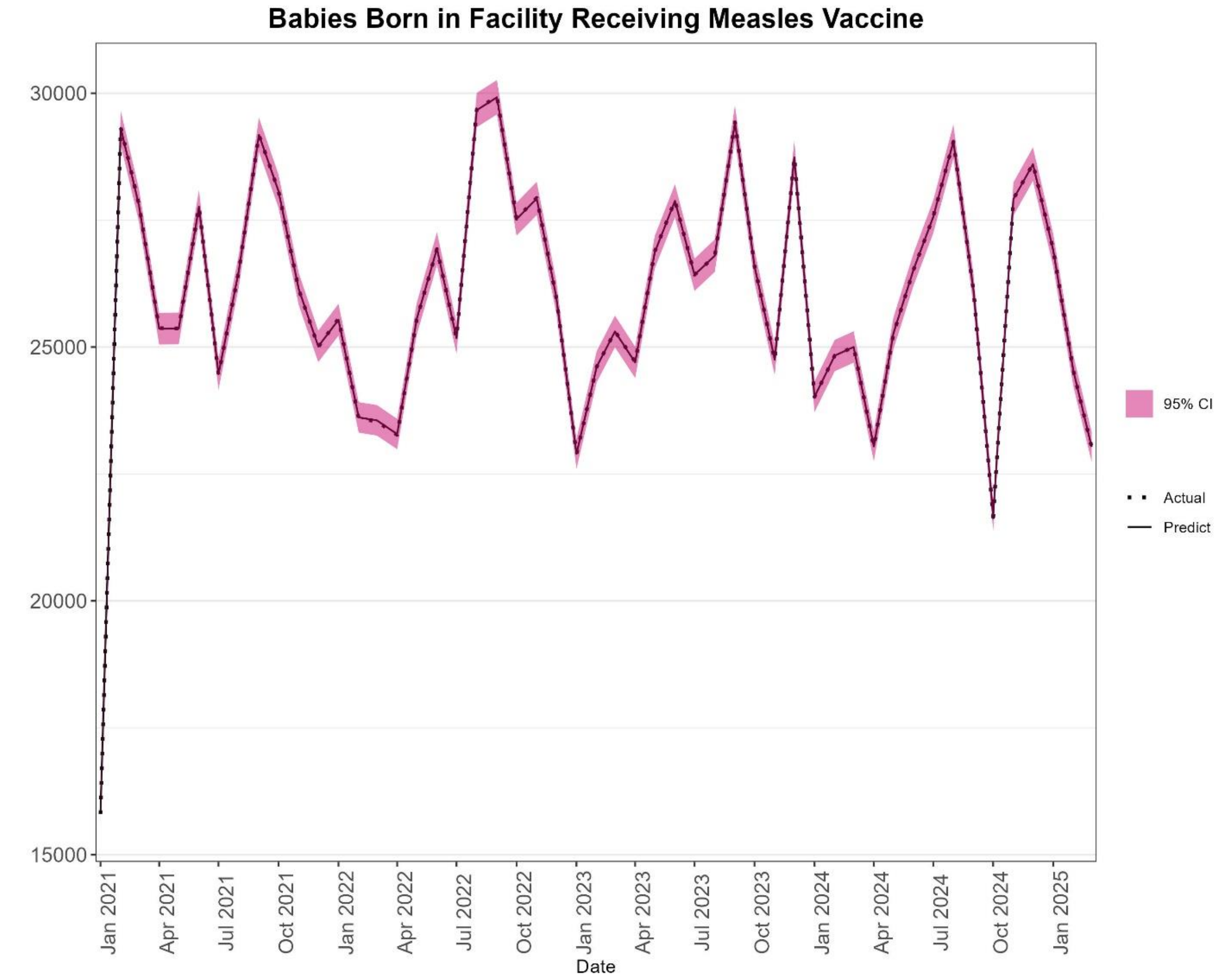

Babies Born in Facility Receiving Measles Vaccine
30000
25000
20000
15000
Jan 2021
Apr 2021
Jul 2021
Oct 2021
Jan 2022
Apr 2022
Jul 2022
Oct 2022
Jan 2023
Apr 2023
Jul 2023
Oct 2023
Jan 2024
Apr 2024
Jul 2024
Oct 2024
Jan 2025
Date
95% CI
Actual
Predict

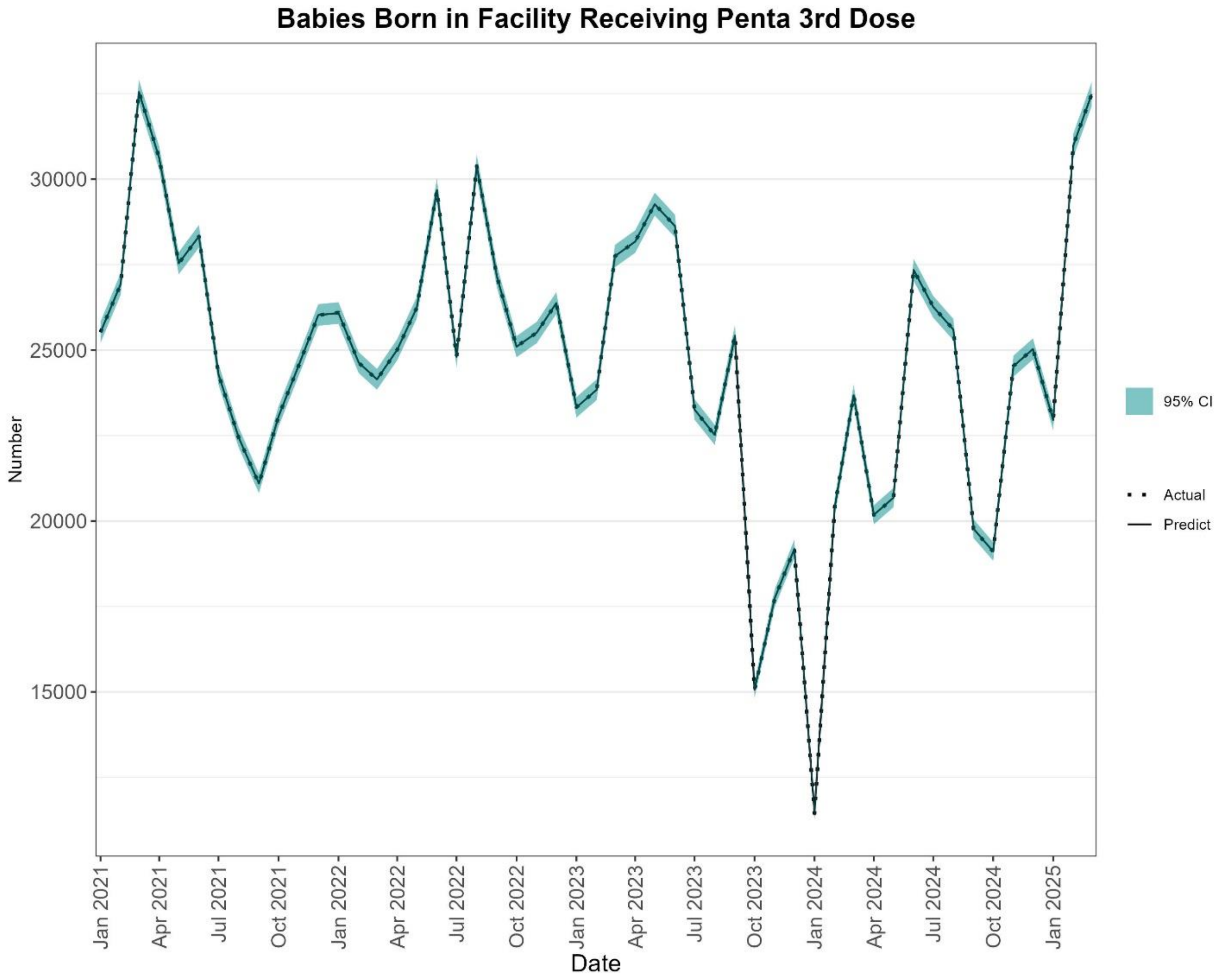
Babies Born in Facility Receiving Penta 3rd Dose
Number
30000
25000
20000
15000
Jan 2021
Apr 2021
Jul 2021
Oct 2021
Jan 2022
Apr 2022
Jul 2022
Oct 2022
Jan 2023
Apr 2023
Jul 2023
Oct 2023
Jan 2024
Apr 2024
Jul 2024
Oct 2024
Jan 2025
Date
95% CI
Actual
Predict

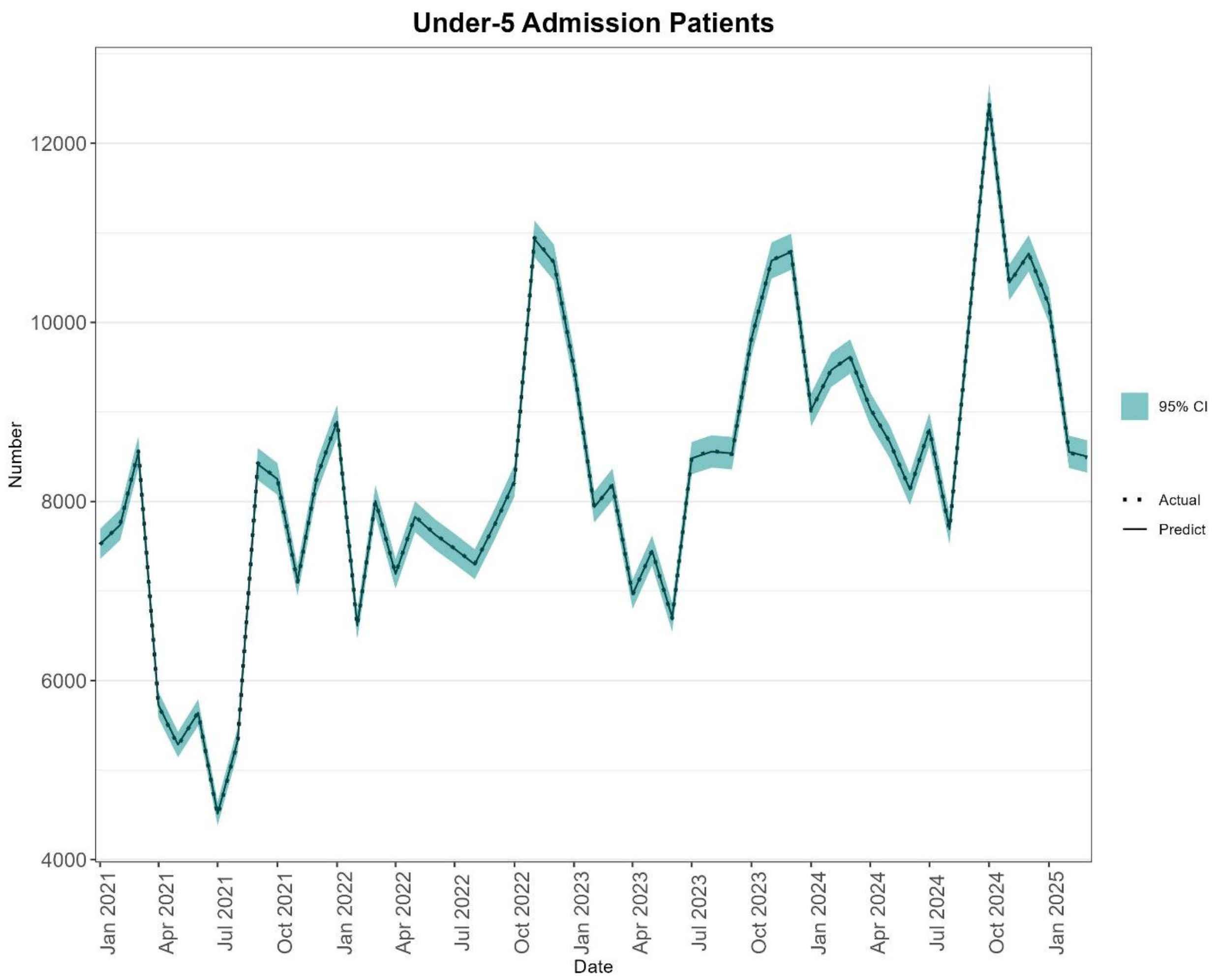

Under-5 Admission Patients
Number
12000
10000
8000
6000
4000
Date
Jan 2021
Apr 2021
Jul 2021
Oct 2021
Jan 2022
Apr 2022
Jul 2022
Oct 2022
Jan 2023
Apr 2023
Jul 2023
Oct 2023
Jan 2024
Apr 2024
Jul 2024
Oct 2024
Jan 2025
95% CI
Actual
Predict

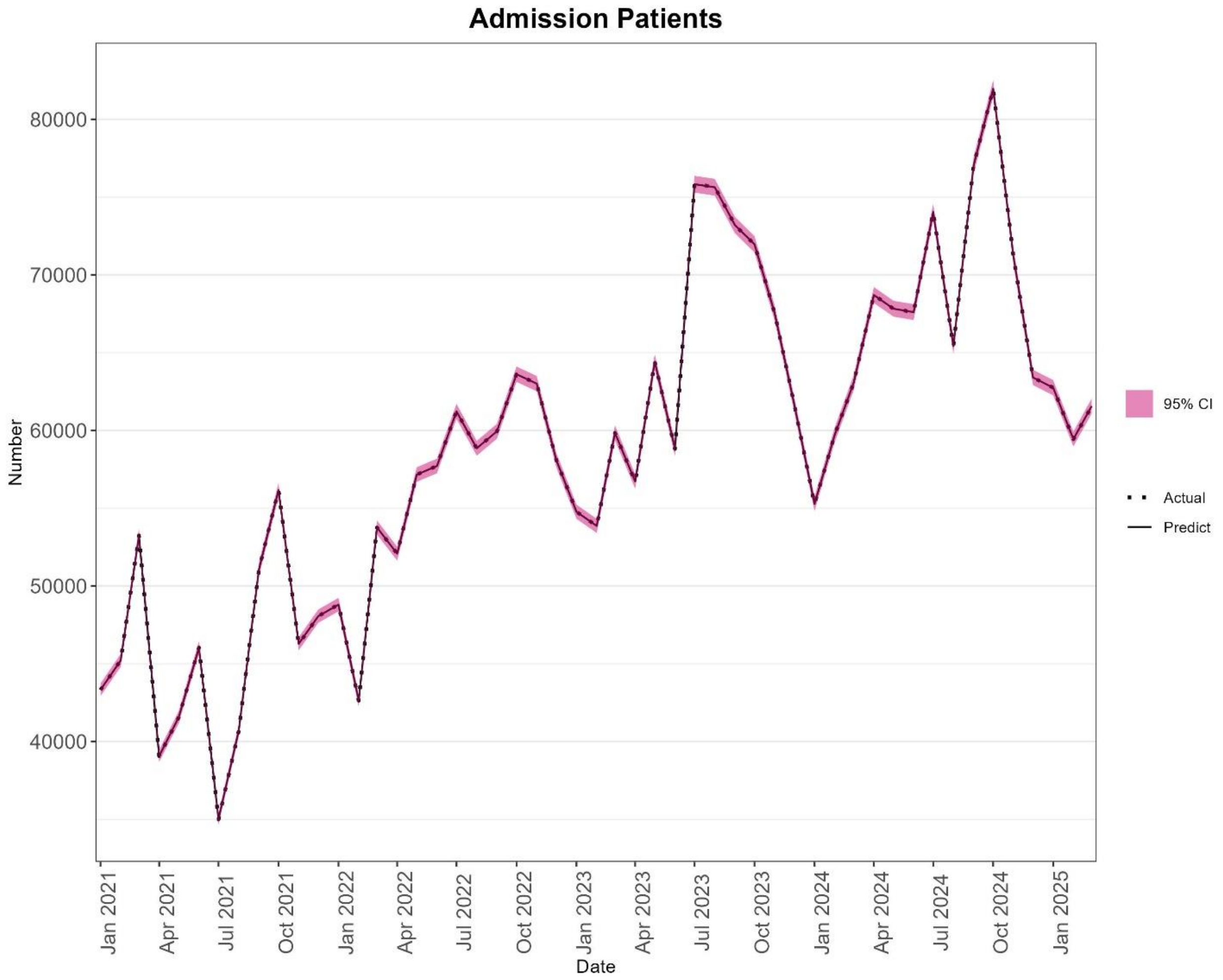

Admission Patients
80000
70000
60000
50000
40000
Number
Jan 2021
Apr 2021
Jul 2021
Oct 2021
Jan 2022
Apr 2022
Jul 2022
Oct 2022
Jan 2023
Apr 2023
Jul 2023
Oct 2023
Jan 2024
Apr 2024
Jul 2024
Oct 2024
Jan 2025
Date
95% CI
Actual
Predict

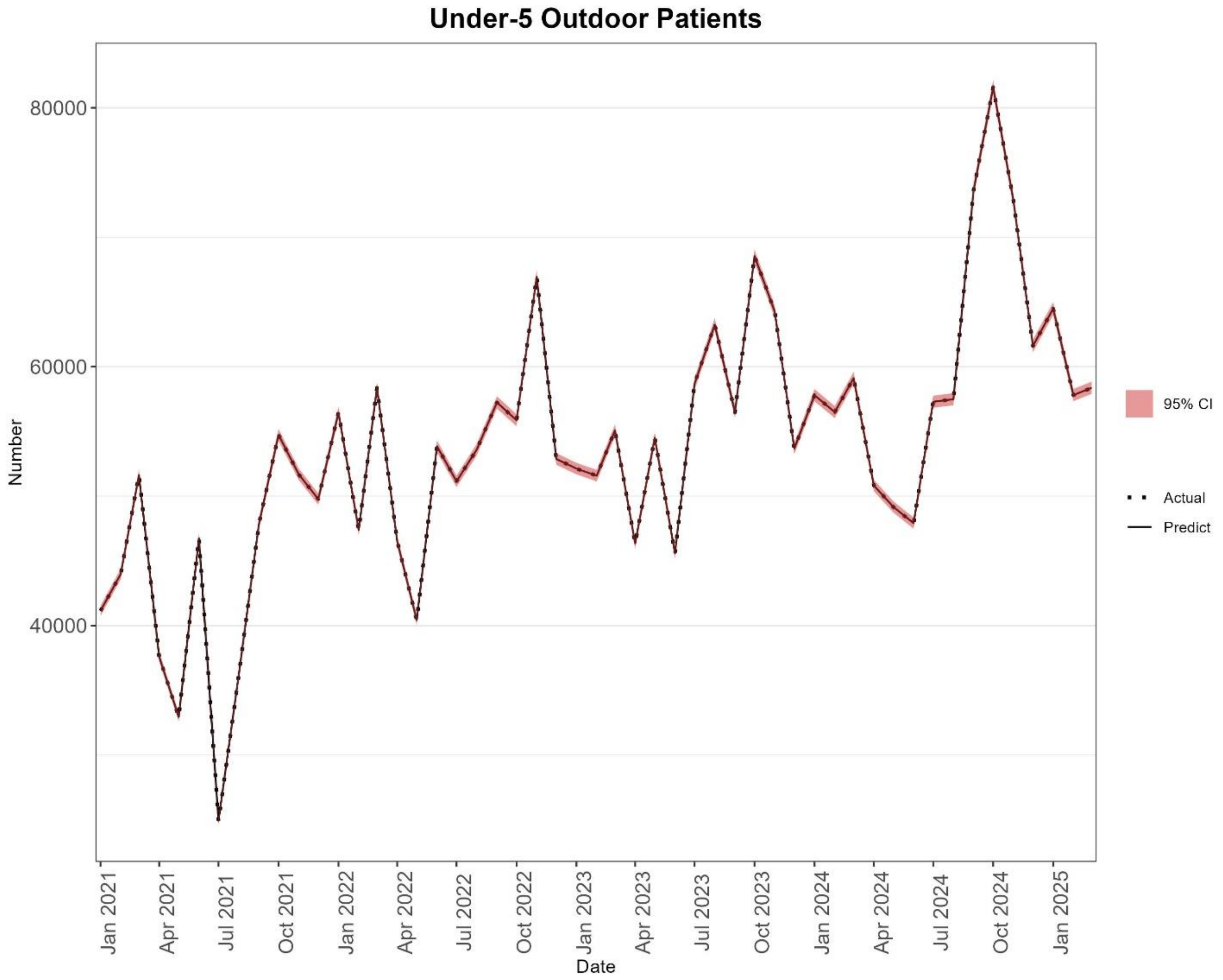

Under-5 Outdoor Patients
80000
60000
40000
Number
Jan 2021
Apr 2021
Jul 2021
Oct 2021
Jan 2022
Apr 2022
Jul 2022
Oct 2022
Jan 2023
Apr 2023
Jul 2023
Oct 2023
Jan 2024
Apr 2024
Jul 2024
Oct 2024
Jan 2025
Date
95% CI
Actual
Predict

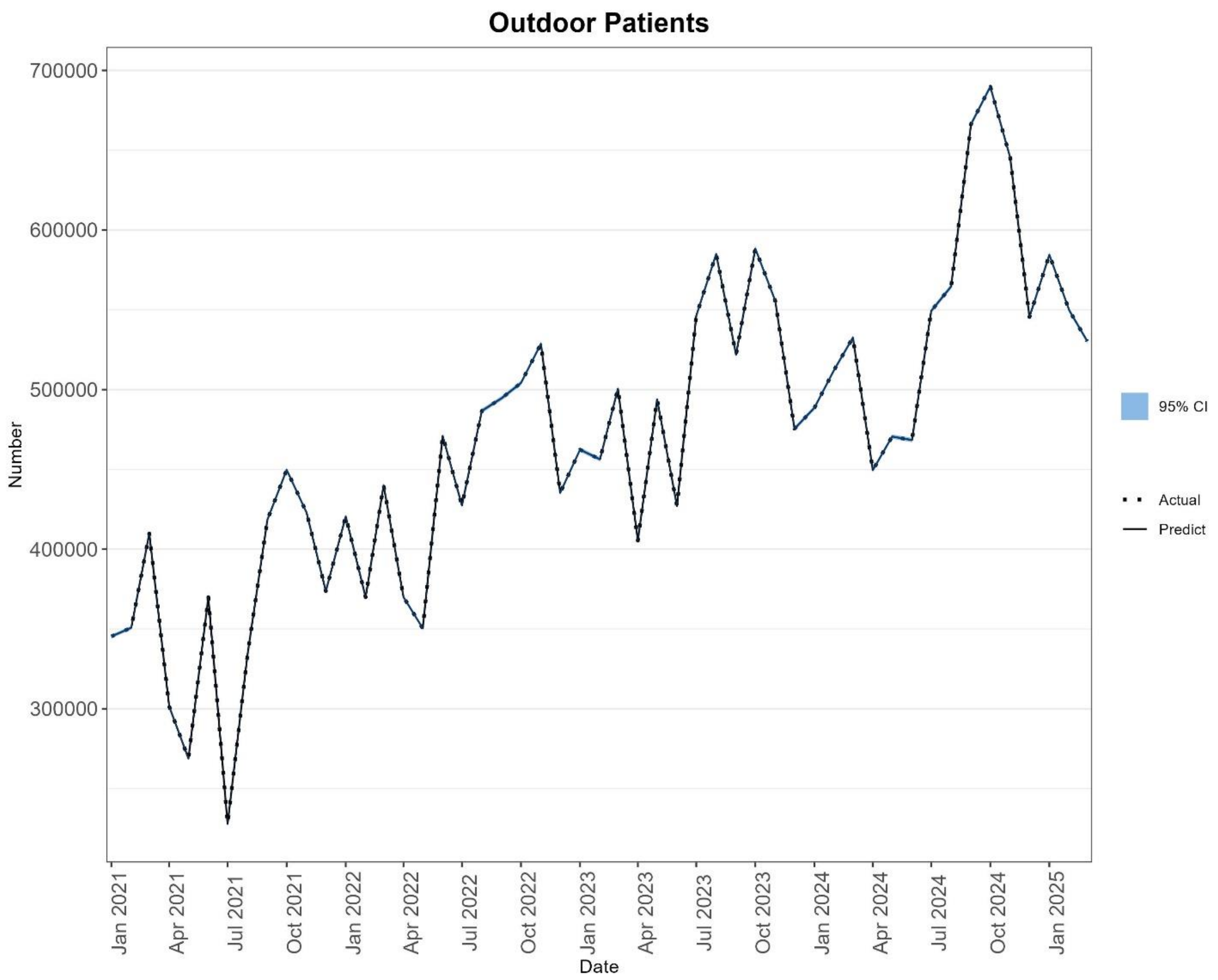

Outdoor Patients
700000
600000
500000
400000
300000
Number
Jan 2021
Apr 2021
Jul 2021
Oct 2021
Jan 2022
Apr 2022
Jul 2022
Oct 2022
Jan 2023
Apr 2023
Jul 2023
Oct 2023
Jan 2024
Apr 2024
Jul 2024
Oct 2024
Jan 2025
Date
95% CI
Actual
Predict

Rajshahi

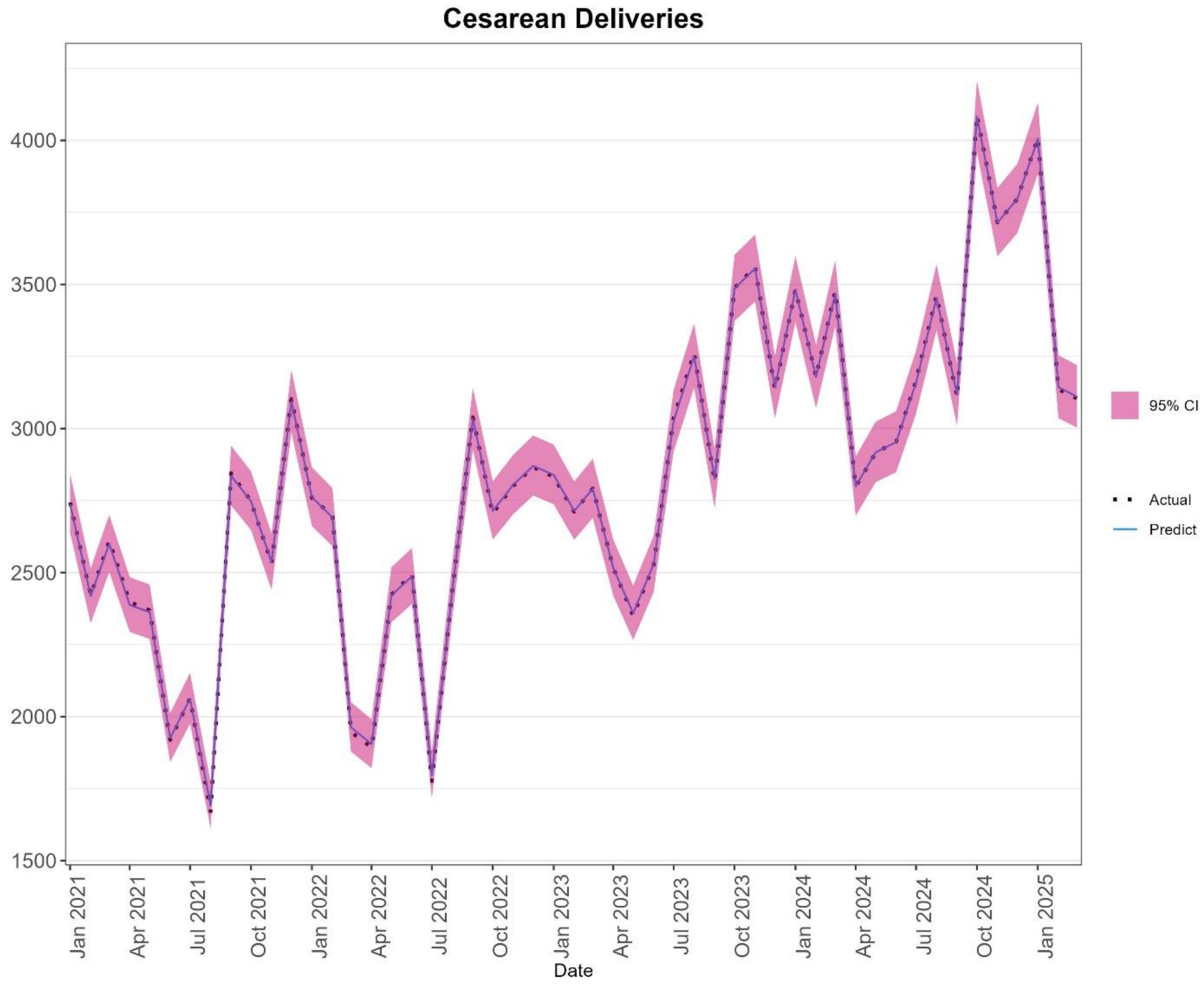

Cesarean Deliveries
4000
3500
3000
2500
2000
1500
Jan 2021
Apr 2021
Jul 2021
Oct 2021
Jan 2022
Apr 2022
Jul 2022
Oct 2022
Jan 2023
Apr 2023
Jul 2023
Oct 2023
Jan 2024
Apr 2024
Jul 2024
Oct 2024
Jan 2025
Date
95% CI
Actual
Predict

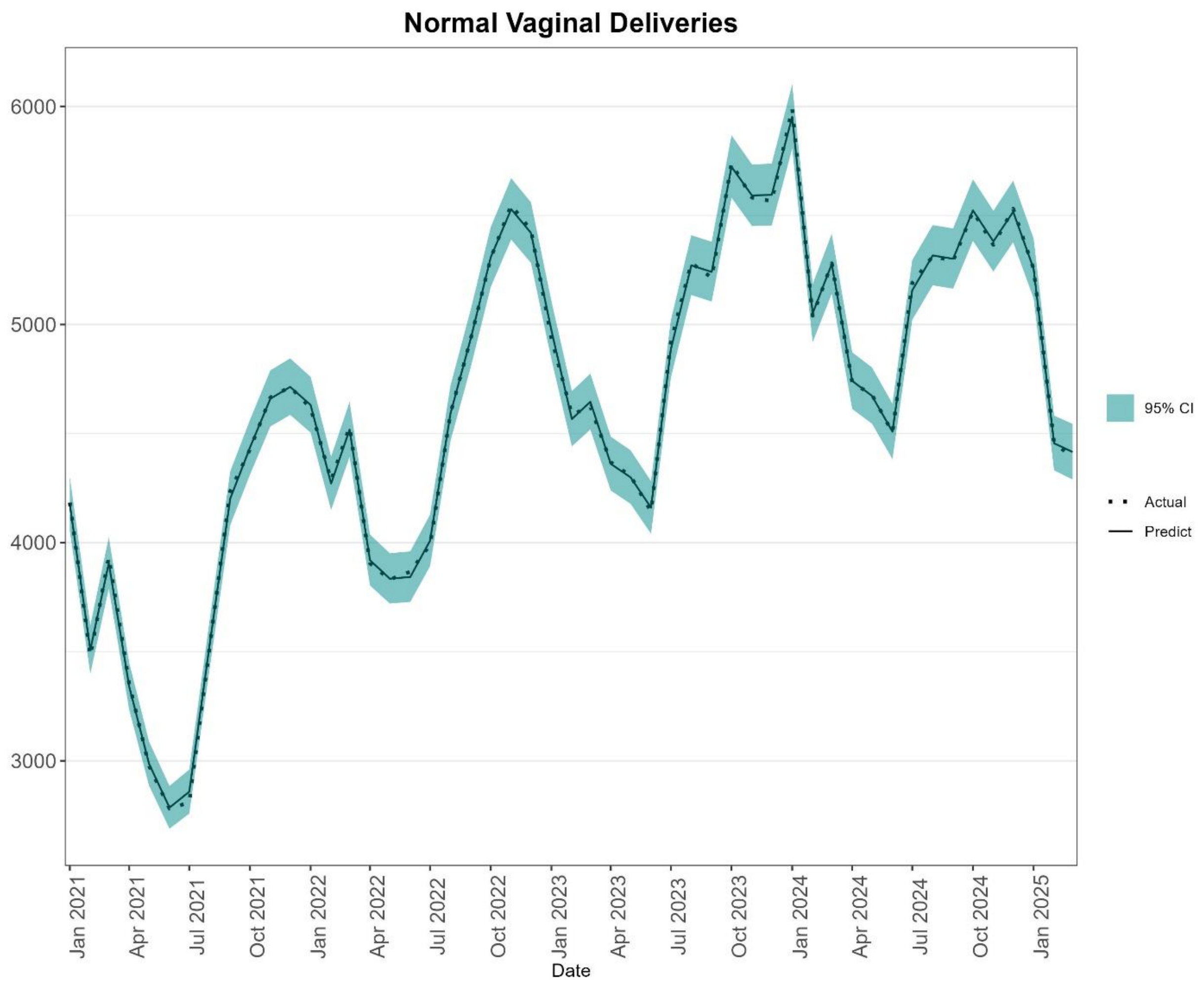

Normal Vaginal Deliveries
6000
5000
4000
3000
Jan 2021
Apr 2021
Jul 2021
Oct 2021
Jan 2022
Apr 2022
Jul 2022
Oct 2022
Jan 2023
Apr 2023
Jul 2023
Oct 2023
Jan 2024
Apr 2024
Jul 2024
Oct 2024
Jan 2025
Date
95% CI
Actual
Predict

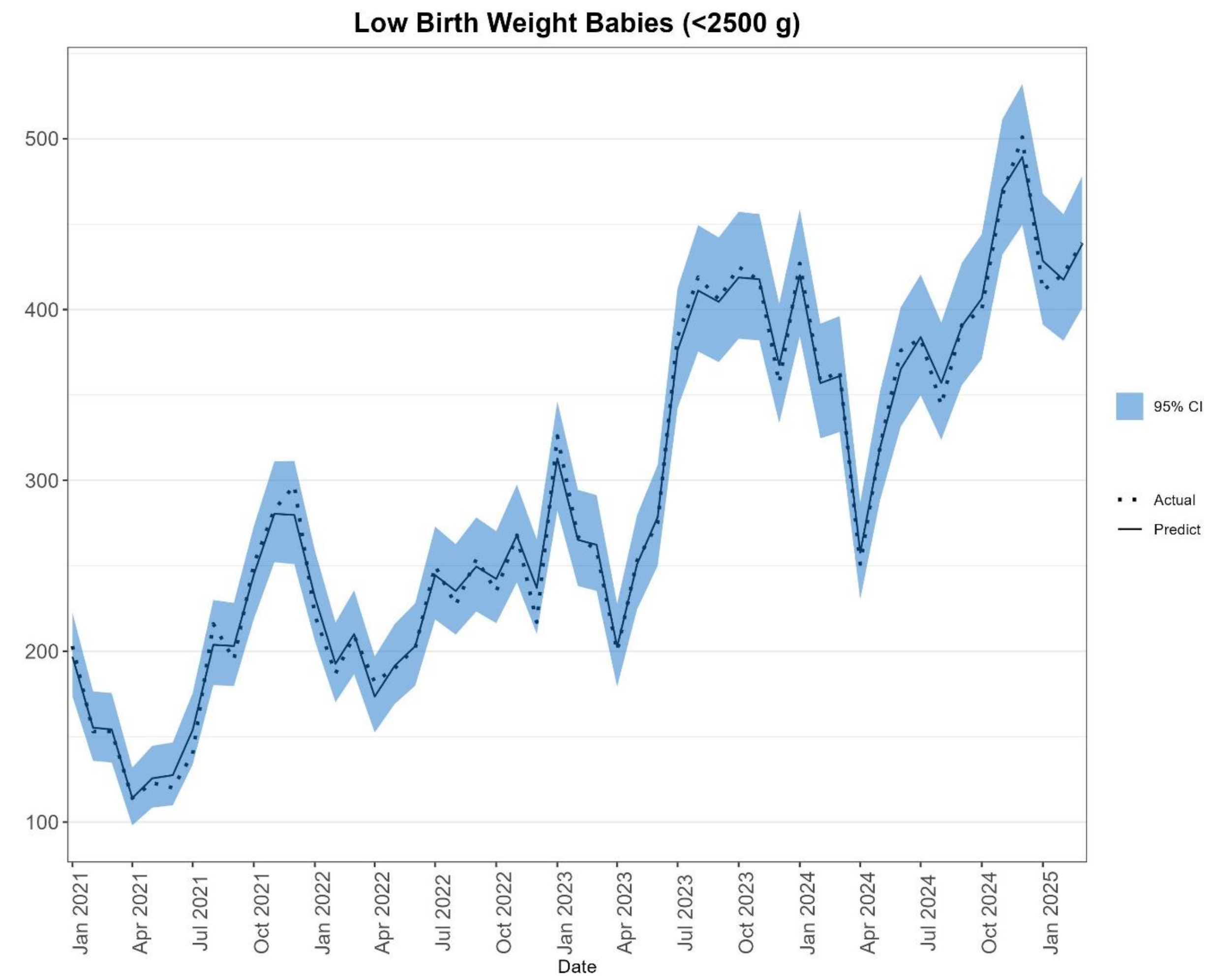

Low Birth Weight Babies (<2500 g)
500
400
300
200
100
Jan 2021
Apr 2021
Jul 2021
Oct 2021
Jan 2022
Apr 2022
Jul 2022
Oct 2022
Jan 2023
Apr 2023
Jul 2023
Oct 2023
Jan 2024
Apr 2024
Jul 2024
Oct 2024
Jan 2025
Date
95% CI
Actual
Predict

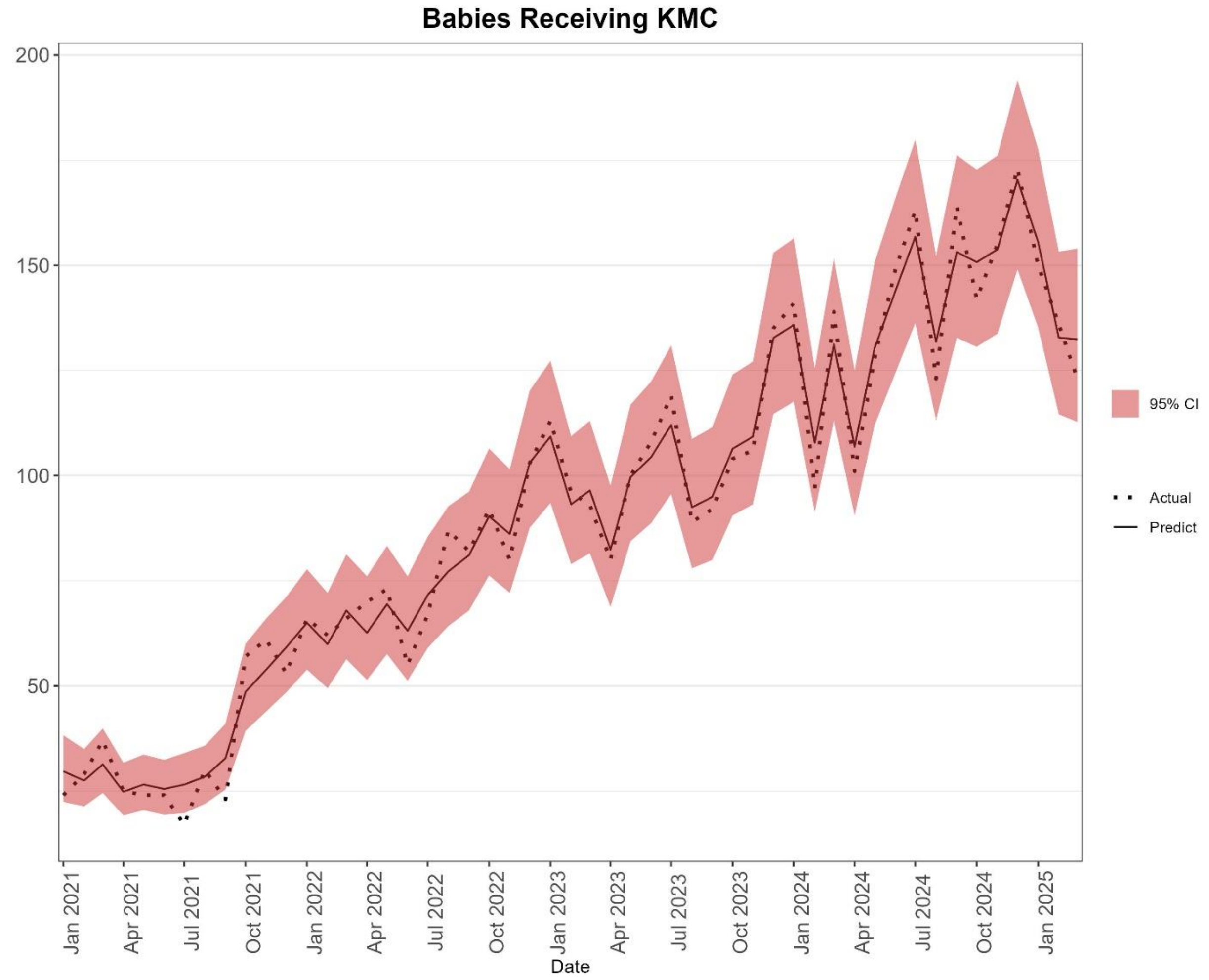
Babies Receiving KMC
200
150
100
50
95% CI
Actual
Predict
Jan 2021
Apr 2021
Jul 2021
Oct 2021
Jan 2022
Apr 2022
Jul 2022
Oct 2022
Jan 2023
Apr 2023
Jul 2023
Oct 2023
Jan 2024
Apr 2024
Jul 2024
Oct 2024
Jan 2025
Date

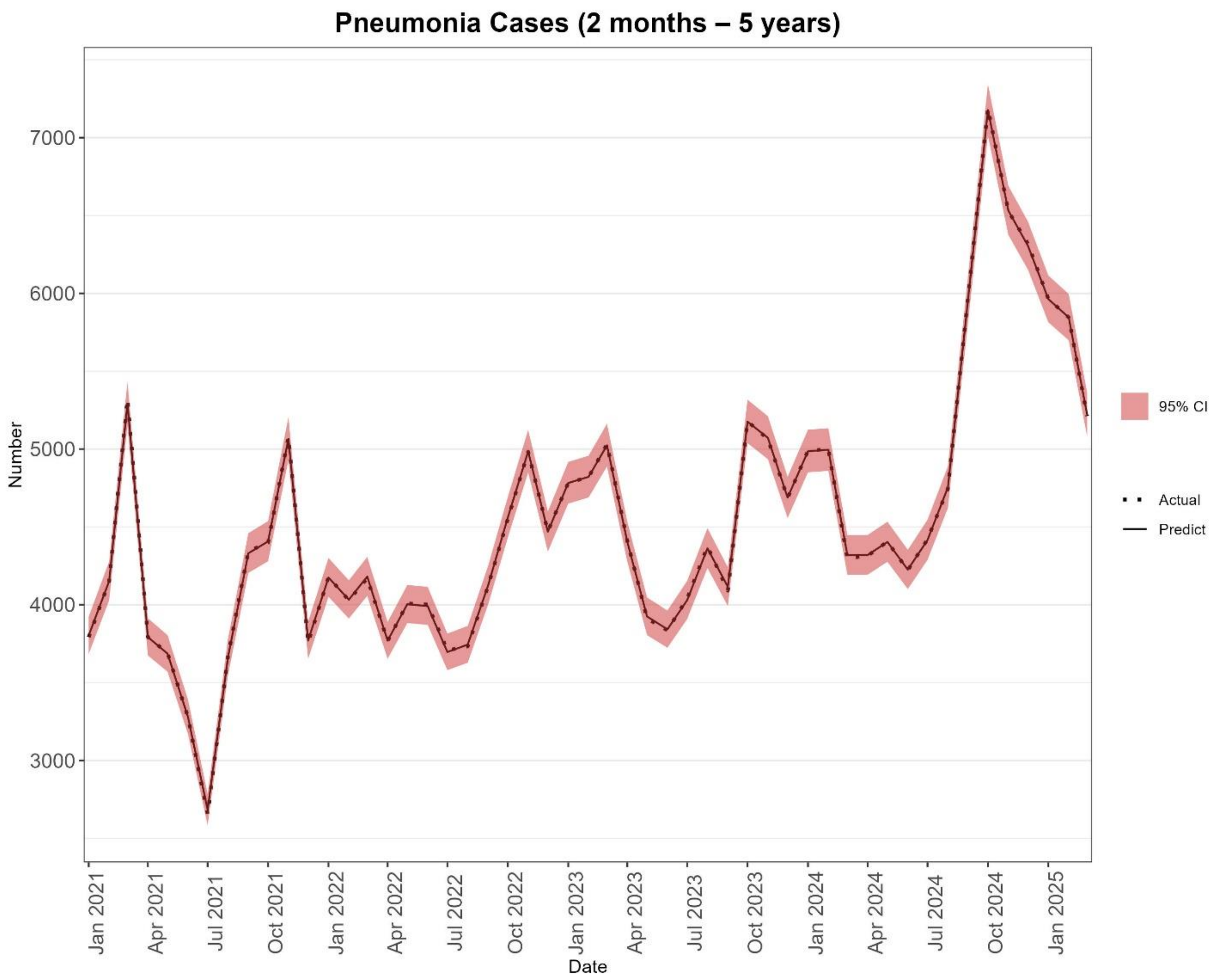

Pneumonia Cases (2 months – 5 years)
7000
6000
5000
4000
3000
Number
Jan 2021
Apr 2021
Jul 2021
Oct 2021
Jan 2022
Apr 2022
Jul 2022
Oct 2022
Jan 2023
Apr 2023
Jul 2023
Oct 2023
Jan 2024
Apr 2024
Jul 2024
Oct 2024
Jan 2025
Date
95% CI
Actual
Predict

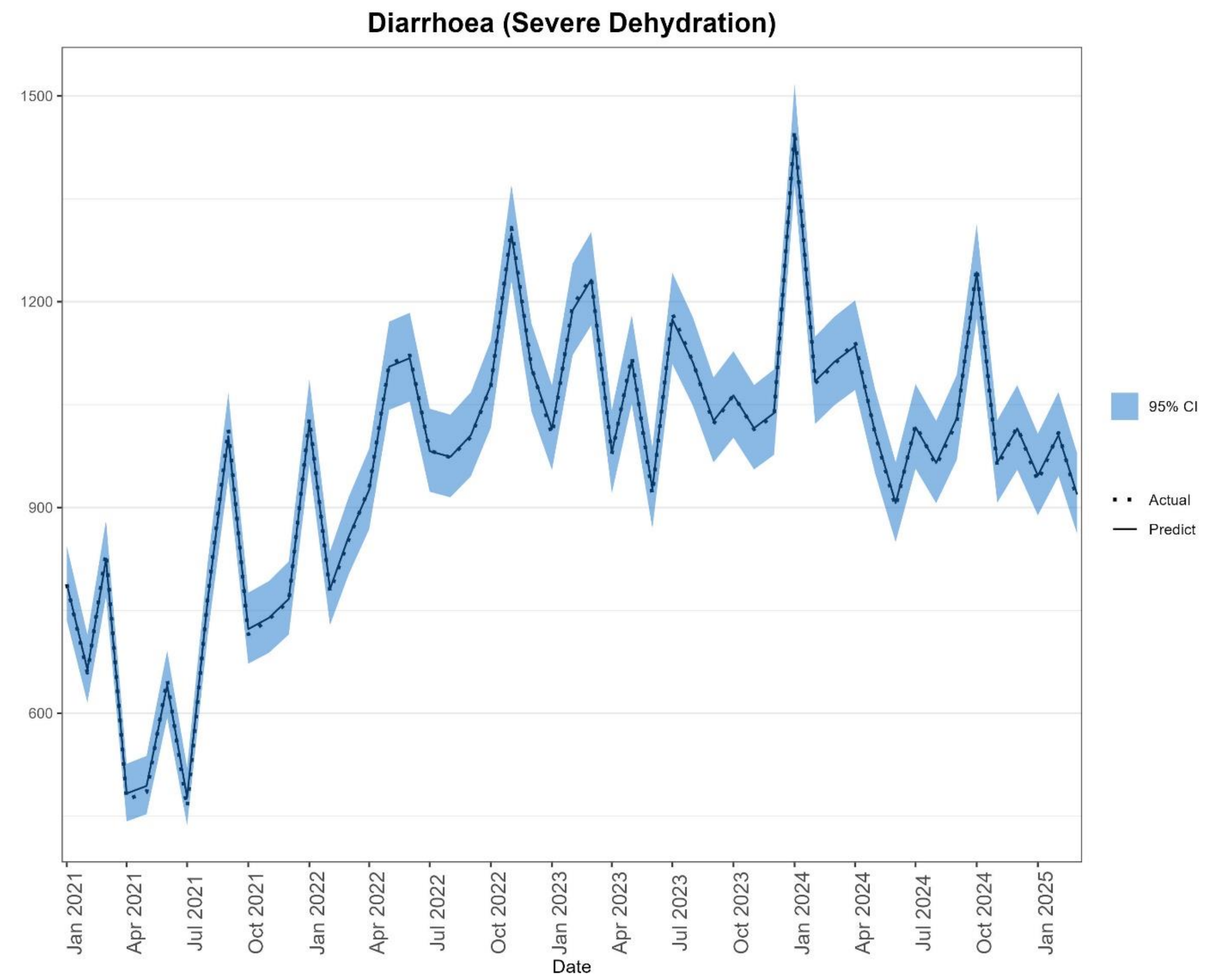

Diarrhoea (Severe Dehydration)
1500
1200
900
600
Jan 2021
Apr 2021
Jul 2021
Oct 2021
Jan 2022
Apr 2022
Jul 2022
Oct 2022
Jan 2023
Apr 2023
Jul 2023
Oct 2023
Jan 2024
Apr 2024
Jul 2024
Oct 2024
Jan 2025
Date
95% CI
Actual
Predict

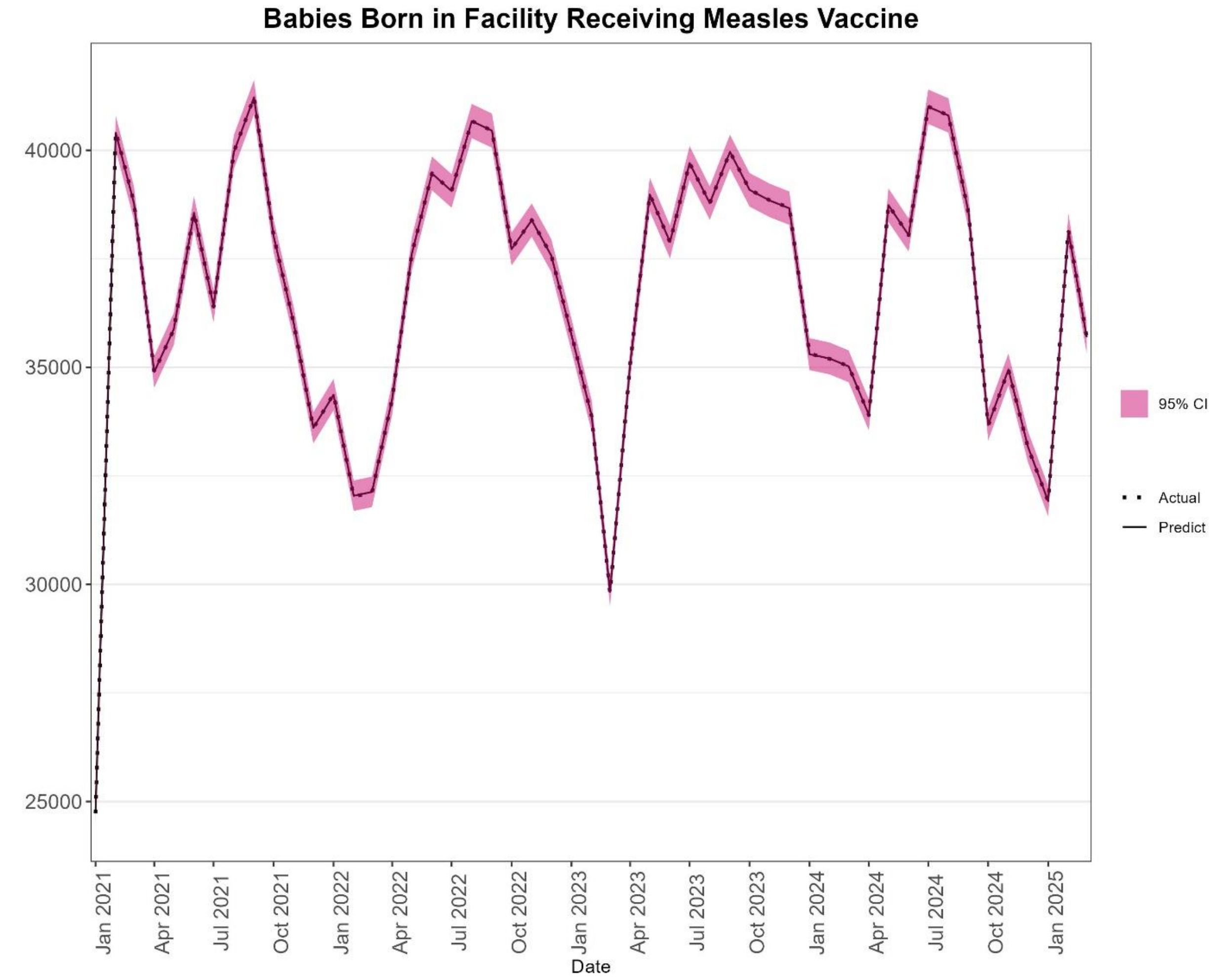

Babies Born in Facility Receiving Measles Vaccine
40000
35000
30000
25000
Jan 2021
Apr 2021
Jul 2021
Oct 2021
Jan 2022
Apr 2022
Jul 2022
Oct 2022
Jan 2023
Apr 2023
Jul 2023
Oct 2023
Jan 2024
Apr 2024
Jul 2024
Oct 2024
Jan 2025
Date
95% CI
Actual
Predict

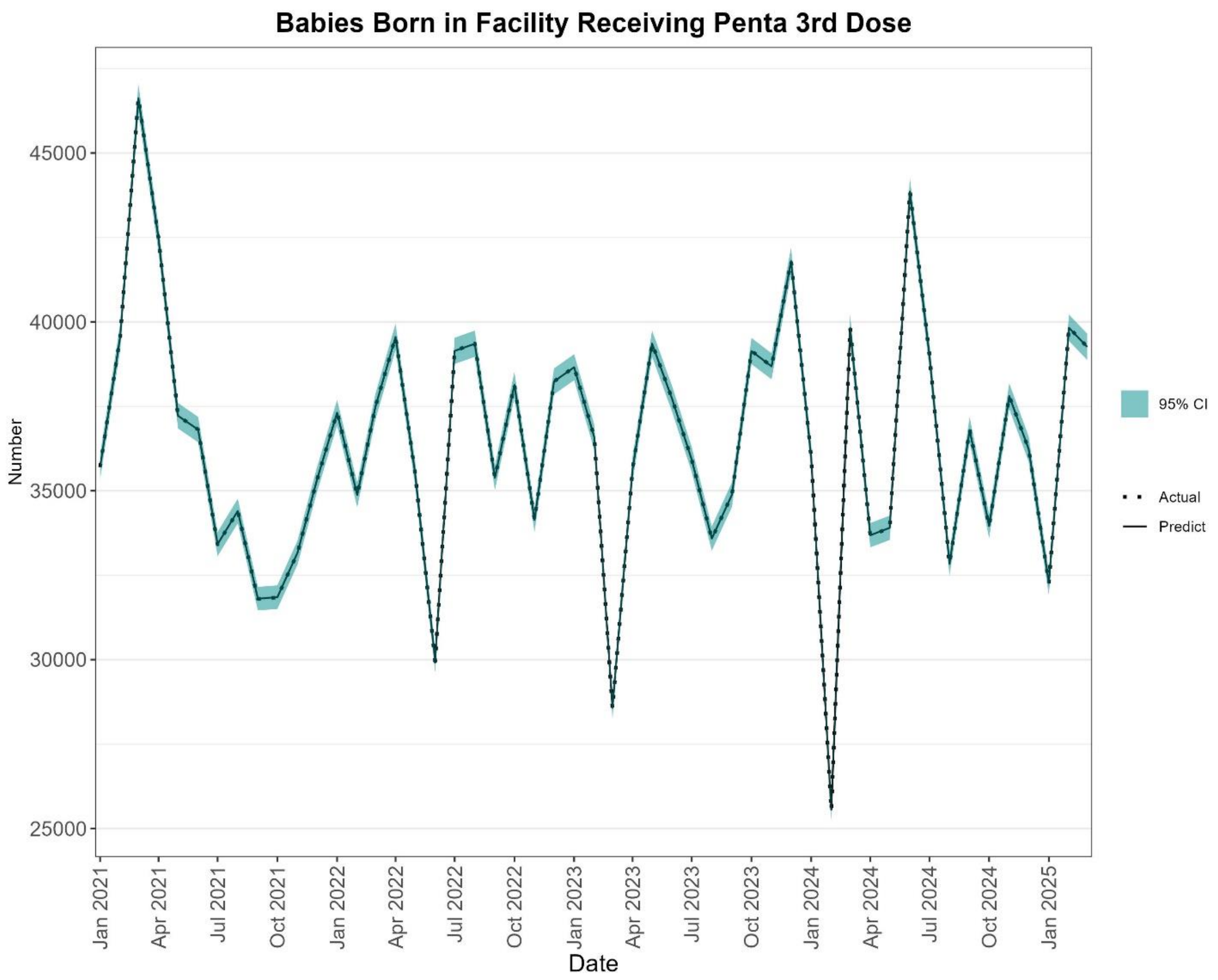
Babies Born in Facility Receiving Penta 3rd Dose
Number
45000
40000
35000
30000
25000
Jan 2021
Apr 2021
Jul 2021
Oct 2021
Jan 2022
Apr 2022
Jul 2022
Oct 2022
Jan 2023
Apr 2023
Jul 2023
Oct 2023
Jan 2024
Apr 2024
Jul 2024
Oct 2024
Jan 2025
Date
95% CI
Actual
Predict

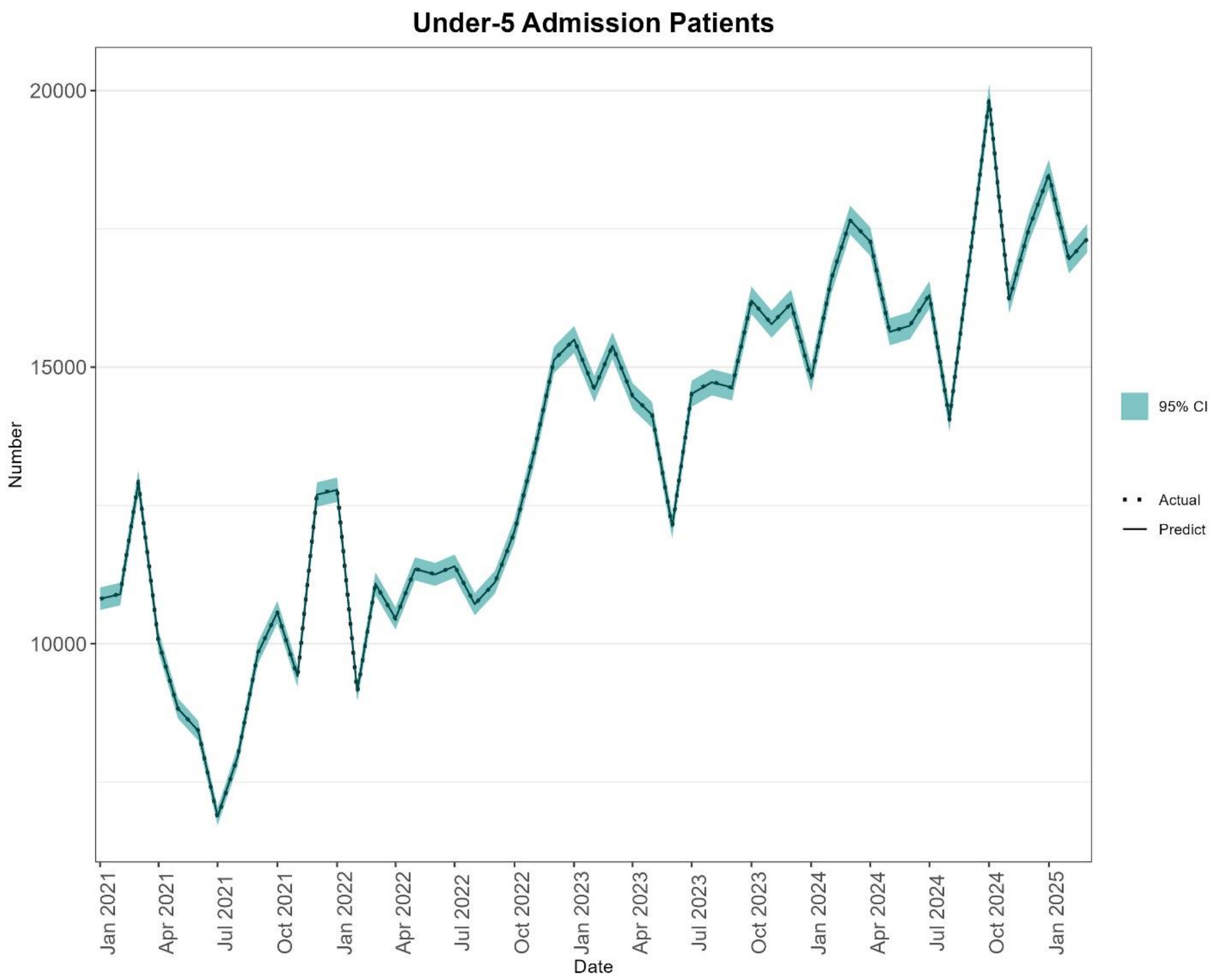
Under-5 Admission Patients
20000
15000
10000
Number
Jan 2021
Apr 2021
Jul 2021
Oct 2021
Jan 2022
Apr 2022
Jul 2022
Oct 2022
Jan 2023
Apr 2023
Jul 2023
Oct 2023
Jan 2024
Apr 2024
Jul 2024
Oct 2024
Jan 2025
Date
95% CI
Actual
Predict

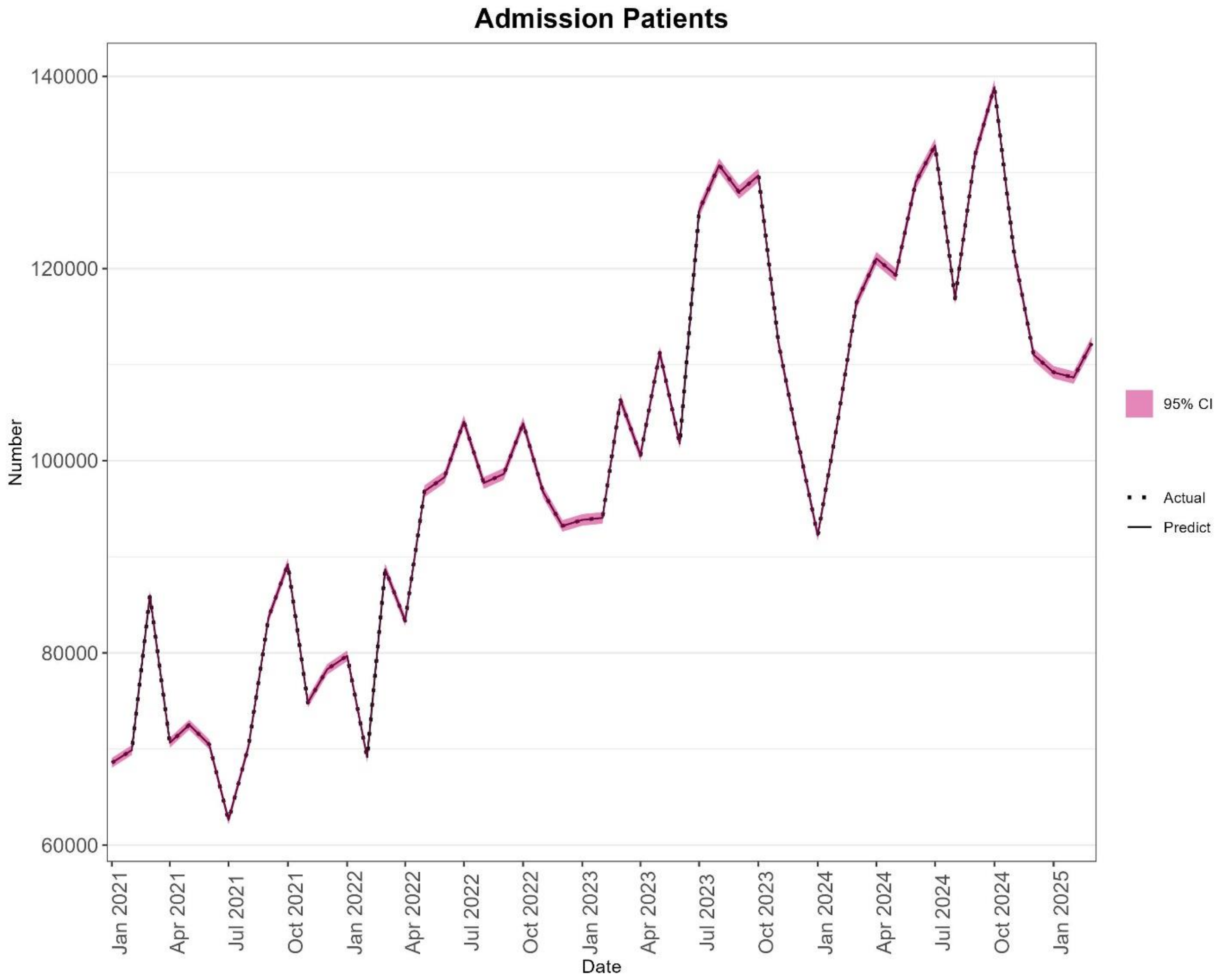

Admission Patients
140000
120000
100000
80000
60000
Number
Jan 2021
Apr 2021
Jul 2021
Oct 2021
Jan 2022
Apr 2022
Jul 2022
Oct 2022
Jan 2023
Apr 2023
Jul 2023
Oct 2023
Jan 2024
Apr 2024
Jul 2024
Oct 2024
Jan 2025
Date
95% CI
Actual
Predict

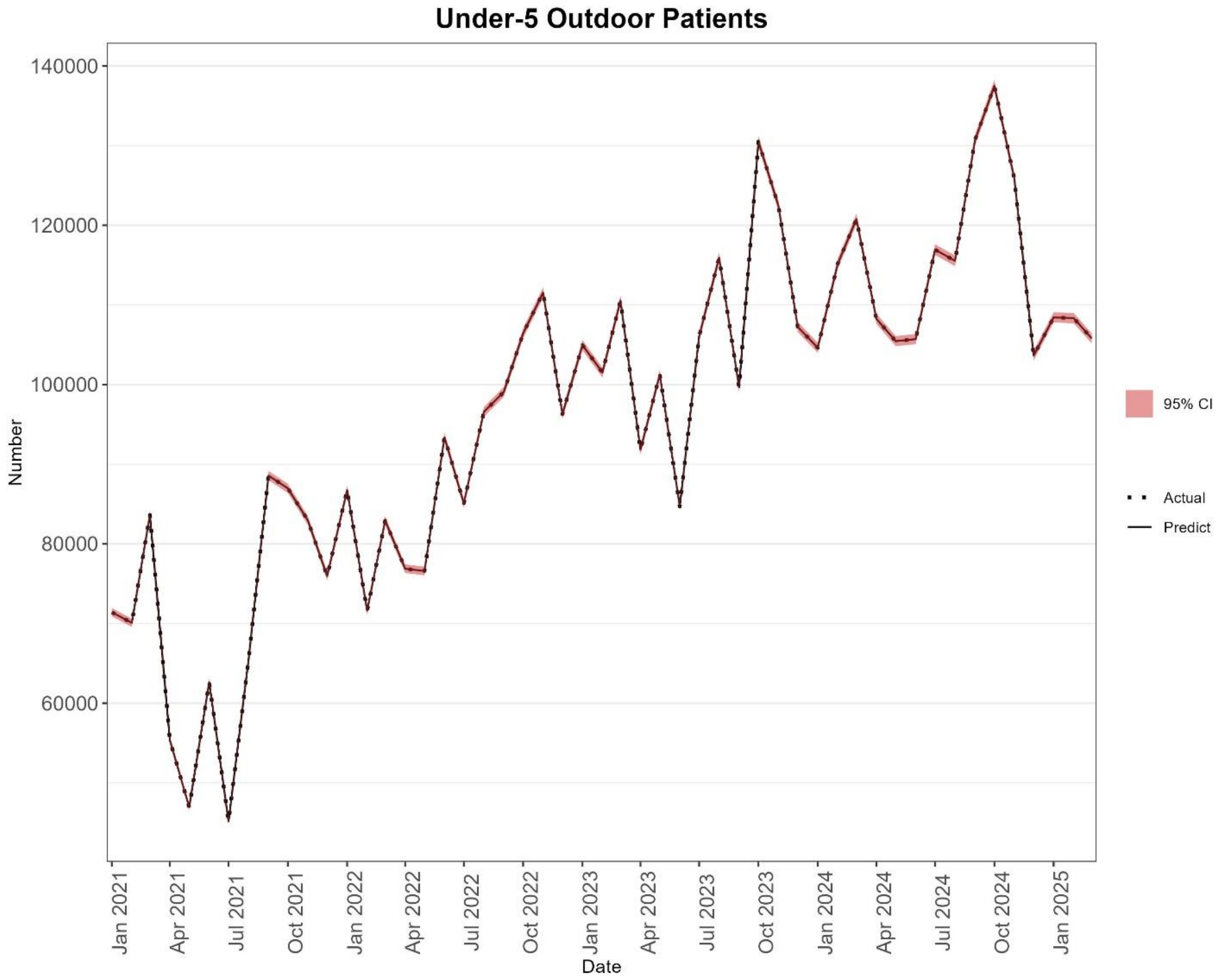
Under-5 Outdoor Patients
140000
120000
100000
80000
60000
Number
Jan 2021
Apr 2021
Jul 2021
Oct 2021
Jan 2022
Apr 2022
Jul 2022
Oct 2022
Jan 2023
Apr 2023
Jul 2023
Oct 2023
Jan 2024
Apr 2024
Jul 2024
Oct 2024
Jan 2025
Date
95% CI
Actual
Predict

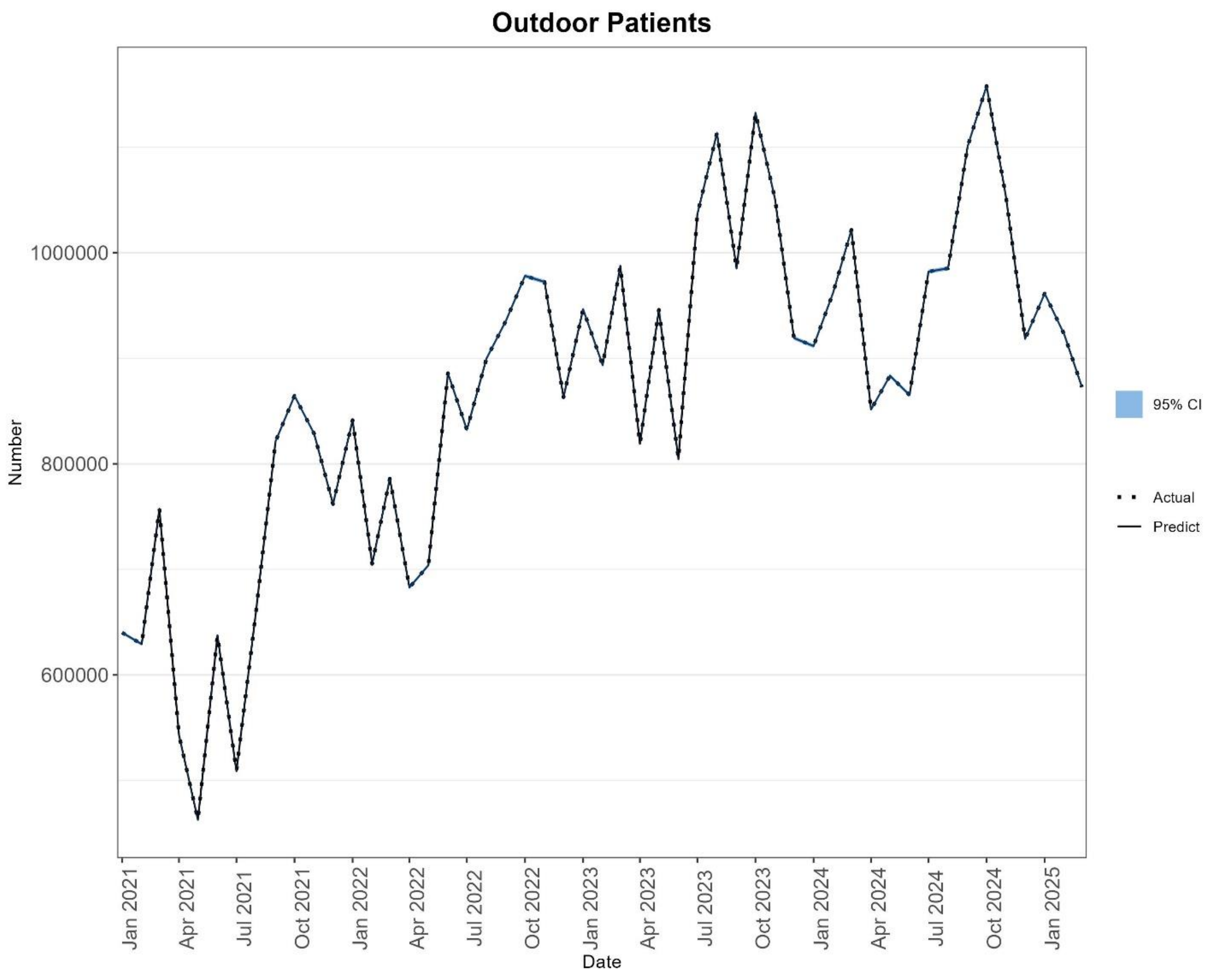

Outdoor Patients
1000000
800000
600000
Number
Jan 2021
Apr 2021
Jul 2021
Oct 2021
Jan 2022
Apr 2022
Jul 2022
Oct 2022
Jan 2023
Apr 2023
Jul 2023
Oct 2023
Jan 2024
Apr 2024
Jul 2024
Oct 2024
Jan 2025
Date
95% CI
Actual
Predict

Rangpur

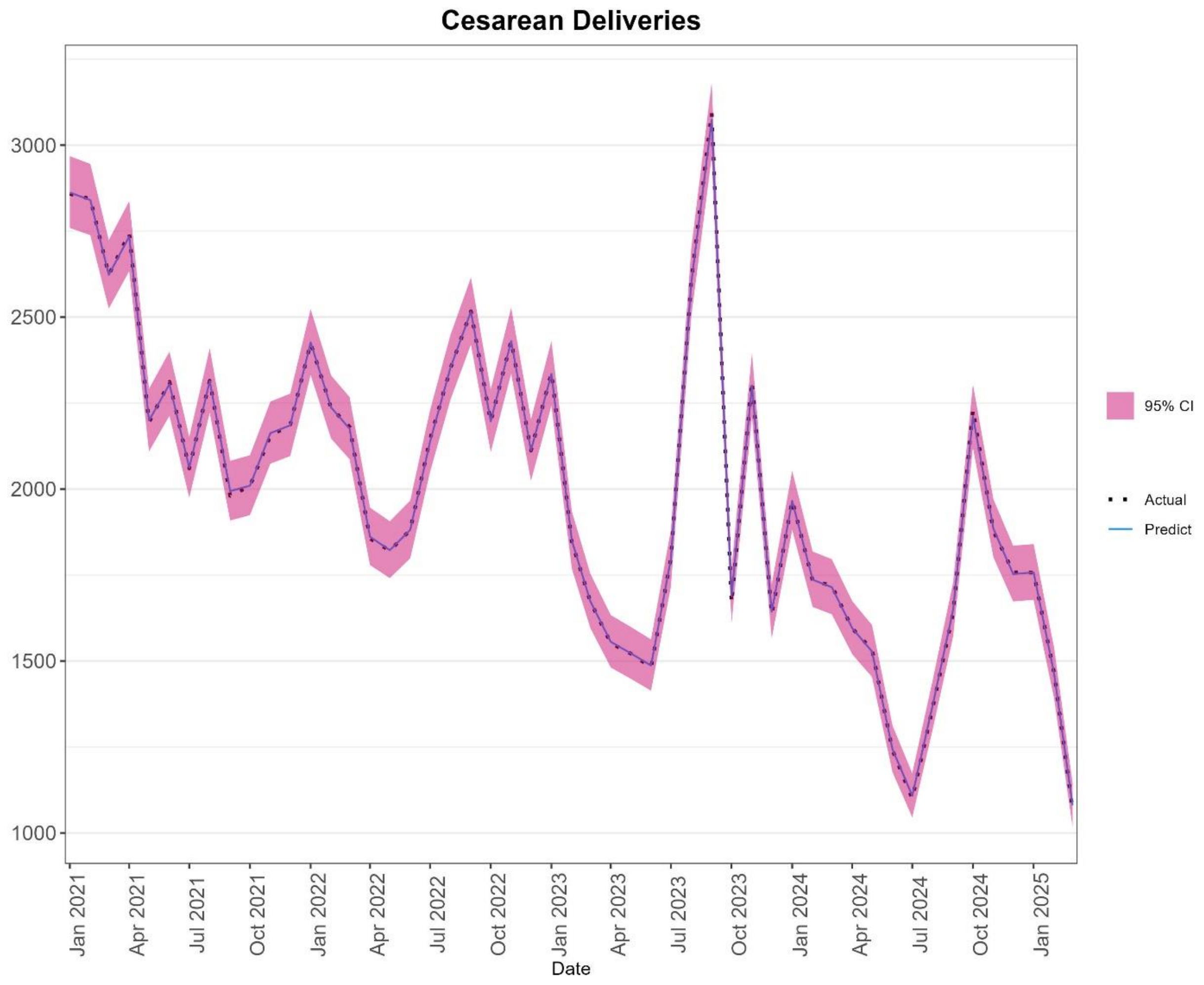
Cesarean Deliveries
3000
2500
2000
1500
1000
Jan 2021
Apr 2021
Jul 2021
Oct 2021
Jan 2022
Apr 2022
Jul 2022
Oct 2022
Jan 2023
Apr 2023
Jul 2023
Oct 2023
Jan 2024
Apr 2024
Jul 2024
Oct 2024
Jan 2025
Date
95% CI
Actual
Predict

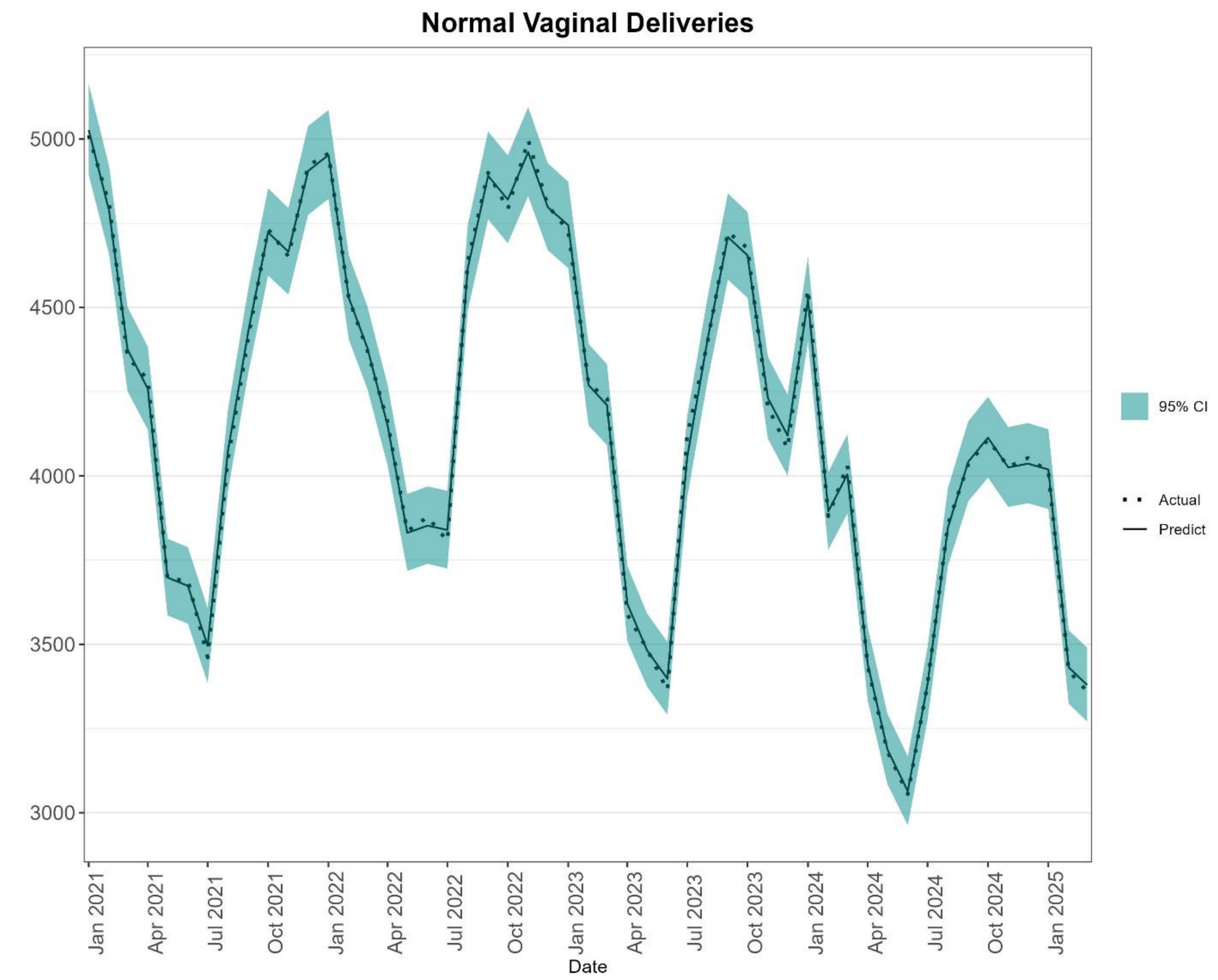

Normal Vaginal Deliveries
5000
4500
4000
3500
3000
Jan 2021
Apr 2021
Jul 2021
Oct 2021
Jan 2022
Apr 2022
Jul 2022
Oct 2022
Jan 2023
Apr 2023
Jul 2023
Oct 2023
Jan 2024
Apr 2024
Jul 2024
Oct 2024
Jan 2025
Date
95% CI
Actual
Predict

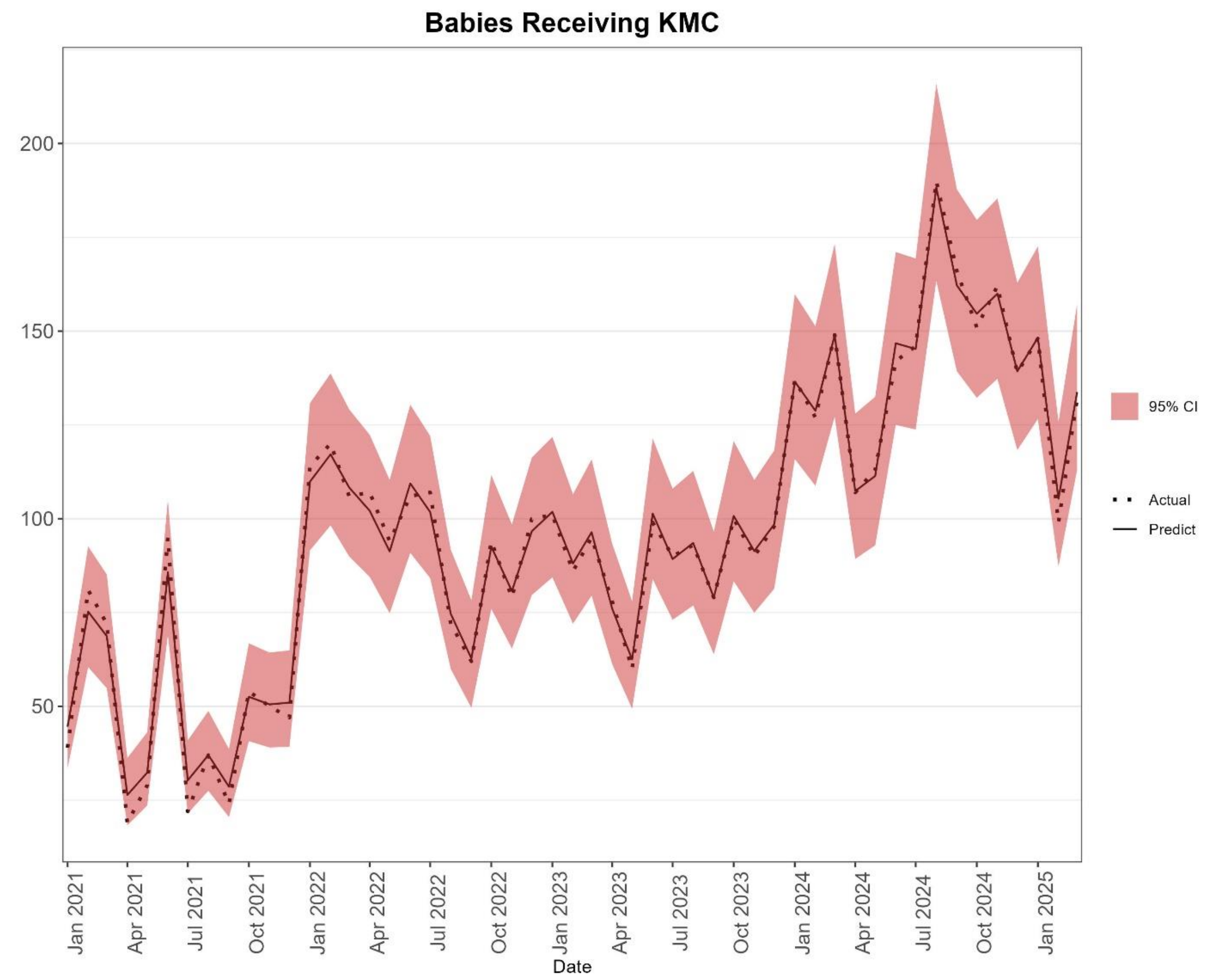

Babies Receiving KMC
200
150
100
50
Jan 2021
Apr 2021
Jul 2021
Oct 2021
Jan 2022
Apr 2022
Jul 2022
Oct 2022
Jan 2023
Apr 2023
Jul 2023
Oct 2023
Jan 2024
Apr 2024
Jul 2024
Oct 2024
Jan 2025
Date
95% CI
Actual
Predict

Low Birth Weight Babies (<2500 g)

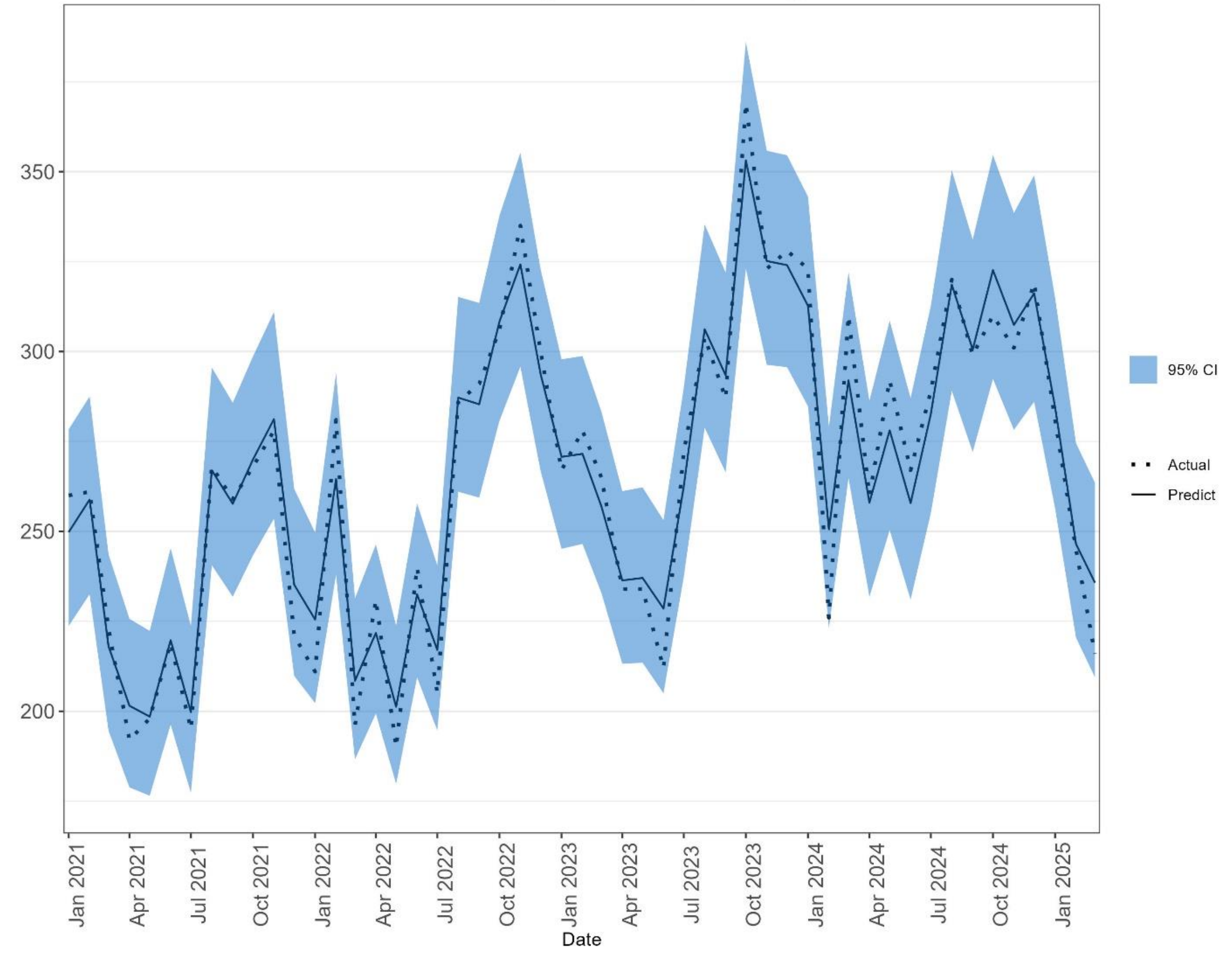

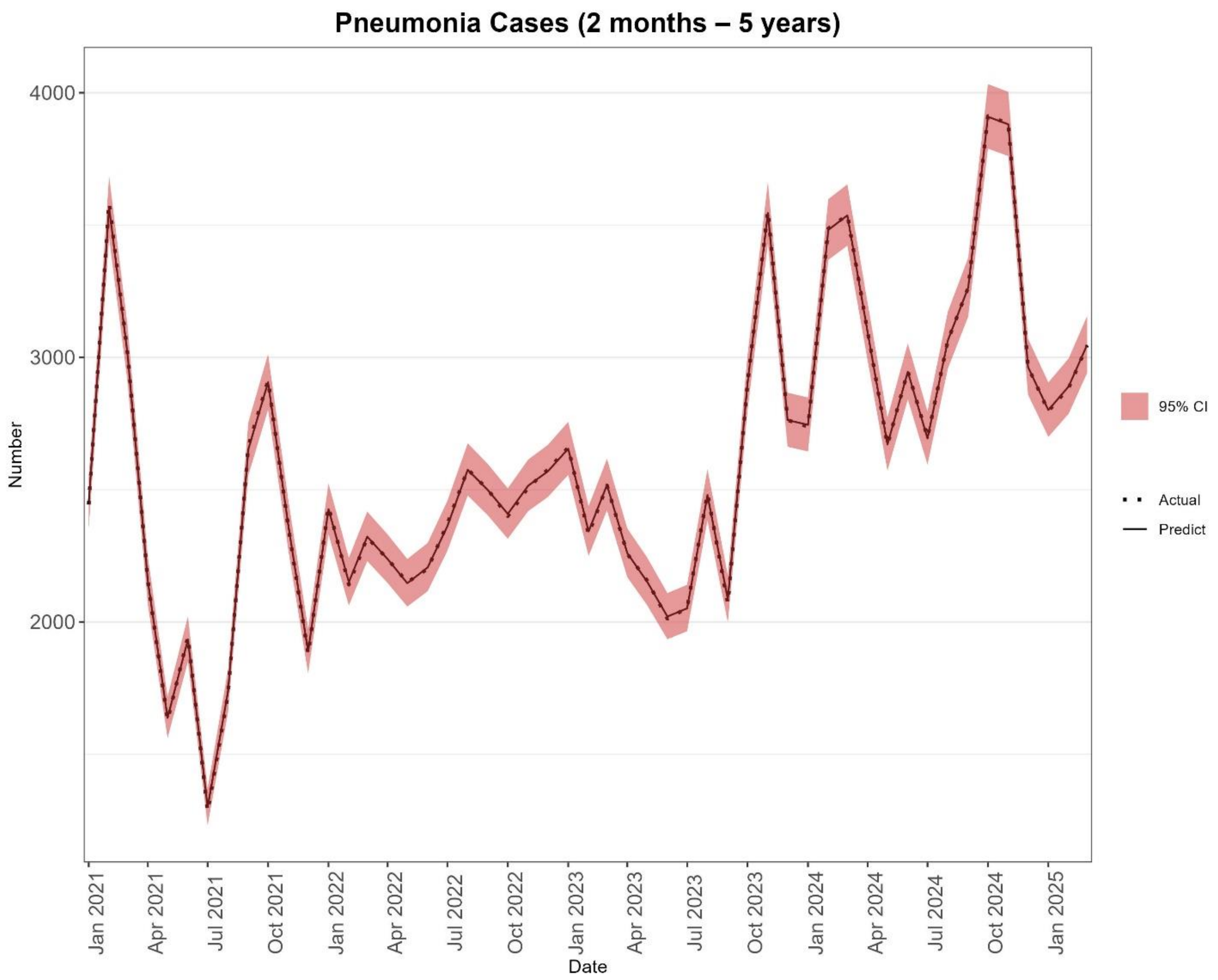

Pneumonia Cases (2 months – 5 years)
4000
3000
2000
Number
Jan 2021
Apr 2021
Jul 2021
Oct 2021
Jan 2022
Apr 2022
Jul 2022
Oct 2022
Jan 2023
Apr 2023
Jul 2023
Oct 2023
Jan 2024
Apr 2024
Jul 2024
Oct 2024
Jan 2025
Date
95% CI
Actual
Predict

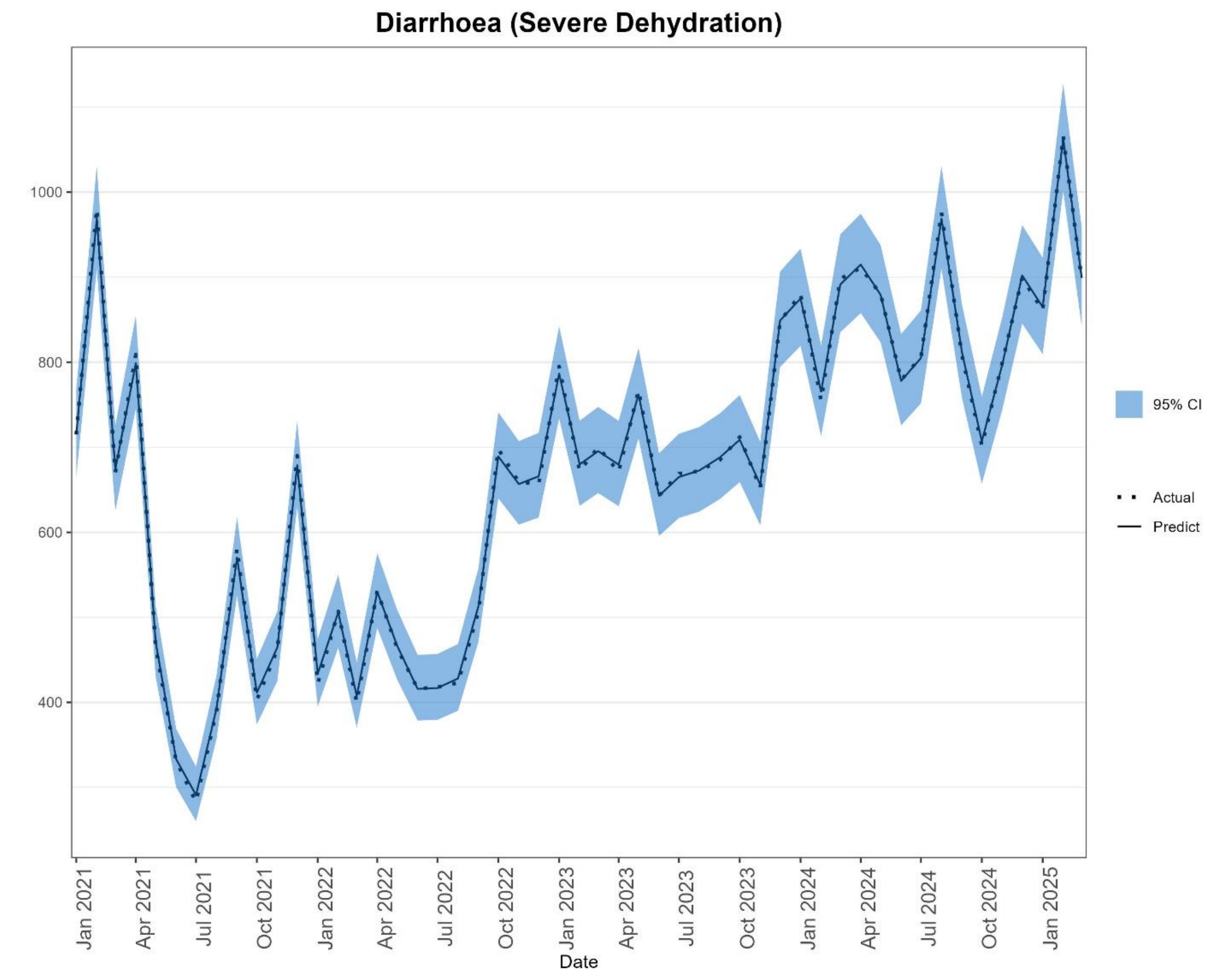
Diarrhoea (Severe Dehydration)
1000
800
600
400
Jan 2021
Apr 2021
Jul 2021
Oct 2021
Jan 2022
Apr 2022
Jul 2022
Oct 2022
Jan 2023
Apr 2023
Jul 2023
Oct 2023
Jan 2024
Apr 2024
Jul 2024
Oct 2024
Jan 2025
Date
95% CI
Actual
Predict

Babies Born in Facility Receiving Measles Vaccine

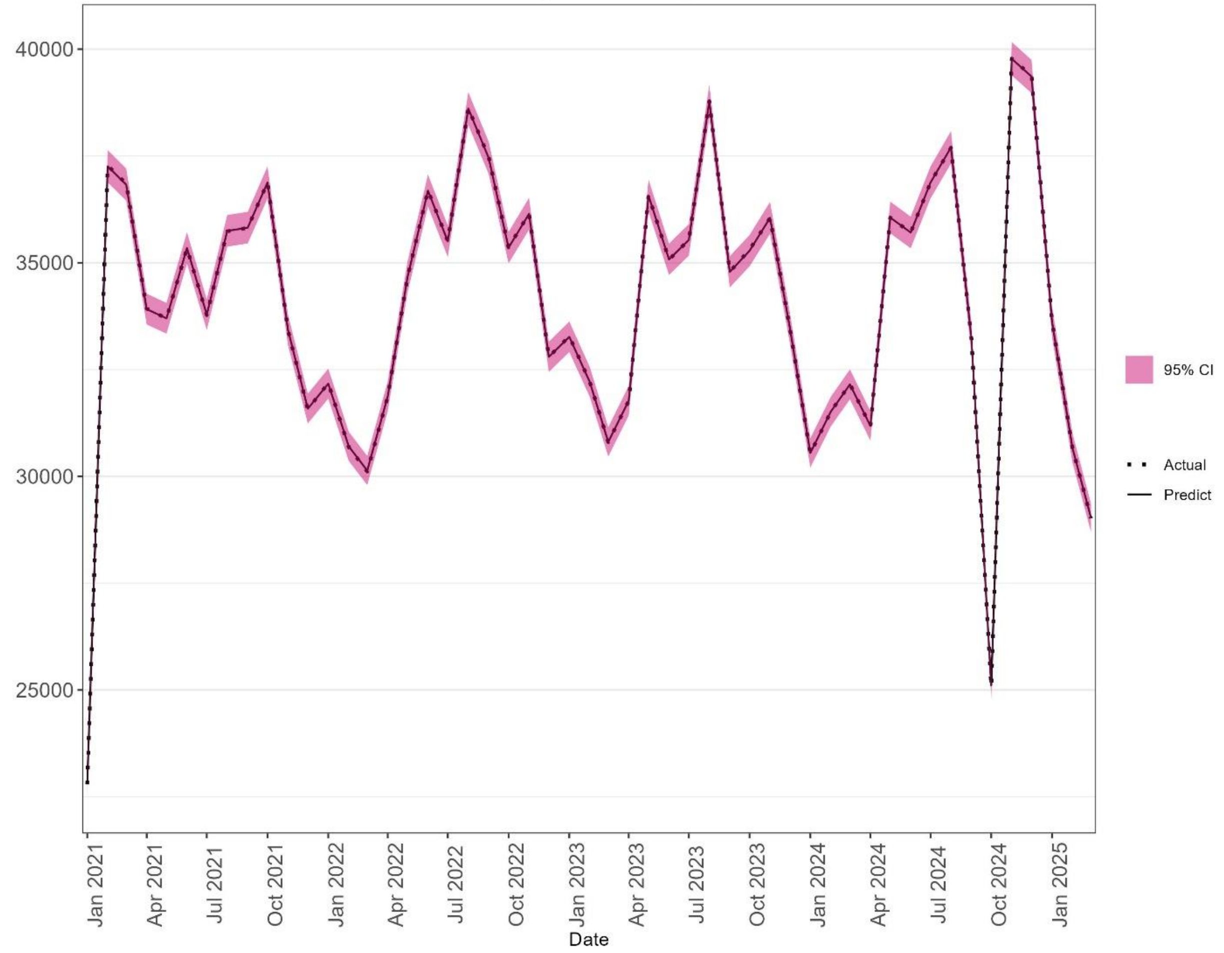

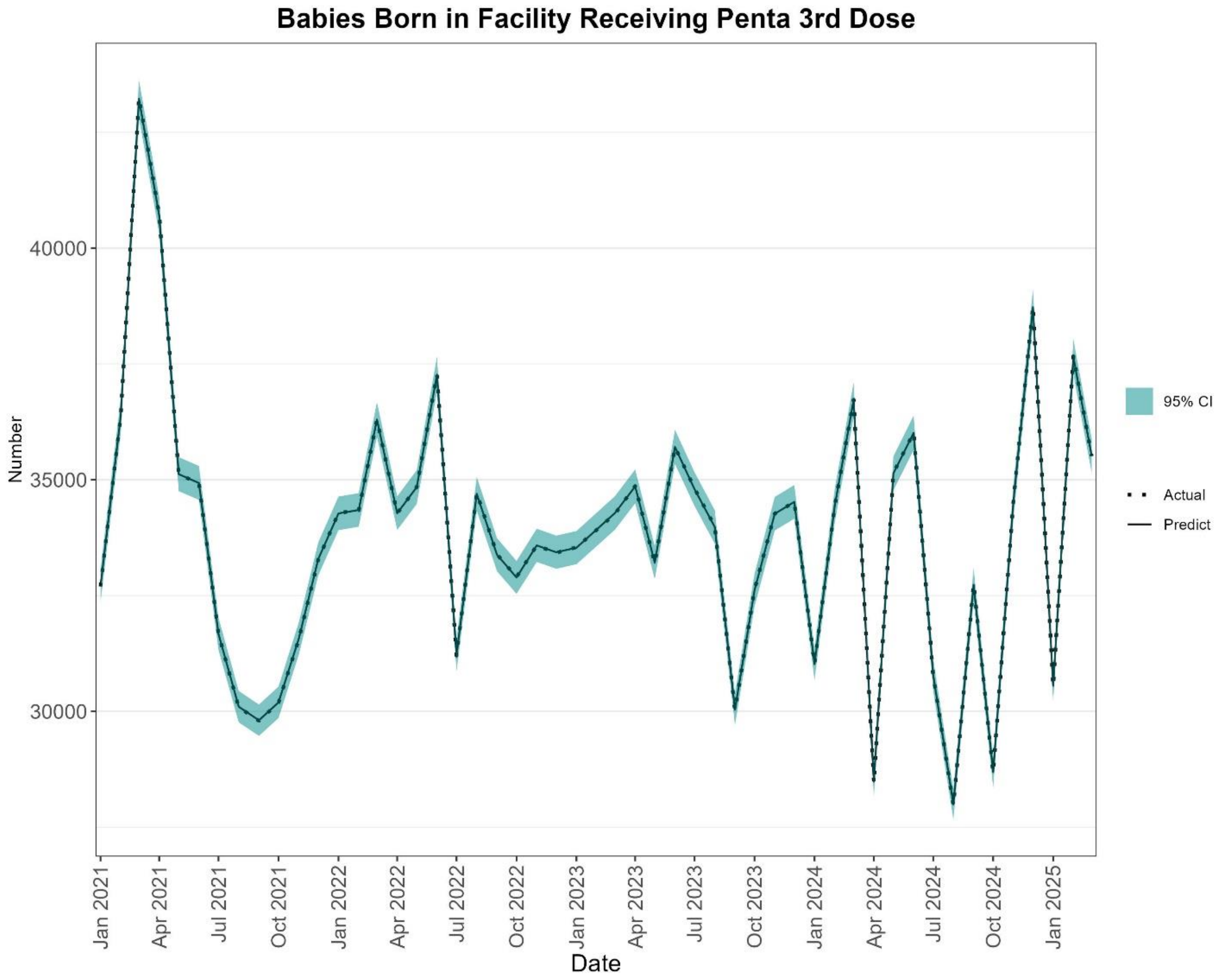

Babies Born in Facility Receiving Penta 3rd Dose
Number
40000
35000
30000
Jan 2021
Apr 2021
Jul 2021
Oct 2021
Jan 2022
Apr 2022
Jul 2022
Oct 2022
Jan 2023
Apr 2023
Jul 2023
Oct 2023
Jan 2024
Apr 2024
Jul 2024
Oct 2024
Jan 2025
Date
95% CI
Actual
Predict

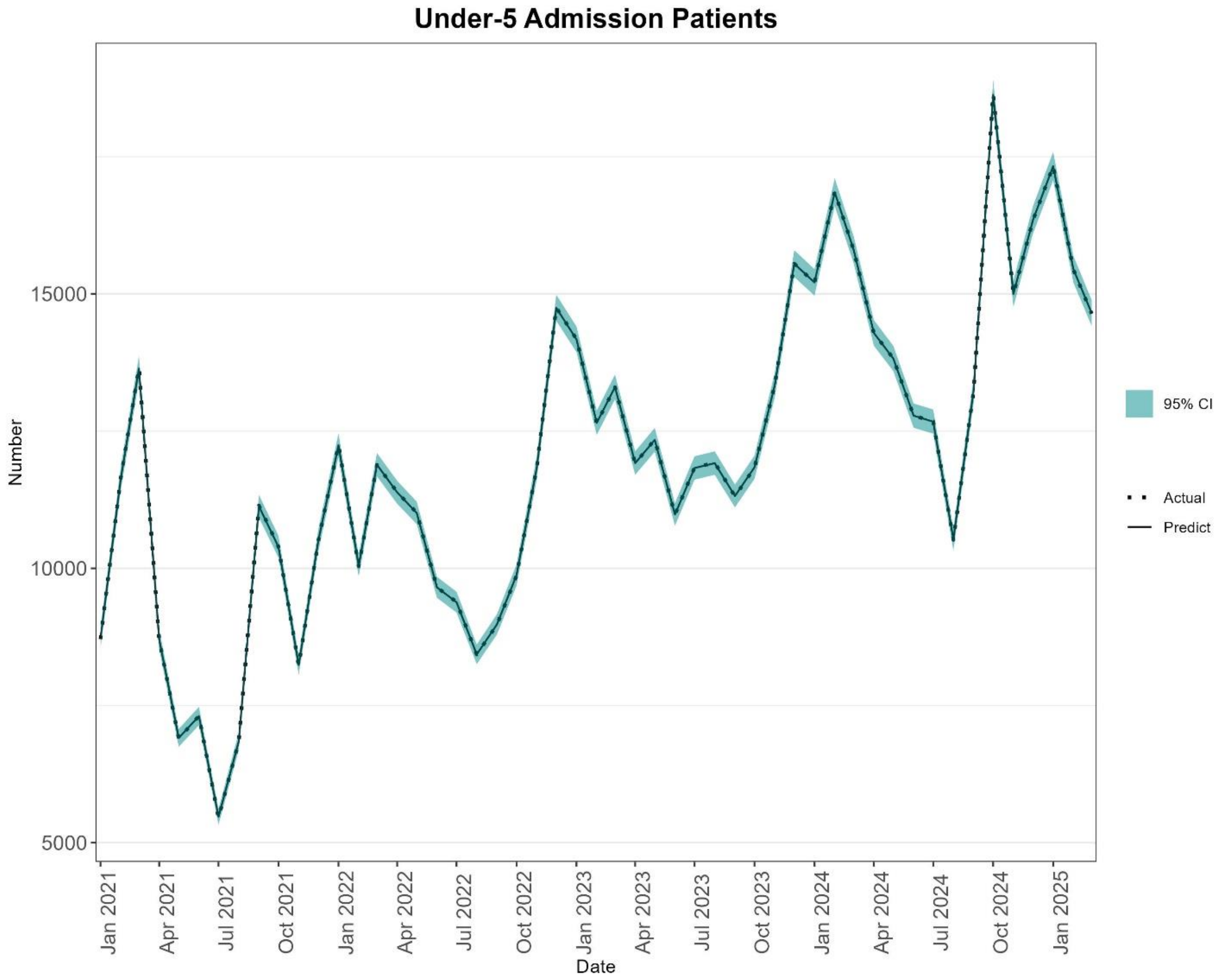

Under-5 Admission Patients
Number
15000
10000
5000
Jan 2021
Apr 2021
Jul 2021
Oct 2021
Jan 2022
Apr 2022
Jul 2022
Oct 2022
Jan 2023
Apr 2023
Jul 2023
Oct 2023
Jan 2024
Apr 2024
Jul 2024
Oct 2024
Jan 2025
Date
95% CI
Actual
Predict

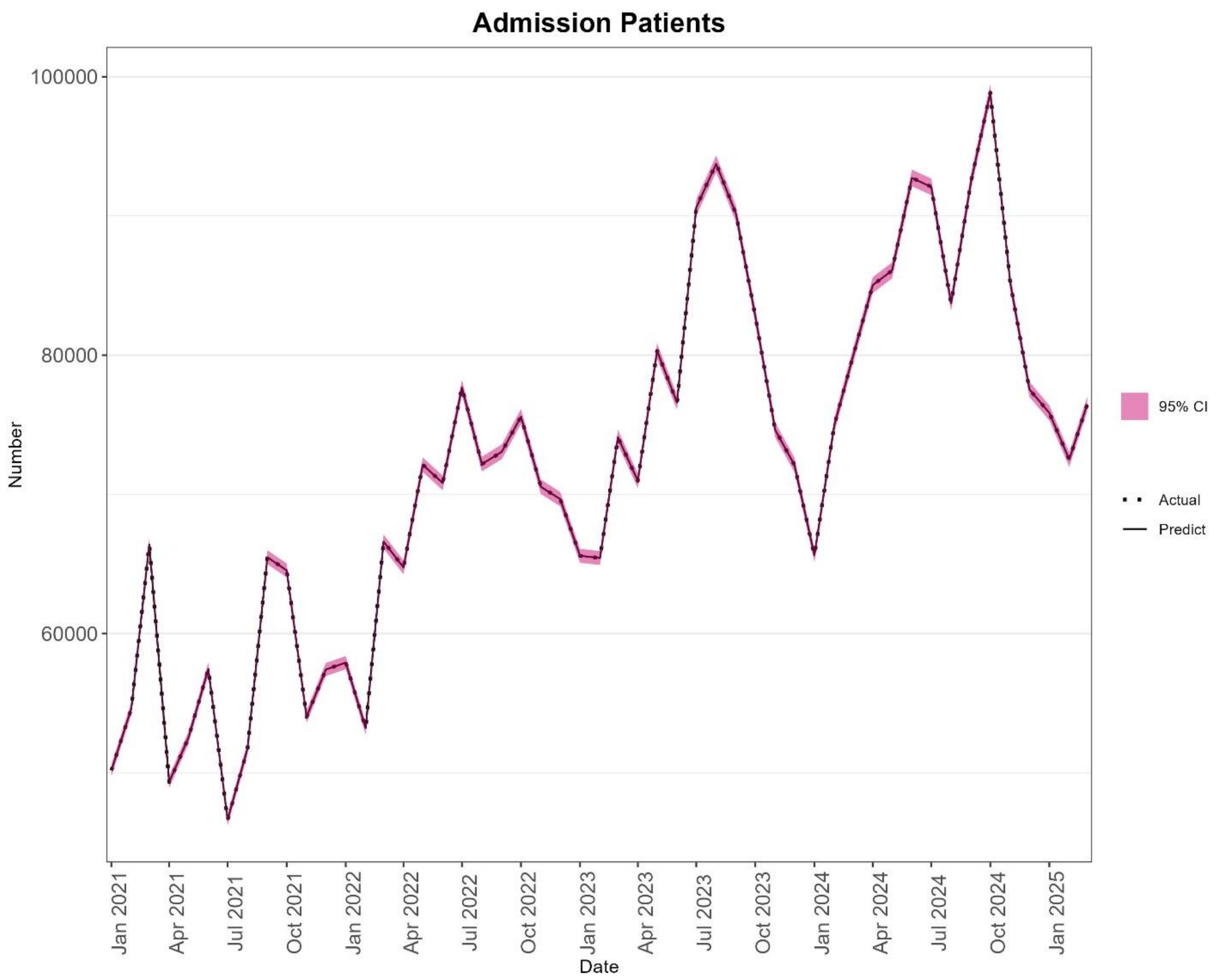

Admission Patients
100000
80000
60000
Number
Jan 2021
Apr 2021
Jul 2021
Oct 2021
Jan 2022
Apr 2022
Jul 2022
Oct 2022
Jan 2023
Apr 2023
Jul 2023
Oct 2023
Jan 2024
Apr 2024
Jul 2024
Oct 2024
Jan 2025
Date
95% CI
Actual
Predict

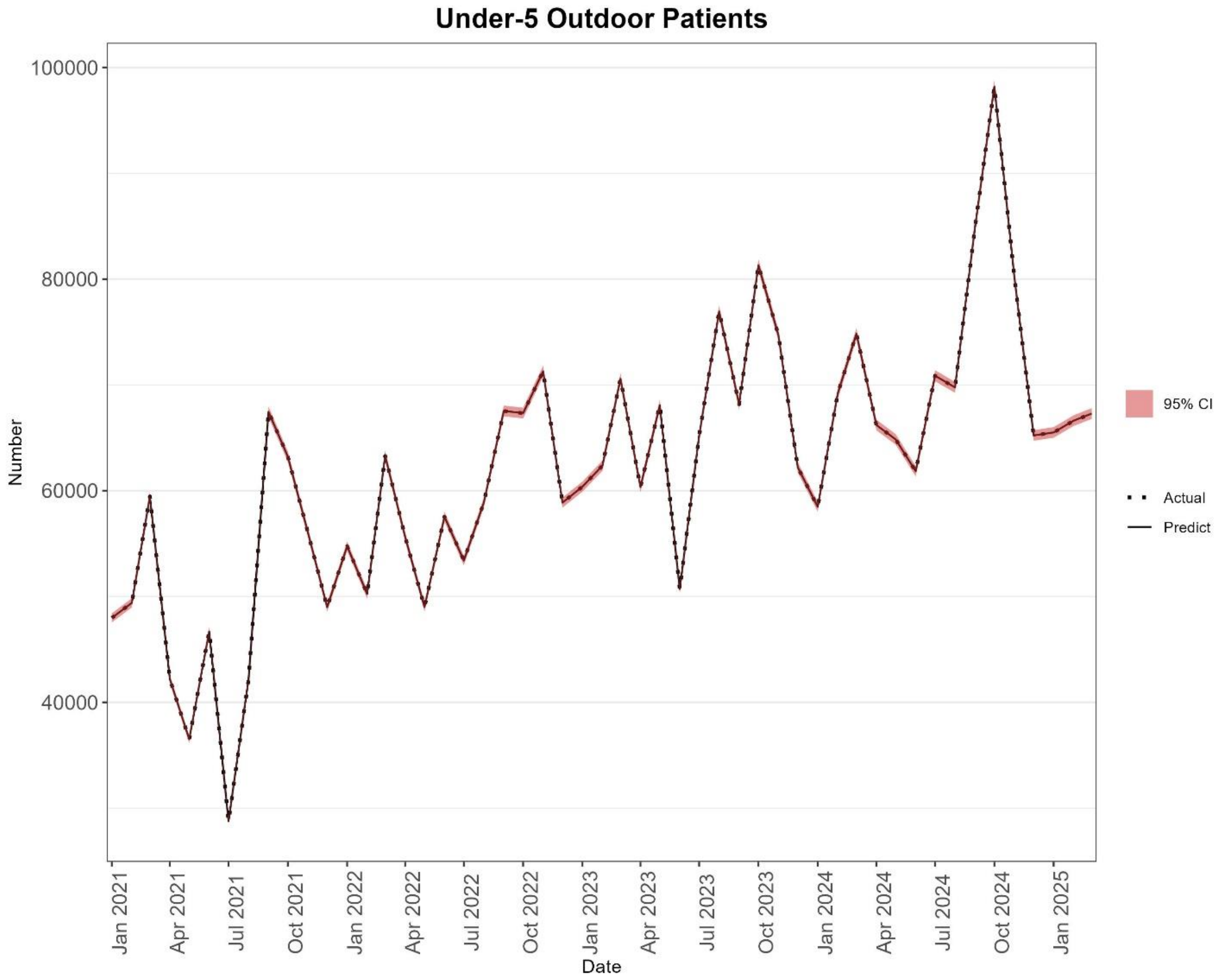
Under-5 Outdoor Patients
100000
80000
60000
40000
Number
Jan 2021
Apr 2021
Jul 2021
Oct 2021
Jan 2022
Apr 2022
Jul 2022
Oct 2022
Jan 2023
Apr 2023
Jul 2023
Oct 2023
Jan 2024
Apr 2024
Jul 2024
Oct 2024
Jan 2025
Date
95% CI
Actual
Predict

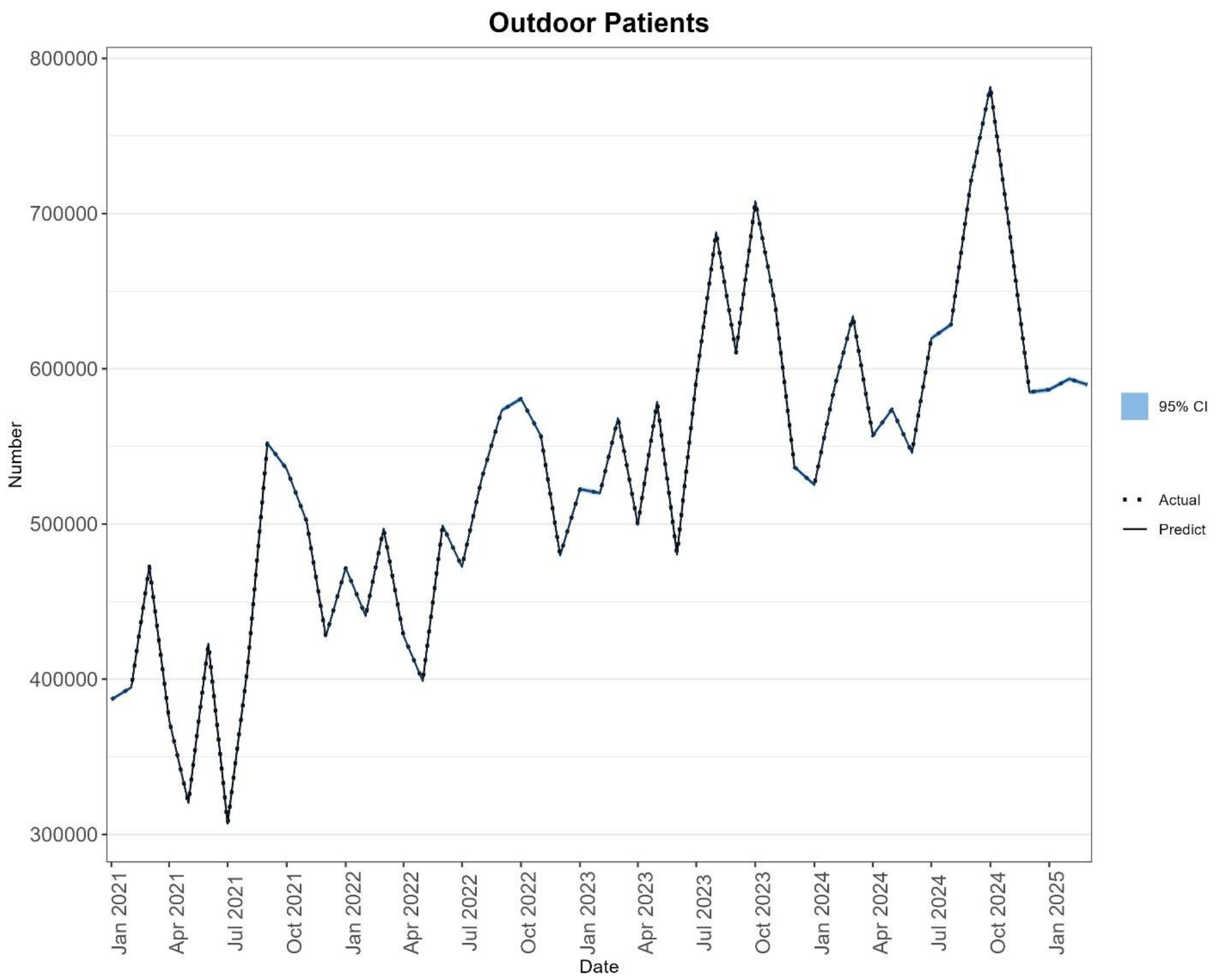

Outdoor Patients
800000
700000
600000
500000
400000
300000
Number
Jan 2021
Apr 2021
Jul 2021
Oct 2021
Jan 2022
Apr 2022
Jul 2022
Oct 2022
Jan 2023
Apr 2023
Jul 2023
Oct 2023
Jan 2024
Apr 2024
Jul 2024
Oct 2024
Jan 2025
Date
95% CI
Actual
Predict

Sylhet

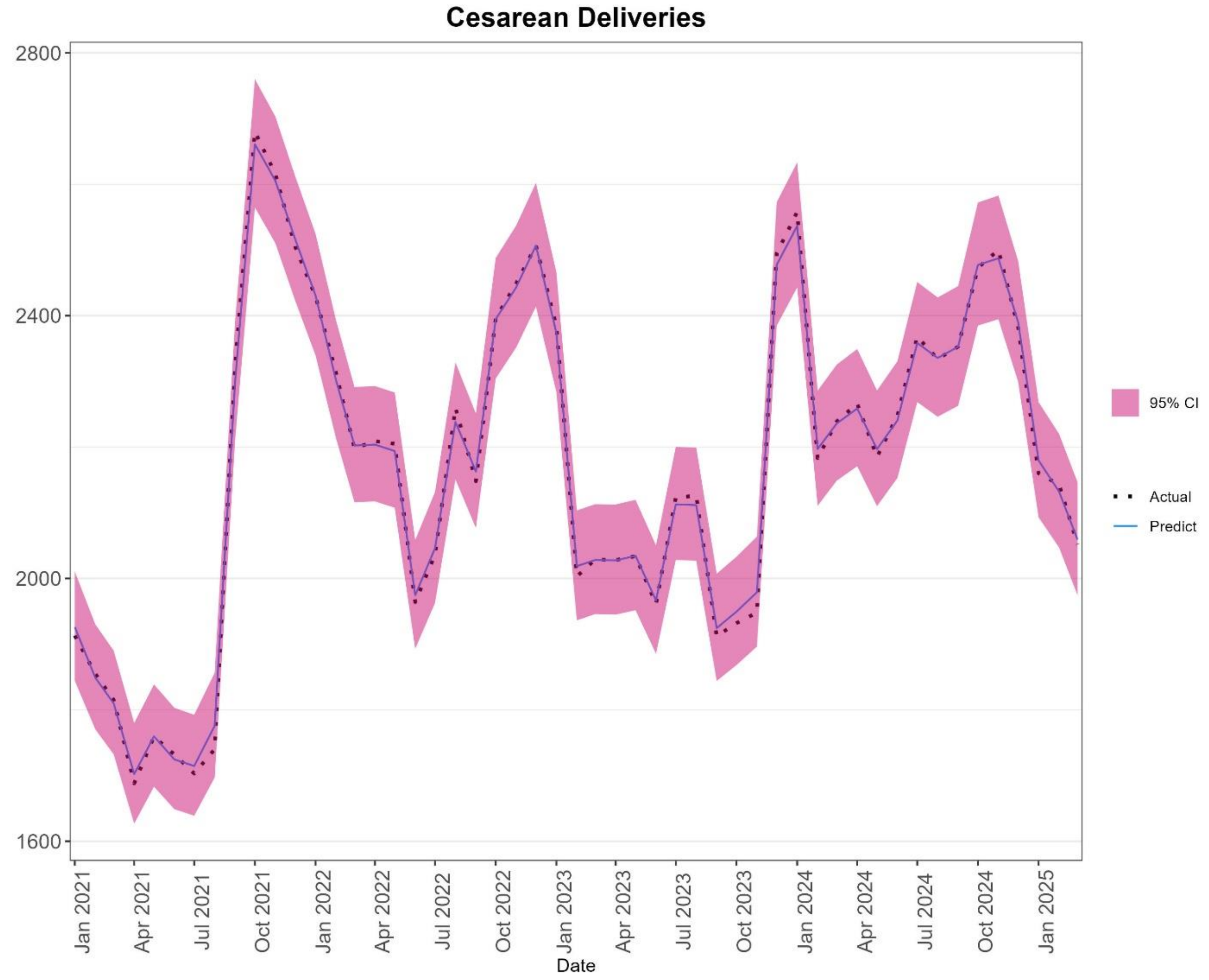
Cesarean Deliveries
2800
2400
2000
1600
Jan 2021
Apr 2021
Jul 2021
Oct 2021
Jan 2022
Apr 2022
Jul 2022
Oct 2022
Jan 2023
Apr 2023
Jul 2023
Oct 2023
Jan 2024
Apr 2024
Jul 2024
Oct 2024
Jan 2025
Date
95% CI
Actual
Predict

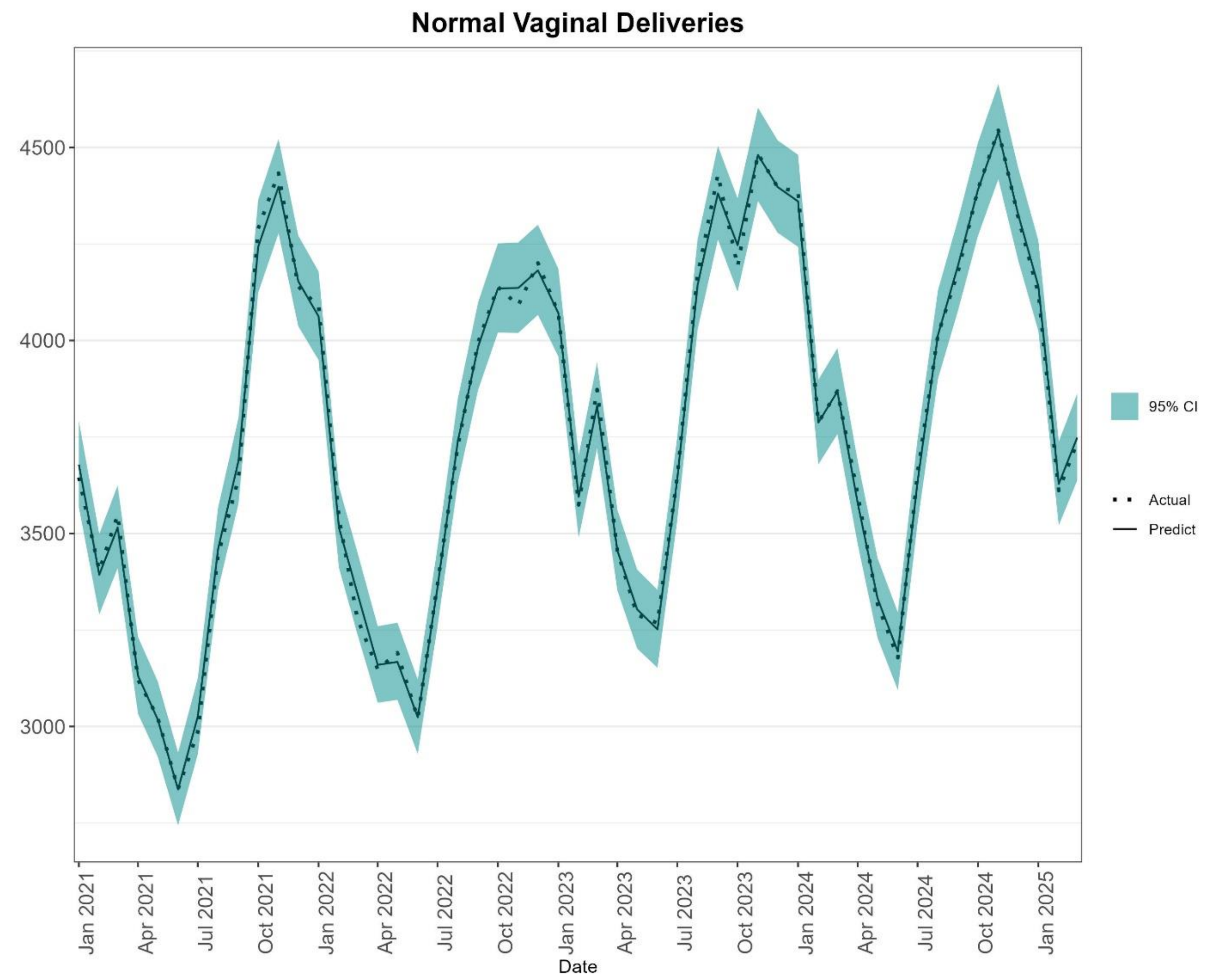

Normal Vaginal Deliveries
4500
4000
3500
3000
Jan 2021
Apr 2021
Jul 2021
Oct 2021
Jan 2022
Apr 2022
Jul 2022
Oct 2022
Jan 2023
Apr 2023
Jul 2023
Oct 2023
Jan 2024
Apr 2024
Jul 2024
Oct 2024
Jan 2025
Date
95% CI
Actual
Predict

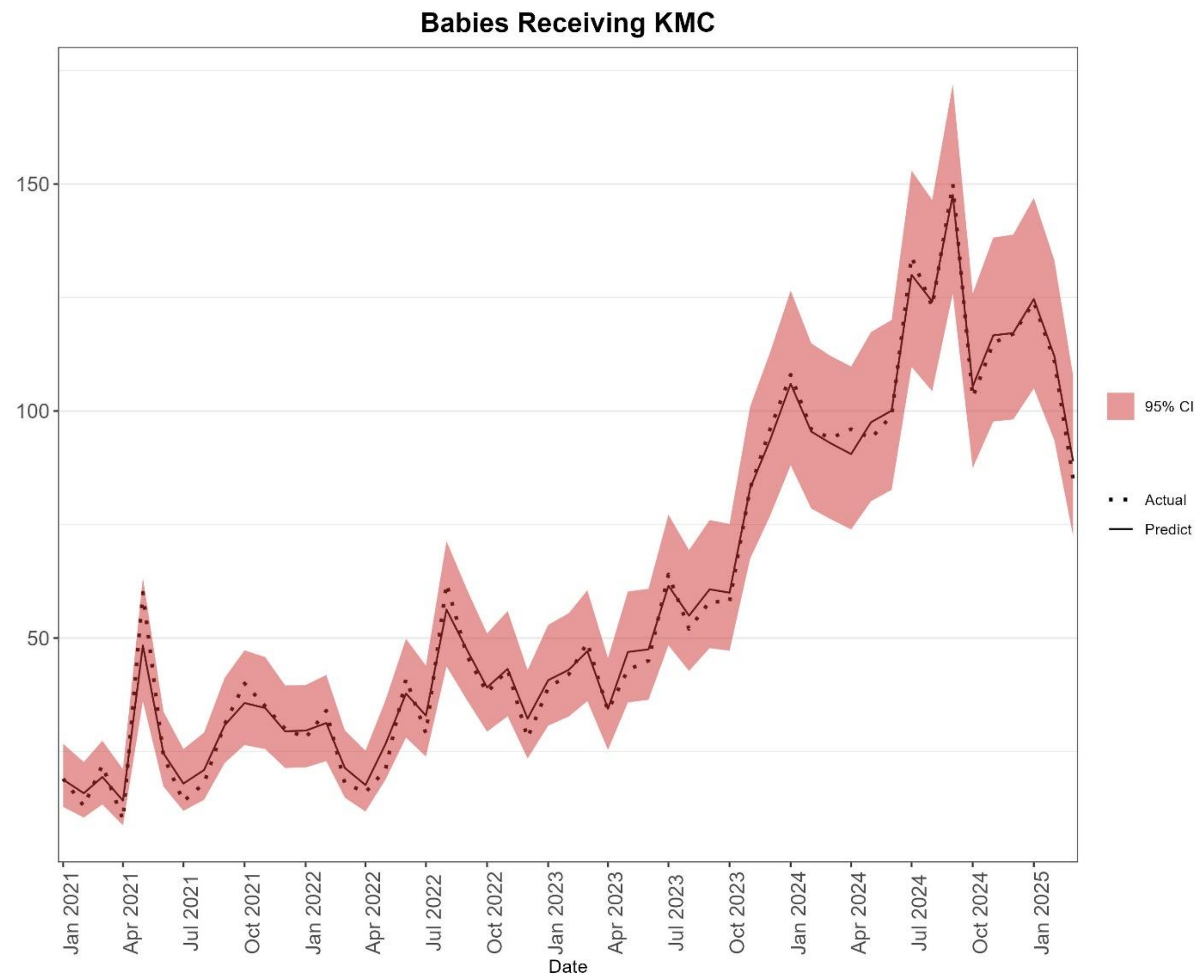

Babies Receiving KMC
150
100
50
Jan 2021
Apr 2021
Jul 2021
Oct 2021
Jan 2022
Apr 2022
Jul 2022
Oct 2022
Jan 2023
Apr 2023
Jul 2023
Oct 2023
Jan 2024
Apr 2024
Jul 2024
Oct 2024
Jan 2025
Date
95% CI
Actual
Predict

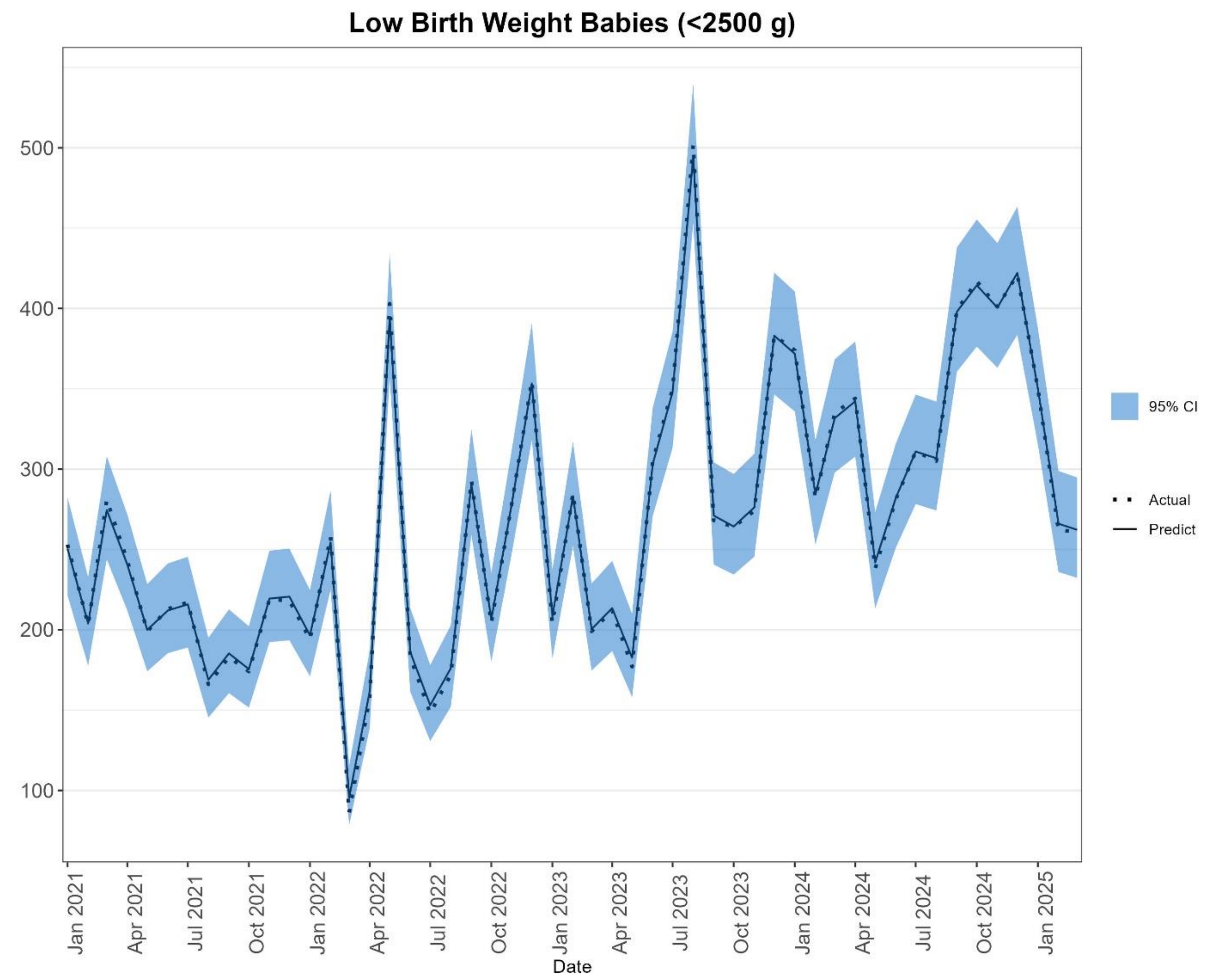

Low Birth Weight Babies (<2500 g)
500
400
300
200
100
Jan 2021
Apr 2021
Jul 2021
Oct 2021
Jan 2022
Apr 2022
Jul 2022
Oct 2022
Jan 2023
Apr 2023
Jul 2023
Oct 2023
Jan 2024
Apr 2024
Jul 2024
Oct 2024
Jan 2025
Date
95% CI
Actual
Predict

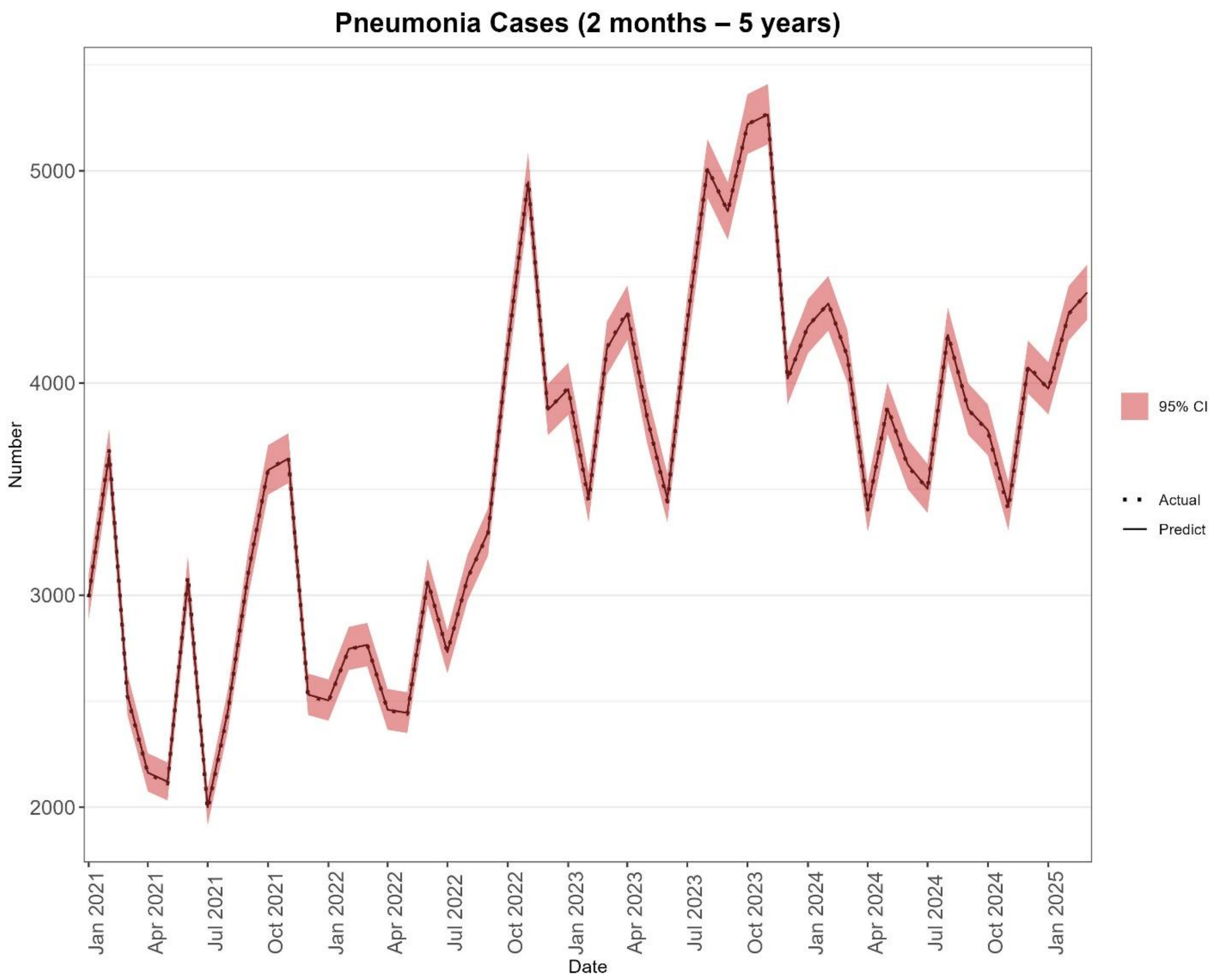

Pneumonia Cases (2 months – 5 years)
Number
5000
4000
3000
2000
Jan 2021
Apr 2021
Jul 2021
Oct 2021
Jan 2022
Apr 2022
Jul 2022
Oct 2022
Jan 2023
Apr 2023
Jul 2023
Oct 2023
Jan 2024
Apr 2024
Jul 2024
Oct 2024
Jan 2025
Date
95% CI
Actual
Predict

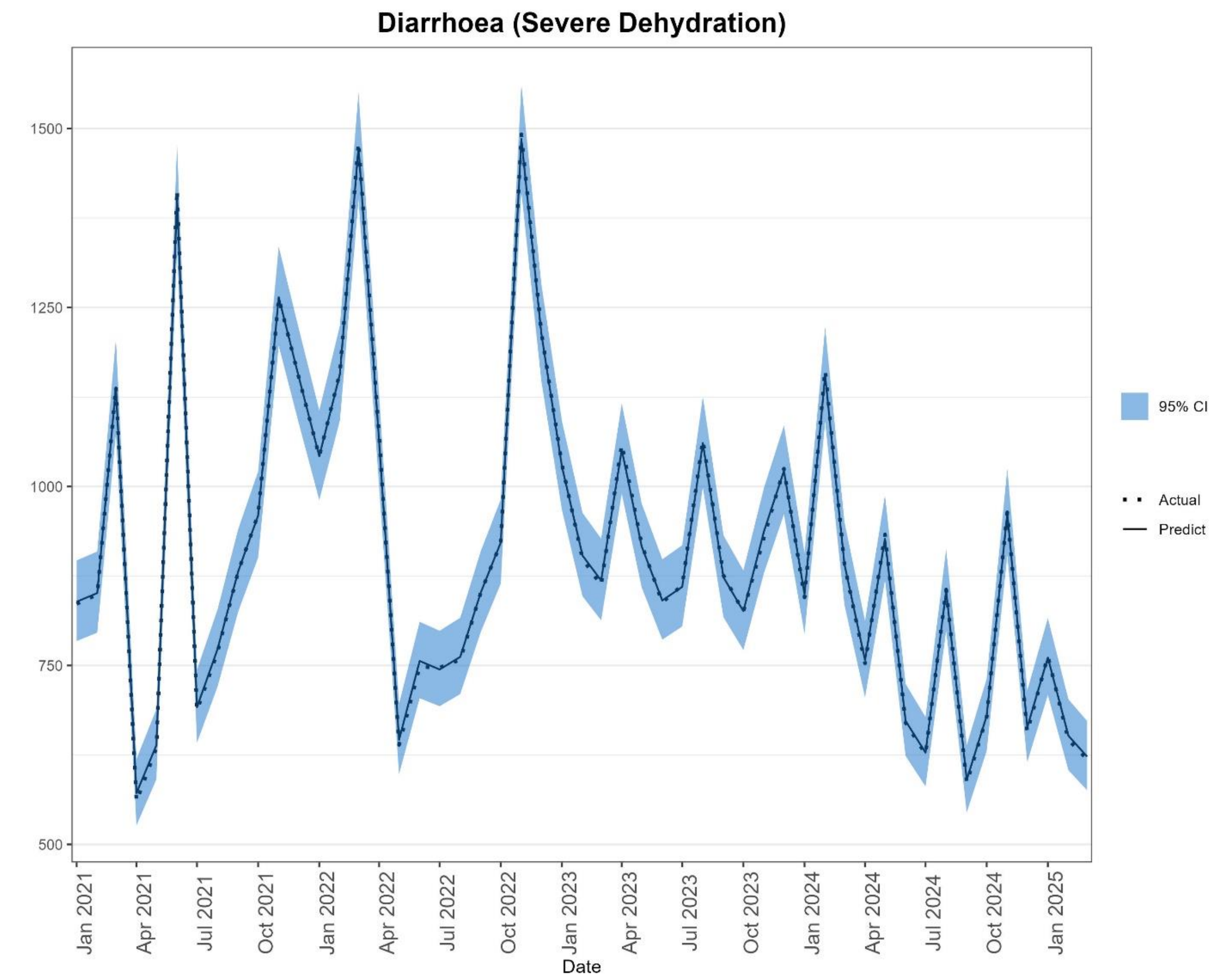

Diarrhoea (Severe Dehydration)
1500
1250
1000
750
500
Jan 2021
Apr 2021
Jul 2021
Oct 2021
Jan 2022
Apr 2022
Jul 2022
Oct 2022
Jan 2023
Apr 2023
Jul 2023
Oct 2023
Jan 2024
Apr 2024
Jul 2024
Oct 2024
Jan 2025
Date
95% CI
Actual
Predict

Babies Born in Facility Receiving Measles Vaccine

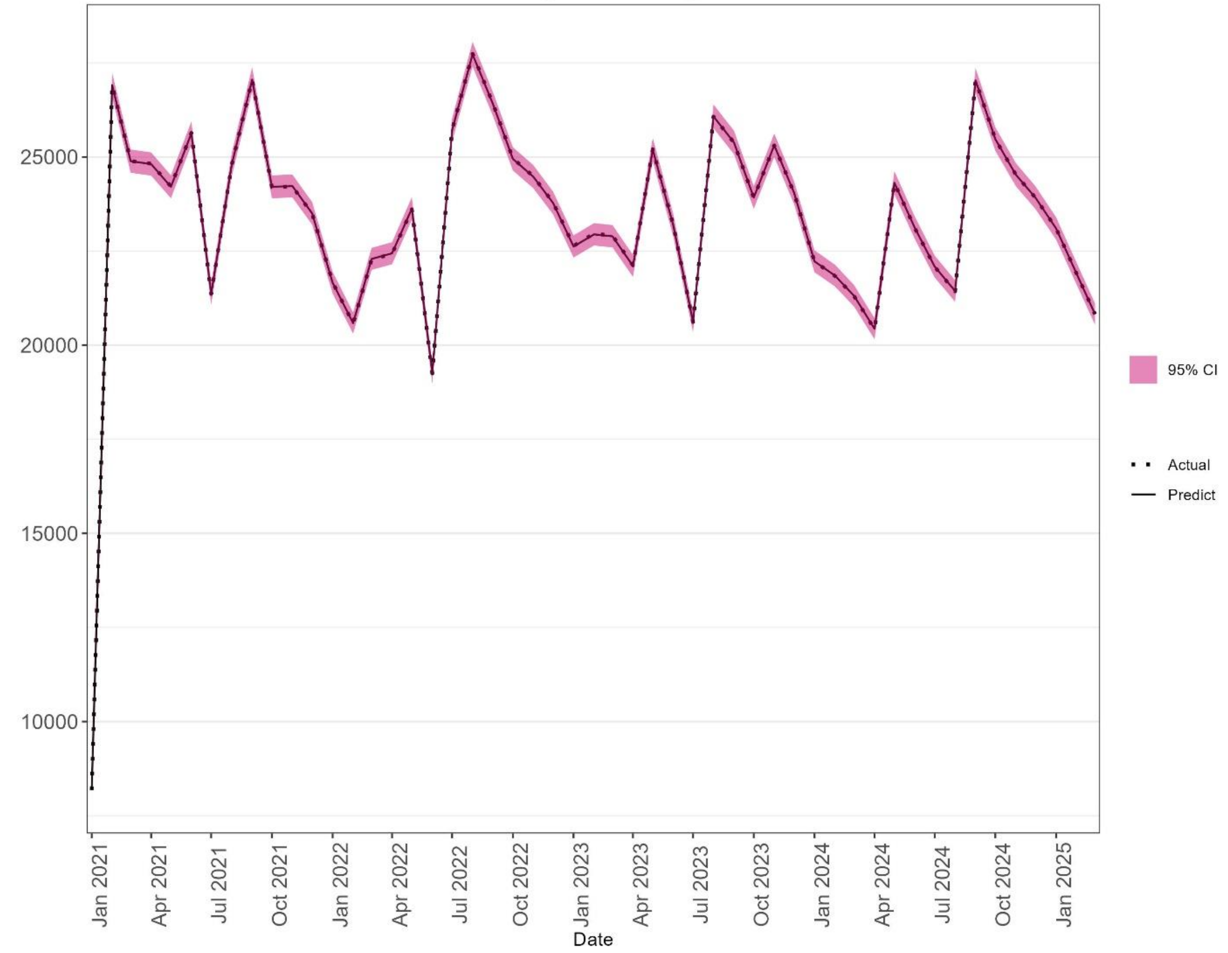

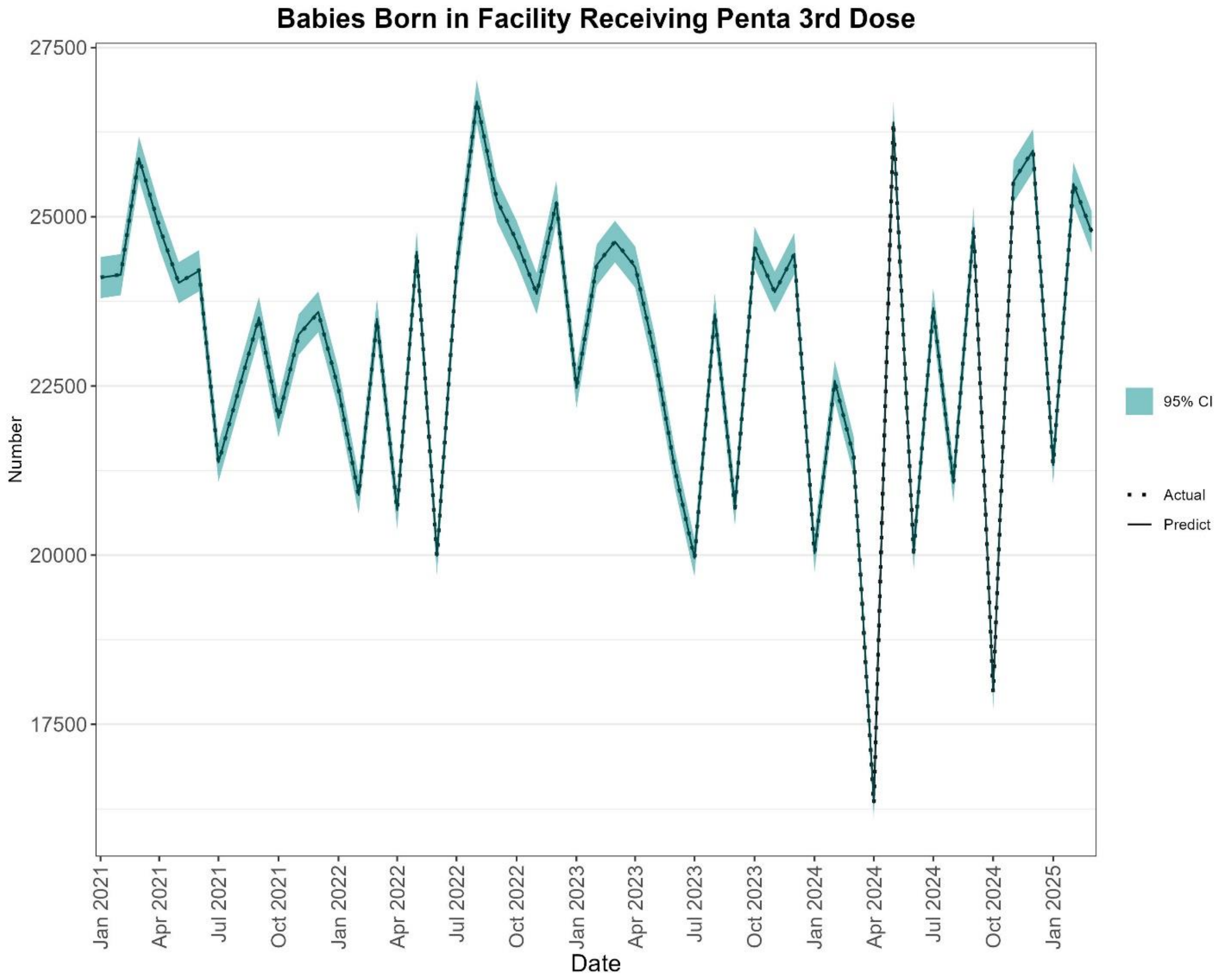

Babies Born in Facility Receiving Penta 3rd Dose
27500
25000
22500
20000
17500
Number
Jan 2021
Apr 2021
Jul 2021
Oct 2021
Jan 2022
Apr 2022
Jul 2022
Oct 2022
Jan 2023
Apr 2023
Jul 2023
Oct 2023
Jan 2024
Apr 2024
Jul 2024
Oct 2024
Jan 2025
Date
95% CI
Actual
Predict

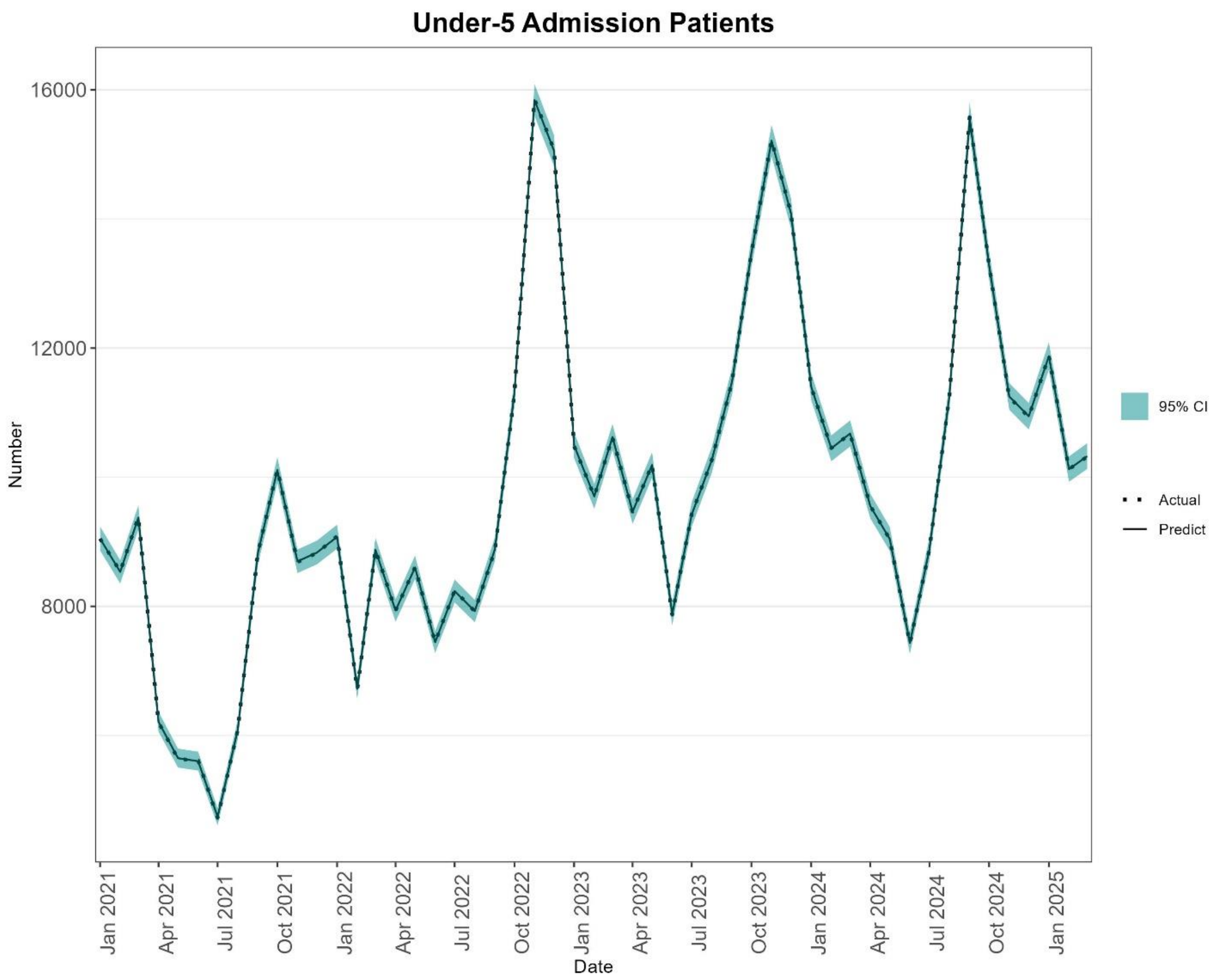

Under-5 Admission Patients
16000
12000
8000
Number
Jan 2021
Apr 2021
Jul 2021
Oct 2021
Jan 2022
Apr 2022
Jul 2022
Oct 2022
Jan 2023
Apr 2023
Jul 2023
Oct 2023
Jan 2024
Apr 2024
Jul 2024
Oct 2024
Jan 2025
Date
95% CI
Actual
Predict

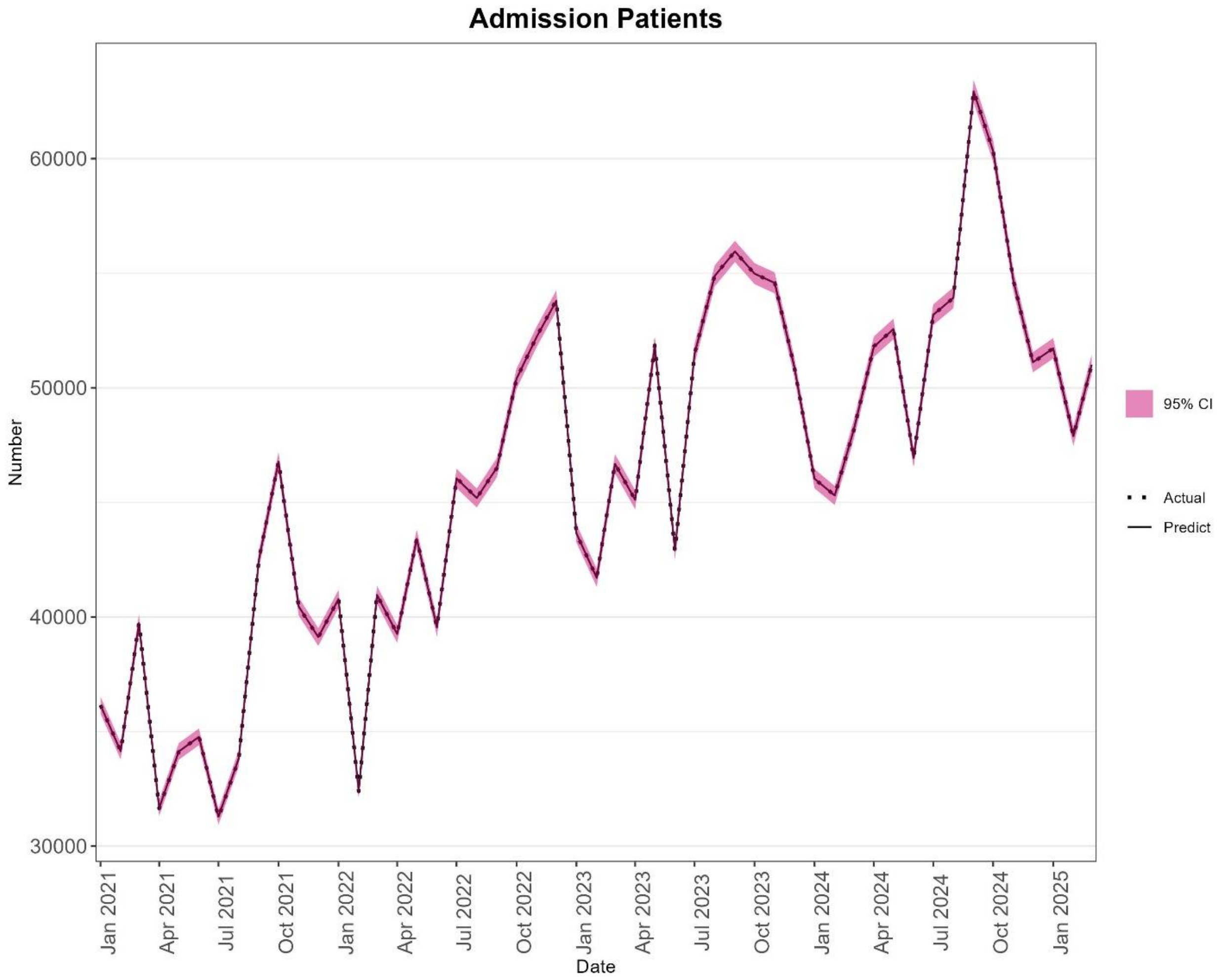

Admission Patients
60000
50000
40000
30000
Number
Jan 2021
Apr 2021
Jul 2021
Oct 2021
Jan 2022
Apr 2022
Jul 2022
Oct 2022
Jan 2023
Apr 2023
Jul 2023
Oct 2023
Jan 2024
Apr 2024
Jul 2024
Oct 2024
Jan 2025
Date
95% CI
Actual
Predict

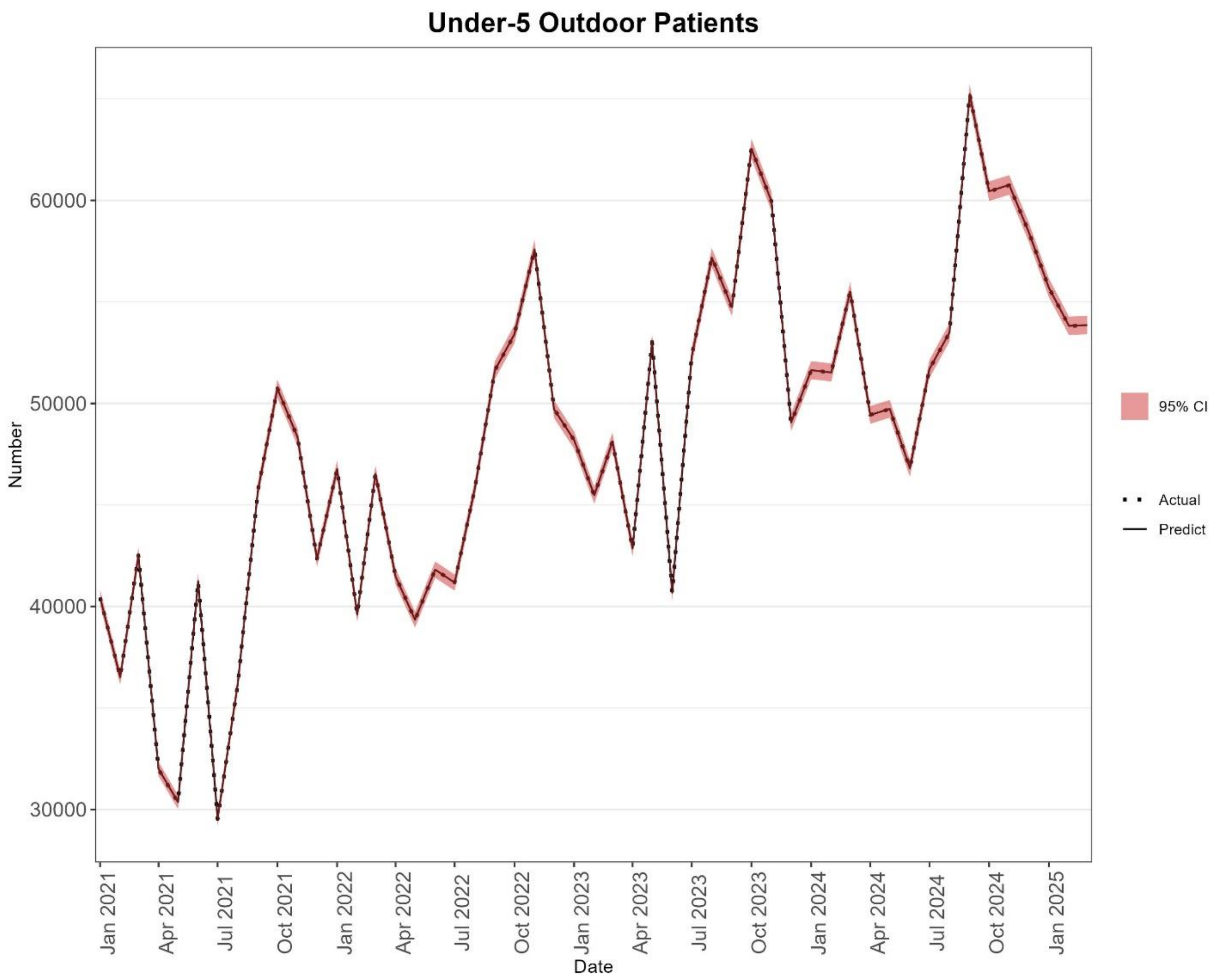
Under-5 Outdoor Patients
Number
60000
50000
40000
30000
Date
Jan 2021
Apr 2021
Jul 2021
Oct 2021
Jan 2022
Apr 2022
Jul 2022
Oct 2022
Jan 2023
Apr 2023
Jul 2023
Oct 2023
Jan 2024
Apr 2024
Jul 2024
Oct 2024
Jan 2025
95% CI
Actual
Predict

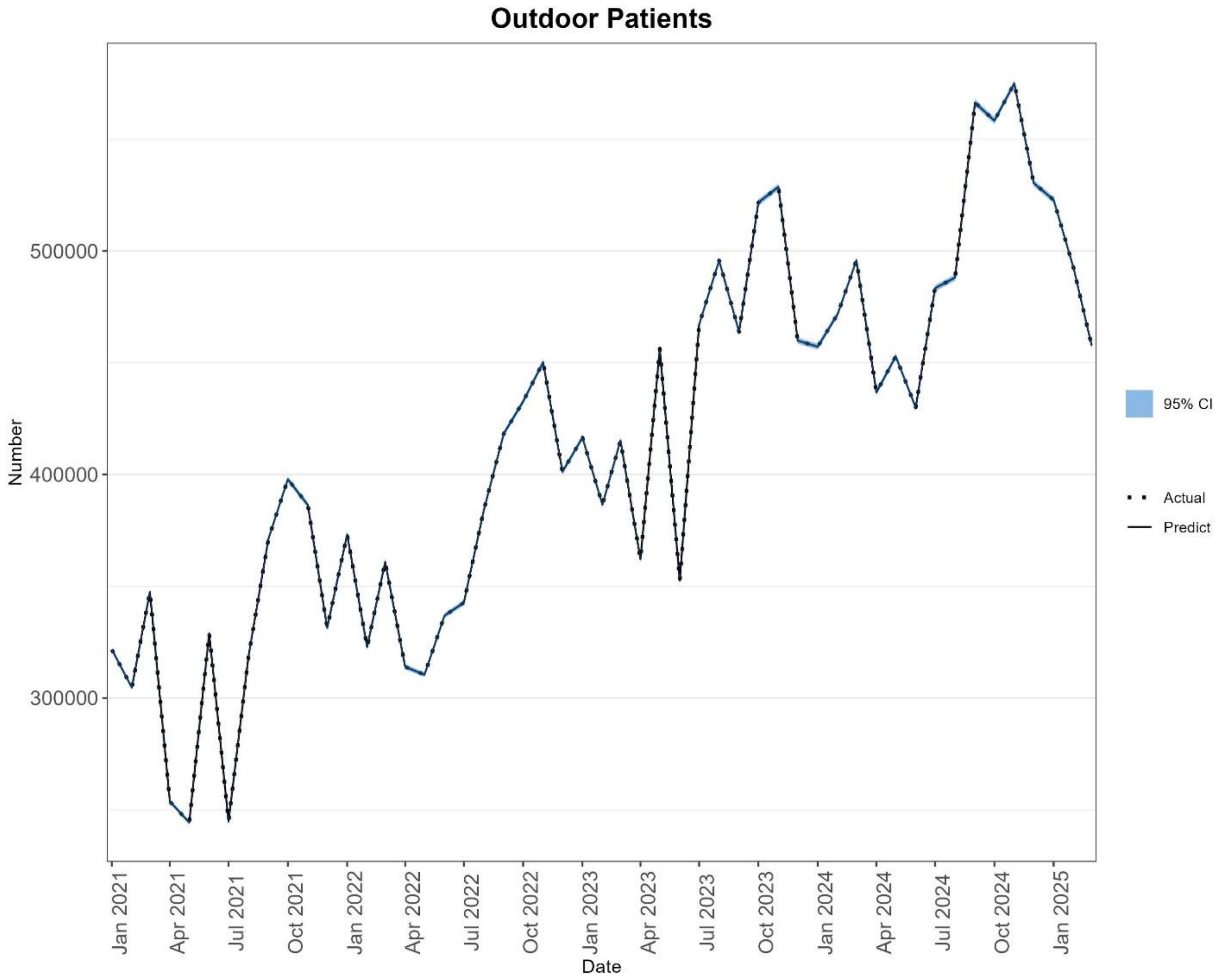
Outdoor Patients
Number
500000
400000
300000
Date
Jan 2021
Apr 2021
Jul 2021
Oct 2021
Jan 2022
Apr 2022
Jul 2022
Oct 2022
Jan 2023
Apr 2023
Jul 2023
Oct 2023
Jan 2024
Apr 2024
Jul 2024
Oct 2024
Jan 2025
95% CI
Actual
Predict
