# Supplementary material for: Using DHIS2 routine data for health system preparedness in resource-limited settings: A Bayesian predictive approach in Bangladesh
Source: PLOS Glob Public Health. 2026 Mar 3;6(3):e0005231. doi: 10.1371/journal.pgph.0005231 (PMC12956080; doi:10.1371/journal.pgph.0005231)
Supplement: S5 File — (PDF) [file pgph.0005231.s005.pdf]

S5_file: Description of study indicators

Indicator name	Definition	Unit	Time resolution	Data source	Missing (%)	Min	Max
IMCI_Pneumonia (2 months-5 years)	Number of children aged 2 months to 5 years have pneumonia	Count	Monthly	DHIS2	0%	1119	16492
IMCI_Diarrhoea: Severe Dehydration	Number of children diagnosed with diarrhoea	Count	Monthly	DHIS3	0%	283	4271
MR given 0-11m	Number of child aged 0 to11 months who received the measles–rubella (MR) vaccine	Count	Monthly	DHIS4	0%	8179	97509
Penta 3 given 0-11m Total	Number of child aged 0 to 11 months the third dose of pentavalent vaccine	Count	Monthly	DHIS5	0%	11447	104136
No. of Cesarean Section	Number of C-section delivery	Count	Monthly	DHIS6	0%	718	23421
No. of Normal Deliveries	Number of normal delivery	Count	Monthly	DHIS7	0%	1418	20568
Total No. of low birth weight babies (less than 2500)	Number of live birth with a weight of less than 2,500 grams	Count	Monthly	DHIS8	0%	65	132362
Babies born in facility received KMC	Number of newborns born in health facilities who received Kangaroo Mother Care (KMC)	Count	Monthly	DHIS9	0%	3	355
Admission Patients	Total number of patients admitted	Count	Monthly	DHIS10	0%	31248	207612
Admission patients (0-4 years)	Number of patients aged 0–4 years admitted	Count	Monthly	DHIS11	0%	4491	37352
Outdoor Patients	Total number of patients who received outpatient services	Count	Monthly	DHIS12	0%	144418	2521220
Outdoor patients (0-4 years)	Number of children aged 0–4 years who received outpatient services	Count	Monthly	DHIS13	0%	16767	303568
