# Supplementary material for: Using DHIS2 routine data for health system preparedness in resource-limited settings: A Bayesian predictive approach in Bangladesh
Source: PLOS Glob Public Health. 2026 Mar 3;6(3):e0005231. doi: 10.1371/journal.pgph.0005231 (PMC12956080; doi:10.1371/journal.pgph.0005231)
Supplement: Attachment — Submitted filename: Response to Reviewers.docx [file pgph.0005231.s007.docx]

**Response to the Editor and Reviewers**

Manuscript ID: PGPH-D-25-02646

Title: Using DHIS2 routine data for health system preparedness in resource-limited settings: A Bayesian predictive approach in Bangladesh

Dear Dr. Chowdhury and Reviewers,

We sincerely thank the Academic Editor and the reviewers for their careful evaluation of our manuscript and for the constructive and insightful comments. We are grateful for the opportunity to revise our manuscript. We have carefully addressed all comments and suggestions, and we believe that these revisions have significantly improved the clarity, rigor, and presentation of the paper.

All changes in the revised manuscript are highlighted in the tracked-changes version. Below, we provide a detailed, point-by-point response to each comment. Reviewer comments are shown in italics, followed by our responses in regular text.

Reviewers' comments and responses:

Reviewer #1: The manuscript applies accurate statistical frameworks, however, the statistical section lacks sufficient detail and transparency for reproducibility and proper evaluation. The manuscript does not provide adequate information on model specification, prior selection, model convergence, diagnostics, or validation procedures. It is unclear how the authors addressed potential overdispersion or autocorrelation beyond first order, and no measures of model performance are reported.

I recommend the authors:
*Include a clear description of priors, hyperparameters, and assumptions.*

Response: Thank you for your review. We have revised the Methods section to provide a clearer description of the prior distributions, hyperparameters, and model assumptions used in the INLA framework. Specifically, we now describe how Gaussian priors for regression coefficients were informed by preliminary linear regression estimates and their standard errors, and we explicitly state the priors assigned to the hyperparameters governing the AR(1) and seasonal components (autocorrelation parameter ρ and precision τ). We have also added the key modeling assumptions and clarified the use of posterior predictive checks for model assessment. These changes are reported in the revised manuscript

*Provide diagnostic plots and model fit indices in supplementary materials.*

Response: Thank you for your review. We assessed model adequacy using posterior predictive checks, which are a standard and robust diagnostic in Bayesian analysis. Observed data were compared with posterior predictive 95% credible intervals derived from the fitted INLA model, and the results are presented in Supplementary S4_File.

*Demonstrate the robustness of forecasts through sensitivity analyses or model comparison.*

Response: We thank the reviewer for this suggestion. While the primary objective of the study was prediction rather than formal model comparison, multiple plausible model specifications were evaluated during model development to identify the best predictive structure. Forecasts were consistent across alternative specifications, and the final model was selected based on predictive performance. We used posterior predictive accuracy to assess the model performance. This process demonstrates the robustness of the reported forecasts.

*Best of luck with your revisions :) I may not be right, but please do pay attention to these points.*

Response: Thank you very much for your valuable comments. We tried to address them and we believe these changes have substantially improved our paper.

Reviewer #2: It is an excellent work where researchers used DHIS 2 platform to have an idea on trend of service utilisation of maternal, neonatal and child health, vaccination status especially penta 3 and measles. It also explored disparities of service utilization and delivery between different divisions of Bangladesh and seasonal variations as well. This work also showed projection of different services in public health facilities . Such evidences will help policy makers for future planning , priority setting as well. Although having some limitation it might have impact in planning to handle crisis.

*Despite having good quality of this manuscript information regarding IRB clearance / permission from appropriate authority seems missing.*

Response: Thank you for your comment. This study used routinely collected secondary administrative data attained from the Directorate General of Health Services (DGHS), Bangladesh. The dataset included aggregated, anonymised for individual-level information. As the study involved analysis of secondary data and posed no risk to individuals, formal ethical approval was not required.

Reviewer #3: This manuscript, “Using DHIS2 routine data for health system preparedness in resource-limited settings: A Bayesian predictive approach in Bangladesh”, presents a well-designed and policy-relevant study applying Bayesian log-linear Poisson regression to forecast service utilization trends using DHIS2 data from Bangladesh. The study is technically rigorous, original, and offers important implications for data-driven health system planning and preparedness in LMICs.

The topic aligns strongly with PLOS Global Public Health’s scope, focusing on strengthening health systems and improving resource allocation through innovative data use. The paper is well-structured and methodologically sound, and the conclusions are appropriately supported by the data. The authors provide clear evidence of rising health service utilization and highlight regional disparities, which are both timely and significant findings for the Bangladeshi health system.

The following minor revisions are suggested primarily to improve clarity, reporting precision, and conciseness.

*1. Abstract: The abstract is informative but overly detailed with repeated numeric projections.
Suggested Action: Summarise the main findings more concisely and reduce numeric repetition.*

Response: Thank you for your comment. We have summarized the findings “The analysis revealed rising trends across most service areas relative to 2024 levels. Kangaroo Mother Care (KMC) has the highest projected expansion, with coverage forecast to rise by over 75% by 2026. Over the same time period, outpatient visits and pneumonia treatment are also expected to increase by about 30%. More moderate increases are seen in low birth weight (LBW) deliveries, caesarean sections, and normal deliveries. Notable regional disparities persist, with Dhaka and Chittagong showing the highest service utilization, while Barisal and Sylhet consistently report the lowest levels”.

*2. Methods (Model Validation): The analytical approach is well-explained, but details on model validation or diagnostic checks are missing.*
Suggested Action: Briefly describe how model fit or predictive performance was assessed (e.g., residual checks, posterior predictive accuracy, etc).

Response: Thank you for your comment. We added a posterior predictive accuracy for model performance in S4_File.

*3. Data Availability Statement: The data are restricted under DGHS permission. While this is reasonable, the statement could more clearly justify restricted access.*

Suggesred Action: Expand the justification for restricted access and outline how data can be requested formally by indicate contact address. Alternatively deposit anonymised data in a public repository.

Response: Access to this data will be granted to qualified researchers upon reasonable request in DGHS (contact: dghs.portal.gov.bd). We are also able to share the anonymised data which was used in this analysis.

*4. Language and Clarity: The manuscript is well-written overall but includes some long sentences and minor grammatical inconsistencies.
Suggested Action: Edit for brevity and clarity. Consider light professional copy editing.*

i) Examples of Long or Complex Sentences
*a). Page 2, lines 33-38 (Abstract) “**By analysing service utilization trends and projecting future service volume at national and regional level, we aim to improve region-specific health planning and promote more efficient and equitable health service provision.”
Suggested Edit: Long but clear; could be split after “level.” → “**By analysing service utilization trends and projecting future service volume at national and regional levels, we aim to improve region-specific health planning. This can promote more efficient and equitable service provision.”*

Response: Thank you for comment. We simplified the long sentence as suggested.

b) *Page 6, lines 120-126 “**Resource allocation decisions in this context are frequently made without sufficient data-driven insights, leading to inefficiencies, supply shortages, and gaps in service delivery.”
Grammatically correct but wordy; could shorten to: “**Resource allocation decisions often lack data-driven insights, causing inefficiencies and service gaps.”*

Response: Thank you for comment. We simplified the long sentence as suggested.

c) *Page 7, lines 155-162 “Routine health information systems, such as the District Health Information Software 2 (DHIS2), offer a promising opportunity to strengthen health system preparedness and planning (16). As a widely implemented digital platform for collecting, managing, and analyzing health data, DHIS2 has been adopted in over 60 countries and serves as a backbone for health service reporting in Bangladesh.”
These two sentences are dense and repetitive; merging or trimming one clause would improve flow.*

Response: Thanks for your comments. We trimmed the “As a widely implemented digital platform for collecting, managing, and analyzing health data, DHIS2 has been adopted in over 60 countries and serves as a backbone for health service reporting in Bangladesh.”

d) *Page 10, lines 270-278 “**We employed a Bayesian approach to forecast service utilisation. As recommended by earlier research, a log-linear model was used to fit the model and forecast future health services, assuming that the number of persons using health services at the 't' time point follows a Poisson distribution (20).”
Repetition of “model”; could simplify to: “**Following prior research, we used a Bayesian log-linear Poisson model to forecast future health service use.”*

Response: Thank you for comment. We simplified the long sentence as suggested.

e) *Page 17, lines 535-542 (Discussion) “In addition to the overall upward trend in facility-based deliveries, our study revealed a clear seasonal pattern, with deliveries peaking between September and December.*

*This finding is consistent with previous research indicating a strong seasonality in childbirth patterns in Bangladesh, where nearly twice as many births occur in December compared to July (30).”
Long but grammatically fine; may read better if split after “December.”*

Response: *Thank you for comment. We have revised the sentence by splitting it after “December” to improve readability and flow, while retaining the original meaning and supporting reference.
f) Page 20, lines 640-647 “**Without proactive investments, existing health facilities may become overstretched, especially in high-demand regions like Dhaka and Chattogram. Bangladesh’s healthcare system continues to face challenges related to limited bed capacity, shortages of trained personnel, and inadequate infrastructure, particularly in peripheral and rural areas.”
Sentence length acceptable but repetitive phrase “continues to face challenges related to” could be simplified: “**The healthcare system remains constrained by limited bed capacity, staff shortages, and inadequate infrastructure.”*

Response: Thank you for this suggestion. We have simplified the phrasing as recommended by revising the sentence to: “The healthcare system remains constrained by limited bed capacity, staff shortages, and inadequate infrastructure,” reducing repetition while preserving the original meaning.
ii) Examples of Minor Grammatical or Stylistic Inconsistencies.

*a) Page 5, line 102 “The timely delivery of treatment is directly affected by the efficient use of medical resources, which is an increasing problem for healthcare services (2).”
Issue / Suggestion: Awkward phrasing; consider “inefficient use … is an increasing problem.”*

Response: Thank you, we have revised as suggested.

b*) Page 7, line 148 “The country is organized into eight major administrative divisions: Barishal, Chattogram, Dhaka, Khulna, Rajshahi, Rangpur, Mymensingh, and Sylhet.”
Issue / Suggestion: Consistent; no issue—listed here because commas occasionally vary elsewhere (“and”)—ensure consistent punctuation style.*

Response: Thank you for your review. We have changed all “, and” to “and”

c) *Page 9, line 220 “During regular service delivery, healthcare practitioners document health services in real time, which are then uploaded daily and some cases monthly to the DHIS2 platform and recorded into facility registers (19).”
Issue / Suggestion: Missing preposition: “in some cases monthly.”*

Response: Thank you for your review, we have revised as suggested.

*d) Page 11, line 310 “Projections indicate that the number of normal deliveries will rise by approximately 6% in 2025 and 10% in 2026 compared to 2024 (Fig 1).”
Issue / Suggestion: Numerically correct but check for consistent use of “%” symbol (some later sentences spell out “percent”).*

Response: Thank you for noting this. We have checked the manuscript and ensured consistent use of the “%” symbol throughout, including in this sentence and all subsequent references, replacing any instances where “percent” was spelled out.


*e) Page 18, line 585 “Due in large part to a lack of knowledge about the dangers of needless C-sections, many families are choosing surgical procedures over natural births, which is why the number of normal deliveries is declining.”

Issue / Suggestion: Stylistic: avoid causal phrase “which is why” in formal academic writing → “leading to a decline in normal deliveries.”*

Response: Thank you so much for your comment. We have revised as suggested.

*f) Page 23, line 730 “Service utilisation is also driven by governments and partners’ activities to increase demand of and access to services.”
Issue / Suggestion: Slightly awkward phrase “demand of” → should be “demand for.”*

Response: Thank you so much for your comment. We have revised as suggested.

*g) Page 25, line 805 “These include the persistently low service coverage during daily hospital visits and the admission data set in Sylhet and Barisal compared to high utilization in Dhaka and Chattogram.”
Issue / Suggestion: “Admission data set” unclear; perhaps “admission data show low service coverage.”*

Response: Thank you so much for your comment. We have changed it.

*h) Page 26, line 820 “Leveraging platforms like DHIS2 for real-time forecasting and operational decision-making can transform the country’s ability to respond to rising and shifting healthcare needs.”
Issue / Suggestion: Fine but ensure hyphen consistency (“real-time” used elsewhere as “real time”).*

Response: Thank you so much for your comment. We have changed it.


*5. References: Minor inconsistencies in citation formatting (capitalization, punctuation).
Suggested Action: Standardize all references per PLOS Global Public Health guidelines.*

Response: Thank you so much. We have changed the inconsistencies.

*6. Figures and Supplementary Files: Figures are clear and relevant, but figure legends could be slightly expanded for standalone interpretation.
Suggested Action: Ensure legends briefly summarize key findings for each figure.*

Response: Thank you for this comment. We have carefully considered the suggestion to expand the figure legends. However, we have chosen to retain concise legends, as detailed interpretations of each figure are already clearly presented in the Results section. This approach avoids redundancy and maintains consistency with the journal’s preference for integrating figure interpretation within the main text, while still ensuring that figures are adequately referenced and understandable.

*Reviewer #4: The manuscript addresses a very important and novel topic using DHIS2 routine data to assess maternal, child, and newborn health indicators in Bangladesh. The seasonal patterns in child health outcomes are highlighted well, and the indicators selected are appropriate.
Methods: Data cleaning procedures are unclear, including handling of missing values or inconsistencies.*

*Please describe how missing data were managed. Consider a table summarizing all indicators, definitions, and units for clarity.*

Response: Thank you for this comment. Prior to analysis, the dataset was systematically assessed for outliers, internal inconsistencies, and missing values. Completeness checks were performed for all study variables, and no missing data were identified. To improve clarity, we have added a detailed variable description table, including indicator definitions and units, in Supplementary File S5.

*Analysis: The approach for modeling seasonality is not clearly described. Please clarify whether Fourier terms, dummy variables, or other methods were used.*

Response: Thank you for this comment. For the monthly time-series data, seasonality was modeled using a cyclic seasonal component with a 12-month period, implemented through the INLA seasonal model. This approach captures recurring intra-annual patterns while accounting for temporal dependence in the data.

*Results/Figures: In Figure 1, the colors for “observed 2024” and “predicted 2025” are too similar; use more distinct colors for readability.*

Response: Thank you for your comment. We have updated the figure.

*Language and Formatting: Typo in Discussion: “utlisation” instead of “utilization.” Some references are old, and journal formatting is inconsistent. The manuscript uses “Chittagong” and “Chattogram” interchangeably, clarify that they refer to the same division.*

Response: Thank you for these observations. We have corrected the typographical error by replacing “utlisation” with “utilization.” We have also reviewed and updated the reference list where appropriate and ensured consistency with the journal’s formatting requirements. In addition, we have standardized the terminology by using “Chattogram” throughout the manuscript and clarified that it refers to the same administrative division previously known as “Chittagong.”
